# Unraveling Cross-Ring Dissociation Mechanisms of Hexoses in Collision-Induced Dissociation

**DOI:** 10.1021/acs.jpca.6c00953

**Published:** 2026-04-20

**Authors:** Hock-Seng Nguan, Yen-Ting Lin, Chi-Kung Ni

**Affiliations:** † Institute of Atomic and Molecular Sciences, 38017 Academia Sinica, P.O. Box 23-166, Taipei 10617, Taiwan; ‡ Department of Chemistry, National Tsing Hua University, Hsinchu 30013, Taiwan; § Molecular Science and Technology, Taiwan International Graduate Program, Academia Sinica, Taipei 115201, Taiwan

## Abstract

Understanding the fragmentation mechanisms of carbohydrates in mass spectrometry is essential for interpreting mass spectra and accurately identifying carbohydrate structures. In this study, we used β-glucose, methyl β-mannose, and four disaccharides—Manβ-(1→2)-Manβ, Manβ-(1→3)-Manβ, Manβ-(1→4)-Manβ, and GlcNAcβ-(1→2)-Manα—as model systems. Combining high-level quantum chemistry calculations with collision-induced dissociation mass spectrometry experiments, we performed an extensive investigation of the cross-ring dissociation mechanisms of hexoses under collision-induced dissociation. In addition to the dissociation pathways proposed in previous studies, which account for the major cross-ring fragments, we identified new mechanisms that explain the minor cross-ring fragments. The newly proposed pathways begin with the same initial step as that of the previously reported mechanism: a ring-opening reaction initiated by hydrogen transfer from O1 to O5, followed by cleavage of the O5–C1 bond. The key distinction is that the established mechanism proceeds through a 1,2-hydrogen shift, whereas the newly identified pathways involve alternative hydrogen-shift processes. The low energy barrier associated with the 1,2-hydrogen shift explains the formation of major cross-ring fragments, while the higher energy barriers associated with the alternative hydrogen shifts account for minor fragments. Both the previously reported and newly proposed mechanisms occur only for hexose at the reducing end. The energy barriers for cross-ring dissociation of the hexose not at the reducing end are significantly higher, indicating that the hexose not at the reducing end contributes far less to cross-ring fragmentation. Applying these newly elucidated mechanisms to *N*-glycans enables the interpretation of cross-ring fragments that would otherwise be misassigned as a mixture of different *N*-glycan linkages in the sample.

## Graphical abstract

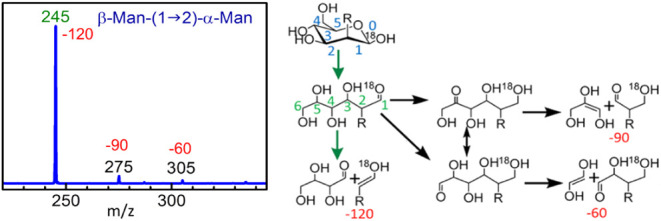

## Introduction

Carbohydrates are one of the major classes of biomolecules, alongside proteins, lipids, and nucleic acids. They serve as the primary sources of energy for organisms. At the cellular level, carbohydrates play essential roles in numerous biological processes, including cell–cell signaling, immune responses, and protein folding.^1^ Carbohydrate structures are highly diverse due to the presence of multiple chiral centers on most of their carbon atoms and the abundance of hydroxyl groups that can form multiple glycosidic bonds. Characterizing carbohydrate structures is, therefore, challenging, as the vast structural diversity and prevalence of stereoisomers create a significant bottleneck in advancing glycoscience research.

Several analytical approaches have been developed to characterize carbohydrate structures, including nuclear magnetic resonance (NMR) spectroscopy,^2,3^ enzymatic digestion methods,^4^ ion mobility spectrometry,^5–7^ infrared action spectroscopy,^8–10^ and mass spectrometry. Both NMR and enzymatic digestion require relatively large sample quantities, which are impractical for biological samples that are difficult to obtain in bulk. Furthermore, the specificity and availability of the appropriate enzymes can limit the utility of enzymatic digestion in structural elucidation. Techniques such as ion mobility spectrometry and infrared action spectroscopy can differentiate carbohydrate isomers based on their distinct ion mobilities or IR spectra; however, both methods usually rely on the availability of standard compounds.

Tandem mass spectrometry is the most widely used method for carbohydrate structural determination because of its high sensitivity.^11–20^ In typical tandem mass spectrometry, carbohydrates are dissociated into fragment ions to generate MS^2^ spectra. Structural assignments are then made by comparing the MS^2^ spectra to those of known carbohydrate standards in databases or by inferring the structures directly based on the dissociation mechanisms. Comparison to the standards is limited to the available standards in the database. Determining complete structures solely from MS^2^ spectra is often difficult, because many carbohydrate isomers produce highly similar fragment patterns. Multistage tandem mass spectrometry is therefore required for complete structural determination. In multistage tandem mass spectrometry, one of the fragment ions produced in MS^2^ is isolated and subjected to further dissociation to produce an MS^3^ spectrum. This process can be repeated, with a fragment from each stage selected for subsequent dissociation, until the desired MS^n^ spectrum is obtained or until the number of ions left in the mass spectrometer becomes too small to proceed. Carbohydrate structures can then be elucidated from these spectra.^17–20^ The derivation of carbohydrate structures from tandem mass spectra depends on a clear understanding of their dissociation mechanisms in the mass spectrometer. In principle, with sufficient knowledge of these mechanisms, carbohydrate structures can be accurately determined from tandem mass spectra without the need for a comparison to spectral libraries of standards.

Among the various fragmentation methods, collision-induced dissociation (CID) is one of the most commonly used techniques in tandem mass spectrometry, and quantum-chemistry calculations are frequently employed to investigate the associated dissociation mechanisms. Since the 1990s, theoretical computational approaches such as semiempirical methods and Hartree–Fock calculations have been used to study the conformations and CID-induced fragmentation of disaccharides^21–24^ and oligosaccharides.^25,26^ Subsequently, more accurate methods—including density functional theory (DFT) and MP2—have been applied to investigate CID mechanisms in mono- and disaccharides.^27–31^ In CID tandem mass spectrometry, the internal energy is gradually accumulated via repeated collisions. Carbohydrates possess highly diverse conformational landscapes due to variations in glycosidic dihedral angles and changes in ring conformations. The energy barriers between these conformers are typically much lower than those for bond dissociation. As a result, the energy often reaches isomerization thresholds first, allowing carbohydrates to interconvert readily among many conformers before achieving sufficient energy for fragmentation. Dissociation starts when the molecular internal energy reaches the levels of the dissociation pathways with low energy barriers. Because the lowest-energy dissociation pathways are not necessarily accessed directly from the lowest-energy conformers, extensive conformational sampling is essential to avoid bias that would arise from considering only a small number of stable conformers. A variety of conformational sampling strategies have been developed, including random structural searches,^30–32^ metadynamics,^33^ and machine-learning-based approaches.^34,35^

Cross-ring dissociation is a major dissociation pathway of hexose sodium ion adducts in CID. In our previous studies,^27,29–33^ we demonstrated that cross-ring dissociation of singly charged hexose sodium ion adducts in CID proceeds through a sequence of reactions. The process, as illustrated in Figure 1, begins with a ring-opening reaction involving hydrogen transfer from O1 to O5, followed by cleavage of the O5–C1 bond to open the ring. After ring-opening, the pathway may proceed directly through a retro-aldol reaction or, alternatively, undergo additional hydrogen shifts that change the position of the carbonyl group before the retro-aldol reaction occurs. These mechanisms account for the major and minor cross-ring fragments typically observed in the CID spectra of singly charged sodium ion adducts of hexose monosaccharides. For example, in aldo-hexose monosaccharides, the dominant cross-ring fragment corresponds to the loss of a neutral species with m = 60, while the less intense fragments correspond to losses of m = 90 (Figure 2a). In contrast, for keto-hexose monosaccharides, the predominant cross-ring fragment is the loss of m = 90, with m = 60 appearing as minor fragments (Figure 2a). These differences are consistent with the mechanistic model in Figure 1a: the major fragment arises from a direct retro-aldol reaction following ring-opening, whereas the minor fragments require additional hydrogen shifts prior to retro-aldol cleavage. The distinct patterns of major cross-ring fragments thus provide a straightforward method for distinguishing aldo-hexoses from keto-hexoses.^36^ In addition to the aforementioned dissociation reactions, secondary dissociation, where a fragment generated from dissociation retains sufficient internal energy to undergo further dissociation without additional excitation, may occur. Loss of neutral m = 60 in the secondary dissociation after the loss of m = 60 in the primary dissociation, resulting in the loss of neutral m = 120, is a common secondary dissociation, as illustrated for the hexose in Figure 1a. However, the intensity of the fragments from secondary dissociation is typically low. For example, the fragment *m*/*z* 83 in Figure 2a is generated from secondary dissociation and exhibits low intensity.

**Figure 1:**
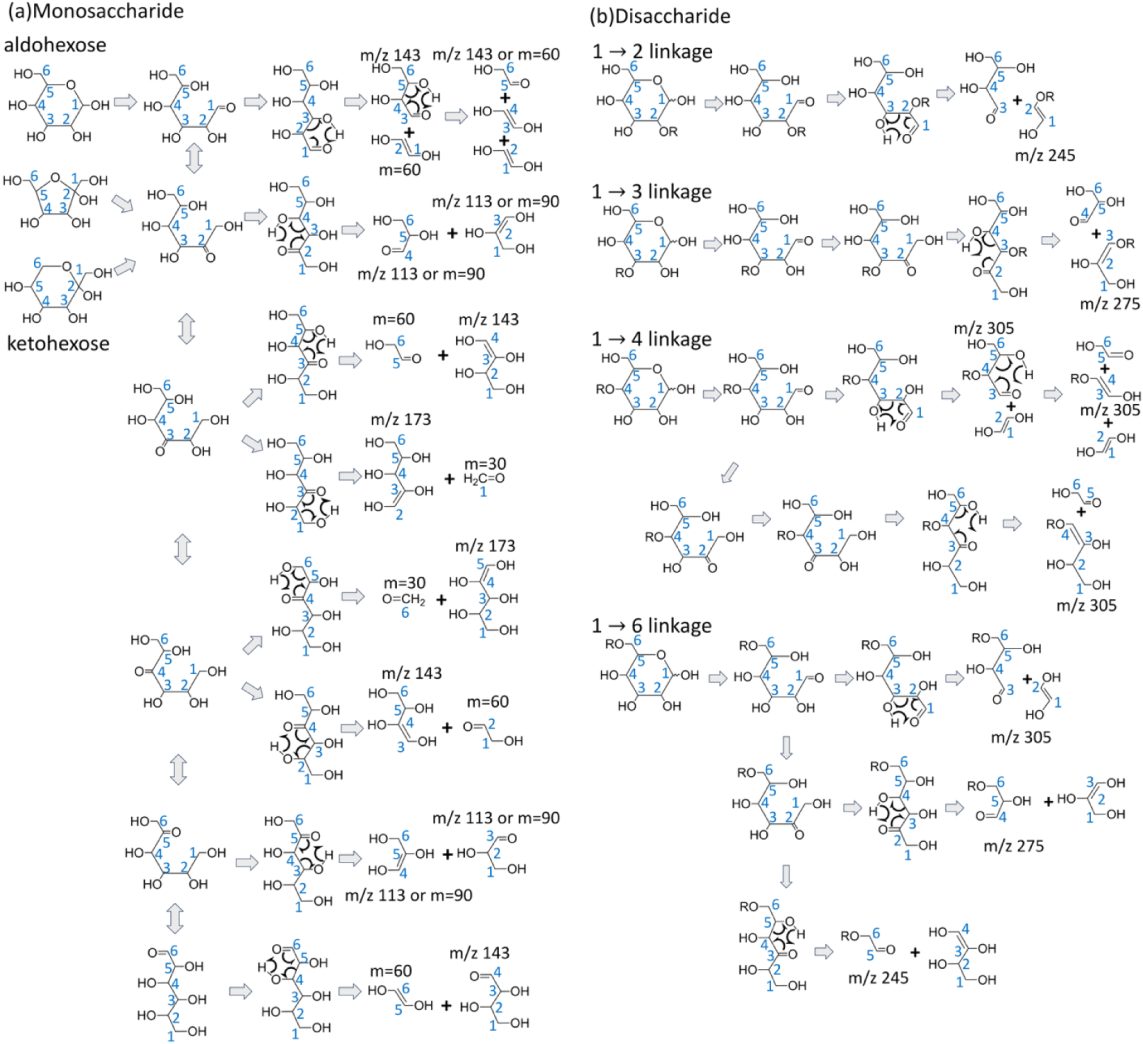
(a) Cross-ring dissociation mechanisms of singly charged aldo-hexose and keto-hexose sodium ion adducts. Position of the sodium ion is not shown. *m*/*z* values represent the mass-to-charge ratio of the fragments with sodium ion adducts. (b) Application of the dissociation mechanisms in Figure 1(a) to the singly charged sodium ion adducts of disaccharides and oligosaccharides with an aldo-hexose at the reducing end. Only oligosaccharides in which the aldo-hexose at the reducing end connects to the other sugars through one glycosidic bond are shown. For oligosaccharides in which the aldo-hexose at the reducing end connects to the other sugars through more than one glycosidic bond (branched oligosaccharides), please see ref.^36^. For keto-hexose at the reducing end, see ref.^36^.

**Figure 2:**
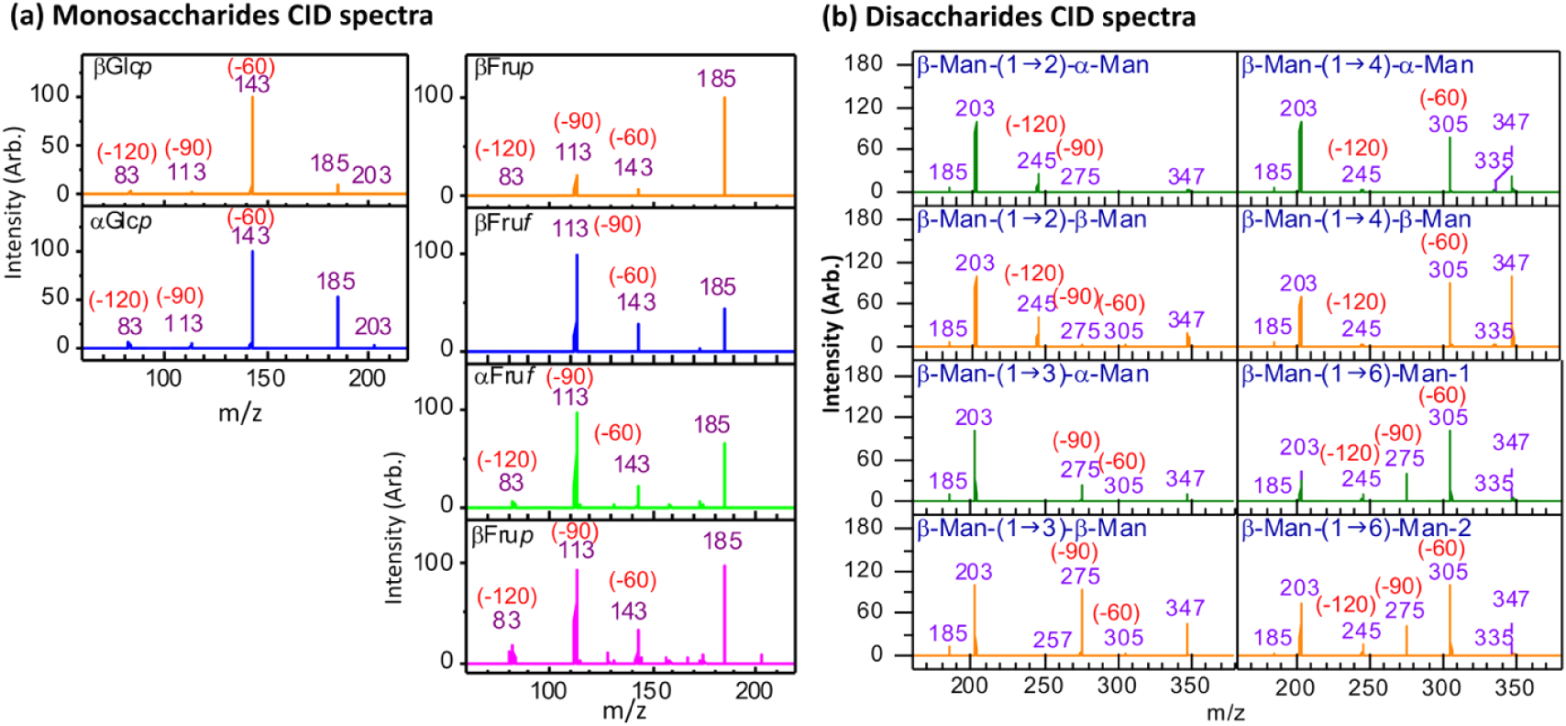
(a) Singly charged glucose (an aldo-hexose) and fructose (a keto-hexose) sodium ion adduct CID spectra. (b) CID spectra of singly charged mannose disaccharide sodium ion adduct with different linkages.

The same dissociation mechanisms can be extended from the singly charged sodium ion adducts of monosaccharides to those of disaccharides and larger oligosaccharides.^17–20,36^ For example, the CID spectra of an aldo-hexose located at the reducing end of disaccharides or oligosaccharides with different glycosidic linkages exhibit characteristic cross-ring fragments, as illustrated in Figure 1b. According to these mechanisms, disaccharides with a 1→2 linkage show a dominant neutral loss of m = 120, corresponding to the fragment at *m*/*z* 245 in Figure 2b; those with a 1→3 linkage show a neutral loss of m = 90, corresponding to the fragment at *m*/*z* 275 in Figure 2b; those with a 1→4 linkage show a neutral loss of m = 60, corresponding to the fragment at *m*/*z* 305 in Figure 2b; and those with a 1→6 linkage exhibit neutral losses of m = 60, 90, and 120, corresponding to fragments at *m*/*z* 305, 275, and 245 in Figure 2b. These cross-ring dissociation pathways rationalize the major cross-ring fragments observed for any disaccharide or oligosaccharide containing an aldo-hexose at the reducing end based on the mechanisms shown in Figure 1b, and they are used in tandem mass spectrometry for glycosidic bond linkage determination. In addition to the major cross-ring fragments, minor cross-ring fragments, including the neutral losses of m = 60 and 90 in 1→2 linkage, m = 60 and 120 in 1→3 linkage, and m = 90 and 120 in 1→4 linkage, are also observed. The aforementioned mechanisms do not account for these minor cross-ring fragments observed in CID spectra, except that the loss of neutral m = 120 in a 1→4 linkage may result from secondary dissociation, as illustrated in Figure 1b for the 1→4 linkage. Although the intensities of these minor cross-ring fragments in Figure 2b are very low, as will be demonstrated in a later section, some oligosaccharides produce non-negligible “minor” cross-ring fragments that require proper explanation. Without such an understanding, these fragments could be misinterpreted as evidence for mixtures containing oligosaccharides with different linkages, leading to errors in structural determination.

In this study, we examined singly charged sodium ion adducts of β-glucose, methyl β-mannose, and a series of disaccharides: Manβ-(1→2)-Manβ, Manβ-(1→3)-Manβ, Manβ-(1→4)-Manβ, and GlcNAcβ-(1→2)-Manβ as model systems to investigate the cross-ring dissociation pathways responsible for the minor cross-ring fragments observed in Figure 2b. We combined extensive conformational searching of reactant structures and transition states with high-level quantum-chemical calculations and CID experimental measurements to elucidate these dissociation mechanisms. Our results show that, in addition to the major cross-ring fragments originating from cleavage of hexose at the reducing end, as illustrated in Figure 1, the minor cross-ring fragments also arise predominantly from dissociation of the hexose at the reducing end. Application of the newly discovered mechanisms to *N*-glycans enables an accurate determination of *N*-glycan structures.

## Methodology

### Experimental Methods

The purity of the compounds was first verified by using high-performance liquid chromatography (HPLC). For HPLC-electrospray ionization (ESI)-MS experiments, 5 μL of the sample, dissolved in deionized water (DIW) at a concentration of 2 × 10^–4^ M, was injected into the HPLC system (Dionex Ultimate 3000, Thermo Fisher Scientific Inc., Waltham, MA, USA) with a Hypercarb column (100 × 2.1 mm, particle size: 3 μm, Thermo Fisher Scientific Inc.). The eluents were then directly injected into the ESI source of a linear ion trap mass spectrometer (LTQ XL, Thermo Fisher Scientific Inc.). A NaCl solution (2 × 10^–4^ M NaCl dissolved in methanol: DIW = 50:50 solution (v/v%)) was added to the HPLC eluents before entering the ESI source. The mobile phase of HPLC consisted of DI water (solution A) and HPLC-grade acetonitrile (solution B). The gradient of the mobile phase was changed linearly from A = 100% and B = 0% at t = 0 min to A = 90% and B = 10% at 30 min, with a flow rate of 200 μL/min. For the direct injection of ESI emission into the mass spectrometer, the analytes were dissolved in a methanol/DIW (50:50, v/v) solution at 2 × 10^–4^ M with 2 × 10^–4^ M NaCl and loaded into the ESI source of the same linear ion trap mass spectrometer. The temperature of the ESI source and transfer capillary was set to 280 °C and 350 °C, respectively. The voltages of the ion spray, capillary and tube lens were set to 4000 V and 80 V, respectively. Helium gas was used as both the buffer gas and the collision gas in the ion trap. The linear ion trap settings were: 1 u of isolation width, 30 ms of activation time, 30% of normalized collision energy, and 0.25 of Q value in positive mode.

### Computational Methods

The calculations began with a search for the reactant states of the target reactions. This was carried out using metadynamics-enhanced sampling in molecular dynamics (MD) simulations. We employed the multiwalker well-tempered version of metadynamics,^37^ in which atomic and molecular interaction potentials and forces were calculated using the density functional tight-binding (DFTB) method. For the metadynamics simulations involving cyclic hexoses, the puckering index^38^ for each cyclic structure was used as a collective variable (CV), a structural descriptor employed to enhance sampling in metadynamics. For disaccharides, additional CVs consisting of two dihedral angles describing the relative orientations of the mannose units around the glycosidic bond were included. For linear saccharides, either in monosaccharide form or at the reducing end of disaccharides, CVs comprising five dihedral angles corresponding to the five C–C bonds were used. In all cases, the CV describing sodium ion–oxygen coordination numbers was applied to account for the various possible sodium ion positions. To enhance sampling of diverse structures, three separate metadynamics simulations were conducted with different energy increments: 0.001, 0.01, and 0.1 hartree. Each simulation was run for 35 ps. The resulting structures from all three simulations were combined and screened to remove highly similar structures while retaining distinct conformers, using the approach proposed by Ballester and Richards.^39^

The distinct structures obtained from the metadynamics sampling were evaluated to determine whether they could serve as suitable reactant-state candidates, hereafter referred to as reaction candidates for the target reaction. A structure was considered a reaction candidate if it was likely to approximate a low-energy transition state (TS) of the reaction of interest. Since all reactions under consideration involve hydrogen-atom transfer from an oxygen or carbon atom to another oxygen or carbon atom, the structural criteria for selecting reactant candidates that could lead to low-lying TSs were defined as follows: (1) The sodium ion must coordinate to the oxygen atom of either the H-atom donor or acceptor with a Na^+^–O distance of less than 2.5 Å. (2) The distance between the donor and acceptor atoms must fall within the range of 3–3.5 Å. The first criterion ensures that sodium ion coordination can lower the barrier for hydrogen transfer, while the second ensures that the donor–acceptor distance is short enough to avoid high reaction barriers. If the number of reactant candidates was insufficient, the donor–acceptor distance threshold was gradually increased from 3 Å until at least 2000 reactant candidates were identified. This adjustment was particularly necessary for reactions such as H migration from C6 to C1, where very few structures satisfied the initial 3 Å distance criterion.

From each set of reactant candidates, the first 2000 lowest-energy conformers were selected for geometrically constrained optimization to generate initial guesses for the TSs. During the constrained optimization, harmonic restraint forces were applied selectively to certain atomic distances to approximate the transition-state geometry, while the remaining atomic coordinates were allowed to relax to their optimal configurations. The choice of atoms to constrain was based on the type of reaction, and the specific distances for the constraints were determined from the geometries of TSs reported in previous studies of similar reactions.^40^ Constrained optimizations were performed by using DFTB, with a harmonic force constant of 150 kJ/mol·Å^2^.

The resulting predicted TS structures were then screened to identify distinct conformers. Each screened structure was evaluated to ensure it resembled the expected TS geometry before proceeding to further TS optimization using a higher-level DFT method at the M06-2X/6-31+(d,p) level of theory. If fewer than 100 guessed TS structures were obtained, the constrained optimization procedure was repeated with adjusted parameters to generate additional guessed TS structures. For each reaction, the 100–200 lowest-energy TS structures were then selected for DFT optimization. The TS structures were confirmed via intrinsic reaction coordinate (IRC) calculations. The corresponding reactant states for the confirmed TSs were obtained from IRC calculations and subsequently optimized to achieve fully relaxed geometries. If the number of DFT-optimized TS structures was less than three, the constrained optimization procedure was again repeated with modified parameters to generate additional TS guesses for subsequent DFT calculations.

All metadynamics simulations and the geometrically constrained structure optimization calculations were carried out using CP2K software (version 8.1),^41^ and the DFT calculations utilized Gaussian 16 ^42^ The version of DFTB utilized in these calculations was the GFN-xTB model.^43^ Figure 3a shows the procedure of our calculations and the representative structures of glucose and the disaccharides used in this study (Figure 3b).

**Figure 3:**
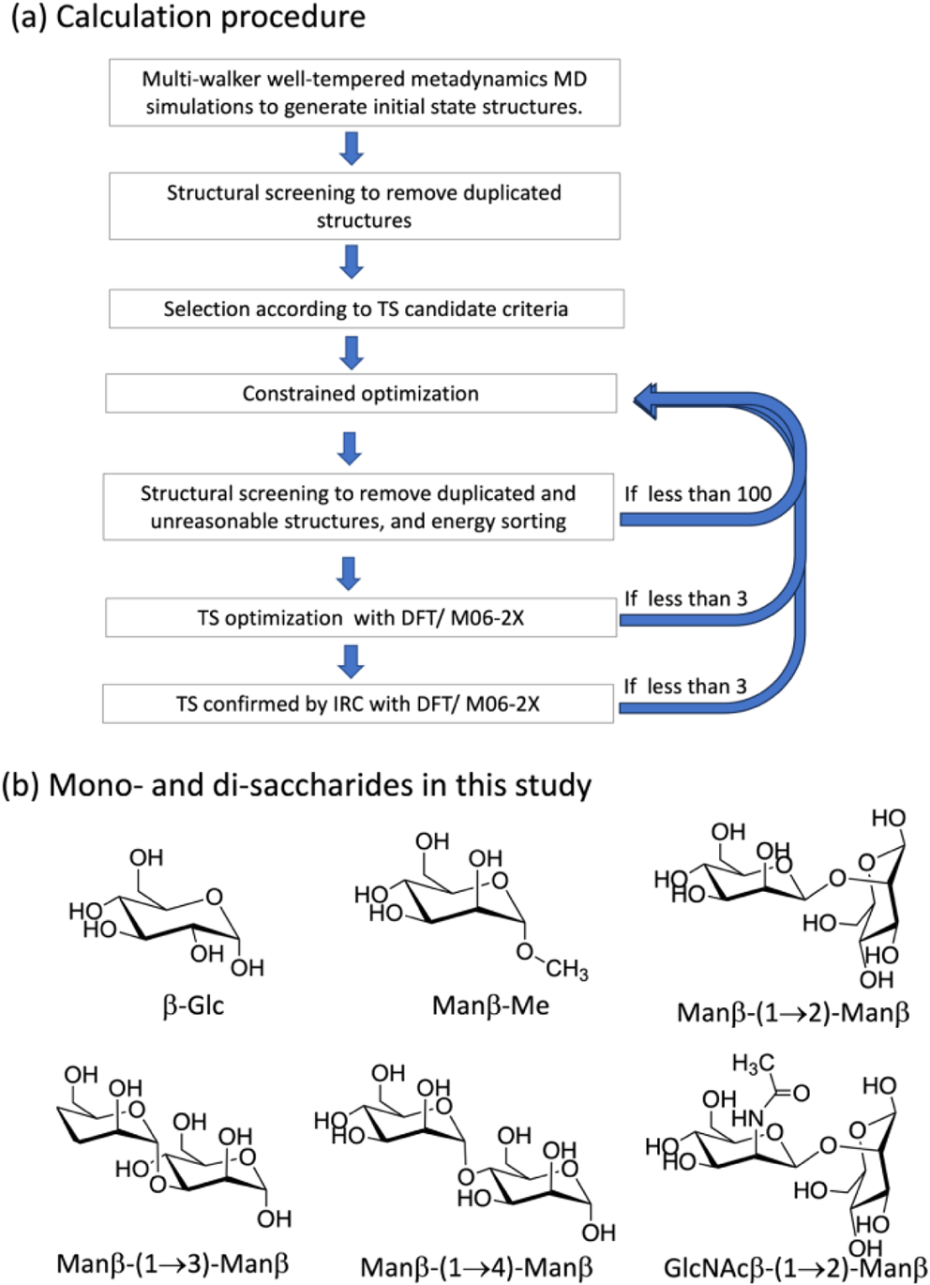
(a) Calculation procedure, in which, in the last three steps, if the number of structures is less than the specified limit, the constrained optimization is repeated with modified restraints to increase the number of TSs. (b) Representative structures of β-glucose, methyl β-mannose, and mannose disaccharides used in this study.

## Results and Discussion

In the following paragraphs, all the ions of monosaccharides and disaccharides represent the singly charged monosaccharide and disaccharide sodium ion adducts unless otherwise stated.

### (A) β-Glc and β-Man-Me

The calculations of disaccharide cross-ring dissociation require a huge amount of computational time. To save computational time, we first calculated the reactions of monosaccharides and identified the reactions with low barriers. Then, analogous reactions of the disaccharides were investigated.

In our previous study, we found that the barrier of the ring-opening reaction through O5–C1 bond cleavage (denoted as reaction (0) in Figure 4a) of β-glucose is typically less than 200 kJ/mol. It is much lower than that of alternative ring-opening reactions, which involve cleavage of the C–C bonds of the sugar ring via H-atom transfer from OH groups to a nearest neighbor C-atom. The barriers of those alternative ring-opening reactions typically exceed 310 kJ/mol.^27,29^ Meanwhile, the barriers of 1,2 and 1,3 hydrogen shifts (denoted as reactions (1) and (2), respectively, in Figure 4a) following the O5–C1 bond cleavage, which lead to the major cross-ring dissociation (Figure 1a), are very low (165 and 163 kJ/mol) compared to the ring-opening reaction. To account for the minor cross-ring fragments observed in CID spectra that cannot be fully explained by those low-energy reactions, we explored other possible dissociation pathways involving high-barrier reactions. We first considered alternative hydrogen shifts (i.e., other than 1,2 and 1,3 hydrogen shifts) after the O5–C1 ring-opening reaction. The cleavage of the other C–C bonds, which leads to cross-ring dissociation different from the major cross-ring dissociation, requires the generation of a C=O functional group at other C atoms without passing through the C2 atom. These changes can be achieved by transferring the H atom from (O4, C4), (O5, C5), or (O6, C6) to (O1), as illustrated by reactions (3), (4), and (5) in Figure 4a. Our calculations show that the lowest barriers of reactions (3), (4), and (5) are 203, 195, and 201 kJ/mol, respectively (Figure 4d). The corresponding transition-state geometries are shown in Figure S3 of Supporting Information. The relatively low barriers of these hydrogen shifts, compared to the alternative ring-opening reactions which typically exceed 310 kJ/mol,^27,29^ indicate that these reactions may occur in linear hexose (i.e., after ring-opening) and provide a prerequisite for us to study these reactions in disaccharides with different linkages.

**Figure 4:**
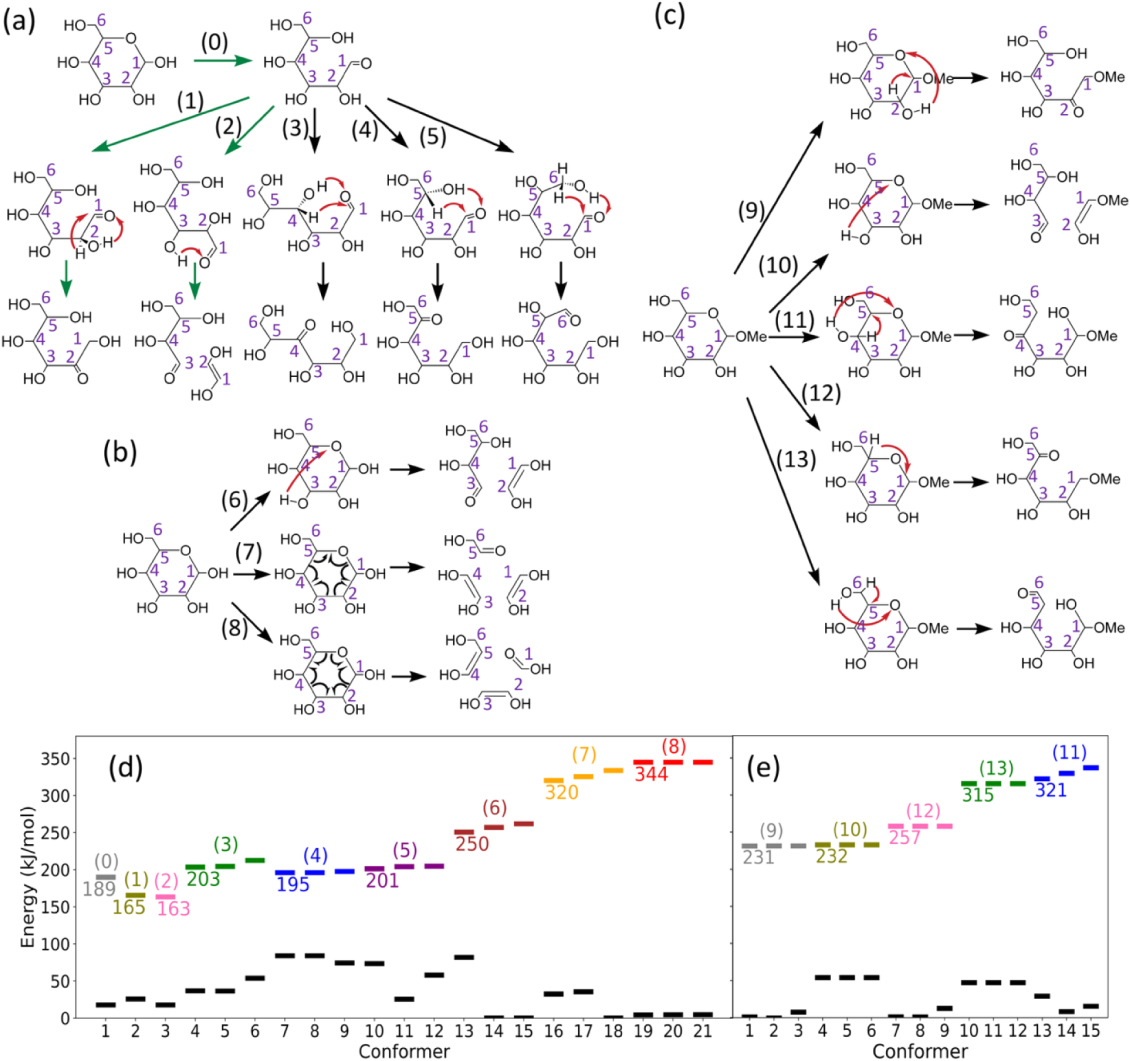
(a) Hydrogen shifts of a singly charged β-glucose sodium ion adduct after the ring-opening reaction through O5–C1 bond cleavage, applicable to hexose at the reducing end. Green arrows represent the pathways studied previously; black arrows represent the pathways studied in this work. The position of the sodium ion is not shown. (b) Cross-ring reactions of singly charged a β-glucose sodium ion adduct without going through the ring-opening reaction via O5–C1 bond cleavage, applicable to both hexoses at the reducing end and those not at the reducing end. (c) Hydrogen shift and cross-ring reactions of a singly charged methyl β-mannose sodium ion adduct without going through the ring-opening reaction via O5–C1 bond cleavage, applicable to hexoses not at the reducing end. (d) and (e) Zero-point corrected energies of the first three lowest TSs and their corresponding reactants for the reactions in (a)–(c), calculated using the DFT/M06–2X method. The full results are presented in Figures S1 and S2 of Supporting Information. The global energy minima of singly charged β-glucose and Manβ-Me sodium ion adducts are used as energy references, respectively. Black dashes represent reactant states, and the dashes above reactant states represent the corresponding TSs. Different colors of TSs represent different reactions, indicated by the symbols above the dashes.

Next, we investigated the reactions that lead to cross-ring dissociation without starting from the ring-opening reaction. Three reactions were found, as illustrated by reactions (6), (7), and (8) in Figure 4b. Reaction (6) involves H-atom transfer from O3 to O5, leading to direct cross-ring dissociation. Reactions (7) and (8) involve simultaneous cleavage of three C–C bonds without H-atom transfer; analogous reactions have been proposed to explain the cross-ring dissociation of permethylated hexoses.^44^ In this study, we show that the barriers for cross-ring reactions without starting from ring-opening are significantly higher than those of reactions (1)–(5) (Figure 4c) such that they cannot compete with those reactions under CID.

We also examined the ring-opening reactions for hexoses not located at the reducing end. To mimic the hexose not at the reducing end, the hydrogen atom connected to the O1 of a mannose was replaced by a methyl group. Mannose was chosen because it is more closely related to mannose disaccharides, which are the main focus of this work. Two types of reactions were considered for the cross-ring dissociation of the hexose not at the reducing end. The first type is the direct cross-ring dissociation without an H-atom shift and ring-opening reaction. These reactions are analogous to reactions (7) and (8). Because the barriers of reactions (7) and (8) are very high (more than 300 kJ/mol) compared to the other ring-opening reactions, they suggest that the barriers of the analogous reactions for the hexose not at the reducing end are high. Thus, this type of reaction is not further investigated. The second type of reaction involves the generation of a C=O functional group in the hexose, which can further undergo a retro-aldol reaction easily and eventually lead to C–C bond cleavage. Reactions (9), (10), (11), (12), and (13) involve H-atom shifts from O atoms or C atoms at positions 2, 3, 4, 5, and 6 toward O5 and generate a C=O functional group at the corresponding positions (Figure 4c). Except for reaction (10), which leads directly to a cross-ring reaction, all the other reactions lead to ring-opening. The barriers of reactions (9), (10), and (12) are relatively low (231, 232, and 257 kJ/mol, Figure 4e) compared to those of reactions (11) and (13) (more than 300 kJ/mol). However, all of them are much higher than those of hexose at the reducing end. We will consider reactions (9), (10), and (12) to evaluate the cross-ring dissociation for the hexose not at the reducing end in subsequent disaccharide calculations.

### (B) Manβ-(1→2)-Manβ

The CID spectrum of a singly charged Manβ-(1→2)-Manβ sodium ion adduct, in which the O1 atom of the reducing-end mannose is replaced by ^18^O, is shown in Figure 5a. The major cross-ring fragment at *m*/*z* 247 corresponds to the loss of a neutral species with m = 120. Figure 5b shows an enlarged spectrum to highlight the minor cross-ring fragments at *m*/*z* 245, 275, 277, 305, 307, and 337, corresponding to neutral losses of m = 122, 92, 90, 62, 60, and 30, respectively. The major fragment at *m*/*z* 247 can be explained by the mechanisms indicated with green arrows in Figure 5c, which are analogous to those described in Figure 1b. The pathway begins with a ring-opening reaction of the reducing-end mannose (denoted as RO), followed by a retro-aldol reaction via hydrogen transfer from O1 to O3, resulting in cleavage of the C2–C3 bond (denoted as c2 in Figure 5c, with the numbering referring to the numbering of the C–C or C–O bonds in the ring). This reaction generates the major cross-ring fragment at *m*/*z* 247. Our quantum-chemistry calculations indicate that the transition-state energy for this reaction is relatively low (152 kJ/mol, Figure 5e) compared to the other ring-opening reactions. While these mechanisms account for the formation of the major cross-ring fragment at *m*/*z* 247, they cannot explain the observed minor cross-ring fragments at *m*/*z* 245, 275, 277, 305, 307, and 337.

**Figure 5:**
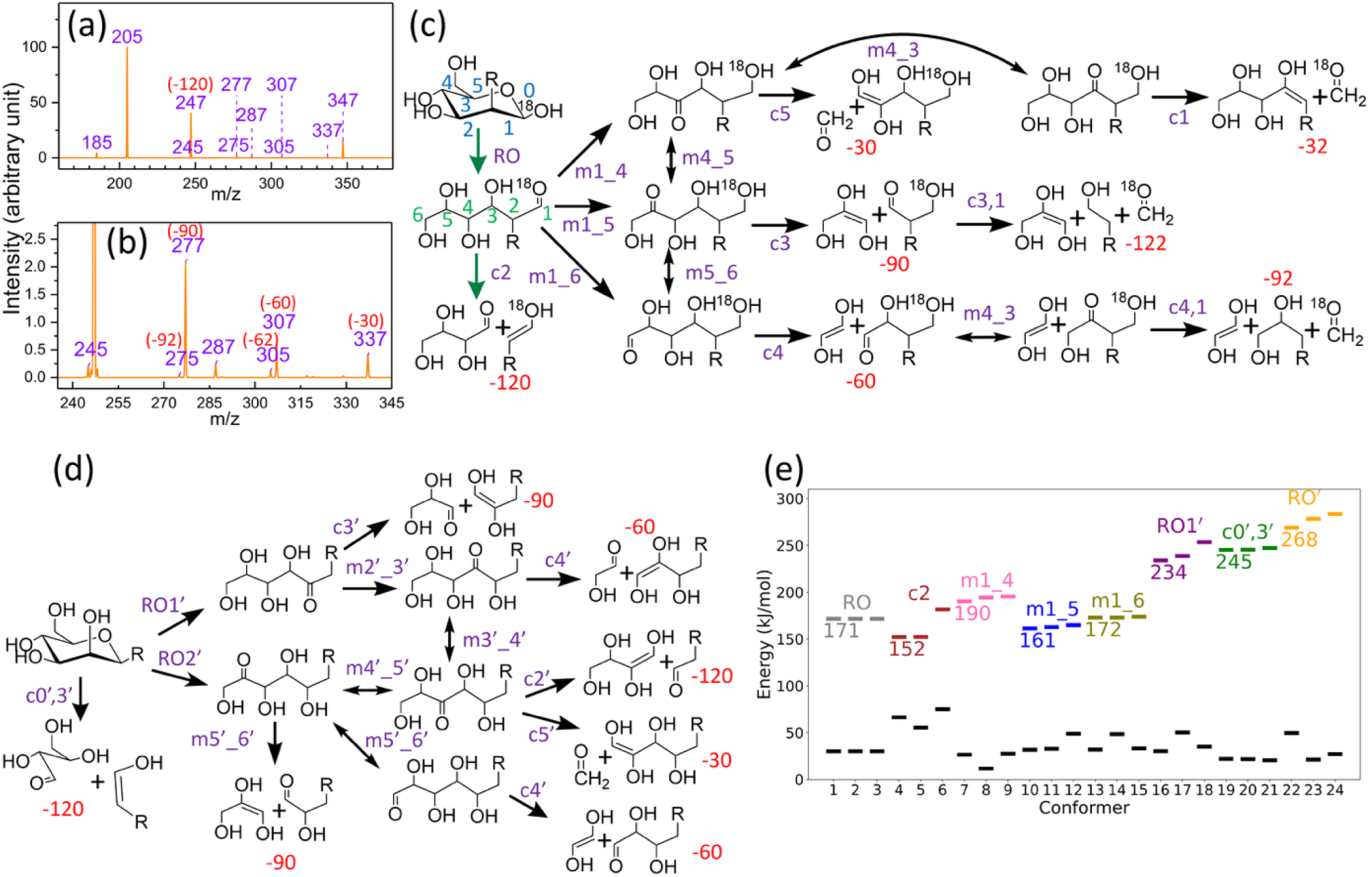
(a) CID spectrum of the singly charged Manβ-(1→2)-Manβ sodium adduct. The O1 atom at the reducing end is replaced by ^18^O. (b) Enlarged CID spectrum showing the minor cross-ring fragments. (c) Reaction mechanisms of cross-ring dissociations for the singly charged sodium ion adduct of hexose at the reducing end. Reactions indicated by green arrows have been discussed in our previous studies,^27,29–33^ while reactions indicated by black arrows are newly studied in this work. Position of the sodium ion is not shown. (d) Reaction mechanisms of cross-ring dissociations for the singly charged sodium ion adduct of hexose not at the reducing end. Position of sodium ion is not shown. (e) Zero-point corrected energies of the first three lowest TSs for each type of reaction and the corresponding reactants, calculated using the DFT/M06–2X method. The full results are in Figure S4a of Supporting Information. The global minimum of the singly charged sodium ion adduct of Manβ-(1→2)-Manβ is used as the energy reference. Black dashes represent reactant states, and the dashes above reactant states represent the corresponding TSs. Different colors of TSs represent different reactions, indicated by the symbols above the dashes.

To explain the minor cross-ring fragment formation, alternative pathways indicated by black arrows in Figure 5c were investigated, where the key initial reactions are hydrogen-shift reactions of m1_4, m1_5, and m1_6, analogous to reactions (3), (4), and (5) in monosaccharides. These reactions cause the migration of the carbonyl (C=O) functional group to other carbon atoms, which further facilitates cleavage of C–C bonds other than the major pathway. Migration of hydrogen from O4 to O1 changes the C=O group from C1 to C4 (denoted as m1_4). The subsequent retro-aldol reaction cleaves the C5–C6 bond (denoted as c5), generating a fragment at *m*/*z* 337, corresponding to the loss of a neutral species with m = 30. Following the m1_4 reaction, the subsequent m4_3 and c1 reactions produce fragments at *m*/*z* 335. Similarly, hydrogen migration from O5 to O1 and from O6 to O1 changes the C=O group from C1 to C5 (denoted as m1_5) and from C1 to C6 (denoted as m1_6), respectively. The retro-aldol reactions following these C=O functional group position changes generate fragments at *m*/*z* 277 (loss of neutral species with m = 90) and *m*/*z* 307 (loss of neutral species with m = 60). Subsequent secondary dissociation of these fragments leads to the formation of fragments at *m*/*z* 245 (loss of total neutrals of 122 Da) and *m*/*z* 275 (loss of total neutrals of 92 Da), respectively. Quantum-chemical calculations indicate that the transition-state energies for these hydrogen-shift-driven C–C bond cleavages (161, 172, and 190 kJ/mol) are higher than those of the c2 reaction, as illustrated in Figure 5e. The geometries of these lowest transition states are shown in Figure S4b of Supporting Information.

To understand how accessible the reactant states are for these four main reactions (c2, m1_4, m1_5, and m1_6), we analyze the distinct reactant structures generated by metadynamic MD simulations of Manβ-(1→2)-Manβ with the reducing-end mannose in linear form. We calculated the number of reaction candidates for each reaction, as discussed in the methods section, such that the O–O distance between the H donor and acceptor is less than 3 Å. For the c2 reaction, the geometries primarily require restrictions on the relative positions of three carbon atoms, C1, C2, and C3, such that the distance between the O atoms of the H donor and acceptor is sufficiently short for a low-barrier reaction to occur. For the m1_4, m1_5, and m1_6 reactions, low-lying TSs require that a larger number of carbon atoms be restricted, which are 4, 5, and 6, respectively. Fewer geometric restrictions result in a larger number of accessible reactant states, and a greater number of reactant states implies that more molecules can undergo the reaction. The results, illustrated in Figure S4c of Supporting Information, show that the c2 reaction has the largest number, followed by m1_4, m1_5, and m1_6. With its low reaction barrier and highly accessible reactant states, the c2 reaction is the most favorable cross-ring dissociation pathway. In contrast, the other C–C bond cleavages, whose reactant states are less accessible and whose reaction barriers are higher, account for the formation of the minor cross-ring fragments. These findings are consistent with the CID spectra shown in Figure 5a and b.

The dissociation mechanisms described above occur only for the mannose at the reducing end because the initial step—ring-opening via hydrogen transfer from O1 to O5—can take place only at the reducing end. In this study, we also considered the possibility that minor cross-ring fragments might originate from the mannose and not at the reducing end. Based on calculations for the monosaccharide Man–OMe, cross-ring dissociation of hexose not at the reducing end mainly proceeds via three pathways. These are the c0′,3′ reaction, the RO1′ reaction, and the RO2′ reaction, which are analogous to reactions (6) and (10), reaction (9), and reaction (12) in Figure 4b, respectively. The c0′,3′ reaction directly generates a fragment at *m*/*z* 247, corresponding to the loss of a neutral species with m = 120, while the RO1′ and RO2′ reactions lead to the production of a fragment at *m*/*z* 277, corresponding to the loss of neutral species with m = 90. Subsequent hydrogen migrations—m2′_3′, m4′_5′, or m5′_6′—following the RO1′ and RO2′ reactions lead to additional fragments at *m*/*z* 337, 307, and 247, as illustrated in Figure 5d. Quantum chemistry calculations show that the transition-state energies for these three reactions are very high (Figure 5e) compared to those of the ring-opening reaction at the reducing end, indicating that these pathways are unlikely to occur. Consequently, any corresponding fragments in the CID spectra originating from hexose not at the reducing end are expected to have very low intensities.

### (C) Manβ-(1→3)-Manβ

The CID spectrum of the singly charged Manβ-(1→3)-Manβ sodium ion adduct, as illustrated in Figure 6a, shows that the major cross-ring fragment occurs at *m*/*z* 277. This fragment can be explained by the reaction pathway RO→m1_2→c3, indicated by the green arrows in Figure 6c. The minor fragments at *m*/*z* 305, 307, 337, and 245, as illustrated in Figure 6b, can be rationalized by the pathways indicated with black arrows in Figure 6c, which involve alternative hydrogen shifts. These reactions are analogous to those for the reducing-end hexose described in Figure 4a and provide plausible explanations for the formation of many previously unexplained minor cross-ring fragments observed in Figure 6b.

**Figure 6:**
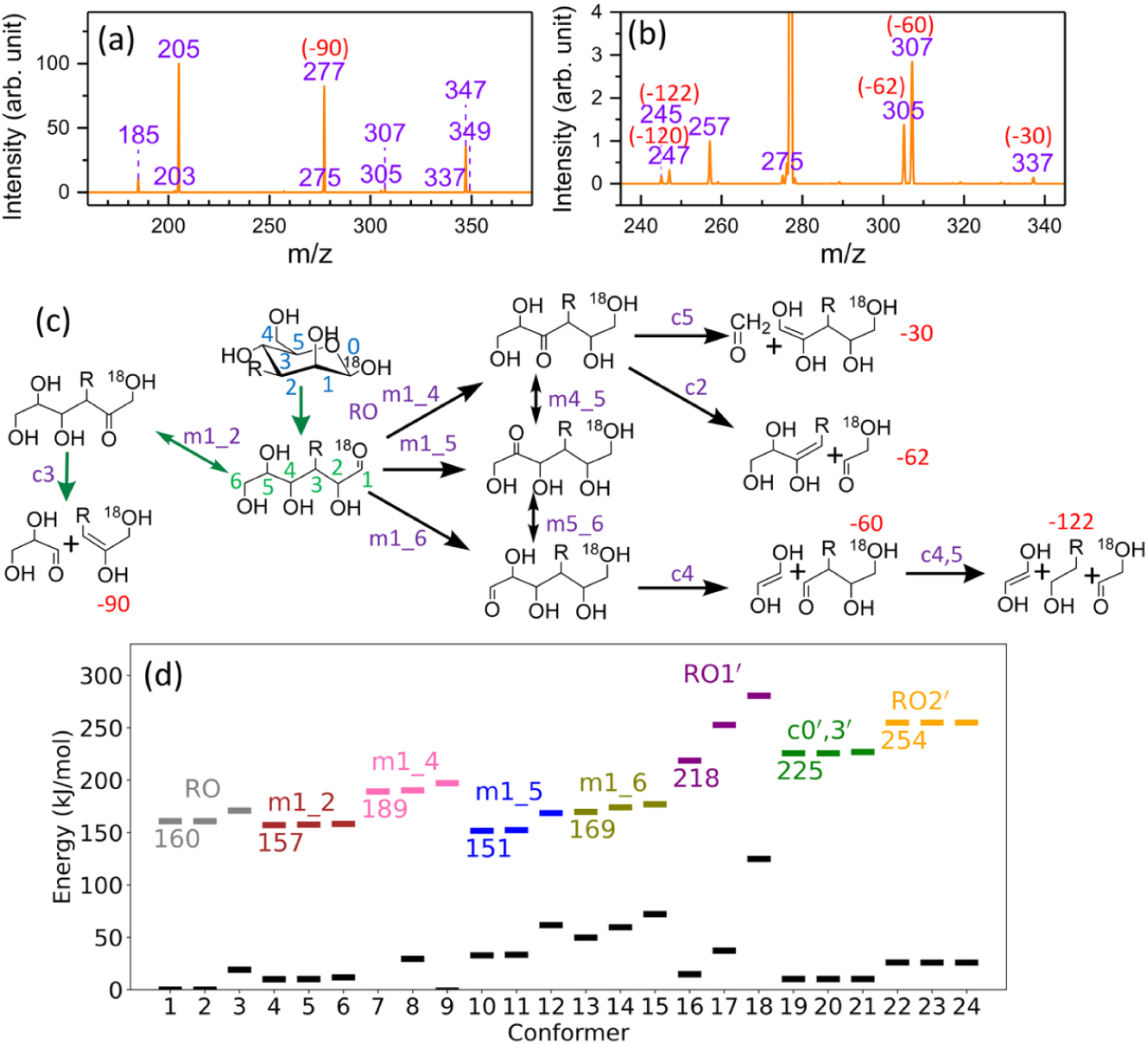
(a) CID spectrum of the singly charged Manβ-(1→3)-Manβ sodium adduct. The O1 atom at the reducing end is replaced by ^18^O. (b) Enlarged CID spectrum showing the minor cross-ring fragments. (c) Reaction mechanisms of cross-ring dissociations for the singly charged sodium ion adduct of hexose at the reducing end. Reactions indicated by green arrows have been discussed in our previous studies,^27,29–33^ while reactions indicated by black arrows are newly studied in this work. Position of sodium ion is not shown. (d) Zero-point corrected energies of the first three lowest TSs for each type of reaction and the corresponding reactants, calculated using the DFT/M06–2X method. The full results are presented in Supporting Information Figure S5a. The global minimum structure of the singly charged Manβ-(1→3)-Manβ sodium ion adduct is used as the energy reference. Black dashes represent reactant states, and dashes with different colors represent TSs of different reactions.

The three lowest TS energies for the reactions shown in Figure 6c are presented in Figure 6d. The ratio of distinct reactant states for these reactions, calculated from the metadynamic MD simulation, and the corresponding TS configurations are illustrated in Figure S5b and S5c of the Supporting Information, respectively. Among these shifts, m1_5 has the lowest TS energy, followed by m1_2, m1_6, and m1_4. Although the m1_5 reaction has a barrier slightly lower than that of m1_2, it requires more sequential steps to produce cross-ring fragmentation, which explains why m1_2 dominates the formation of the *m*/*z* 277 fragment. Furthermore, the number of accessible reactant states for m1_4, m1_5, and m1_6 is significantly smaller than that for m1_2, further contributing to the higher intensity of *m*/*z* 277 from reaction m1_2.

The fragment at *m*/*z* 247 cannot be explained by the cross-ring dissociation of the reducing-end mannose. It is likely produced through cross-ring dissociation not at the reducing end via pathways such as c0′,3, or RO1′→m2′_3′→m3′_4′→c2′, or RO2′→m5′_4′→c2′, as illustrated by the mechanism shown in Figure 5d. The barriers of the first step in each reaction are 225 kJ/mol (c0′,3′), 218 kJ/mol (RO1′), and 254 kJ/mol (RO2′), respectively. Considering both the energies and the number of required reaction steps, the c0′,3′ pathway likely contributes dominantly to the *m*/*z* 247 fragment intensity.

### (D) Manβ-(1→4)-Manβ

Figure 7a and b show the CID spectrum of the singly charged Manβ-(1→4)-Manβ sodium ion adduct. The scheme in Figure 7c illustrates the various cross-ring reactions occurring for the reducing-end mannose with a 1→4 linkage. The cross-ring fragments at *m*/*z* 305, 307, and 335, corresponding to neutral losses of m = 62, 60, and 32, are generated through the reaction sequences RO→c2, RO→m1_2→m2_3→c4, and RO→m1_2→m2_3→c1, respectively. These dissociation pathways, indicated by green arrows in Figure 7c, are analogous to the mechanisms described in Figure 1b. The fragment at *m*/*z* 245 can be attributed to secondary dissociation of the fragment at *m*/*z* 305; i.e., after formation of the fragment at *m*/*z* 305, the residual internal energy allows further dissociation by eliminating neutral species with m = 60, resulting in the fragment at *m*/*z* 245 (loss of total neutral mass of 122 Da), i.e., RO→c2→c2,4. Minor cross-ring fragments at *m*/*z* 275 (loss of neutral species with m = 92) can be produced via the sequence RO→m1_5→c3, as illustrated by the black arrows in Figure 7c.

**Figure 7:**
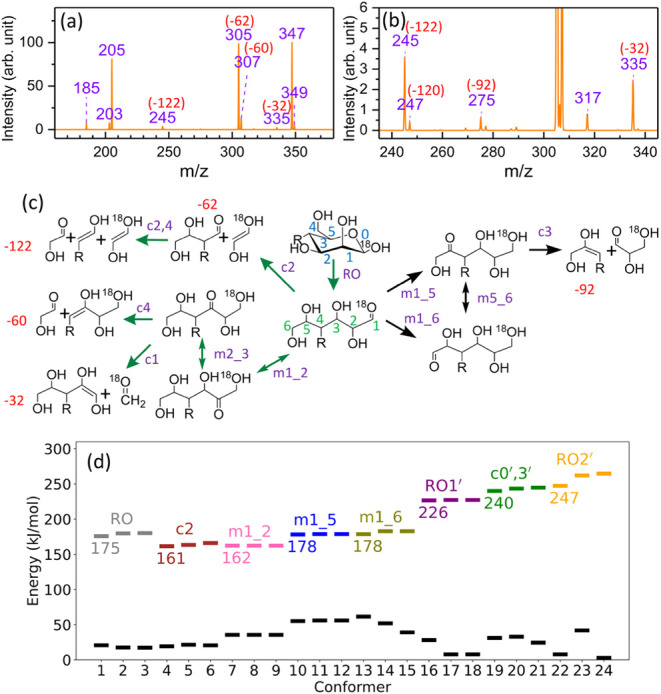
(a) CID spectrum of a singly charged Manβ-(1→4)-Manβ sodium adduct. The O1 atom at the reducing end is replaced by ^18^O. (b) Enlarged CID spectrum showing the minor cross-ring fragments. (c) Reaction mechanisms of cross-ring dissociations for the singly charged sodium ion adduct of hexose at the reducing end. Reactions indicated by green arrows have been discussed in our previous studies,^27,29–33^ while reactions indicated by black arrows are newly studied in this work. Position of sodium ion is not shown. (d) Zero-point corrected energies of the first three lowest TSs for each type of reaction and the corresponding reactants, calculated using the DFT/M06–2X method. The full results are presented in Supporting Information Figure S6a. The global minimum structure of the singly charged Manβ-(1→4)-Manβ sodium ion adduct is used as the energy reference. Black dashes represent reactant states, and dashes with different colors represent TSs of different reactions.

In contrast, other minor fragments, such as *m*/*z* 247 (loss of neutral species with m = 120) and *m*/*z* 277 (loss of neutral species with m = 90), whose intensities are much lower, arise from cross-ring dissociation of the mannose not at the reducing end, as they cannot be explained by the reducing-end reaction scheme in Figure 7c.

The calculated TS energies for the key reactions are listed in Figure 7d. The relative accessibility of reactant states for the four reactions occurring at the reducing end, right after the RO reaction, and the corresponding TS configurations are illustrated in Figure S6b and S6c, respectively. Among these reactions, the c2 reaction has the lowest TS energy. Although the m1_2 reaction also has a relatively low TS energy, it requires two additional isomerization steps before C–C bond cleavage can occur. Moreover, the reactant states of the m1_2 are found to be less accessible compared to those of the c2. These factors explain the differences in peak intensities between the major cross-ring fragments: *m*/*z* = 305 (from the c2 reaction) versus *m*/*z* = 307 and 335 (from the m1_2 reaction).

The TS energies for the m1_5 and m1_6 reactions are similar (178 kJ/mol), significantly higher than those of the c2 and m1_2 reactions, which accounts for the much lower intensity of fragments at *m*/*z* 275 (from the m1_5 or m1_6 reactions) compared to those at *m*/*z* 305, 307, and 335 from the c2 and m1_2 reactions. Additionally, the smaller number of accessible reactant states for the m1_5 and m1_6 reactions further contributes to the lower fragment intensities. Figure 7d also shows the TS energies of the reactions RO1′ (226 kJ/mol), c0′,3′ (240 kJ/mol), and RO2′ (247 kJ/mol), which are the major reactions occurring for the mannose not at the reducing end. These energies are considerably higher than those of the reactions at the reducing end, consistent with the CID spectra, where the peak intensities of *m*/*z* 247, 277, and 337 (not from reducing-end reactions) are much lower than those of *m*/*z* 305, 307, and 335 (from reducing-end reactions).

### (E) Application to *N*-Glycans

Here, we demonstrate that the newly proposed cross-ring dissociation mechanisms can aid in the structural characterization of *N*-glycans. *N*-glycosylation is one of the most important post-translational modifications of proteins; however, the structural determination of *N*-glycans is challenging. The *N*-glycan used in this study is GlcNAcβ-(1→2)-Manα-(1→6)-[Manα-(1→3)]-GlcNAcβ-(1→4)-GlcNAc, whose structure is illustrated at the top of Figure 8a. Structural determination of *N*-glycans by mass spectrometry has been reported in our previous work.^45^ Here, we show how the new cross-ring dissociation mechanisms can clarify the minor cross-ring fragments in CID spectra, thereby helping to determine the linkage position of the GlcNAcβ-(1→2)-Manα moiety in this *N*-glycan. To determine the linkage of GlcNAcβ-(1→2)-Manα, the CID sequence 1136→712→406→ fragments (upper part of Figure 8a) was used to generate the disaccharide GlcNAcβ-(1→2)-Manα. The structure of this disaccharide was then analyzed by subsequent CID spectroscopy (lower part of Figure 8a) to determine its linkage. According to the dissociation mechanisms presented in Figure 1b, the major cross-ring dissociation for a disaccharide with a 1→2-linked aldo-hexose at the reducing end corresponds to the loss of neutral species with m = 120, producing the fragment at *m*/*z* 286. Indeed, this fragment has the highest intensity in the spectrum compared to other cross-ring fragments at *m*/*z* 346 (loss of neutral species with m = 60) and *m*/*z* 316 (loss of neutral species with m = 90). Notably, the intensity ratios of the minor cross-ring fragments *m*/*z* 316 and 346 relative to the major fragment *m*/*z* 286 are significantly higher than those observed for the Man-(1→2)-Man disaccharide shown in Figure 5a.

**Figure 8:**
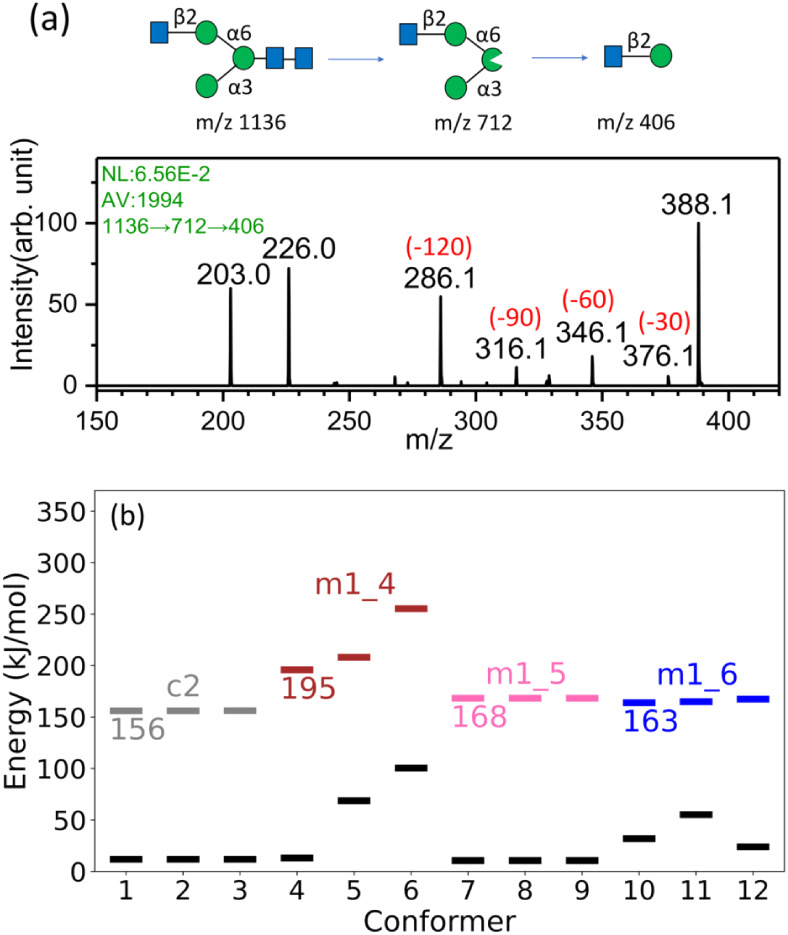
(a) Top: CID sequence of a singly charged *N*-glycan sodium ion adduct and the corresponding fragment structures. Bottom: CID spectra. (b) Zero-point corrected energies of the first three lowest TSs for each type of reaction and the corresponding reactants calculated using the DFT/M06–2X method. The full results are presented in Supporting Information Figure S7a. The global minimum structure of the singly charged β-GlcNAc-(1→2)-Man sodium ion adduct (reducing end mannose is in linear form) is used as the energy reference. Black dashes represent reactant states, and dashes with different colors represent TSs of different reactions.

If the CID spectrum were interpreted solely based on the cross-ring dissociation mechanisms in Figure 1b, the fragments at *m*/*z* 316 and 346 in the CID spectrum of Figure 8a would be considered as the *N*-glycans with other linkages mixed into the sample. Using the newly proposed cross-ring dissociation mechanisms shown in Figure 5b, these two fragments can be rationalized as resulting from cross-ring dissociation occurring at the mannose of GlcNAcβ-(1→2)-Manα. However, the question remains: why are the intensity ratios of the ions corresponding to neutral losses of m = 90 and 60 relative to the fragment from the neutral loss of m = 120 higher than in the Manβ-(1→2)-Man disaccharide? To address this, we performed quantum-chemical calculations on GlcNAcβ-(1→2)-Manα. Based on the Manβ-(1→2)-Manβ study described in the aforementioned sections, all major and minor cross-ring fragments are generated after ring-opening of the reducing-end monosaccharide. Therefore, in the calculations for GlcNAcβ-(1→2)-Manα, we bypassed the ring-opening step and directly calculated the transition states of the retro-aldol reactions and hydrogen shifts that occur after ring-opening, i.e., treating mannose in linear form (denoted as GlcNAcβ-(1→2)-Man).

The calculated TS energies for cross-ring dissociation and hydrogen shifts are shown in Figure 8b, with the corresponding TS geometries illustrated in Figure 8d. The results are similar to those for Manβ-(1→2)-Manβ: the c2 reaction has the lowest TS energy (156 kJ/mol, yielding fragment *m*/*z* 286), while the m1_5 (168 kJ/mol, leading to fragment *m*/*z* 316) and m1_6 (163 kJ/mol, leading to fragment *m*/*z* 346) reactions have higher TS energies. However, the differences in TS energies between c2, m1_5, and m1_6 are smaller than those in Manβ-(1→2)-Manβ. The relative numbers of reactant states for these four reactions, as illustrated in Figure S7 of Supporting Information, show significant differences from those of Manβ-(1→2)-Manβ. This indicates that, for GlcNAcβ-(1→2)-Man, the c2 and m1_4 reactions are less accessible relative to m1_5 and m1_6. The combination of smaller TS energy differences and fewer accessible conformers for the c2 reaction explains the higher intensity ratios of fragments *m*/*z* 316 and 346 relative to *m*/*z* 286 in GlcNAcβ-(1→2)-Man.

## Conclusions

In this study, we investigated the CID cross-ring dissociation mechanisms of singly charged sodium ion adducts of β-glucose, methyl-β-mannose, and mannose disaccharides (Manβ-(1→2)-Manβ, Manβ-(1→3)-Manβ, and Manβ-(1→4)-Manβ) using high-level quantum chemical calculations to obtain the transition state energy of each reaction, as well as metadynamic MD simulations to obtain the accessible reactant states. The calculated results were used to explain the experimental measurements of CID mass spectrometry. Consistent with our previous work, we confirm that the cross-ring dissociation of hexose sodium ion adducts follows a stepwise process. This process begins with ring opening via hydrogen transfer from O1 to O5, accompanied by cleavage of the O5–C1 bond. After ring opening, the system either proceeds directly through a retro-aldol reaction or undergoes a 1,2-hydrogen shift that repositions the carbonyl group prior to the retro-aldol step. These pathways successfully explain the major cross-ring fragments commonly observed in CID spectra and provide a basis for distinguishing oligosaccharides with different glycosidic linkages.

However, these previously established mechanisms do not account for the presence of minor cross-ring fragments. To address this limitation, we propose new dissociation pathways that share the same initial ring-opening step but differ in the subsequent hydrogen-transfer processes. Instead of the conventional 1,2-hydrogen shift, the newly identified pathways involve alternative rearrangements, including 1,4-, 1,5-, and 1,6-hydrogen shifts. The relatively low energy barrier of the 1,2-hydrogen shift explains the dominance of major fragments, whereas the higher energy barriers associated with these alternative hydrogen shifts rationalize the lower abundance of minor fragments observed experimentally.

Our results further show that both the previously reported and newly proposed mechanisms are localized at the reducing end of the hexose. In contrast, cross-ring dissociation at nonreducing residues is energetically unfavorable due to significantly higher transition-state barriers, indicating that such contributions to CID fragmentation are minimal. This site specificity of cross-ring dissociation provides additional insight into the structural origin of the observed fragment ions.

To demonstrate the broader applicability of these findings, we applied the proposed mechanisms to a representative *N*-glycan, GlcNAcβ-(1→2)-Manα-(1→6)-[Manα-(1→3)]-GlcNAcβ-(1→4)-GlcNAc. In this system, the energy differences between transition states leading to major and minor cross-ring fragments are smaller than those observed in mannose disaccharides. As a result, minor fragments appear with relatively higher abundance. Importantly, this observation supports the conclusion that these minor fragments arise from intrinsic dissociation pathways rather than from sample heterogeneity or mixtures of different glycan linkages.

Overall, this work expands the mechanistic understanding of cross-ring fragmentation in the CID of sodium-adducted carbohydrates by identifying additional hydrogen-shift pathways responsible for minor fragment formation. These insights improve the interpretation of CID spectra and enhance the reliability of structural assignments in glycan analysis. It should be noted, however, that the proposed mechanisms are specific to singly charged sodium ion adducts under CID conditions. They are not applicable to other ion types (e.g., protonated or deprotonated species) or alternative fragmentation techniques, such as higher-energy collisional dissociation (HCD), electron transfer dissociation (ETD), or electron capture/attachment dissociation (ECD/EAD), where fundamentally different mechanisms may govern fragmentation behavior.

## Supplementary Material



## Notes

Full results of zero-point corrected energies of the TSs for each type of reaction and the corresponding reactants; XYZ coordinates of TSs and reactants; geometries of TS; and the numbers of accessible reactant states calculated by metadynamic MD simulations (PDF)
