# Supplementary material for: Unraveling Cross-Ring Dissociation Mechanisms of Hexoses in Collision-Induced Dissociation
Source: J Phys Chem A. 2026 Apr 20;130(17):3430–41. doi: 10.1021/acs.jpca.6c00953 (PMC13137245; doi:10.1021/acs.jpca.6c00953)
Supplement: Supplementary file 1 [file jp6c00953_si_001.pdf]

Supporting Information

Unraveling Cross-Ring Dissociation Mechanisms of Hexoses in Collision-Induced Dissociation

Hock-Seng Nguan[1], Yen-Ting Lin[1, 2, 3], and Chi-Kung Ni[*1, 2,3]

1. Institute of Atomic and Molecular Sciences, Academia Sinica, P. O. Box 23-166, Taipei 10617, Taiwan.

2. Department of Chemistry, National Tsing Hua University, Hsinchu 30013, Taiwan

* Corresponding authors: E-mail: nguanhs@gmail.com, ckni@po.iams.sinica.edu.tw

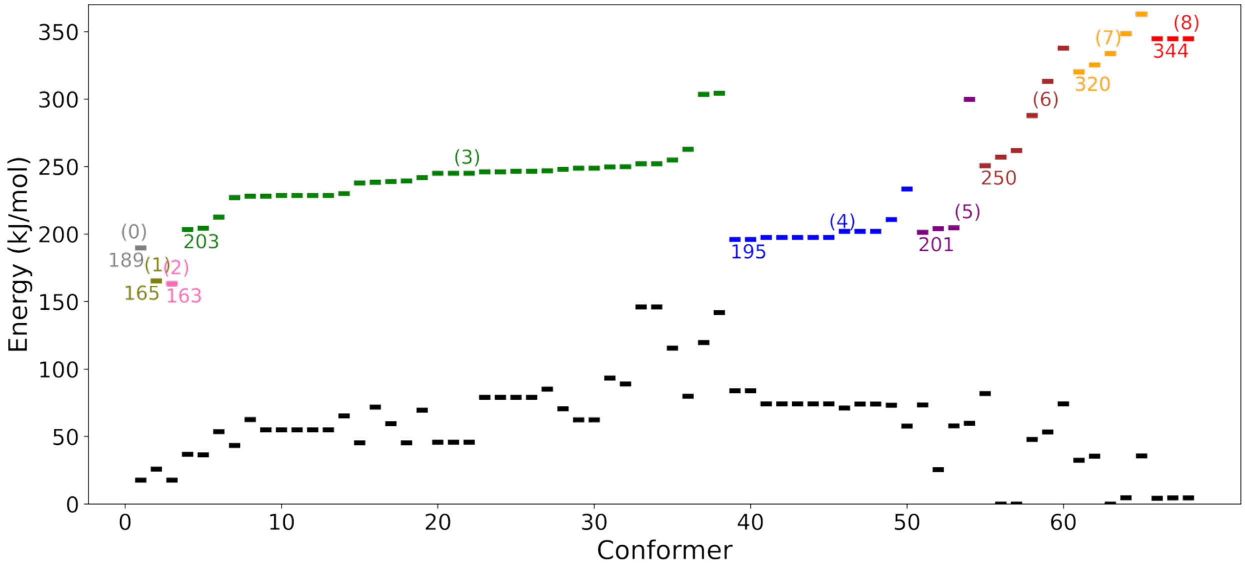

Figure S1. Full results of zero-point corrected energies of the TSs for each type of reaction and the corresponding reactants, calculated using the DFT/M06-2X method for β-Glucose. The global minimum structure of β-Glucose is used as the energy reference. Black dashes represent reactant states, and dashes with different colors represent TSs of different reactions.

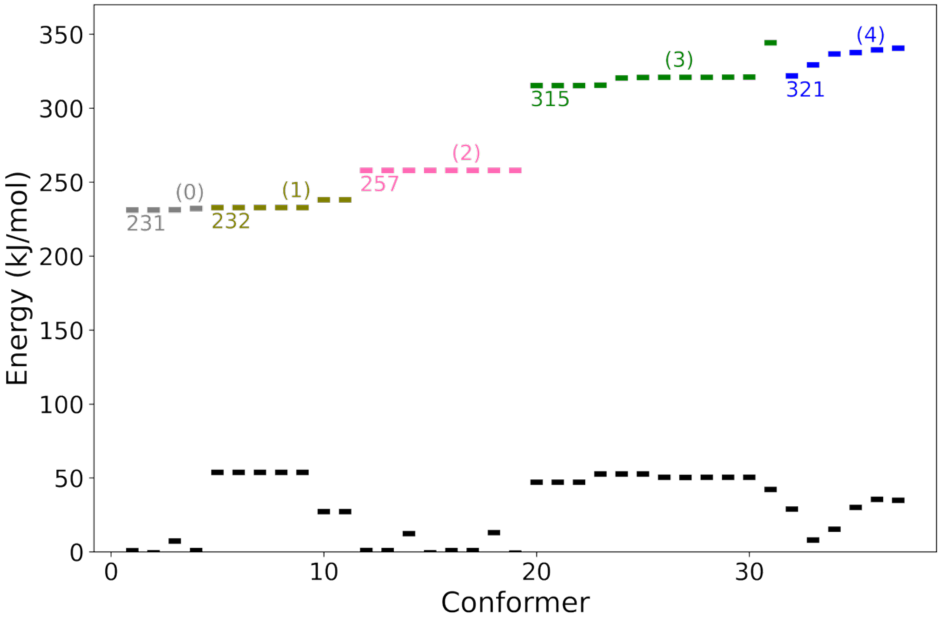

Figure S2. Full results of zero-point corrected energies of the TSs for each type of reaction and the corresponding reactants, calculated using the DFT/M06-2X method for Manβ-Me. The global minimum structure of Manβ-Me is used as the energy reference. Black dashes represent reactant states, and dashes with different colors represent TSs of different reactions.

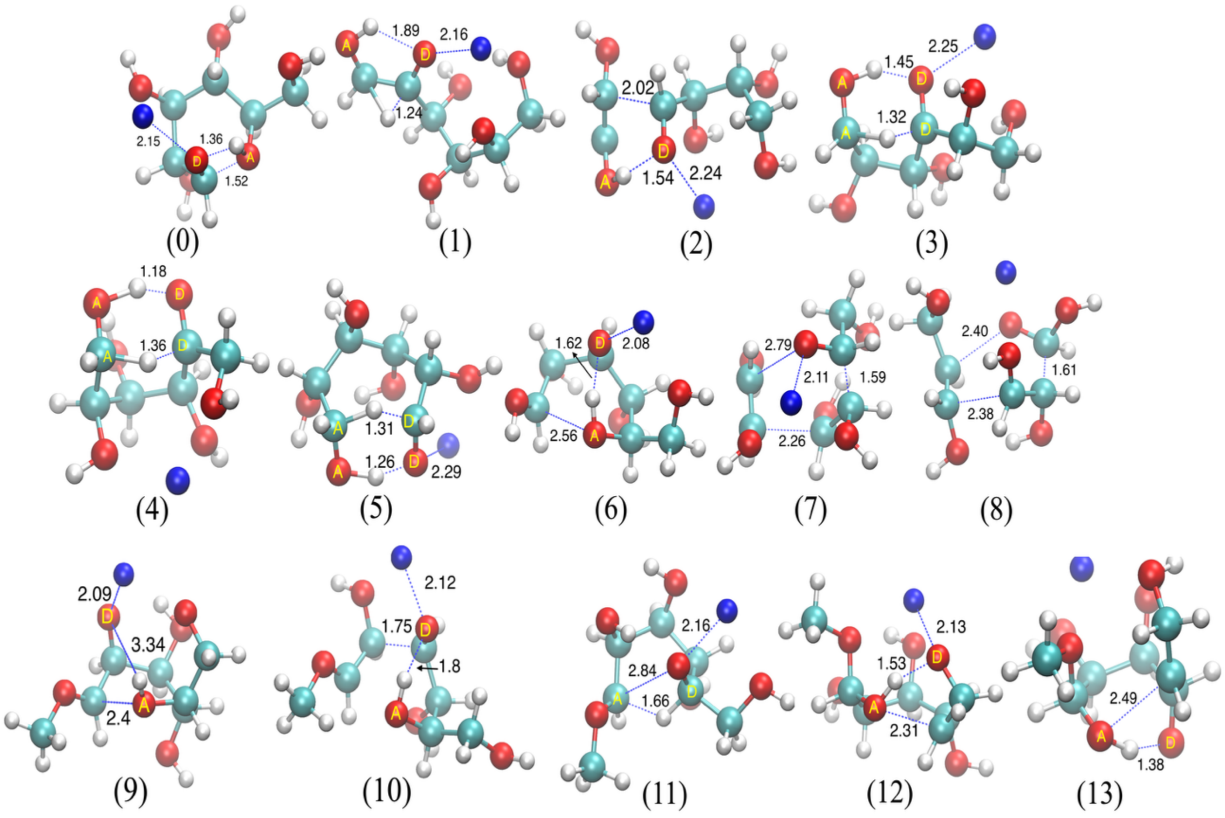

Figure S3. Geometry of the lowest energy transition state of each type of reaction.

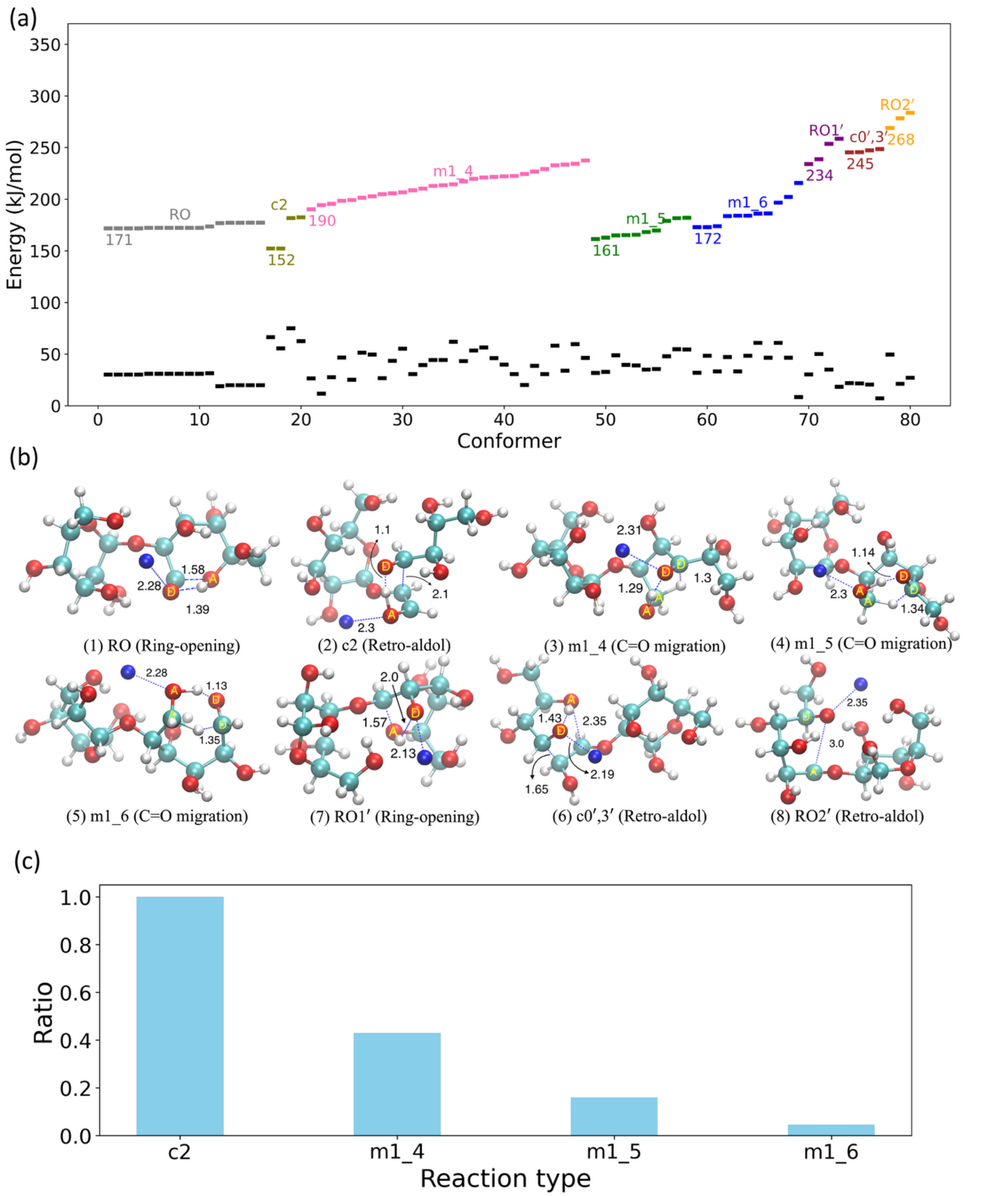

Figure S4. (a) Full results of zero-point corrected energies of the TSs for each type of reaction and the corresponding reactants, calculated using the DFT/M06-2X method for Manβ-(1→2)-Manβ. The global minimum structure of Manβ-(1→2)-Manβ is used as the energy reference. Black dashes represent reactant states, and dashes with different colors represent TSs of different reactions. (b) Geometry of the lowest energy transition state of each type of reaction. (c) Relative ratio of the number of reactant states for each type of reaction.

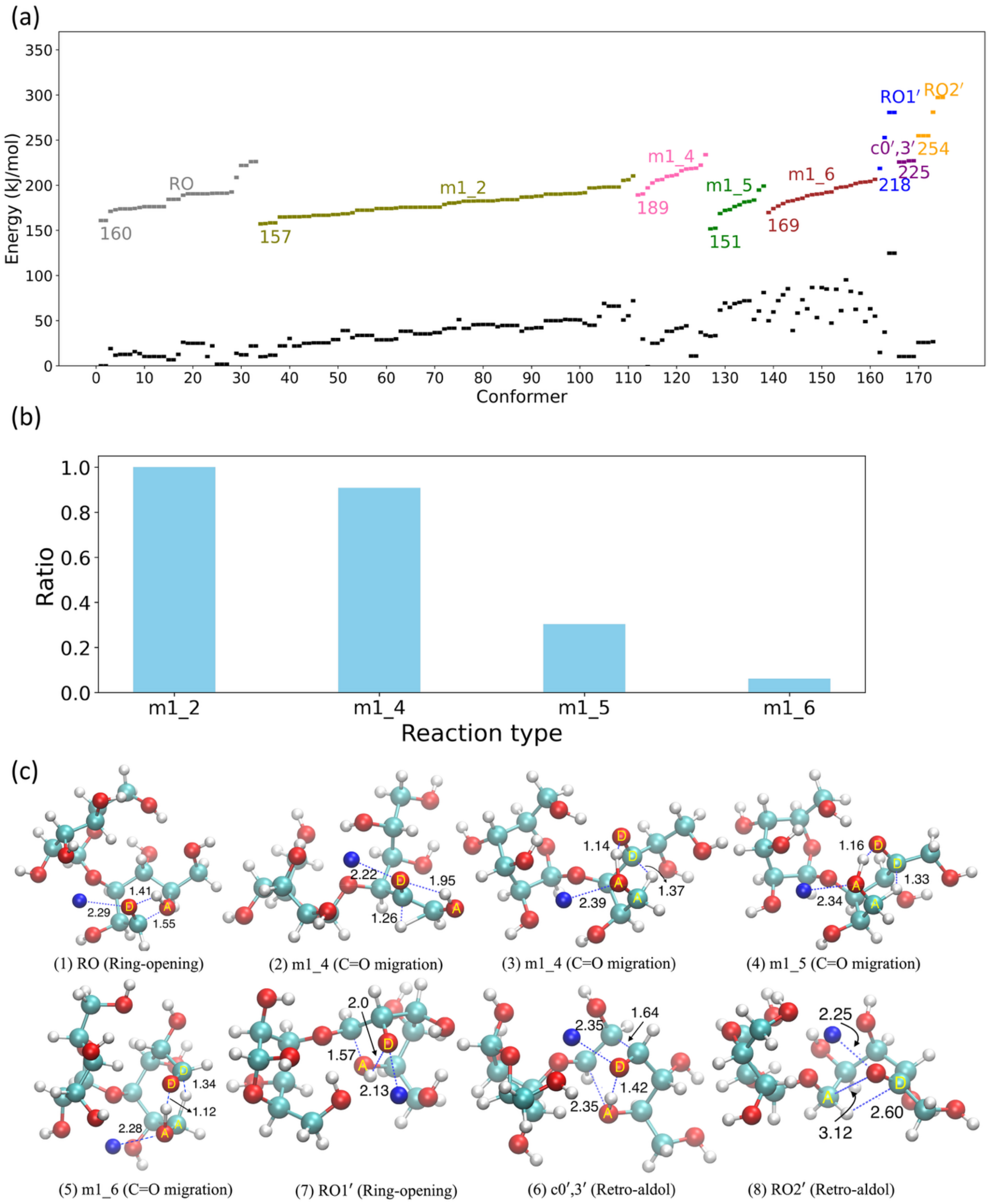

Figure S5. Full results of zero-point corrected energies of the TSs for each type of reaction and the corresponding reactants, calculated using the DFT/M06-2X method for Manβ-(1→3)-Manβ. The global minimum structure of Manβ-(1→3)-Manβ is used as the energy reference. Black dashes represent reactant states, and dashes with different colors represent TSs of different reactions. (b) Relative ratio of the number of reactant states for each type of reaction. (c) Geometry of the lowest energy transition state of each type of reaction.

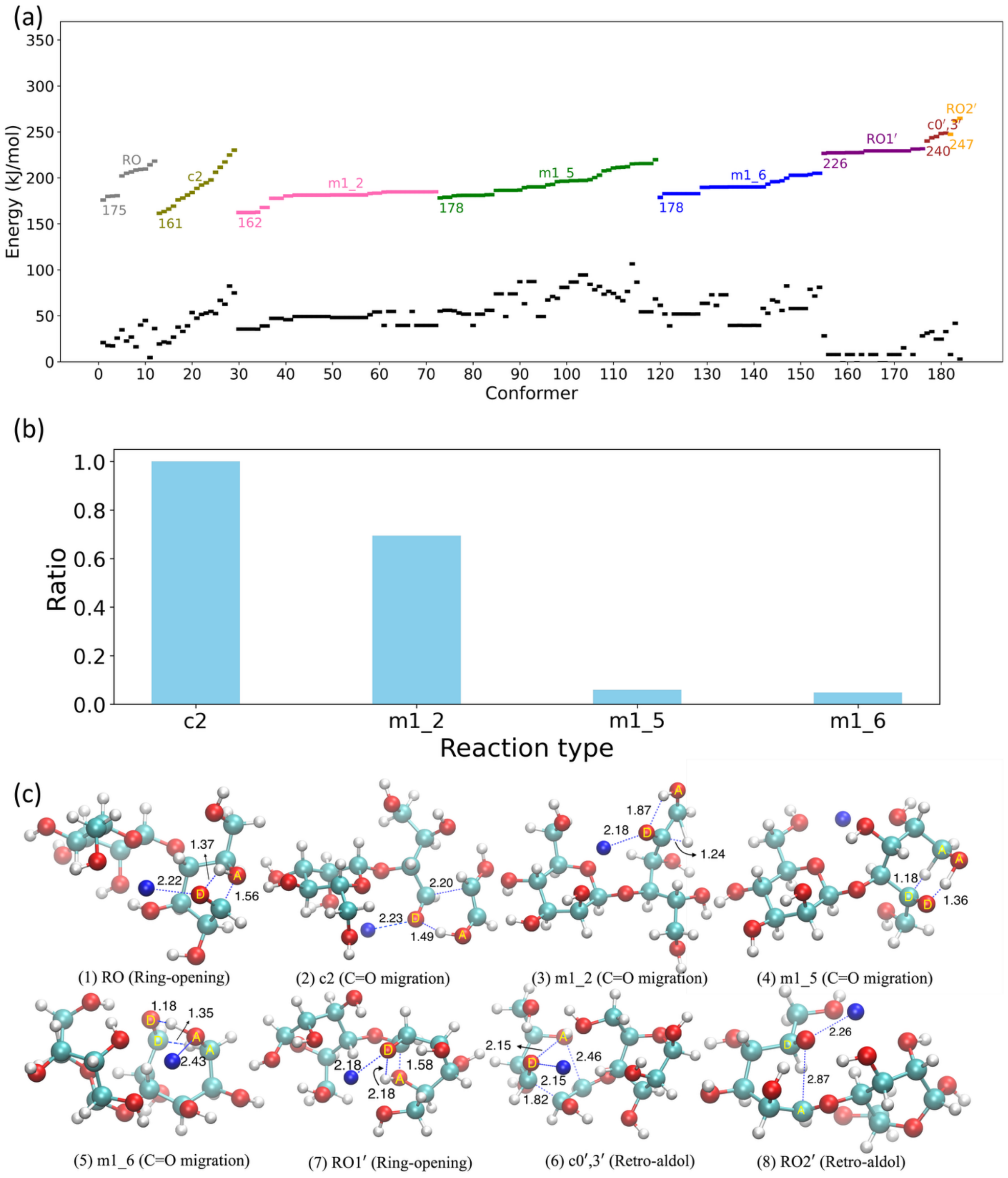

Figure S6. Full results of zero-point corrected energies of the TSs for each type of reaction and the corresponding reactants, calculated using the DFT/M06-2X method for Manβ-(1→4)-Manβ. The global minimum structure of Manβ-(1→4)-Manβ is used as the energy reference. Black dashes represent reactant states, and dashes with different colors represent TSs of different reactions. (b) Relative ratio of the number of reactant states for each type of reaction. (c) Geometry of the lowest energy transition state of each type of reaction.

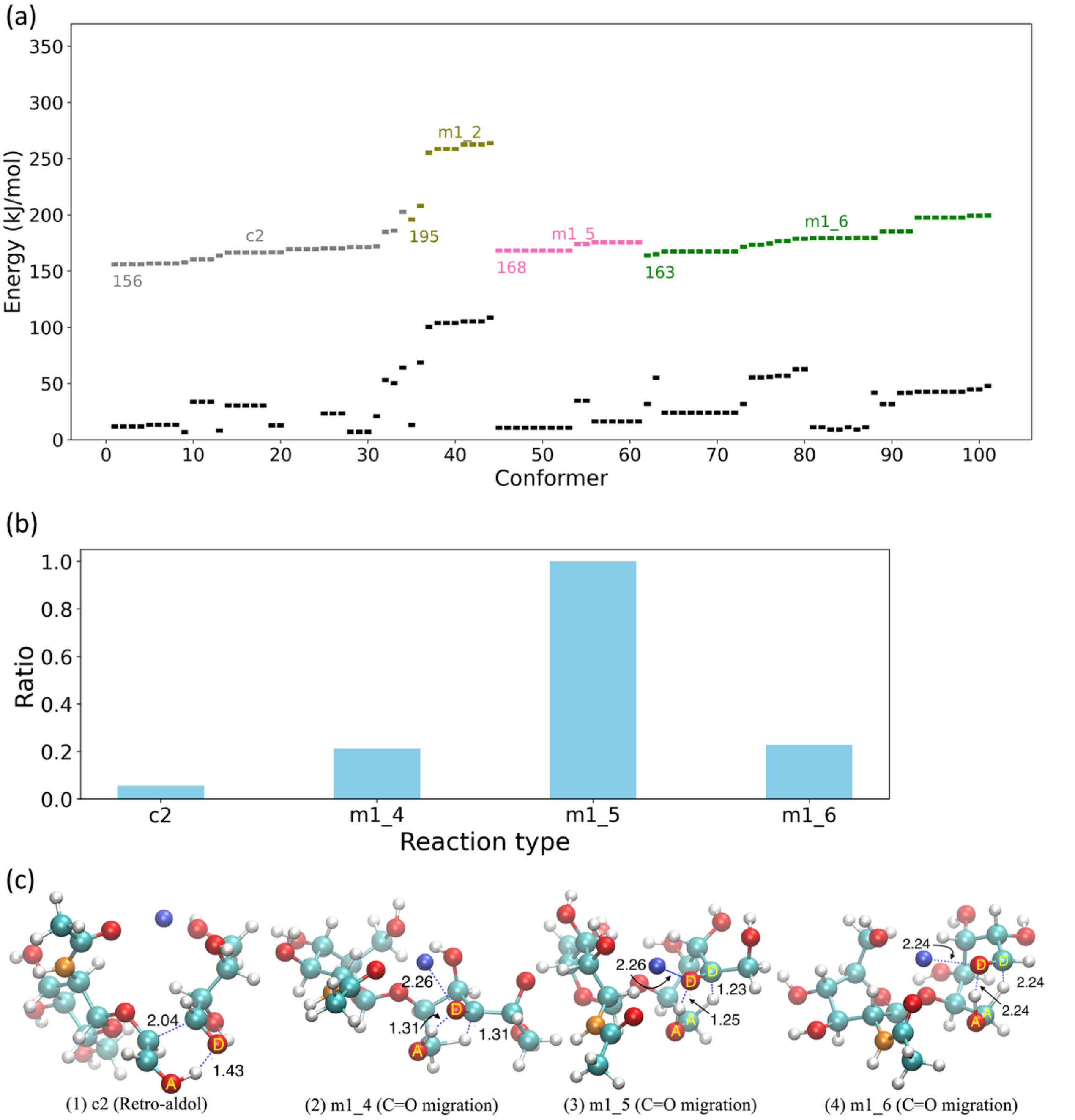

Figure S7. Full results of zero-point corrected energies of the TSs for each type of reaction and the corresponding reactants, calculated using the DFT/M06-2X method for β-GlcNAc -(1→2)-Man (reducing end mannose is in linear form). The global minimum structure of β-GlcNAc-(1→2)-Man is used as the energy reference. Black dashes represent reactant states, and dashes with different colors represent TSs of different reactions. (b) Relative ratio of the number of reactant states for each type of reaction. (c) Geometry of the lowest energy transition state of each type of reaction.

The following are the XYZ coordinates for the transition states and reactant states obtained from DFT/M06-2X method calculation which energies were shown in Figure 4d, Figure 4e, Figure 5e, Figure 6d, Figure 7d and Figure 8b in the main text.

Transition state of 1st (0) reaction of βGlc
25

O -1.119641 -0.452239 -1.639206
O -0.764144 2.494757 0.470612
O -1.726688 -0.830109 1.321824
O 1.277906 -0.520164 2.092246
O 0.659536 0.675194 -1.327772
O 2.637563 -1.282604 -0.679486
C -0.853191 0.828827 -1.269968
C -1.289957 1.230905 0.132291
C -0.779184 0.243826 1.191967
C 0.607986 -0.318466 0.873108
C 1.389247 0.616543 -0.060977
C 2.774497 0.094909 -0.375296
H 0.23323 -0.339821 -1.682394
H -1.264959 3.204766 0.051314
H -1.722504 -1.138075 2.236868
H 1.966972 -1.187118 1.975035
H 3.44946 -1.621606 -1.072819
H -1.080831 1.595609 -2.020395
H -2.388159 1.222309 0.159902
H -0.71804 0.798825 2.133201
H 0.477599 -1.270315 0.343649
H 1.439953 1.63635 0.325822
H 3.186768 0.662233 -1.216693
H 3.411883 0.25341 0.503076
Na -2.34582 -1.879501 -0.599005

Reactant state of 1st (0) reaction of βGlc
25

C -0.331811 1.700026 0.328801
C 1.631322 0.369462 0.302321
C -1.177783 0.571107 -0.251534
C -0.602457 -0.731799 0.243902
C 0.847179 -0.858012 -0.197738
O -0.887738 2.873332 -0.147506
O 0.987447 1.561162 -0.137758
O -2.539855 0.668881 0.165929
O -1.384478 -1.821866 -0.248678
O 1.271266 -2.082105 0.347879
H -0.34158 1.663641 1.433041
H -0.421721 3.640884 0.209397
H 1.679591 0.333563 1.402614
H -1.116605 0.637397 -1.34599
H -2.807552 1.597263 0.078591
H -0.628534 -0.751511 1.344229
H -0.834139 -2.618839 -0.170364
H 0.890824 -0.875415 -1.299627
H 2.229727 -2.165734 0.218833
C 3.036111 0.432986 -0.280402
H 3.584886 1.258081 0.185222
H 2.958281 0.620264 -1.35889
O 3.649178 -0.819396 -0.016711
H 4.574039 -0.807247 -0.28635
Na -3.540831 -1.315912 -0.089823

Transition state of 1st (1) reaction of βGlc
25

C -2.84874 0.304348 0.069041
C -1.427286 0.054789 0.063924
C -0.515978 1.06011 0.796326
O -0.011507 0.501315 1.989439
C 0.674047 1.602469 -0.015579
C 1.826007 0.681135 -0.456078
C 2.52353 -0.153915 0.615537
O 1.779824 -1.296903 1.000747
O 1.403734 -0.199326 -1.489428
O 0.096316 2.145032 -1.198964
O -1.111982 -1.193465 -0.111087
O -3.599618 -0.689991 -0.247789
H 2.588345 1.363928 -0.861938
H -3.327868 1.271762 0.203033
H -1.708158 0.683162 -0.964902
H -1.121913 1.951041 1.01401
H 1.115361 2.388889 0.609642
H -2.982406 -1.472133 -0.271713
H 3.462161 -0.525056 0.197611
H 2.759431 0.474518 1.481584
H 0.428558 3.032315 -1.378525
H -0.690233 0.463108 2.674622
H 1.070351 -0.997917 1.59338
H 0.995247 0.337447 -2.183515
Na 0.687957 -2.217998 -0.73597

Reactant state of 1st (1) reaction of βGlc
25

C -2.910143 0.264718 -0.212107
C -1.412753 0.194845 -0.482627
C -0.681012 1.036616 0.593467
O -0.521141 0.309013 1.795901
C 0.681611 1.619979 0.177115
C 1.873608 0.691028 -0.09349
C 2.293526 -0.278218 1.008562
O 1.454349 -1.418089 1.095263
O 1.643623 -0.063022 -1.283077
O 0.437693 2.32652 -1.03451
O -1.039075 -1.165287 -0.481661
O -3.525159 -0.73626 0.062065
H 2.728004 1.363049 -0.261406
H -3.392964 1.256551 -0.254891
H -1.218618 0.650835 -1.462354
H -1.299672 1.928378 0.766018
H 0.977665 2.299585 0.986331
H -1.83519 -1.655699 -0.197876
H 3.293714 -0.650897 0.774597
H 2.342563 0.249399 1.967372
H 0.744485 3.238587 -0.979382
H -1.253622 0.449151 2.407493
H 0.631872 -1.148231 1.53884
H 1.475108 0.568384 -1.99754
Na 0.92139 -2.158718 -0.951325

Transition state of 1st (2) reaction of βGlc
25

C 2.521933 0.3801 -0.614258
C 2.534947 -0.577834 0.387733
C 0.623243 -0.236368 0.931459
O 0.480452 1.02534 1.134855
C -0.194588 -0.895538 -0.164767
C -1.627093 -1.241759 0.303411
C -2.387569 -0.127232 1.01763
O -2.636974 0.972989 0.14311
O -2.351989 -1.560436 -0.882179
O -0.235664 0.002746 -1.268688
O 2.588364 -1.899176 0.017932
O 2.316042 1.634682 -0.331245
H -1.56364 -2.103384 0.979576
H 2.538955 0.104071 -1.668045
H 2.902721 -0.302876 1.37427
H 0.78764 -0.890009 1.799846
H 0.294836 -1.830385 -0.464986
H 3.357802 -2.33997 0.40007
H -3.338112 -0.525637 1.387178
H -1.822368 0.264006 1.866071
H -0.79001 -0.414956 -1.945363
H 1.742831 1.653214 0.517783
H -3.273681 0.676905 -0.521682
H -2.678028 -2.467139 -0.868152
Na -0.751458 2.087162 -0.412191

Reactant state of 1st (2) reaction of βGlc
25

C 2.376168 0.45284 -0.649966
C 2.147073 -0.576377 0.459603
C 0.685416 -0.593043 0.969154
O 0.307754 0.694702 1.438615
C -0.267933 -1.022984 -0.1383
C -1.761382 -1.12416 0.22691
C -2.41437 0.089952 0.881456
O -2.288505 1.260439 0.072333
O -2.406026 -1.34202 -1.028747
O -0.088328 -0.121068 -1.230063
O 2.426259 -1.871862 -0.000987
O 2.12623 1.633002 -0.531164
H -1.888923 -1.985693 0.89546
H 2.815032 0.04274 -1.578423
H 2.790858 -0.286007 1.304433
H 0.627598 -1.334107 1.776602
H 0.055748 -2.023214 -0.451386
H 3.353871 -2.103388 0.123147
H -3.475019 -0.133454 1.040242
H -1.959525 0.316901 1.844302
H -0.652758 -0.43858 -1.951401
H 0.845884 0.945491 2.200317
H -2.901843 1.168943 -0.668605
H -3.008385 -2.09327 -0.994218
Na -0.164676 2.049157 -0.344864

Transition state of 1st (3) reaction of βGlc
25

C -2.135778 1.16817 0.409011
C -2.296059 -0.228825 -0.229506
C -0.998332 -0.956958 0.136559
O -0.510542 -1.852639 -0.830564
C -0.002103 0.195987 0.33211
C 1.210469 -0.072537 1.247555
C 1.95225 -1.318882 0.75289
O 2.247144 -1.207336 -0.63413
O 2.1374 0.999907 1.155318
O 0.233574 0.936172 -0.719305
O -3.374007 -0.951506 0.295393
O -1.781248 2.155665 -0.351973
H 0.893886 -0.234441 2.284518
H -2.741492 1.418906 1.284818
H -2.357923 -0.098845 -1.317784
H -1.17009 -1.45907 1.102455
H -0.87809 0.852999 1.071621
H -4.199129 -0.725304 -0.15108
H 2.900253 -1.387484 1.291333
H 1.364353 -2.21899 0.951738
H -0.906288 1.794458 -0.831356
H -1.085211 -2.627724 -0.887631
H 1.510708 -1.615002 -1.119636
H 1.85761 1.747226 1.698246
Na 2.437287 1.017222 -1.144261

Reactant state of 1st (3) reaction of βGlc
25

C -2.814343 1.107636 0.189763
C -2.275428 -0.228361 -0.296449
C -1.00977 -0.697325 0.451775
O -0.50312 -1.811988 -0.25706
C 0.059528 0.391091 0.560059
C 1.396041 -0.063237 1.195393
C 2.064505 -1.300201 0.6052
O 2.154467 -1.209453 -0.814601
O 2.346794 0.997364 1.033765
O 0.313493 0.993784 -0.710292
O -3.198249 -1.278919 -0.130565
O -2.203018 2.134499 -0.010496
H 1.219227 -0.251005 2.262019
H -3.784898 1.10068 0.718701
H -1.98733 -0.100774 -1.349871
H -1.306563 -0.998363 1.468404
H -0.340149 1.163925 1.228464
H -3.864177 -1.278141 -0.828606
H 3.084899 -1.346098 0.993158
H 1.530931 -2.204619 0.898103
H -0.383195 1.659847 -0.842281
H -1.217674 -2.460089 -0.344809
H 1.316982 -1.554446 -1.164251
H 2.195893 1.705443 1.671503
Na 2.520901 0.973066 -1.257363

Transition state of 2nd (3) reaction of βGlc
25

C -2.113701 1.176596 0.42452
C -2.298447 -0.214128 -0.222882
C -1.006308 -0.96092 0.125589
O -0.527747 -1.845812 -0.856373
C -0.000665 0.181666 0.326693
C 1.201885 -0.110554 1.237842
C 1.938723 -1.352753 0.717616
O 2.249652 -1.19571 -0.660466
O 2.062957 1.02085 1.155135
O 0.240303 0.918142 -0.724768
O -3.38035 -0.927407 0.307726
O -1.758115 2.165886 -0.334412
H 0.868628 -0.279248 2.268224
H -2.70914 1.428509 1.306952
H -2.369821 -0.075456 -1.309416
H -1.177779 -1.475373 1.084913
H -0.858314 0.848813 1.072581
H -4.207243 -0.684093 -0.126204
H 2.882999 -1.456744 1.259976
H 1.343783 -2.256133 0.881365
H -0.890229 1.804093 -0.819731
H -1.111415 -2.612735 -0.928381
H 1.514057 -1.579386 -1.166694
H 2.57658 1.113835 1.96599
Na 2.42867 1.074262 -1.112781

Reactant state of 2nd (3) reaction of βGlc
25

C 2.805272 -1.110026 0.212038
C 2.27236 0.221904 -0.292619
C 1.007867 0.705593 0.447186
O 0.505195 1.812984 -0.277107
C -0.063142 -0.378767 0.563661
C -1.395131 0.086113 1.189063
C -2.061604 1.31873 0.584902
O -2.160382 1.196192 -0.83113
O -2.279685 -1.036346 1.033156
O -0.309304 -0.988102 -0.706134
O 3.199482 1.271071 -0.139163
O 2.194071 -2.138041 0.019448
H -1.206267 0.271243 2.254059
H 3.772776 -1.098329 0.746634
H 1.985412 0.081445 -1.344532
H 1.303516 1.018799 1.460369
H 0.318405 -1.149925 1.241152
H 3.86844 1.255267 -0.834079
H -3.082343 1.386473 0.973767
H -1.525435 2.228996 0.856925
H 0.36588 -1.681193 -0.808162
H 1.224291 2.454013 -0.378582
H -1.324593 1.534761 -1.19177
H -2.884972 -1.092366 1.78179
Na -2.491261 -1.01932 -1.252319

Transition state of 3rd (3) reaction of βGlc
25

C -2.128458 1.149694 0.402035
C -2.379813 -0.24545 -0.20577
C -1.052411 -1.010764 -0.010138
O -0.647212 -1.828898 -1.063825
C -0.034609 0.108247 0.273677
C 1.139353 -0.223602 1.210702
C 1.942149 -1.402413 0.670177
O 2.494658 -1.013636 -0.588534
O 2.031222 0.880937 1.297908
O 0.243434 0.864157 -0.761936
O -3.397582 -0.917637 0.479591
O -1.755638 2.122797 -0.372998
H 0.760385 -0.505727 2.199559
H -2.696847 1.432684 1.292911
H -2.598286 -0.118449 -1.274196
H -1.187928 -1.652322 0.870015
H -0.874635 0.780426 1.028375
H -4.254871 -0.781682 0.059584
H 2.743706 -1.628005 1.38093
H 1.289426 -2.272185 0.547859
H -0.904831 1.747483 -0.866025
H -0.36442 -1.262597 -1.796778
H 2.88784 -1.781158 -1.01974
H 1.756082 1.493002 1.990917
Na 2.434186 1.221137 -0.94356

Reactant state of 3rd (3) reaction of βGlc
25

C -2.828617 1.102778 0.172651
C -2.345202 -0.257386 -0.289961
C -1.0353 -0.756461 0.37329
O -0.580034 -1.850533 -0.399083
C 0.026441 0.33121 0.546768
C 1.348122 -0.145835 1.183502
C 2.046556 -1.351931 0.580399
O 2.312705 -1.077092 -0.804117
O 2.295672 0.928889 1.121498
O 0.293332 0.960696 -0.711083
O -3.383386 -1.16793 -0.06906
O -2.164045 2.1016 -0.010706
H 1.135638 -0.401365 2.228997
H -3.820202 1.14082 0.654706
H -2.132005 -0.141199 -1.366436
H -1.298174 -1.165566 1.356477
H -0.372924 1.090904 1.229922
H -3.135044 -2.031626 -0.424482
H 2.989261 -1.493912 1.118542
H 1.417002 -2.237485 0.675087
H -0.426982 1.606857 -0.836788
H -0.167738 -1.503737 -1.204942
H 2.749124 -1.840429 -1.201358
H 2.156492 1.557911 1.839602
Na 2.49451 1.156321 -1.135348

Transition state of 1st (4) reaction of βGlc
25

C -1.037366 -0.012881 -1.487213
C -1.119802 1.229627 -0.60742
C -1.21165 0.839038 0.880326
O -2.481298 0.350563 1.200226
C -0.081696 -0.144319 1.295225
C 0.169055 -1.249248 0.259503
C 1.587393 -1.764146 0.030965
O 2.390898 -0.78248 -0.636767
O -0.764286 -2.143907 0.18705
O 1.140594 0.562746 1.531713
O 0.05714 2.020291 -0.833407
O -1.968436 -0.906291 -1.369825
H 0.036768 -0.527165 -0.889559
H -0.589328 0.135481 -2.481101
H -2.021708 1.797399 -0.859117
H -1.067443 1.748332 1.474426
H -0.414912 -0.6514 2.207212
H -0.173563 2.789583 -1.367572
H 2.030976 -2.014837 1.001482
H 1.494924 -2.666227 -0.577385
H 1.184339 0.823981 2.459234
H -2.766548 -0.31532 0.557543
H 3.11218 -1.232885 -1.092688
H -1.546451 -1.727468 -0.596118
Na 2.171661 1.422248 -0.245146

Reactant state of 1st (4) reaction of βGlc
25

C 1.828295 -0.351425 -1.408835
C 1.52687 -1.153409 -0.143187
C 1.205155 -0.32932 1.125528
O 2.248473 0.558487 1.391772
C -0.178316 0.357009 1.154705
C -0.555641 1.091729 -0.136011
C -2.036494 1.462126 -0.228274
O -2.734007 0.322979 -0.75849
O 0.266153 2.232443 -0.135691
O -1.206137 -0.59243 1.459039
O 0.455329 -2.065074 -0.443086
O 2.291935 0.760566 -1.424993
H -0.355141 0.455581 -1.016442
H 1.587304 -0.889067 -2.350556
H 2.441831 -1.715055 0.087565
H 1.196574 -1.046499 1.955631
H -0.130216 1.119198 1.94207
H 0.824471 -2.920083 -0.695677
H -2.440484 1.735754 0.751953
H -2.134381 2.310625 -0.911731
H -1.240188 -0.742496 2.410807
H 2.26928 1.247286 0.709252
H -3.620917 0.586497 -1.030311
H 0.525169 2.480501 -1.032566
Na -1.839681 -1.710751 -0.33745

Transition state of 2nd (4) reaction of βGlc
25

C -1.037707 -0.013073 -1.487139
C -1.11957 1.229665 -0.607592
C -1.211301 0.839431 0.880247
O -2.481049 0.351405 1.20043
C -0.081569 -0.144191 1.295149
C 0.168849 -1.249236 0.25947
C 1.587012 -1.7644 0.030616
O 2.390652 -0.782737 -0.636935
O -0.764619 -2.143825 0.187348
O 1.140916 0.562485 1.53173
O 0.057559 2.019986 -0.833895
O -1.968996 -0.906146 -1.369275
H 0.036371 -0.527505 -0.889663
H -0.589907 0.134914 -2.481188
H -2.021312 1.797728 -0.859216
H -1.066733 1.748801 1.474141
H -0.414966 -0.651201 2.207111
H -0.17298 2.788694 -1.368972
H 2.030616 -2.015533 1.001015
H 1.494224 -2.666264 -0.578013
H 1.184678 0.823678 2.459261
H -2.766529 -0.314604 0.557965
H 3.112595 -1.233106 -1.091853
H -1.546987 -1.727355 -0.595443
Na 2.171904 1.422112 -0.244989

Reactant state of 2nd (4) reaction of βGlc
25

C 1.828289 -0.351423 -1.408837
C 1.526868 -1.15341 -0.143191
C 1.205157 -0.329323 1.125527
O 2.248475 0.558482 1.391772
C -0.178314 0.357006 1.154707
C -0.555639 1.091729 -0.136009
C -2.036492 1.462128 -0.22827
O -2.734007 0.322983 -0.758492
O 0.266158 2.23244 -0.135689
O -1.206136 -0.592431 1.45904
O 0.455325 -2.065072 -0.443087
O 2.291938 0.760564 -1.424994
H -0.355142 0.45558 -1.01644
H 1.587287 -0.889059 -2.350559
H 2.441829 -1.715058 0.087558
H 1.196576 -1.046505 1.955628
H -0.130212 1.119195 1.942072
H 0.824462 -2.920082 -0.695683
H -2.440482 1.735751 0.751958
H -2.134379 2.310631 -0.911723
H -1.240185 -0.7425 2.410807
H 2.269283 1.247282 0.709252
H -3.620914 0.586506 -1.030319
H 0.525169 2.480501 -1.032565
Na -1.839684 -1.710747 -0.337451

Transition state of 3rd (4) reaction of βGlc
25

C -0.281496 -1.095117 -1.083689
C -1.730539 -0.760699 -0.740264
C -1.876282 -0.149865 0.666898
O -1.616225 -1.190526 1.574672
C -0.910409 1.051443 0.837889
C 0.513019 0.64432 0.450719
C 1.482621 1.723209 8.9e-05
O 2.599885 1.036612 -0.576193
O 1.063303 -0.336928 1.130354
O -1.320308 2.177163 0.109924
O -2.139079 0.193798 -1.707043
O 0.39775 -1.939823 -0.366222
H 0.233966 0.126239 -0.74792
H -0.053248 -1.041798 -2.15521
H -2.312755 -1.688594 -0.782018
H -2.899732 0.237808 0.760199
H -0.887179 1.328061 1.899972
H -3.013156 -0.013645 -2.057155
H 1.008766 2.391124 -0.720184
H 1.794757 2.295446 0.882317
H -1.737313 1.89367 -0.718627
H -1.890915 -0.944506 2.466099
H 3.17916 1.675305 -1.008699
H 0.586513 -1.335518 0.65555
Na 2.811549 -1.175065 -0.053465

Reactant state of 3rd (4) reaction of βGlc
25

C -1.376392 -1.739772 -0.676898
C -2.137067 -0.442989 -0.365282
C -1.657982 0.479614 0.772458
O -1.438864 -0.386742 1.881849
C -0.432169 1.390427 0.509958
C 0.81798 0.594002 0.146858
C 2.047745 1.395893 -0.214408
O 2.979321 0.399214 -0.675478
O 1.21046 -0.268127 1.213291
O -0.713748 2.368534 -0.456441
O -2.141909 0.251918 -1.606819
O -0.271154 -2.054724 -0.297873
H 0.573352 0.011047 -0.757595
H -1.927544 -2.390476 -1.381019
H -3.149457 -0.769501 -0.09179
H -2.491054 1.165295 0.971606
H -0.237413 1.924064 1.449911
H -3.030766 0.321719 -1.974945
H 1.818833 2.111409 -1.00764
H 2.445659 1.911028 0.665771
H -1.114006 1.944716 -1.229467
H -1.734281 0.0247 2.703237
H 3.800163 0.815585 -0.961463
H 0.40876 -0.612736 1.6445
Na 2.188382 -1.727318 -0.139587

Transition state of 1st (5) reaction of βGlc
25

C 1.536902 -0.974375 -0.92997
C 2.044782 -0.124684 0.251319
C 1.347082 1.242259 0.401152
O 1.53958 1.998978 -0.775786
C -0.181137 1.203883 0.637294
C -1.008278 1.071083 -0.6494
C -0.771077 -0.222179 -1.436811
O -1.175414 -1.34128 -0.89999
O -2.379877 1.06948 -0.239227
O -0.575903 0.184452 1.549069
O 1.884288 -0.809546 1.474537
O 1.011601 -2.126733 -0.626952
H -0.7882 1.928864 -1.288854
H 2.128991 -0.923953 -1.851452
H 3.109955 0.077503 0.067067
H 1.800301 1.723877 1.275874
H -0.46168 2.173891 1.065691
H 1.960595 -1.764004 1.320956
H -0.87099 -0.140245 -2.528706
H 0.541853 -0.24133 -1.421697
H 0.218975 -0.286056 1.865513
H 2.360012 2.504627 -0.734502
H -0.116939 -1.995159 -0.696146
H -2.914173 1.64032 -0.80287
Na -2.473867 -0.879733 0.930402

Reactant state of 1st (5) reaction of βGlc
25

C 2.244324 -0.658567 -1.091344
C 2.33567 -0.021968 0.284727
C 1.283402 1.07673 0.55738
O 1.158284 1.898306 -0.594159
C -0.148543 0.62797 1.003918
C -1.26768 1.167203 0.091523
C -1.238576 0.508777 -1.288249
O -1.959195 -0.435538 -1.540591
O -2.477391 0.817812 0.757908
O -0.386917 -0.767376 1.120604
O 2.216406 -0.957783 1.349935
O 0.935708 -1.157844 -1.284243
H -1.162982 2.250362 -0.013331
H 2.990253 -1.463327 -1.15167
H 3.318356 0.468415 0.33676
H 1.674365 1.669713 1.391087
H -0.341494 1.079557 1.984175
H 2.983056 -1.542009 1.389727
H -0.550157 0.930286 -2.032693
H 2.493063 0.102131 -1.840587
H 0.457073 -1.223219 1.290073
H 1.80527 2.612642 -0.574488
H 0.874182 -1.584494 -2.147112
H -3.190405 1.407107 0.483709
Na -2.408847 -1.462604 0.460089

Transition state of 2nd (5) reaction of βGlc
25

C 1.355378 0.03675 -1.297988
C 1.536537 0.973247 -0.078422
C 0.248951 1.701029 0.358722
O -0.199774 2.476749 -0.737553
C -0.974532 0.867975 0.778471
C -1.802122 0.2544 -0.361747
C -1.034174 -0.675629 -1.303329
O -0.575846 -1.807051 -0.852166
O -2.808127 -0.47306 0.31255
O -0.600841 -0.142407 1.707073
O 2.062458 0.2617 1.028219
O 1.632417 -1.22699 -1.104961
H -2.213038 1.075611 -0.958009
H 1.626755 0.459924 -2.272503
H 2.232115 1.766748 -0.378919
H 0.524785 2.318908 1.220467
H -1.653703 1.572901 1.274092
H 3.026229 0.247408 0.97645
H 0.061695 0.095846 -1.506349
H -1.362901 -0.66749 -2.353056
H -1.434284 -0.516712 2.033749
H 0.149587 3.374645 -0.699297
H 0.57117 -1.786631 -1.14386
H -3.577077 -0.629849 -0.248371
Na 0.907831 -1.724498 1.150552

Reactant state of 2nd (5) reaction of βGlc
25

C 1.506672 0.333648 -1.34604
C 1.43604 1.184928 -0.047767
C 0.021871 1.627351 0.361188
O -0.537286 2.37536 -0.704072
C -1.073733 0.602364 0.737077
C -1.86908 -0.056196 -0.422255
C -1.485486 -1.482524 -0.773169
O -0.392965 -1.972262 -0.585553
O -3.238482 -0.140156 -0.082771
O -0.559643 -0.34331 1.669622
O 2.035227 0.529099 1.063679
O 2.225316 -0.860627 -1.049103
H -1.723494 0.542148 -1.334121
H 2.021433 0.897208 -2.129014
H 1.973189 2.123771 -0.228846
H 0.157888 2.243027 1.258934
H -1.831858 1.215582 1.236438
H 2.994741 0.610175 0.99506
H 0.501476 0.097609 -1.7107
H -2.300405 -2.06472 -1.236369
H -1.233611 -0.538531 2.331543
H -0.285783 3.30434 -0.645666
H 2.363271 -1.358808 -1.863559
H -3.72273 0.626848 -0.409125
Na 1.237348 -1.605537 0.926074

Transition state of 3rd (5) reaction of βGlc
25

C -0.580679 1.676725 -0.074641
C 0.479852 1.157505 -1.055607
C 1.71823 0.683399 -0.267273
O 1.94319 1.667673 0.724462
C 1.605328 -0.712771 0.393613
C 0.267529 -1.140121 1.082674
C -0.551702 -0.010347 1.721098
O -1.840488 -0.062645 1.603109
O -0.569886 -1.827001 0.174271
O 1.854539 -1.709356 -0.594994
O -0.085954 0.1223 -1.83841
O -1.836713 1.345907 -0.245126
H 0.529488 -1.82882 1.898695
H -0.377689 2.687292 0.297363
H 0.773475 1.998986 -1.69655
H 2.568571 0.613819 -0.957913
H 2.384873 -0.727058 1.163942
H 0.619397 -0.37467 -2.275577
H -0.208812 1.061461 0.997043
H -0.134329 0.401944 2.649157
H 2.749937 -2.059939 -0.522048
H 2.879277 1.881201 0.807083
H -2.101841 0.721961 0.753346
H 0.005675 -2.455709 -0.28837
Na -2.09426 -0.740166 -1.110168

Reactant state of 3rd (5) reaction of βGlc
25

C -2.398088 0.704551 0.020036
C -1.084438 1.373597 -0.370408
C -0.046763 1.392394 0.793664
O -0.436682 0.540612 1.870857
C 1.414626 1.026809 0.438438
C 1.780702 -0.479354 0.245672
C 1.049704 -1.420363 1.189198
O 0.207316 -2.19077 0.784799
O 1.551549 -0.941741 -1.064155
O 1.800818 1.690286 -0.756838
O -0.640199 0.676289 -1.526663
O -2.218615 -0.697472 0.172867
H 2.852323 -0.56416 0.480745
H -3.110387 0.843184 -0.795923
H -1.298 2.414098 -0.644168
H -0.014561 2.426571 1.156556
H 2.020082 1.371885 1.285225
H 0.116221 1.150606 -1.897898
H -2.81526 1.152652 0.926199
H 1.347423 -1.399657 2.248159
H 2.408133 2.419107 -0.584384
H -0.520097 1.037183 2.692786
H -1.883005 -0.867144 1.064728
H 2.058355 -0.380675 -1.66725
Na -0.688107 -1.618825 -1.273755

Transition state of 1st (6) reaction of βGlc
25

C -2.043557 -0.478638 -0.930946
O 0.40823 -0.083817 -1.545945
C 0.804241 1.063693 -0.824692
C 0.078017 1.144853 0.545875
O -0.994068 2.083672 0.484216
C -0.503179 -0.218731 1.013623
C -2.072357 -0.484159 0.51141
O -2.989325 0.402684 1.061226
O 0.286184 -1.250642 0.691169
C 2.31843 1.204806 -0.749454
O 2.928855 0.190864 0.071261
O -1.813765 -1.576795 -1.546427
H 0.474892 1.932677 -1.409763
H 0.795006 1.450175 1.320627
H -0.745668 -0.132529 2.088582
H -2.243883 -1.496004 0.886813
H -2.189763 0.442239 -1.497523
H -1.613818 -1.444947 -2.490785
H -2.595486 1.288483 1.088139
H 0.469508 -0.814352 -0.86087
H -0.675549 2.989825 0.5694
H 2.562126 2.190606 -0.334603
H 2.716526 1.13256 -1.766344
H 3.874134 0.3859 0.093571
Na 2.279412 -1.767572 1.017623

Reactant state of 1st (6) reaction of βGlc
25

C 1.618025 -0.978411 -0.20414
O 0.236534 -1.373327 -0.065056
C -0.376335 -0.766432 1.057942
C -0.203998 0.778119 1.017689
O 0.767543 1.217537 1.947658
C 0.343504 1.189628 -0.355296
C 1.695557 0.50581 -0.625063
O 2.771622 1.174866 -0.034602
O -0.603186 0.830471 -1.368058
C -1.806053 -1.264406 1.144663
O -2.608739 -0.811798 0.034903
O 2.174038 -1.738949 -1.201923
H 0.12143 -1.118755 1.977202
H -1.167076 1.279985 1.206943
H 0.480419 2.272425 -0.398763
H 1.868599 0.527599 -1.706259
H 2.109856 -1.123165 0.772115
H 2.315832 -2.645611 -0.901787
H 2.594294 1.298793 0.908384
H -0.499954 -0.129005 -1.501891
H 0.430007 1.214577 2.851018
H -2.256562 -0.911962 2.079279
H -1.784753 -2.357014 1.146506
H -3.275412 -1.489444 -0.130585
Na -2.769399 1.091395 -1.183665

Transition state of 2nd (6) reaction of βGlc
25

C -2.011934 -0.217543 -0.963826
O 0.341493 0.145289 -1.512415
C 0.77634 1.170905 -0.635086
C 0.069073 1.037769 0.733516
O -0.960571 2.00883 0.698478
C -0.456242 -0.408598 0.986056
C -1.991522 -0.607849 0.436536
O -2.836881 0.191855 1.225052
O 0.353774 -1.362517 0.497226
C 2.28823 1.314415 -0.592369
O 2.953709 0.200986 0.036598
O -1.952212 -1.136747 -1.85178
H 0.427682 2.120258 -1.058678
H 0.78329 1.259065 1.541437
H -0.690678 -0.514964 2.062144
H -2.15587 -1.683006 0.54862
H -2.106576 0.83226 -1.243628
H -1.777779 -0.786046 -2.74435
H -3.705616 -0.217525 1.316743
H 0.468655 -0.688083 -0.962807
H -1.560012 1.902101 1.449754
H 2.527595 2.229174 -0.038164
H 2.648828 1.408796 -1.621575
H 3.893419 0.42074 0.057464
Na 2.364445 -1.854448 0.74171

Reactant state of 2nd (6) reaction of βGlc
25

C -0.884165 -1.101582 -0.954987
O 0.404249 -0.517687 -1.015057
C 0.531464 0.895818 -0.804671
C -0.034265 1.336149 0.569182
O -0.602225 2.614205 0.482051
C -1.006857 0.287235 1.133647
C -1.818353 -0.332915 -0.00467
O -2.401012 0.763847 -0.688362
O -0.228538 -0.718609 1.789634
C 2.020665 1.159565 -0.899483
O 2.65522 0.307625 0.066993
O -0.663271 -2.403143 -0.46738
H 0.00757 1.441312 -1.598786
H 0.784118 1.417939 1.291513
H -1.668608 0.790584 1.844974
H -2.573917 -1.031492 0.372139
H -1.32157 -1.125075 -1.961546
H -0.738161 -3.047399 -1.181363
H -3.246612 0.527292 -1.086187
H -0.768485 -1.17519 2.446103
H -1.405164 2.563662 -0.056808
H 2.207782 2.213642 -0.670458
H 2.390196 0.923607 -1.901822
H 3.551356 0.619553 0.23634
Na 1.51047 -1.632177 0.608069

Transition state of 3rd (6) reaction of βGlc
25

C -2.04506 -0.735256 -0.852978
O 0.343906 -0.572613 -1.372697
C 0.72523 0.743369 -0.998713
C 0.25246 1.127012 0.436066
O -0.270328 2.4262 0.481933
C -0.576013 0.013217 1.109759
C -2.185725 -0.135743 0.429618
O -2.715063 1.15782 0.381166
O 0.051895 -1.150733 1.102015
C 2.225123 0.922084 -1.154527
O 2.903247 0.038767 -0.234841
O -1.890388 -2.011383 -0.9215
H 0.243142 1.438004 -1.697492
H 1.142078 1.166556 1.081516
H -0.937835 0.373262 2.087696
H -2.624417 -0.807783 1.170002
H -2.047643 -0.134778 -1.761122
H -1.61403 -2.304195 -1.807147
H -3.660604 1.164201 0.574139
H 0.365349 -1.100089 -0.529331
H -1.2184 2.411622 0.293408
H 2.493346 1.960853 -0.931468
H 2.518761 0.668154 -2.176942
H 3.851636 0.142257 -0.378762
Na 2.156944 -1.42642 1.342868

Reactant state of 3rd (6) reaction of βGlc
25

C 0.883108 -1.102401 0.954892
O -0.404672 -0.517218 1.015117
C -0.530643 0.89644 0.804706
C 0.035737 1.336428 -0.569022
O 0.605305 2.613716 -0.481466
C 1.007009 0.286403 -1.133718
C 1.818003 -0.334715 0.004444
O 2.401995 0.761116 0.688489
O 0.227497 -0.718548 -1.789588
C -2.019664 1.161338 0.899207
O -2.65487 0.309741 -0.067077
O 0.660678 -2.403757 0.467294
H -0.006488 1.441457 1.598972
H -0.782521 1.419473 -1.291368
H 1.669223 0.789053 -1.845119
H 2.57264 -1.0342 -0.372511
H 1.3207 -1.126311 1.961349
H 0.736254 -3.048246 1.180988
H 3.249306 0.5246 1.082644
H 0.766349 -1.175017 -2.44702
H 1.40834 2.561931 0.057122
H -2.20582 2.215553 0.669958
H -2.389508 0.926036 1.901595
H -3.550952 0.622007 -0.236096
Na -1.51239 -1.630699 -0.607792

Transition state of 1st (7) reaction of βGlc
25

C 0.136503 1.695875 -0.889265
O 0.548211 -1.015282 -1.383168
C -0.415801 -1.456358 -0.559038
C -0.156353 -1.043364 0.957429
O 1.183733 -1.424994 1.252116
C -0.355621 0.378106 1.393951
C 0.957047 1.783815 0.198714
O 2.213985 1.197851 0.162072
O -1.530307 0.911756 1.360011
C -1.80843 -0.995866 -1.046859
O -2.720449 -0.836699 0.071607
O -1.066998 2.24215 -0.936946
H -0.442604 -2.562319 -0.465105
H -0.869572 -1.611053 1.574196
H 0.317645 0.755382 2.16228
H 0.80302 2.542259 0.96394
H 0.370809 1.039078 -1.723809
H -1.275563 2.770821 -0.15084
H 2.888266 1.878871 0.287559
H -2.184697 0.292521 0.836461
H 1.230551 -1.876412 2.102011
H -2.21313 -1.737615 -1.737775
H -1.713461 -0.040675 -1.569092
H -3.641285 -0.836418 -0.220315
Na 2.505504 -1.035087 -0.599511

Reactant state of 1st (7) reaction of βGlc
25

C 0.113248 0.996866 -1.189484
O 0.265606 -0.434622 -1.306214
C -0.479382 -1.221841 -0.366025
C -0.045708 -0.850562 1.058854
O 1.255071 -1.446971 1.187641
C 0.076263 0.670944 1.321297
C 0.731087 1.419887 0.14714
O 2.131275 1.122977 0.063625
O -1.157534 1.310573 1.540026
C -1.987293 -1.308528 -0.622538
O -2.710708 -0.387769 0.184874
O -1.182137 1.409337 -1.36605
H -0.10586 -2.236717 -0.538944
H -0.734628 -1.297271 1.786949
H 0.715672 0.792989 2.208551
H 0.570578 2.491384 0.29654
H 0.711657 1.383912 -2.017904
H -1.665019 1.431326 -0.52364
H 2.652685 1.869566 0.378264
H -1.880049 0.668443 1.60308
H 1.54121 -1.430518 2.10968
H -2.304357 -2.327945 -0.375065
H -2.15992 -1.122753 -1.687174
H -3.656423 -0.475502 0.014312
Na 2.459325 -0.961428 -0.707395

Transition state of 2nd (7) reaction of βGlc
25

C 0.233829 1.720389 -0.861789
O 0.471906 -0.980369 -1.425415
C -0.483178 -1.41862 -0.582889
C -0.186296 -1.046317 0.927793
O 1.136608 -1.502433 1.195821
C -0.31618 0.379208 1.395684
C 1.018418 1.734683 0.255688
O 2.242468 1.080202 0.234688
O -1.475191 0.947694 1.429236
C -1.876883 -0.920384 -1.024736
O -2.761526 -0.787885 0.106373
O -0.895912 2.412281 -0.833356
H -0.535007 -2.52558 -0.517735
H -0.915001 -1.590338 1.547114
H 0.379691 0.696231 2.171126
H 0.878837 2.503638 1.01026
H 0.449634 1.053515 -1.695942
H -1.38983 2.320156 -1.659376
H 2.940555 1.704986 0.470313
H -2.15975 0.39585 0.919833
H 1.178345 -1.940923 2.052638
H -2.298447 -1.618301 -1.750951
H -1.762203 0.053367 -1.512865
H -3.689591 -0.825564 -0.156207
Na 2.439788 -1.116064 -0.654136

Reactant state of 2nd (7) reaction of βGlc
25

C 0.129646 1.016093 -1.18576
O 0.238265 -0.404616 -1.342788
C -0.494281 -1.206881 -0.389913
C -0.047184 -0.861357 1.035121
O 1.247946 -1.485897 1.149829
C 0.083698 0.655711 1.328492
C 0.731342 1.421196 0.154753
O 2.132356 1.122205 0.044804
O -1.113507 1.249535 1.719915
C -2.006658 -1.294894 -0.613945
O -2.718326 -0.3616 0.173155
O -1.178646 1.45105 -1.203956
H -0.119794 -2.215663 -0.594093
H -0.744348 -1.296908 1.758742
H 0.765564 0.749415 2.184257
H 0.572034 2.491575 0.309875
H 0.728643 1.41921 -2.009851
H -1.542672 1.424975 -2.097848
H 2.647523 1.76325 0.547427
H -1.816832 1.032015 1.084402
H 1.509913 -1.532859 2.077606
H -2.304993 -2.313107 -0.337605
H -2.204259 -1.159412 -1.684445
H -3.636508 -0.635194 0.276705
Na 2.445604 -0.970174 -0.710486

Transition state of 3rd (7) reaction of βGlc
25

C -1.974816 -0.974312 -0.870959
O 0.866868 -0.907091 -0.576186
C 0.694525 0.396207 -0.586651
C 0.197534 1.063876 0.928163
O -0.340653 2.330865 0.737991
C -0.698247 0.146286 1.550152
C -2.856212 -0.085903 -0.4034
O -2.854944 1.191196 -0.925282
O -0.290537 -1.037069 1.856688
C 1.94219 1.220456 -0.918801
O 3.034154 0.690196 -0.149506
O -1.755815 -2.17057 -0.263667
H -0.137048 0.778932 -1.211496
H 1.160146 1.115454 1.448763
H -1.734172 0.416449 1.738353
H -3.537925 -0.297547 0.418624
H -1.371262 -0.749237 -1.743561
H -0.826549 -2.379937 -0.456082
H -3.747136 1.480354 -1.156755
H -1.024911 -1.639751 2.069777
H -1.072478 2.283905 0.099701
H 1.785541 2.278761 -0.689487
H 2.153026 1.10257 -1.988201
H 3.748006 1.336585 -0.109942
Na 2.863849 -1.555127 -0.186456

Reactant state of 3rd (7) reaction of βGlc
25

C 0.882379 1.103175 -0.954782
O -0.404673 0.516683 -1.015817
C -0.529935 -0.897076 -0.804797
C 0.036805 -1.336541 0.568865
O 0.607539 -2.613358 0.481385
C 1.007057 -0.285658 1.133748
C 1.817686 0.336119 -0.004389
O 2.40234 -0.759274 -0.688564
O 0.226548 0.718394 1.789681
C -2.01892 -1.162349 -0.899128
O -2.654201 -0.311202 0.067397
O 0.658281 2.404168 -0.466452
H -0.005792 -1.442068 -1.599063
H -0.781383 -1.420389 1.291186
H 1.669723 -0.787862 1.845047
H 2.57186 1.036075 0.372595
H 1.320281 1.128363 -1.961053
H 0.734066 3.049144 -1.179687
H 3.249761 -0.522366 -1.082252
H 0.764985 1.175486 2.447025
H 1.410425 -2.560853 -0.057353
H -2.204944 -2.216671 -0.67031
H -2.388752 -0.926724 -1.901453
H -3.549547 -0.624705 0.237961
Na -1.513434 1.629386 0.607202

Transition state of 1st (8) reaction of βGlc
25

C 1.086386 -1.202923 -0.859712
O 0.8655 0.025089 -1.245308
C -1.225869 1.11949 -0.820471
C -1.949034 0.701752 0.241837
O -2.907519 -0.296367 0.10893
C -0.477483 -0.921154 1.176164
C -0.059811 -1.809777 0.092252
O -1.121454 -2.367993 -0.605076
O 0.448591 -0.249847 1.80796
C -0.341986 2.328897 -0.801643
O 0.365055 2.426062 0.448625
O 2.300455 -1.263973 -0.021561
H -1.409356 0.674799 -1.794379
H -1.941192 1.236843 1.190735
H -1.456697 -1.050624 1.635322
H 0.468036 -2.619451 0.626005
H 1.181686 -1.953841 -1.663216
H 2.912475 -1.925861 -0.364054
H -1.824905 -1.722281 -0.767394
H 0.097245 0.217867 2.581754
H -3.789249 0.077023 -0.024797
H -0.964701 3.220456 -0.944104
H 0.369089 2.260659 -1.626423
H 0.372751 3.346533 0.7354
Na 2.198779 0.977854 0.208729

Reactant state of 1st (8) reaction of βGlc
25

C -0.854482 -1.134626 -0.946241
O 0.414958 -0.501823 -1.013886
C 0.508474 0.908822 -0.797513
C -0.073464 1.327476 0.571629
O -0.760682 2.54788 0.359278
C -1.034965 0.263838 1.117822
C -1.819602 -0.368286 -0.035585
O -2.420667 0.607738 -0.85232
O -0.244648 -0.715341 1.795273
C 1.992906 1.201066 -0.883119
O 2.636343 0.371872 0.09715
O -0.57233 -2.413512 -0.422132
H -0.03162 1.453808 -1.579349
H 0.734433 1.448052 1.303554
H -1.717278 0.750519 1.825599
H -2.565411 -1.080347 0.343855
H -1.28863 -1.201428 -1.949652
H -0.733737 -3.092357 -1.087745
H -2.51769 1.45405 -0.393458
H -0.821976 -1.301919 2.3012
H -0.686408 3.121491 1.130971
H 2.164853 2.260809 -0.666884
H 2.368747 0.961447 -1.882027
H 3.54937 0.657825 0.215538
Na 1.528033 -1.617837 0.597231

Transition state of 2nd (8) reaction of βGlc
25

C -1.086652 1.202762 -0.859676
O -0.865512 -0.02519 -1.245313
C 1.226139 -1.119222 -0.820499
C 1.949251 -0.701316 0.241781
O 2.907478 0.297039 0.108835
C 0.477262 0.921081 1.176169
C 0.059383 1.809773 0.092397
O 1.12089 2.368414 -0.604798
O -0.448658 0.249422 1.807824
C 0.342515 -2.328821 -0.80167
O -0.364476 -2.426173 0.448608
O -2.300785 1.263504 -0.021597
H 1.409472 -0.674468 -1.794407
H 1.941593 -1.236418 1.190675
H 1.456415 1.050772 1.635396
H -0.468685 2.619218 0.626278
H -1.182061 1.953703 -1.663146
H -2.91275 1.925601 -0.363788
H 1.824507 1.722924 -0.767272
H -0.097199 -0.218395 2.581504
H 3.78929 -0.07613 -0.024962
H 0.965425 -3.22024 -0.94416
H -0.368587 -2.26072 -1.626439
H -0.372116 -3.346684 0.73526
Na -2.198563 -0.978258 0.208688

Reactant state of 2nd (8) reaction of βGlc
25

C 0.813632 1.151387 -0.948972
O -0.438722 0.482764 -1.014827
C -0.489482 -0.927091 -0.798979
C 0.117995 -1.334131 0.562959
O 0.862296 -2.515288 0.313359
C 1.032028 -0.234619 1.121804
C 1.802828 0.43135 -0.020833
O 2.520296 -0.503565 -0.787975
O 0.194692 0.709995 1.791254
C -1.965046 -1.265874 -0.87316
O -2.624208 -0.45683 0.112285
O 0.486237 2.426478 -0.440548
H 0.055154 -1.456132 -1.590162
H -0.678588 -1.512922 1.29515
H 1.728503 -0.693115 1.834155
H 2.493304 1.189676 0.370192
H 1.250068 1.219817 -1.951247
H 0.635197 3.104766 -1.109645
H 2.37665 -1.411301 -0.481615
H 0.741086 1.298612 2.328069
H 0.924993 -3.059423 1.107966
H -2.102132 -2.33053 -0.656375
H -2.355203 -1.037499 -1.869241
H -3.52384 -0.776462 0.246451
Na -1.583786 1.580905 0.584636

Transition state of 3rd (8) reaction of βGlc
25

C 1.085969 -1.203296 -0.859703
O 0.865474 0.024794 -1.245261
C -1.225464 1.119927 -0.820439
C -1.94879 0.702418 0.241852
O -2.907647 -0.295337 0.108896
C -0.477853 -0.921013 1.176144
C -0.060471 -1.809781 0.092222
O -1.122305 -2.367596 -0.605139
O 0.448433 -0.250055 1.807994
C -0.341146 2.329017 -0.80158
O 0.365959 2.425888 0.448672
O 2.300015 -1.264611 -0.021589
H -1.409114 0.675334 -1.794361
H -1.94077 1.237482 1.190763
H -1.457131 -1.050155 1.635261
H 0.467029 -2.619674 0.625982
H 1.18107 -1.954219 -1.66323
H 2.911205 -1.92786 -0.362944
H -1.825512 -1.721622 -0.76747
H 0.097211 0.217802 2.581758
H -3.789232 0.078389 -0.024847
H -0.963546 3.220803 -0.943999
H 0.369884 2.260554 -1.62638
H 0.373952 3.34634 0.735499
Na 2.199278 0.977139 0.208582

Reactant state of 3rd (8) reaction of βGlc
25

C 0.812713 1.152008 -0.94885
O -0.439017 0.482296 -1.014682
C -0.488571 -0.927618 -0.798956
C 0.118958 -1.333984 0.563118
O 0.864117 -2.514762 0.31397
C 1.032292 -0.233847 1.121803
C 1.802533 0.432448 -0.021026
O 2.519691 -0.502226 -0.788804
O 0.194481 0.710428 1.791202
C -1.963947 -1.267305 -0.873257
O -2.623644 -0.458591 0.112117
O 0.484102 2.426716 -0.440268
H 0.056594 -1.45617 -1.590088
H -0.677581 -1.513117 1.295255
H 1.72901 -0.691881 1.834213
H 2.492899 1.190994 0.369797
H 1.249087 1.220933 -1.951128
H 0.633891 3.105404 -1.108766
H 2.37869 -1.409777 -0.480685
H 0.74065 1.299618 2.327625
H 0.9266 -3.058735 1.108703
H -2.100571 -2.332036 -0.65663
H -2.354136 -1.039014 -1.869347
H -3.521763 -0.781151 0.249283
Na -1.585008 1.580348 0.584227

Transition state of 1st (9) reaction of Manβ-Me
28

C 1.714203 -0.127283 0.522767
O 0.383665 -0.533299 -1.438431
C -0.844573 -1.206181 -1.168752
C -0.773455 -1.740794 0.269159
O 0.341447 -2.616247 0.377414
C -0.583985 -0.750263 1.428073
C 0.571414 0.350744 1.326488
O 0.133248 1.582462 1.09281
O -1.79722 -0.053 1.632173
C -2.056274 -0.343761 -1.516659
O -2.051645 0.909966 -0.854935
O 2.45025 0.713205 -0.071196
H -0.900718 -2.092203 -1.820306
H -1.711805 -2.274002 0.479244
H -0.380408 -1.386773 2.295634
H 1.110174 0.257437 2.321454
H 2.026505 -1.175011 0.564039
C 3.630705 0.249578 -0.776605
H 0.209169 0.254417 -1.9666
H -1.550996 0.792093 2.04928
H 0.165929 -3.452643 -0.069354
H -2.973274 -0.900277 -1.287615
H -2.053451 -0.140402 -2.5922
H -2.120847 0.685194 0.101751
H 3.992221 -0.676138 -0.327438
H 4.366679 1.044494 -0.685263
H 3.346476 0.094572 -1.817091
Na -0.831984 2.759299 -0.332278

Reactant state of 1st (9) reaction of Manβ-Me
28

C 1.316685 -0.331947 -0.368541
O 0.324909 0.659254 -0.673336
C -0.812399 0.079582 -1.313312
C -1.227203 -1.246573 -0.614425
O -0.642998 -2.347524 -1.291789
C -0.692115 -1.367847 0.81663
C 0.84357 -1.084543 0.879133
O 1.060717 -0.253752 2.015336
O -1.419572 -0.460607 1.632232
C -1.861562 1.172731 -1.412433
O -2.087182 1.738244 -0.122979
O 2.492048 0.309212 -0.05434
H -0.538583 -0.192458 -2.344108
H -2.319728 -1.327403 -0.575447
H -0.885233 -2.393676 1.139536
H 1.399426 -2.022093 0.977654
H 1.426649 -1.00189 -1.2341
C 3.201078 0.827763 -1.181704
H 2.015381 -0.106283 2.084356
H -1.377482 -0.772227 2.544996
H -1.208028 -2.657293 -2.008345
H -2.785701 0.745653 -1.81734
H -1.496759 1.945573 -2.097732
H -2.890536 2.27044 -0.13789
H 4.098748 1.303236 -0.790617
H 2.590501 1.563825 -1.71137
H 3.481703 0.013378 -1.857517
Na -0.358369 1.561601 1.359531

Transition state of 2nd (9) reaction of Manβ-Me
28

C 1.714233 -0.127366 0.522703
O 0.383587 -0.533293 -1.438447
C -0.844655 -1.206155 -1.168742
C -0.773511 -1.740776 0.269165
O 0.341359 -2.616275 0.37738
C -0.58396 -0.750251 1.428069
C 0.57147 0.350713 1.326442
O 0.133342 1.582444 1.092738
O -1.797168 -0.052951 1.63222
C -2.056354 -0.343718 -1.516616
O -2.051697 0.910003 -0.854878
O 2.450327 0.713094 -0.071226
H -0.900828 -2.092172 -1.8203
H -1.711875 -2.273948 0.479283
H -0.38037 -1.386768 2.295622
H 1.110239 0.257416 2.3214
H 2.026467 -1.175114 0.563937
C 3.630842 0.249594 -0.776588
H 0.209079 0.254473 -1.966537
H -1.550903 0.792132 2.04932
H 0.16579 -3.452664 -0.069383
H -2.973355 -0.900225 -1.287558
H -2.05355 -0.140349 -2.592154
H -2.120862 0.685222 0.101809
H 4.367665 1.043475 -0.683036
H 3.34726 0.097103 -1.817615
H 3.990918 -0.6775 -0.329106
Na -0.832006 2.759318 -0.332234

Reactant state of 2nd (9) reaction of Manβ-Me
28

C -1.307925 0.496147 -0.140717
O -0.353977 -0.239123 -0.915401
C 0.80408 0.53954 -1.229705
C 1.249633 1.385384 -0.011788
O 0.662149 2.663837 -0.178897
C 0.761093 0.804377 1.3219
C -0.779791 0.557557 1.297542
O -0.996993 -0.706121 1.917491
O 1.478243 -0.403516 1.559183
C 1.830113 -0.439557 -1.785032
O 2.105202 -1.513913 -0.891832
O -2.487682 -0.21225 -0.121378
H 0.554514 1.258243 -2.022983
H 2.345392 1.450714 0.010378
H 0.999082 1.523444 2.112321
H -1.300278 1.347476 1.848043
H -1.430089 1.497511 -0.577869
C -3.249267 -0.122175 -1.328978
H -1.954636 -0.844556 1.960329
H 1.446669 -0.599059 2.504785
H 1.152236 3.336296 0.309474
H 2.747479 0.092268 -2.058297
H 1.41804 -0.894786 -2.688199
H 2.745657 -1.226137 -0.227779
H -2.673289 -0.510365 -2.173027
H -3.537894 0.917095 -1.515242
H -4.140862 -0.727908 -1.177627
Na 0.229529 -2.059926 0.363912

Transition state of 3rd (9) reaction of Manβ-Me
28

C 1.714292 -0.127496 0.522597
O 0.383439 -0.533283 -1.438486
C -0.8448 -1.206119 -1.168721
C -0.773595 -1.74075 0.269177
O 0.341232 -2.616316 0.37732
C -0.583903 -0.750232 1.428065
C 0.571566 0.350672 1.326357
O 0.133495 1.582418 1.092602
O -1.797071 -0.052883 1.632308
C -2.056503 -0.343661 -1.516526
O -2.051789 0.910055 -0.854774
O 2.450456 0.712915 -0.071291
H -0.901024 -2.092129 -1.820283
H -1.711974 -2.273869 0.479362
H -0.380279 -1.38676 2.295602
H 1.110351 0.257401 2.321306
H 2.02642 -1.175274 0.563784
C 3.631079 0.249629 -0.776557
H 0.208899 0.25457 -1.966433
H -1.550755 0.792177 2.049417
H 0.165601 -3.45267 -0.069485
H -2.973502 -0.900153 -1.287425
H -2.053749 -0.14028 -2.592062
H -2.120864 0.685261 0.101918
H 3.988704 -0.679792 -0.331942
H 4.369338 1.041737 -0.679245
H 3.348616 0.10141 -1.818495
Na -0.832062 2.759352 -0.332164

Reactant state of 3rd (9) reaction of Manβ-Me
28

C -1.057724 0.775128 0.148316
O -0.561103 0.097979 -1.011883
C 0.803501 0.286392 -1.394889
C 1.566649 1.153795 -0.385808
O 1.129415 2.498043 -0.460704
C 1.323192 0.765582 1.076944
C -0.186507 0.478934 1.373059
O -0.39582 -0.883108 1.708162
O 2.065432 -0.399103 1.429383
C 1.381387 -1.108971 -1.659669
O 1.075129 -2.033967 -0.628332
O -2.31118 0.220904 0.367626
H 0.816844 0.830817 -2.350568
H 2.642586 1.07212 -0.594495
H 1.647657 1.615166 1.683561
H -0.536214 1.112784 2.194788
H -1.113077 1.853466 -0.037723
C -3.348557 0.810191 -0.425926
H 0.333584 -1.170117 2.275001
H 2.933673 -0.153416 1.771199
H 1.420019 2.910665 -1.283187
H 2.462315 -1.047241 -1.832635
H 0.922323 -1.507071 -2.567985
H 1.61142 -1.816933 0.151325
H -3.463433 1.86618 -0.16523
H -4.266174 0.276949 -0.182365
H -3.124698 0.708966 -1.491909
Na -1.199406 -1.956782 -0.108723

Transition state of 1st (10) reaction of Manβ-Me
28

C -1.322724 1.060023 0.667306
O 0.650386 1.094128 -0.942279
C 1.753504 0.637077 -0.170746
C 1.304277 -0.338499 0.934459
O 1.016793 0.332748 2.158218
C 0.039206 -1.127068 0.549122
C -1.363201 -0.279814 1.154846
O -2.43139 -1.060447 0.65761
O -0.123185 -1.391194 -0.731631
C 2.816324 0.017745 -1.072075
O 3.915516 -0.2786 -0.231087
O -2.067411 1.415302 -0.308312
H 2.193586 1.517385 0.311393
H 2.106377 -1.070629 1.08857
H -0.073767 -1.993782 1.227448
H -1.22098 -0.326978 2.234492
H -0.639106 1.776432 1.122902
C -1.876444 2.750806 -0.858485
H -3.238071 -0.86388 1.150385
H 0.270551 0.267085 -1.313554
H 1.83976 0.533606 2.619431
H 3.078293 0.745644 -1.848937
H 2.414134 -0.887992 -1.547588
H 4.657481 -0.603961 -0.751435
H -0.887255 2.7804 -1.314969
H -1.974186 3.48117 -0.054348
H -2.668447 2.878305 -1.591081
Na -1.968155 -2.313627 -1.216312

Reactant state of 1st (10) reaction of Manβ-Me
28

C -0.644004 1.1492 0.442516
O 0.32564 0.989617 -0.589513
C 1.555321 0.503805 -0.046388
C 1.31663 -0.797117 0.766587
O 1.367406 -0.5633 2.159996
C -0.103892 -1.305681 0.510745
C -1.116798 -0.236884 0.949961
O -2.38966 -0.586332 0.406587
O -0.289301 -1.639483 -0.869338
C 2.526361 0.308811 -1.198894
O 3.72734 -0.117423 -0.592738
O -1.738489 1.818668 -0.055141
H 1.97473 1.256043 0.637073
H 2.039698 -1.561303 0.46097
H -0.259673 -2.229115 1.076302
H -1.174945 -0.222972 2.041896
H -0.16883 1.707884 1.266062
C -1.509311 3.203751 -0.329574
H -2.902938 0.235108 0.339445
H -0.115229 -0.827218 -1.37557
H 2.286055 -0.496027 2.447851
H 2.64202 1.257405 -1.735307
H 2.14104 -0.447308 -1.899947
H 4.437672 -0.164279 -1.241594
H -0.742058 3.313592 -1.098794
H -1.205183 3.721411 0.586162
H -2.455282 3.608724 -0.684191
Na -2.424219 -2.30193 -1.006264

Transition state of 2nd (10) reaction of Manβ-Me
28

C -1.3226 1.060083 0.667172
O 0.650244 1.093946 -0.942338
C 1.753416 0.637015 -0.170809
C 1.304301 -0.338588 0.934421
O 1.016893 0.332611 2.158215
C 0.03921 -1.127114 0.549175
C -1.363096 -0.279774 1.154771
O -2.431302 -1.060402 0.657558
O -0.12312 -1.391317 -0.731613
C 2.816332 0.017827 -1.07211
O 3.915646 -0.278079 -0.231121
O -2.06756 1.415408 -0.308195
H 2.193356 1.517401 0.311324
H 2.1064 -1.070751 1.088386
H -0.073751 -1.993807 1.227522
H -1.221026 -0.326841 2.234453
H -0.63886 1.776461 1.122635
C -1.876743 2.750933 -0.858399
H -3.238138 -0.863383 1.149904
H 0.269876 0.266897 -1.31306
H 1.839901 0.533722 2.619248
H 3.078066 0.745663 -1.84911
H 2.414347 -0.888089 -1.547446
H 4.657584 -0.603551 -0.751439
H -0.8875 2.780697 -1.314754
H -1.974766 3.481298 -0.0543
H -2.668636 2.878254 -1.591151
Na -1.968017 -2.313963 -1.216143

Reactant state of 2nd (10) reaction of Manβ-Me
28

C -0.644034 1.149242 0.442578
O 0.325674 0.989688 -0.589484
C 1.555313 0.503814 -0.046337
C 1.316547 -0.797166 0.766473
O 1.367362 -0.563486 2.159903
C -0.103992 -1.30565 0.510611
C -1.116922 -0.236863 0.949832
O -2.389735 -0.586188 0.406215
O -0.289452 -1.639385 -0.869516
C 2.526417 0.308897 -1.1988
O 3.727273 -0.117738 -0.592586
O -1.738409 1.818826 -0.055036
H 1.974722 1.255952 0.637228
H 2.039546 -1.561368 0.46073
H -0.259771 -2.229111 1.076123
H -1.175298 -0.223042 2.041754
H -0.168761 1.707763 1.266171
C -1.509111 3.203895 -0.329506
H -2.90295 0.235297 0.339124
H -0.115133 -0.827112 -1.375658
H 2.286014 -0.496655 2.447846
H 2.64232 1.25758 -1.734981
H 2.141118 -0.44703 -1.90006
H 4.437955 -0.163409 -1.241153
H -0.741929 3.313627 -1.098807
H -1.204821 3.721505 0.586201
H -2.455088 3.608957 -0.683998
Na -2.424084 -2.302155 -1.005963

Transition state of 3rd (10) reaction of Manβ-Me
28

C -1.322558 1.060164 0.666619
O 0.649902 1.094201 -0.941897
C 1.753246 0.637248 -0.170602
C 1.304213 -0.338276 0.934694
O 1.016484 0.333141 2.158318
C 0.039257 -1.127016 0.54926
C -1.362983 -0.279794 1.154143
O -2.431314 -1.060199 0.656805
O -0.122597 -1.391532 -0.731588
C 2.815935 0.017941 -1.072094
O 3.915363 -0.278043 -0.231282
O -2.067581 1.415519 -0.308664
H 2.193355 1.51762 0.311389
H 2.106455 -1.070208 1.088965
H -0.073559 -1.993677 1.227705
H -1.221313 -0.326601 2.233897
H -0.639006 1.776651 1.122179
C -1.877 2.75122 -0.858536
H -3.237886 -0.863749 1.149798
H 0.270482 0.267208 -1.313705
H 1.839343 0.53402 2.61971
H 3.077594 0.745739 -1.849155
H 2.413786 -0.887963 -1.54731
H 4.657428 -0.602949 -0.75177
H -2.667322 2.87752 -1.593156
H -0.886752 2.782081 -1.312642
H -1.977859 3.481499 -0.05471
Na -1.967042 -2.315527 -1.215058

Reactant state of 3rd (10) reaction of Manβ-Me
28

C -0.644038 1.149239 0.442588
O 0.325679 0.989684 -0.589461
C 1.555319 0.503802 -0.046323
C 1.316565 -0.797195 0.766469
O 1.36741 -0.563572 2.159896
C -0.103973 -1.305651 0.510594
C -1.116883 -0.236862 0.949865
O -2.389678 -0.586222 0.406268
O -0.289421 -1.639322 -0.869527
C 2.526418 0.308898 -1.198793
O 3.72732 -0.117653 -0.592633
O -1.738441 1.818786 -0.055031
H 1.974703 1.255937 0.637265
H 2.039557 -1.561384 0.460662
H -0.259805 -2.229104 1.076108
H -1.1752 -0.223075 2.041794
H -0.168791 1.707809 1.266168
C -1.509178 3.20386 -0.32949
H -2.903027 0.235191 0.339401
H -0.115283 -0.826994 -1.375642
H 2.286063 -0.496463 2.447773
H 2.642234 1.257569 -1.735019
H 2.141106 -0.447069 -1.900009
H 4.43795 -0.16343 -1.241242
H -2.455153 3.6089 -0.684014
H -0.741978 3.313628 -1.098769
H -1.204932 3.721484 0.586225
Na -2.42416 -2.302104 -1.006024

Transition state of 1st (11) reaction of Manβ-Me
28

C 1.906931 -0.026255 0.414303
O -0.419685 -0.22239 -1.20544
C -0.440414 -0.791803 -0.014037
C -0.758115 0.1067 1.209155
O -0.166953 -0.530428 2.327135
C -0.141899 1.476116 0.981177
C 1.288684 1.357468 0.310903
O 1.288423 1.822405 -0.996901
O -1.024523 2.197873 0.141232
C -1.299717 -2.059934 0.022853
O -2.614272 -1.565833 -0.290646
O 2.554043 -0.405944 -0.623303
H 0.660451 -1.112818 0.327379
H -1.842833 0.217841 1.343586
H -0.037179 1.995956 1.940923
H 1.98302 1.991584 0.881362
H 2.132453 -0.465243 1.39093
C 3.197242 -1.697182 -0.595244
H 0.745795 1.162783 -1.486655
H -0.494034 2.797919 -0.407131
H -0.585742 -0.245465 3.147973
H -1.29157 -2.53397 1.00928
H -0.962995 -2.754821 -0.751551
H -3.257164 -2.281777 -0.234917
H 3.711172 -1.83845 0.35737
H 3.898771 -1.700843 -1.424913
H 2.42056 -2.452537 -0.742573
Na -2.412112 0.605792 -1.285667

Reactant state of 1st (11) reaction of Manβ-Me
28

C 1.316528 -0.343705 -0.356213
O 0.324663 0.635743 -0.696813
C -0.812621 0.033695 -1.316006
C -1.226734 -1.267676 -0.571914
O -0.641134 -2.391323 -1.209572
C -0.692784 -1.338803 0.862946
C 0.842601 -1.052553 0.916534
O 1.058821 -0.182765 2.023424
O -1.421341 -0.404265 1.645847
C -1.862164 1.122563 -1.452013
O -2.085974 1.732642 -0.182913
O 2.491158 0.308911 -0.063577
H -0.538962 -0.273094 -2.337061
H -2.319251 -1.34816 -0.531102
H -0.88546 -2.352972 1.22109
H 1.398845 -1.985853 1.047804
H 1.427873 -1.043602 -1.197532
C 3.203441 0.783708 -1.208078
H 2.013542 -0.033887 2.08849
H -1.380338 -0.683887 2.568955
H -1.201486 -2.721273 -1.920806
H -2.786726 0.68154 -1.840687
H -1.498376 1.870905 -2.164555
H -2.891478 2.260805 -0.213618
H 4.098966 1.275867 -0.833042
H 2.593589 1.497227 -1.768552
H 3.487688 -0.056319 -1.850117
Na -0.357961 1.608888 1.304365

Transition state of 2nd (11) reaction of Manβ-Me
28

C 1.907036 -0.026463 0.414203
O -0.419667 -0.222817 -1.204987
C -0.440585 -0.791637 -0.013327
C -0.757895 0.107468 1.209487
O -0.166768 -0.52938 2.327651
C -0.141364 1.476647 0.980914
C 1.289075 1.357394 0.31047
O 1.288732 1.821873 -0.997492
O -1.023947 2.198264 0.14086
C -1.30013 -2.059526 0.024015
O -2.614388 -1.565249 -0.290547
O 2.553598 -0.406639 -0.623617
H 0.66032 -1.11274 0.328186
H -1.842575 0.218963 1.343956
H -0.036366 1.996734 1.9405
H 1.983693 1.991576 0.880511
H 2.133383 -0.464818 1.390928
C 3.196674 -1.697908 -0.595374
H 0.745793 1.162292 -1.486927
H -0.493531 2.798422 -0.407427
H -0.585363 -0.243954 3.148426
H -1.292635 -2.532926 1.010748
H -0.963261 -2.755032 -0.749764
H -3.258091 -2.280196 -0.231535
H 3.709389 -1.839938 0.357785
H 3.89923 -1.701168 -1.424178
H 2.42007 -2.453054 -0.744149
Na -2.41249 0.604689 -1.286758

Reactant state of 2nd (11) reaction of Manβ-Me
28

C -1.316894 -0.341516 0.359233
O -0.325453 0.641492 0.690742
C 0.811966 0.045449 1.315529
C 1.22652 -1.262418 0.583113
O 0.641023 -2.380038 1.231504
C 0.692732 -1.346349 -0.8511
C -0.842859 -1.061167 -0.907355
O -1.059105 -0.200921 -2.02155
O 1.420997 -0.4184 -1.642145
C 1.861216 1.135665 1.442256
O 2.086477 1.734126 0.167724
O -2.492075 0.307643 0.060975
H 0.538251 -0.252673 2.339094
H 2.319031 -1.342886 0.5432
H 0.885782 -2.363449 -1.20056
H -1.398702 -1.995776 -1.030737
H -1.427648 -1.033869 1.206857
C -3.201721 0.796767 1.201066
H -2.013655 -0.050937 -2.086782
H 1.381577 -0.706867 -2.562575
H 1.205865 -2.709316 1.939449
H 2.785465 0.698296 1.835796
H 1.496756 1.890657 2.147359
H 2.890358 2.264976 0.195839
H -2.591143 1.51828 1.750364
H -3.48321 -0.035003 1.854991
H -4.098892 1.282828 0.822035
Na 0.359045 1.596903 -1.31898

Transition state of 3rd (11) reaction of Manβ-Me
28

C 1.907283 -0.026437 0.413905
O -0.420107 -0.222795 -1.204859
C -0.440763 -0.791491 -0.013071
C -0.757517 0.108049 1.209556
O -0.166635 -0.528849 2.32782
C -0.14054 1.477041 0.98068
C 1.289682 1.357457 0.309765
O 1.288945 1.82138 -0.998388
O -1.023149 2.198907 0.140922
C -1.300832 -2.059016 0.024534
O -2.61495 -1.564254 -0.289852
O 2.553603 -0.40744 -0.623683
H 0.660038 -1.113111 0.328128
H -1.842144 0.220064 1.344055
H -0.035051 1.997118 1.940226
H 1.984534 1.991752 0.879395
H 2.132976 -0.464827 1.390771
C 3.196096 -1.699011 -0.594987
H 0.745525 1.161732 -1.487272
H -0.49271 2.798411 -0.408055
H -0.58475 -0.242674 3.148583
H -1.293373 -2.532371 1.011282
H -0.964392 -2.754715 -0.749258
H -3.258838 -2.279114 -0.231782
H 3.897879 -1.703277 -1.42444
H 2.418981 -2.453887 -0.742392
H 3.709654 -1.840438 0.357812
Na -2.412766 0.603654 -1.28682

Reactant state of 3rd (11) reaction of Manβ-Me
28

C 1.246873 -0.415971 -0.373015
O 0.234237 0.516775 -0.770806
C -0.967437 -0.094512 -1.242016
C -1.245889 -1.415453 -0.485072
O -0.534221 -2.504013 -1.041118
C -0.740081 -1.295872 0.950164
C 0.798161 -1.063338 0.948141
O 1.070664 -0.172531 2.023603
O -1.424678 -0.185484 1.528379
C -2.123294 0.942004 -1.089575
O -1.711133 2.135767 -0.428683
O 2.393599 0.307063 -0.119734
H -0.833563 -0.348813 -2.302368
H -2.324841 -1.608653 -0.473286
H -0.976166 -2.211641 1.497084
H 1.321681 -2.013831 1.088846
H 1.394738 -1.160958 -1.164862
C 3.10807 0.700809 -1.293823
H 2.031837 -0.077605 2.086313
H -1.559339 -0.354778 2.468415
H -1.048573 -2.94085 -1.729085
H -2.927428 0.51186 -0.489038
H -2.527616 1.184931 -2.074572
H -1.675936 2.862382 -1.059087
H 3.444207 -0.183334 -1.84468
H 3.972117 1.269802 -0.95497
H 2.47801 1.324893 -1.933303
Na -0.132298 1.710907 1.169331

Transition state of 1st (12) reaction of Manβ-Me
28

C 1.165378 -0.283663 -1.039627
O 0.316481 0.676481 -1.595476
C -1.853076 0.591318 -0.8017
C -1.919731 -0.47235 0.249335
O -2.872844 -1.380075 -0.277168
C -0.648036 -1.288853 0.568366
C 0.389877 -1.510744 -0.536124
O 1.368143 -2.388562 -0.005048
O -0.023056 -0.749246 1.726009
C -1.912997 2.015235 -0.410629
O -0.680578 2.005609 0.235513
O 1.933633 0.284453 0.003021
H -1.875666 0.239162 -1.828871
H -2.241298 -0.026938 1.19915
H -1.055946 -2.278555 0.81428
H -0.117631 -1.940343 -1.410399
H 1.842975 -0.641132 -1.824996
C 3.219203 0.739909 -0.440904
H 1.185605 -3.302334 -0.253676
H 0.724624 -1.341586 1.911311
H -3.756076 -1.202284 0.068464
H -2.763404 2.222033 0.256808
H -1.95704 2.694628 -1.269415
H 0.159675 1.397885 -0.888894
H 3.702363 1.224501 0.408172
H 3.11156 1.455846 -1.261103
H 3.824307 -0.113682 -0.757187
Na 0.748909 1.388947 1.69175

Reactant state of 1st (12) reaction of Manβ-Me
28

C -0.910126 -0.055525 -1.145384
O 0.323338 -0.527012 -1.564896
C 1.554018 -0.18456 -0.909136
C 1.509417 0.853406 0.24145
O 2.522189 1.774691 -0.094546
C 0.162332 1.556178 0.489229
C -0.827569 1.389845 -0.660696
O -2.113284 1.775311 -0.20463
O -0.398656 1.021771 1.691146
C 2.142844 -1.507203 -0.43208
O 1.178365 -2.182836 0.381006
O -1.447139 -0.914295 -0.13613
H 2.199485 0.253117 -1.676209
H 1.765151 0.348721 1.187225
H 0.360875 2.62471 0.637736
H -0.488575 1.997638 -1.50824
H -1.570281 -0.114307 -2.018003
C -2.819864 -1.271871 -0.350262
H -2.398245 2.598435 -0.617492
H -1.28923 1.400471 1.768072
H 2.778551 2.296179 0.676535
H 3.034886 -1.341858 0.176178
H 2.409339 -2.136631 -1.285133
H 0.505433 -2.509208 -0.235664
H -3.096582 -1.972578 0.438736
H -2.932476 -1.766521 -1.319833
H -3.457163 -0.386617 -0.298108
Na -0.291453 -1.232201 1.827261

Transition state of 2nd (12) reaction of Manβ-Me
28

C 1.165799 -0.282913 -1.039242
O 0.316794 0.677684 -1.593951
C -1.853708 0.590622 -0.801622
C -1.919768 -0.473023 0.249401
O -2.872585 -1.380813 -0.277634
C -0.647628 -1.288887 0.568314
C 0.390386 -1.510278 -0.536211
O 1.368581 -2.388373 -0.005525
O -0.02292 -0.748911 1.725943
C -1.914746 2.014488 -0.411095
O -0.683108 2.005387 0.23666
O 1.934691 0.284237 0.003495
H -1.874743 0.238382 -1.828789
H -2.241577 -0.027812 1.19921
H -1.054949 -2.278827 0.814221
H -0.117141 -1.939459 -1.410688
H 1.842985 -0.639899 -1.825184
C 3.219915 0.740246 -0.440882
H 1.186464 -3.301911 -0.255301
H 0.724015 -1.341795 1.912426
H -3.755307 -1.205489 0.07054
H -2.766034 2.221549 0.255111
H -1.957694 2.69345 -1.270278
H 0.1586 1.39812 -0.886719
H 3.825113 -0.113078 -0.7577
H 3.703321 1.224714 0.408123
H 3.11166 1.456384 -1.260826
Na 0.749288 1.389132 1.69018

Reactant state of 2nd (12) reaction of Manβ-Me
28

C -0.910339 -0.055975 -1.145244
O 0.323238 -0.527162 -1.564696
C 1.553876 -0.183837 -0.909317
C 1.508859 0.853911 0.241435
O 2.521524 1.775421 -0.094225
C 0.161555 1.556307 0.489101
C -0.828258 1.38951 -0.660867
O -2.114185 1.774507 -0.205054
O -0.399306 1.02182 1.691028
C 2.143739 -1.506095 -0.432482
O 1.179943 -2.182405 0.38086
O -1.446949 -0.914619 -0.135659
H 2.198823 0.254358 -1.676531
H 1.76456 0.349115 1.187164
H 0.359769 2.624921 0.637504
H -0.48943 1.997229 -1.508534
H -1.57059 -0.115217 -2.017755
C -2.819426 -1.273128 -0.349778
H -2.398688 2.59835 -0.616807
H -1.29021 1.399821 1.767601
H 2.777591 2.296905 0.676962
H 3.035815 -1.340125 0.175552
H 2.410437 -2.135326 -1.285616
H 0.507043 -2.509202 -0.235636
H -3.457201 -0.388155 -0.298583
H -3.095972 -1.973221 0.439823
H -2.93157 -1.768729 -1.31892
Na -0.290411 -1.232308 1.827151

Transition state of 3rd (12) reaction of Manβ-Me
28

C 1.165425 -0.285309 -1.039413
O 0.317036 0.675286 -1.595402
C -1.852529 0.592711 -0.801896
C -1.920051 -0.471044 0.249028
O -2.873551 -1.378266 -0.277627
C -0.648981 -1.288395 0.568508
C 0.388889 -1.511596 -0.535739
O 1.366274 -2.390176 -0.004369
O -0.023671 -0.748973 1.726044
C -1.911032 2.016644 -0.410585
O -0.678826 2.00562 0.235832
O 1.934064 0.282395 0.003123
H -1.875567 0.240694 -1.829108
H -2.241548 -0.02542 1.198774
H -1.057778 -2.277665 0.814694
H -0.118878 -1.940907 -1.410006
H 1.842755 -0.643347 -1.824763
C 3.218298 0.740508 -0.441975
H 1.182339 -3.303944 -0.251995
H 0.723671 -1.341733 1.911396
H -3.756783 -1.199884 0.067697
H -2.761461 2.224146 0.256623
H -1.954197 2.696229 -1.269271
H 0.161228 1.39701 -0.889176
H 3.702021 1.224358 0.407206
H 3.108421 1.457622 -1.260854
H 3.824218 -0.111642 -0.760583
Na 0.750076 1.388752 1.691908

Reactant state of 3rd (12) reaction of Manβ-Me
28

C -0.910149 -0.05567 -1.145392
O 0.323363 -0.527081 -1.564875
C 1.554021 -0.184473 -0.909168
C 1.509332 0.853498 0.241417
O 2.522018 1.774883 -0.094587
C 0.162194 1.556156 0.489191
C -0.8277 1.38972 -0.660721
O -2.113383 1.775152 -0.204565
O -0.398752 1.021757 1.691145
C 2.143013 -1.507031 -0.432089
O 1.178655 -2.182677 0.381136
O -1.447103 -0.914478 -0.136156
H 2.19942 0.253268 -1.676262
H 1.765131 0.348848 1.187192
H 0.360644 2.624707 0.637663
H -0.488739 1.997543 -1.508256
H -1.570281 -0.114518 -2.018027
C -2.819874 -1.271951 -0.350137
H -2.39881 2.597777 -0.618093
H -1.289291 1.400521 1.768148
H 2.778355 2.296377 0.6765
H 3.035094 -1.341567 0.176077
H 2.409472 -2.136501 -1.28512
H 0.505748 -2.509256 -0.235456
H -3.096483 -1.972826 0.438749
H -2.932665 -1.766381 -1.3198
H -3.457132 -0.386685 -0.297684
Na -0.291261 -1.232114 1.82718

Transition state of 1st (13) reaction of Manβ-Me
28

C 0.208731 -0.516319 -1.510915
O -0.741628 0.349437 -1.988626
C -1.152878 1.504531 0.172926
C -1.838792 0.231623 0.349566
O -2.678554 -0.072732 -0.628001
C -1.079841 -1.038664 0.762779
C -0.311215 -1.562983 -0.491334
O 0.788109 -2.320944 0.008127
O -0.239878 -0.890774 1.909283
C -0.049035 2.213549 0.858776
O 0.812553 1.499745 1.702939
O 1.253241 0.219198 -0.870106
H -1.740493 2.164945 -0.470796
H -2.33512 0.719773 1.284047
H -1.863193 -1.763466 0.991499
H -1.010904 -2.201913 -1.038404
H 0.67237 -1.068428 -2.348138
C 1.925647 1.103388 -1.769946
H 1.025355 -3.018589 -0.614825
H -0.388908 -1.647035 2.489771
H -1.729559 0.110764 -1.608553
H -0.49607 3.080023 1.375288
H 0.55097 2.649062 0.04791
H 0.308752 1.164518 2.457879
H 1.211962 1.787777 -2.235492
H 2.436948 0.529472 -2.550263
H 2.661672 1.660998 -1.187651
Na 1.902706 -0.550008 1.100698

Reactant state of 1st (13) reaction of Manβ-Me
28

C -0.56862 -0.318758 -1.295404
O 0.247476 -1.426186 -1.046222
C 0.975098 -1.443502 0.190648
C 1.945835 -0.259393 0.188581
O 2.901369 -0.442786 -0.835452
C 1.27481 1.103772 -0.063683
C 0.208732 1.018272 -1.182715
O -0.67429 2.112836 -0.992803
O 0.737652 1.667811 1.138834
C 0.125462 -1.664806 1.447373
O -0.427597 -0.501325 2.054999
O -1.697414 -0.295878 -0.459432
H 1.585933 -2.348301 0.096454
H 2.488238 -0.204719 1.140395
H 2.070714 1.760988 -0.422943
H 0.754305 1.11785 -2.128123
H -0.904424 -0.45458 -2.332675
C -2.626234 -1.348362 -0.740597
H -1.108181 2.348443 -1.822056
H 1.07036 2.568815 1.222113
H 2.52879 -0.991783 -1.538733
H 0.746294 -2.189838 2.181538
H -0.709357 -2.320542 1.191265
H 0.265927 -0.065538 2.566986
H -2.123341 -2.318326 -0.725081
H -3.086132 -1.186637 -1.72076
H -3.391558 -1.316362 0.034273
Na -1.535967 1.287402 1.101156

Transition state of 2nd (13) reaction of Manβ-Me
28

C 0.75913 -0.462802 -1.277823
O -0.451719 -0.781079 -1.862743
C -2.018358 -0.114695 -0.145514
C -1.347114 -1.237194 0.498166
O -1.388708 -2.339452 -0.23049
C 0.055825 -1.101299 1.144288
C 1.064858 -1.293457 -0.019899
O 2.373776 -0.915805 0.407556
O 0.351552 0.07445 1.877047
C -2.22477 1.320487 0.12406
O -1.471326 1.858835 1.184558
O 0.764388 0.914201 -0.88147
H -2.715532 -0.487087 -0.899995
H -2.184833 -1.15716 1.310956
H 0.153625 -1.956927 1.818874
H 1.04234 -2.345087 -0.312343
H 1.577519 -0.602197 -2.001602
C 0.654068 1.792476 -2.004611
H 2.868163 -1.684906 0.712359
H -0.408952 0.680393 1.882269
H -0.85515 -1.64245 -1.385745
H -3.303307 1.47006 0.275091
H -1.979664 1.808469 -0.835852
H -1.963739 2.56289 1.6239
H -0.230224 1.54266 -2.596529
H 1.543266 1.713177 -2.63978
H 0.569386 2.808322 -1.614841
Na 2.07287 1.216322 0.981416

Reactant state of 2nd (13) reaction of Manβ-Me
28

C 0.505412 0.080385 -1.279129
O -0.787038 0.582144 -1.43887
C -1.768212 0.462034 -0.406216
C -1.713228 -0.88252 0.350533
O -2.256523 -1.917571 -0.444645
C -0.285491 -1.367076 0.635842
C 0.495487 -1.324632 -0.678782
O 1.85118 -1.725324 -0.471174
O 0.369084 -0.636754 1.662219
C -1.852629 1.705009 0.468082
O -0.756331 1.780148 1.378326
O 1.323725 0.903837 -0.467699
H -2.713922 0.457036 -0.96222
H -2.250692 -0.781682 1.304413
H -0.355764 -2.404952 0.974858
H -0.001439 -1.981521 -1.398522
H 0.923942 0.065218 -2.293225
C 1.451489 2.232386 -0.975379
H 1.970621 -2.650401 -0.713097
H -0.006616 0.269359 1.700386
H -3.213626 -1.829239 -0.521343
H -2.799339 1.665428 1.020373
H -1.861101 2.584929 -0.184555
H -0.92596 2.459919 2.041508
H 0.489251 2.747016 -0.940969
H 1.822343 2.209763 -2.005807
H 2.16672 2.753135 -0.338834
Na 2.527803 -0.262669 1.080188

Transition state of 3rd (13) reaction of Manβ-Me
28

C 0.556819 -0.281828 -1.366628
O -0.723497 -0.355784 -1.864066
C -2.137152 0.086627 0.105132
C -1.479182 -1.167967 0.446598
O -1.679802 -2.114167 -0.461588
C -0.01482 -1.278499 0.950077
C 0.864737 -1.364682 -0.318619
O 2.243789 -1.238634 0.02885
O 0.503677 -0.242044 1.777865
C -2.093453 1.490418 0.544765
O -1.041938 1.906375 1.348762
O 0.782111 0.995051 -0.726477
H -2.992573 -0.129661 -0.541653
H -2.219573 -1.192454 1.351746
H 0.050017 -2.23533 1.478232
H 0.670094 -2.331403 -0.785084
H 1.293218 -0.357498 -2.181687
C 0.68115 2.079655 -1.663758
H 2.673344 -2.101093 0.045845
H -0.056997 -0.108425 2.553464
H -1.18789 -1.251572 -1.503407
H -3.043832 1.716882 1.050583
H -2.153434 2.047526 -0.412137
H -0.235876 1.609413 0.900169
H 0.740239 3.008408 -1.09411
H -0.271381 2.025683 -2.195067
H 1.503622 2.030264 -2.384582
Na 2.389791 0.752776 0.97323

Reactant state of 3rd (13) reaction of Manβ-Me
28

C 0.5026 0.130944 -1.229221
O -0.700771 0.82058 -1.351177
C -1.736191 0.721736 -0.359446
C -1.867106 -0.684416 0.258499
O -2.477967 -1.574573 -0.654561
C -0.517634 -1.352028 0.554244
C 0.309132 -1.300175 -0.727891
O 1.602626 -1.871146 -0.523784
O 0.18459 -0.778323 1.646787
C -1.651077 1.86563 0.643918
O -0.519787 1.784075 1.504749
O 1.434566 0.809068 -0.381791
H -2.655054 0.893973 -0.933271
H -2.440102 -0.610975 1.193796
H -0.718351 -2.397547 0.807182
H -0.236314 -1.837325 -1.509322
H 0.928282 0.115406 -2.239306
C 1.981179 1.973445 -1.019985
H 1.626687 -2.777993 -0.849419
H -0.135809 0.140439 1.801223
H -3.418621 -1.383251 -0.746686
H -2.53326 1.849018 1.28954
H -1.643163 2.815546 0.100081
H 0.263657 2.013689 0.986473
H 2.612795 2.480008 -0.289154
H 1.176889 2.641907 -1.34125
H 2.583111 1.681811 -1.885984
Na 2.388889 -0.661178 1.17942

Transition state of 1st RO reaction of Manβ(1→2)Manβ
46

C 1.543185 -0.581639 1.259701
O 2.425785 0.4218 1.624647
C 3.359168 0.997312 0.691065
C 3.821017 0.075798 -0.457854
O 4.994596 -0.558002 0.027731
C 2.828091 -1.012352 -0.876111
C 2.204778 -1.629549 0.370711
O 1.193349 -2.55934 0.022244
O 1.857799 -0.47906 -1.751537
C 2.86283 2.391335 0.300757
O 1.520056 2.414127 -0.15468
O -1.655107 1.565204 -0.074842
H 4.26798 1.151499 1.281614
H 4.042094 0.69818 -1.335044
H 3.399008 -1.792939 -1.402318
H 3.001673 -2.095426 0.962904
H 1.576273 -3.432154 -0.125131
H 1.117815 -1.112424 -1.821422
H 5.5012 -0.928027 -0.705167
H 3.485275 2.801703 -0.498194
H 2.95811 3.045251 1.174085
H 0.966737 2.161684 0.599984
C -1.277366 -1.364229 -0.421121
O -2.820155 -1.181292 -0.710789
C -3.839578 -0.614777 0.140499
C -3.254619 0.253605 1.271507
O -3.317956 -0.537599 2.441472
C -1.802515 0.676424 1.022602
C -0.888237 -0.536252 0.82337
O 0.405477 0.00753 0.624484
C -4.80278 0.126241 -0.764223
O -4.091815 1.221676 -1.328765
O -0.918643 -0.855151 -1.605634
H -4.360129 -1.454933 0.608213
H -3.870878 1.158002 1.373813
H -0.919997 -1.16547 1.720683
H -1.167616 -2.442148 -0.293429
H -2.302315 -0.694384 -1.580905
H -2.507653 1.696419 -0.52707
H -3.281435 0.016676 3.230342
H -5.651483 0.470359 -0.162157
H -5.164111 -0.546825 -1.548531
H -4.6591 1.736993 -1.914309
H -1.450102 1.196058 1.924112
H 1.205401 -1.049138 2.192319
Na 0.231121 1.110036 -1.475861

Reactant state of 1st RO reaction of Manβ(1→2)Manβ
46

C 1.483518 -0.404521 1.274162
O 2.320468 0.668638 1.542912
C 3.322429 1.113352 0.610199
C 3.869268 0.042014 -0.356285
O 5.009818 -0.496021 0.295035
C 2.915868 -1.111068 -0.673909
C 2.215339 -1.561344 0.603981
O 1.228172 -2.539858 0.313596
O 1.982546 -0.727632 -1.666019
C 2.851946 2.430944 -0.014217
O 1.523282 2.38047 -0.509429
O -1.720666 1.503774 -0.478202
H 4.184992 1.361031 1.237034
H 4.147508 0.532451 -1.298511
H 3.52252 -1.946279 -1.055441
H 2.969521 -1.932076 1.3076
H 1.599178 -3.424815 0.406863
H 1.254516 -1.373832 -1.620741
H 5.571642 -0.955971 -0.340194
H 3.500705 2.709379 -0.848095
H 2.921134 3.216447 0.745404
H 0.949504 2.258596 0.26177
C -1.472023 -1.383014 -0.299367
O -2.804048 -1.104076 -0.660581
C -3.755687 -0.651873 0.297203
C -3.208884 0.470244 1.210193
O -3.181498 -0.042527 2.529445
C -1.789516 0.858835 0.788501
C -0.924847 -0.395375 0.764978
O 0.391064 0.023668 0.460989
C -4.91684 -0.144968 -0.537229
O -4.43713 1.006113 -1.226116
O -0.749845 -1.224016 -1.506647
H -4.08355 -1.479757 0.938831
H -3.858501 1.35108 1.142751
H -0.963604 -0.876074 1.748903
H -1.381827 -2.42142 0.04094
H -1.263354 -1.680351 -2.188719
H -2.580664 1.422881 -0.932575
H -3.236906 0.674337 3.171774
H -5.752472 0.112209 0.124438
H -5.230978 -0.920148 -1.243137
H -5.128953 1.394407 -1.772535
H -1.375619 1.545654 1.538077
H 1.082265 -0.726616 2.241652
Na 0.282929 0.908295 -1.670474

Transition state of 2nd RO reaction of Manβ(1→2)Manβ
46

C 1.543199 -0.582021 1.259345
O 2.425789 0.42131 1.624671
C 3.359044 0.997242 0.691245
C 3.821318 0.075814 -0.457554
O 4.994313 -0.558631 0.028635
C 2.828259 -1.011924 -0.876539
C 2.204807 -1.629605 0.369966
O 1.193384 -2.559219 0.021005
O 1.858085 -0.478067 -1.751761
C 2.862634 2.391351 0.301136
O 1.519736 2.414648 -0.153855
O -1.655312 1.56501 -0.074037
H 4.267764 1.151575 1.281902
H 4.043223 0.6983 -1.334454
H 3.399102 -1.792331 -1.403089
H 3.001632 -2.095763 0.962028
H 1.57634 -3.431937 -0.126856
H 1.118104 -1.111381 -1.822129
H 5.501806 -0.927718 -0.704115
H 3.484903 2.801742 -0.497945
H 2.958338 3.045133 1.174518
H 0.966565 2.16232 0.600964
C -1.27724 -1.363985 -0.42213
O -2.819893 -1.180657 -0.711795
C -3.839403 -0.615149 0.140056
C -3.254585 0.252515 1.271673
O -3.317803 -0.539494 2.4411
C -1.802548 0.675662 1.022982
C -0.888133 -0.536764 0.822922
O 0.405488 0.00735 0.624369
C -4.802919 0.126374 -0.763911
O -4.092463 1.222794 -1.327189
O -0.918348 -0.854172 -1.606307
H -4.359624 -1.455863 0.607133
H -3.870937 1.156773 1.374626
H -0.919744 -1.166586 1.719814
H -1.167546 -2.441982 -0.295082
H -2.301991 -0.692994 -1.581426
H -2.508098 1.697145 -0.52554
H -3.281608 0.014262 3.230348
H -5.651821 0.46941 -0.161507
H -5.163884 -0.546001 -1.54898
H -4.65998 1.738501 -1.912165
H -1.450157 1.194799 1.924791
H 1.205455 -1.049859 2.191811
Na 0.231068 1.110765 -1.475316

Reactant state of 2nd RO reaction of Manβ(1→2)Manβ
46

C 1.483519 -0.404522 1.274154
O 2.320473 0.668636 1.542903
C 3.322437 1.113345 0.610193
C 3.869272 0.042006 -0.356293
O 5.009814 -0.496042 0.29503
C 2.915863 -1.111066 -0.673922
C 2.215344 -1.561344 0.603973
O 1.22819 -2.539875 0.313601
O 1.982534 -0.727611 -1.666017
C 2.851955 2.430938 -0.014223
O 1.523281 2.380471 -0.509407
O -1.720665 1.503799 -0.478187
H 4.185001 1.361023 1.237027
H 4.14752 0.532443 -1.298516
H 3.522504 -1.946279 -1.055464
H 2.969535 -1.932064 1.307589
H 1.599204 -3.424825 0.40689
H 1.254503 -1.373809 -1.620743
H 5.571643 -0.955985 -0.3402
H 3.5007 2.709365 -0.848114
H 2.921162 3.216443 0.745393
H 0.949524 2.258587 0.261806
C -1.472017 -1.383022 -0.299362
O -2.80404 -1.1041 -0.660579
C -3.755688 -0.651889 0.297194
C -3.208897 0.470238 1.210178
O -3.181536 -0.042512 2.529438
C -1.789525 0.858832 0.788505
C -0.924844 -0.395373 0.764979
O 0.391062 0.023681 0.460994
C -4.916827 -0.144988 -0.537254
O -4.437096 1.006099 -1.226116
O -0.749841 -1.224008 -1.506638
H -4.083557 -1.47977 0.938824
H -3.858513 1.351074 1.142713
H -0.963599 -0.876069 1.748906
H -1.381806 -2.421428 0.040942
H -1.263348 -1.680332 -2.188717
H -2.580658 1.422934 -0.932572
H -3.236985 0.67436 3.171756
H -5.752472 0.112185 0.124397
H -5.230948 -0.920165 -1.243174
H -5.128902 1.394394 -1.772555
H -1.375642 1.545645 1.538094
H 1.082272 -0.72662 2.241646
Na 0.2829 0.908331 -1.67044

Transition state of 3rd RO reaction of Manβ(1→2)Manβ
46

C -1.543247 0.582408 1.258945
O -2.425772 -0.420914 1.624555
C -3.359506 -0.996789 0.691534
C -3.821582 -0.075723 -0.457616
O -4.994315 0.559318 0.028414
C -2.828179 1.011426 -0.877156
C -2.204771 1.629646 0.369096
O -1.193262 2.559047 0.019833
O -1.858109 0.476619 -1.751897
C -2.86346 -2.391166 0.301836
O -1.520012 -2.414649 -0.151472
O 1.655715 -1.564508 -0.073205
H -4.268177 -1.150501 1.282426
H -4.043776 -0.698502 -1.334233
H -3.398681 1.791715 -1.404255
H -3.001633 2.0961 0.960883
H -1.576144 3.431734 -0.128392
H -1.117899 1.109632 -1.822721
H -5.501655 0.928404 -0.704444
H -3.484918 -2.801154 -0.49807
H -2.960432 -3.045112 1.174959
H -0.967816 -2.161254 0.603697
C 1.277188 1.363585 -0.423351
O 2.819615 1.179594 -0.71325
C 3.839377 0.615412 0.139193
C 3.254712 -0.250968 1.271847
O 3.31756 0.542447 2.440344
C 1.802786 -0.674697 1.023459
C 0.888128 0.537372 0.822448
O -0.405404 -0.007119 0.624324
C 4.803298 -0.126854 -0.76375
O 4.093846 -1.224874 -1.325121
O 0.917871 0.852998 -1.607065
H 4.359283 1.456889 0.60525
H 3.871312 -1.15492 1.375981
H 0.919625 1.167983 1.718782
H 1.167771 2.441702 -0.297061
H 2.301564 0.691237 -1.582368
H 2.508901 -1.698398 -0.523369
H 3.281529 -0.010379 3.23025
H 5.652616 -0.468086 -0.1609
H 5.163556 0.544573 -1.54996
H 4.661823 -1.740941 -1.909329
H 1.450535 -1.193374 1.925596
H -1.205615 1.050605 2.191271
Na -0.2311 -1.112139 -1.474235

Reactant state of 3rd RO reaction of Manβ(1→2)Manβ
46

C 1.483523 -0.404525 1.274143
O 2.320484 0.668626 1.542901
C 3.322461 1.113332 0.6102
C 3.869319 0.041974 -0.356245
O 5.009794 -0.496126 0.29515
C 2.91588 -1.111057 -0.673931
C 2.21532 -1.561351 0.603937
O 1.228119 -2.539815 0.313503
O 1.982579 -0.727542 -1.666029
C 2.851989 2.43092 -0.014249
O 1.523295 2.380476 -0.509371
O -1.720691 1.503778 -0.478193
H 4.185011 1.361017 1.237051
H 4.147648 0.532404 -1.298447
H 3.522503 -1.946276 -1.055491
H 2.969477 -1.932133 1.307557
H 1.599097 -3.424788 0.406724
H 1.254544 -1.373741 -1.62081
H 5.571666 -0.956055 -0.34005
H 3.500703 2.709288 -0.848184
H 2.921259 3.216459 0.745327
H 0.949572 2.258533 0.261859
C -1.472017 -1.382983 -0.299406
O -2.804049 -1.104033 -0.660607
C -3.755674 -0.65189 0.297217
C -3.208874 0.470226 1.210216
O -3.181469 -0.042569 2.529457
C -1.789516 0.858832 0.788507
C -0.924849 -0.395375 0.764961
O 0.391064 0.023674 0.460979
C -4.916872 -0.145008 -0.537168
O -4.43722 1.006091 -1.226065
O -0.749835 -1.22396 -1.506691
H -4.08349 -1.4798 0.938837
H -3.858499 1.351058 1.14279
H -0.963607 -0.876096 1.748875
H -1.381828 -2.421398 0.04087
H -1.263354 -1.680277 -2.188778
H -2.580676 1.422817 -0.932577
H -3.236851 0.674286 3.1718
H -5.752483 0.112141 0.124535
H -5.231018 -0.920191 -1.243071
H -5.129081 1.394385 -1.772436
H -1.375605 1.545647 1.538078
H 1.082274 -0.726627 2.241632
Na 0.282913 0.908361 -1.670496

Transition state of 1st c2 reaction of Manβ(1→2)Manβ
46

C -0.954929 1.324354 -0.729505
O -1.086593 0.79033 0.57674
C -2.440666 0.810616 1.042607
C -3.347485 -0.008742 0.115631
O -4.664413 0.233935 0.542301
C -3.151647 0.370911 -1.350769
C -1.662389 0.390271 -1.698877
O -1.111452 -0.90846 -1.506746
O -3.811504 -0.630517 -2.108128
C -2.425345 0.251208 2.450269
O -1.879907 -1.07038 2.381055
O 3.584627 1.334923 0.88746
H -2.808903 1.846953 1.07677
H -3.094788 -1.073098 0.220712
H -3.596057 1.360835 -1.522847
H -1.495865 0.729711 -2.729006
H -1.654358 -1.520894 -2.025953
H -4.175236 -0.265398 -2.923418
H -5.265231 -0.341805 0.051835
H -3.452163 0.230774 2.826174
H -1.801716 0.882374 3.09141
H -2.068912 -1.543797 3.198786
C 1.958781 3.092306 -0.483999
O 3.336608 -1.687557 -1.613058
C 3.199442 -1.674916 -0.201012
C 2.266079 -0.490025 0.086019
O 1.740226 -0.571021 1.397197
C 2.952385 0.844977 -0.115496
C 1.256672 1.938249 -0.151718
O 0.391722 1.378185 -1.081607
C 2.531411 -2.961904 0.277841
O 1.179051 -3.019982 -0.182565
O 2.874576 3.549141 0.296815
H 4.165307 -1.542377 0.30521
H 1.447182 -0.569765 -0.63718
H 1.04917 1.700776 0.88629
H 1.859344 3.545202 -1.474737
H 4.170377 -2.097142 -1.870641
H 3.325568 2.681689 0.782947
H 2.325995 -0.048512 1.97289
H 2.491509 -2.995649 1.3671
H 3.092057 -3.831286 -0.083044
H 1.197694 -3.077091 -1.148165
H 3.344152 0.987733 -1.132709
H -1.40325 2.332519 -0.747873
Na -0.288115 -1.445851 0.759436

Reactant state of 1st c2 reaction of Manβ(1→2)Manβ
46

C -1.040567 0.533659 -0.997667
O -0.955896 1.440149 0.056874
C -1.981898 1.54898 1.056795
C -3.405832 1.225414 0.565902
O -3.880448 2.428463 -0.014875
C -3.49477 0.125277 -0.498596
C -2.449669 0.380764 -1.573868
O -2.471799 -0.578511 -2.592104
O -3.329856 -1.187819 0.058672
C -1.521093 0.875155 2.353454
O -1.027327 -0.439801 2.184458
O 1.565283 -0.301341 1.233452
H -2.029168 2.621012 1.276476
H -4.011023 0.940365 1.437684
H -4.479266 0.175085 -0.977581
H -2.703252 1.343018 -2.028655
H -2.262526 -1.436724 -2.200348
H -4.200261 -1.591032 0.152539
H -4.841527 2.412647 -0.09866
H -2.350837 0.839528 3.06559
H -0.733458 1.497113 2.792331
H -0.104435 -0.354256 1.872064
C 1.07527 -2.472604 -0.697458
O 4.412181 0.622601 1.376442
C 4.159267 0.792675 -0.004122
C 3.218939 -0.306955 -0.532092
O 3.599677 -1.564784 0.009645
C 1.748524 -0.092896 -0.161186
C 0.80065 -1.006931 -0.97387
O -0.557992 -0.766068 -0.647844
C 3.647818 2.196451 -0.307902
O 3.160774 2.189467 -1.637814
O 0.346029 -3.133626 0.014833
H 5.127503 0.645336 -0.494441
H 3.310818 -0.335468 -1.623423
H 0.973651 -0.814566 -2.044258
H 1.974229 -2.912257 -1.151927
H 3.618023 0.851828 1.879966
H 2.111795 -1.067786 1.47944
H 4.326846 -1.427936 0.636706
H 4.462743 2.913255 -0.167897
H 2.844859 2.453026 0.402879
H 3.139721 3.085045 -1.991626
H 1.45029 0.937146 -0.370026
H -0.386862 0.933641 -1.781323
Na -1.514791 -2.197437 0.929476

Transition state of 2nd c2 reaction of Manβ(1→2)Manβ
46

C -0.954847 1.324284 -0.729707
O -1.086527 0.790529 0.576642
C -2.440629 0.810842 1.042455
C -3.34743 -0.008655 0.115577
O -4.664365 0.234152 0.542147
C -3.151516 0.370698 -1.350888
C -1.662239 0.389978 -1.698914
O -1.111307 -0.908707 -1.506461
O -3.811347 -0.630889 -2.108061
C -2.42538 0.251617 2.450189
O -1.879996 -1.070006 2.381201
O 3.584649 1.334918 0.887286
H -2.808885 1.847177 1.076466
H -3.094779 -1.073002 0.220868
H -3.595912 1.360588 -1.523201
H -1.495649 0.729194 -2.729106
H -1.654224 -1.52127 -2.025506
H -4.174905 -0.265975 -2.92352
H -5.265183 -0.341675 0.051782
H -3.452224 0.23125 2.826031
H -1.801777 0.882857 3.091283
H -2.068792 -1.543124 3.199156
C 1.958805 3.092245 -0.484161
O 3.336032 -1.687784 -1.613146
C 3.19928 -1.674981 -0.201056
C 2.265998 -0.490049 0.086077
O 1.740349 -0.570922 1.397344
C 2.952274 0.844954 -0.115602
C 1.256731 1.938159 -0.15184
O 0.391823 1.378075 -1.08177
C 2.53134 -2.961897 0.278104
O 1.178942 -3.020058 -0.18218
O 2.874599 3.549127 0.2966
H 4.165302 -1.542399 0.304853
H 1.447004 -0.569862 -0.636992
H 1.04915 1.700754 0.886165
H 1.859298 3.545137 -1.474895
H 4.169646 -2.097562 -1.870927
H 3.325623 2.681706 0.782765
H 2.326253 -0.048389 1.972883
H 2.49153 -2.995433 1.367372
H 3.091962 -3.831344 -0.082665
H 1.19753 -3.076967 -1.147796
H 3.343984 0.987614 -1.13285
H -1.403194 2.332431 -0.748311
Na -0.288081 -1.44567 0.75981

Reactant state of 2nd c2 reaction of Manβ(1→2)Manβ
46

C -1.802943 -0.163003 -1.476897
O -1.146729 1.055075 -1.638944
C -1.523121 2.135645 -0.761754
C -1.861186 1.561188 0.615043
O -2.097681 2.561111 1.577939
C -3.120941 0.711291 0.516456
C -3.192264 -0.009868 -0.85716
O -3.730732 -1.316618 -0.750208
O -3.152816 -0.264045 1.557253
C -0.373361 3.130708 -0.69585
O 0.553459 2.721615 0.295037
O 0.77855 -0.737408 1.37771
H -2.397858 2.647466 -1.190616
H -1.035979 0.91285 0.930198
H -3.991308 1.372792 0.621018
H -3.787974 0.597875 -1.54755
H -4.688266 -1.269798 -0.638047
H -3.097501 0.214066 2.396491
H -1.240658 2.915885 1.855955
H -0.777802 4.115086 -0.43454
H 0.086447 3.197274 -1.687514
H 1.46371 2.744343 -0.037427
C 0.417023 -2.825879 -0.556424
O 3.133685 1.756499 -0.396086
C 3.306804 0.627533 0.451877
C 2.604831 -0.582105 -0.203402
O 3.010162 -1.821875 0.350087
C 1.10657 -0.473398 0.027845
C 0.22466 -1.357635 -0.883782
O -1.122648 -1.040112 -0.595681
C 4.774761 0.350442 0.715744
O 5.291056 -0.208715 -0.489906
O -0.38366 -3.435367 0.122659
H 2.80985 0.877788 1.395357
H 2.820381 -0.550401 -1.280597
H 0.463033 -1.168067 -1.939796
H 1.336288 -3.309822 -0.919027
H 3.742558 1.679423 -1.14447
H 1.378073 -1.423843 1.708715
H 3.932648 -1.980598 0.102839
H 4.882374 -0.35012 1.552191
H 5.275682 1.289891 0.972554
H 6.254789 -0.211227 -0.478149
H 0.806408 0.556014 -0.181037
H -1.858742 -0.627457 -2.468579
Na -2.254757 -2.274564 0.88936

Transition state of 3rd c2 reaction of Manβ(1→2)Manβ
46

C -0.954952 1.324306 -0.729327
O -1.086688 0.7902 0.576881
C -2.440805 0.81052 1.042632
C -3.34766 -0.008626 0.115505
O -4.664581 0.234229 0.542103
C -3.151653 0.371038 -1.350863
C -1.662352 0.390275 -1.698799
O -1.111529 -0.908496 -1.506632
O -3.811555 -0.63036 -2.108229
C -2.425617 0.250856 2.450198
O -1.88041 -1.07082 2.380752
O 3.58463 1.334949 0.887799
H -2.808938 1.846889 1.076938
H -3.095127 -1.073024 0.220546
H -3.595985 1.360987 -1.523
H -1.495654 0.729736 -2.728895
H -1.654711 -1.520936 -2.02555
H -4.174574 -0.265382 -2.923903
H -5.265473 -0.341156 0.051306
H -3.452446 0.23052 2.826074
H -1.801899 0.88178 3.091488
H -2.070906 -1.544909 3.197745
C 1.958828 3.092293 -0.483975
O 3.336783 -1.687058 -1.613233
C 3.19963 -1.674846 -0.201191
C 2.266263 -0.490022 0.086088
O 1.740499 -0.571246 1.397287
C 2.952572 0.845 -0.115257
C 1.256663 1.938312 -0.151596
O 0.39169 1.378204 -1.081423
C 2.531597 -2.961929 0.27742
O 1.179164 -3.01985 -0.182776
O 2.874617 3.54918 0.296834
H 4.165497 -1.542395 0.305059
H 1.447352 -0.569691 -0.637126
H 1.049133 1.700969 0.886439
H 1.859456 3.545064 -1.474777
H 4.169859 -2.097962 -1.87098
H 3.325565 2.681798 0.783097
H 2.326085 -0.048495 1.972953
H 2.49186 -2.995912 1.366678
H 3.092148 -3.831271 -0.083723
H 1.19763 -3.076322 -1.148421
H 3.344515 0.987767 -1.1324
H -1.403304 2.332457 -0.747652
Na -0.28783 -1.445911 0.759836

Reactant state of 3rd c2 reaction of Manβ(1→2)Manβ
46

C -1.171752 0.090566 -1.229088
O -1.170756 1.396681 -0.72657
C -2.169766 1.824847 0.208461
C -3.575251 1.206671 -0.000646
O -4.309251 1.990336 -0.928342
C -3.621126 -0.181502 -0.644095
C -2.55751 -0.313966 -1.726648
O -2.497596 -1.65864 -2.166048
O -3.475285 -1.217076 0.320394
C -1.621763 1.832784 1.635771
O -1.20799 0.578502 2.137455
O 1.442124 0.327149 1.313382
H -2.300818 2.887749 -0.033269
H -4.092042 1.154134 0.967923
H -4.612488 -0.266994 -1.103067
H -2.810345 0.377595 -2.538462
H -2.948611 -1.756886 -3.012158
H -3.48529 -2.039253 -0.195711
H -4.487881 2.86674 -0.568632
H -2.395555 2.225497 2.304536
H -0.778128 2.532662 1.66098
H -0.267656 0.459273 1.891202
C 0.949133 -2.268849 0.637517
O 3.334624 2.121287 0.437299
C 3.956399 0.870011 0.175654
C 3.01097 -0.099157 -0.561723
O 3.413534 -1.433781 -0.352287
C 1.561404 0.074823 -0.083139
C 0.684934 -1.157657 -0.374416
O -0.699163 -0.854677 -0.2907
C 5.267007 1.081792 -0.573903
O 5.779246 -0.198274 -0.900666
O 0.123218 -2.592086 1.464841
H 4.165006 0.427688 1.15564
H 3.017677 0.131512 -1.642184
H 0.931312 -1.546251 -1.374442
H 1.927752 -2.758102 0.580188
H 3.325963 2.675127 -0.354056
H 1.894917 1.167744 1.491913
H 4.347009 -1.509759 -0.606037
H 5.958292 1.647837 0.059407
H 5.069914 1.661681 -1.489991
H 6.668368 -0.131561 -1.265581
H 1.133555 0.922335 -0.631288
H -0.485278 0.113519 -2.084319
Na -1.810308 -1.516349 1.775897

Transition state of 1st m1_4 reaction of Manβ(1→2)Manβ
46

C -1.445977 -0.718853 -1.030156
O -2.330658 0.107365 -1.710092
C -3.326379 0.847521 -0.989958
C -3.99177 0.023776 0.13051
O -4.83549 -0.967004 -0.422188
C -2.997173 -0.777744 0.982277
C -2.115298 -1.59345 0.039325
O -1.135079 -2.25198 0.830396
O -2.228123 0.026726 1.859439
C -2.853871 2.244974 -0.591461
O -2.015056 2.307309 0.553176
O 0.832224 1.817099 0.860472
H -4.099986 1.018361 -1.749191
H -4.548524 0.705334 0.790064
H -3.574394 -1.474586 1.596304
H -2.759965 -2.312602 -0.476676
H -0.750553 -2.987028 0.336706
H -2.241829 0.959833 1.545208
H -5.620904 -0.568732 -0.814561
H -3.736732 2.845161 -0.351359
H -2.361257 2.702542 -1.456353
H -1.093321 2.112145 0.321196
C 1.552218 -0.705796 -1.619862
O 4.416471 1.679327 -0.195767
C 4.001043 0.60663 0.611541
C 2.573313 0.191598 0.24182
O 2.059863 -0.798986 0.939887
C 1.573437 1.276424 -0.218378
C 0.644584 0.479301 -1.159919
O -0.423506 0.022113 -0.368963
C 4.948863 -0.578751 0.504942
O 5.007743 -0.869615 -0.885858
O 1.359047 -1.849328 -1.049306
H 3.9706 0.955431 1.652153
H 2.722972 -0.233463 -0.979245
H 0.277472 1.06043 -2.015196
H 1.965059 -0.716837 -2.632726
H 4.901894 1.314307 -0.951255
H 1.26411 2.613728 1.19515
H 1.568249 -1.584266 0.03545
H 4.556226 -1.423164 1.080295
H 5.931872 -0.287176 0.890426
H 5.676577 -1.53941 -1.065831
H 2.113401 2.051571 -0.769296
H -0.988369 -1.345649 -1.803076
Na -0.019093 -0.493083 1.902559

Reactant state of 1st m1_4 reaction of Manβ(1→2)Manβ
46

C -1.648641 -0.994156 -0.777827
O -2.433099 -0.14943 -1.555164
C -3.210942 0.900074 -0.960553
C -3.868318 0.482864 0.369076
O -4.925256 -0.426865 0.131498
C -2.928086 -0.28935 1.305011
C -2.329355 -1.453694 0.518357
O -1.381786 -2.109476 1.354982
O -1.924376 0.516391 1.89264
C -2.482587 2.242852 -0.936241
O -1.55349 2.403416 0.123878
O 1.126814 1.626729 0.089385
H -4.027778 1.034237 -1.680901
H -4.221411 1.384596 0.88985
H -3.536329 -0.70361 2.114241
H -3.148603 -2.124045 0.241094
H -1.407107 -3.062866 1.219652
H -1.809774 1.337208 1.353928
H -5.678542 0.020119 -0.271205
H -3.234431 3.029497 -0.814382
H -1.997324 2.393679 -1.906802
H -0.645004 2.174635 -0.138819
C 0.95193 -1.262764 -2.226661
O 3.850645 1.842037 0.225955
C 3.637733 0.546882 0.755005
C 2.661025 -0.256708 -0.12514
O 2.026979 -1.184518 0.75212
C 1.663391 0.679776 -0.826989
C 0.452069 -0.041612 -1.453661
O -0.413721 -0.391443 -0.400681
C 4.960696 -0.18304 0.920004
O 5.577657 -0.110757 -0.356948
O 1.012308 -2.357057 -1.717107
H 3.169127 0.672504 1.738232
H 3.23072 -0.798723 -0.892554
H -0.054244 0.641009 -2.151783
H 1.291379 -1.094214 -3.26511
H 4.624214 1.804724 -0.358046
H 1.82805 2.279265 0.258397
H 1.667709 -1.916965 0.223439
H 4.777233 -1.21943 1.227692
H 5.565479 0.323581 1.680421
H 6.482228 -0.440067 -0.320038
H 2.204028 1.197364 -1.628336
H -1.43665 -1.855957 -1.419914
Na 0.136558 -0.415955 1.837655

Transition state of 2nd m1_4 reaction of Manβ(1→2)Manβ
46

C 1.506044 -0.747099 1.036289
O 2.22931 0.217649 1.725556
C 3.062835 1.137881 1.008073
C 3.87865 0.457623 -0.108183
O 4.887547 -0.364419 0.446441
C 3.055798 -0.514671 -0.966933
C 2.324505 -1.479384 -0.029801
O 1.418548 -2.299492 -0.763607
O 2.161736 0.126509 -1.85639
C 2.342863 2.425339 0.601406
O 1.59036 2.359209 -0.600725
O -1.081265 1.419694 -0.913635
H 3.784152 1.45333 1.772636
H 4.304945 1.233476 -0.760929
H 3.766578 -1.088531 -1.570881
H 3.070617 -2.081865 0.497398
H 1.840893 -3.133473 -0.9976
H 2.008327 1.050705 -1.548153
H 5.559598 0.168415 0.886819
H 3.10564 3.193964 0.440644
H 1.714077 2.748329 1.438225
H 0.662158 2.123456 -0.438382
C -1.484351 -1.066768 1.683101
O -3.857438 1.178633 -1.387452
C -4.018019 -0.067226 -0.714275
C -2.594176 -0.390838 -0.238275
O -1.958133 -1.340334 -0.880275
C -1.73911 0.825408 0.188679
C -0.720488 0.184371 1.147759
O 0.384673 -0.188574 0.363563
C -5.031221 0.011256 0.418266
O -4.492664 0.831727 1.439406
O -1.168351 -2.212443 1.180479
H -4.31474 -0.857006 -1.413896
H -2.708655 -0.769476 0.998163
H -0.411195 0.839851 1.971036
H -1.889697 -1.064288 2.698291
H -4.687517 1.448374 -1.799189
H -1.762846 1.889478 -1.420564
H -1.329939 -2.046515 0.105564
H -5.225282 -1.005743 0.786434
H -5.973425 0.42014 0.029865
H -5.167903 1.030606 2.097375
H -2.371955 1.542762 0.718269
H 1.157977 -1.455452 1.794285
Na 0.041237 -0.738285 -1.860811

Reactant state of 2nd m1_4 reaction of Manβ(1→2)Manβ
46

C 1.625753 -1.171779 0.424029
O 2.431943 -0.571786 1.385799
C 3.053033 0.696048 1.135164
C 3.606288 0.821435 -0.297194
O 4.745429 -0.001374 -0.461859
C 2.638008 0.317776 -1.377982
C 2.189739 -1.095831 -0.99715
O 1.163811 -1.553155 -1.873044
O 1.539424 1.178866 -1.602521
C 2.220558 1.883496 1.620034
O 1.197453 2.31328 0.736102
O -1.384499 1.264539 0.481446
H 3.923715 0.674728 1.803238
H 3.840655 1.877506 -0.495996
H 3.206176 0.259882 -2.312676
H 3.06445 -1.75321 -1.028465
H 1.557379 -2.012301 -2.623824
H 1.420171 1.759781 -0.814157
H 5.484269 0.32842 0.062326
H 2.896833 2.735464 1.744882
H 1.809176 1.638508 2.605652
H 0.338328 1.897971 0.924988
C -0.786956 -2.077794 1.949256
O -2.98246 0.798514 -1.664252
C -3.69494 -0.04483 -0.759781
C -2.64793 -0.828012 0.048279
O -1.876696 -1.478264 -0.944282
C -1.81285 0.032794 1.038502
C -0.50135 -0.653363 1.481523
O 0.337737 -0.582456 0.355567
C -4.705479 0.741667 0.064067
O -4.033271 1.771254 0.7741
O -0.755951 -3.019677 1.193104
H -4.249704 -0.806073 -1.323596
H -3.194449 -1.571001 0.648899
H -0.073286 -0.088833 2.323765
H -1.056692 -2.205759 3.013742
H -3.569663 1.081462 -2.375441
H -2.169656 1.836229 0.400798
H -1.31261 -2.162721 -0.545753
H -5.218971 0.058568 0.754036
H -5.45295 1.168921 -0.615421
H -4.663288 2.397809 1.147153
H -2.437414 0.21909 1.921578
H 1.531528 -2.216523 0.734651
Na -0.530964 0.199503 -1.724013

Transition state of 3rd m1_4 reaction of Manβ(1→2)Manβ
46

C 1.580441 -0.601442 1.094895
O 2.47458 0.319785 1.622842
C 3.406859 0.996256 0.767004
C 4.031346 0.067062 -0.292178
O 4.932596 -0.836276 0.316929
C 3.014438 -0.847686 -0.989362
C 2.213808 -1.573513 0.089627
O 1.208527 -2.339418 -0.561545
O 2.17165 -0.166496 -1.902343
C 2.871834 2.328686 0.244751
O 1.968177 2.241435 -0.847625
O -0.869633 1.666289 -0.917101
H 4.216621 1.268324 1.455635
H 4.530613 0.682296 -1.054801
H 3.576739 -1.595139 -1.555905
H 2.908763 -2.214713 0.641579
H 0.909401 -3.054847 0.012824
H 2.175329 0.796426 -1.696195
H 5.73161 -0.381055 0.606427
H 3.721632 2.91818 -0.112541
H 2.415543 2.869392 1.081104
H 1.065781 2.058598 -0.541345
C -1.376389 -0.576779 1.87289
O -4.332447 1.494553 0.491031
C -3.964741 0.43912 -0.363024
C -2.529222 0.082047 -0.029803
O -2.031641 -0.977003 -0.630284
C -1.533638 1.23056 0.254669
C -0.531139 0.561568 1.219021
O 0.496468 0.032785 0.419194
C -4.904909 -0.749124 -0.21096
O -6.192527 -0.201268 -0.412648
O -1.19513 -1.774296 1.424501
H -3.984454 0.765299 -1.417261
H -2.600379 -0.191993 1.246337
H -0.126548 1.243393 1.977351
H -1.718979 -0.475913 2.906607
H -5.295656 1.589889 0.438651
H -1.342501 2.410761 -1.31127
H -1.46058 -1.638386 0.330852
H -4.803614 -1.167029 0.801705
H -4.658492 -1.519412 -0.948672
H -6.874417 -0.872181 -0.299673
H -2.064198 2.046889 0.753337
H 1.189274 -1.149077 1.959026
Na -0.020778 -0.74645 -1.749322

Reactant state of 3rd m1_4 reaction of Manβ(1→2)Manβ
46

C 1.735331 -1.078021 0.583093
O 2.569394 -0.336467 1.411427
C 3.269811 0.815149 0.919274
C 3.830323 0.613946 -0.501489
O 4.917124 -0.291108 -0.480593
C 2.833241 -0.042655 -1.467415
C 2.311389 -1.324021 -0.812957
O 1.271158 -1.905216 -1.59627
O 1.777315 0.811006 -1.862972
C 2.510749 2.122677 1.143798
O 1.502493 2.408098 0.186473
O -1.165985 1.528407 0.252949
H 4.137033 0.875043 1.589055
H 4.126854 1.591404 -0.908876
H 3.392384 -0.315493 -2.368711
H 3.150796 -2.015823 -0.69221
H 1.645277 -2.532899 -2.225157
H 1.692195 1.545787 -1.207826
H 5.68648 0.105847 -0.056262
H 3.233814 2.942878 1.087264
H 2.095877 2.114887 2.157687
H 0.620042 2.117345 0.473691
C -0.691648 -1.570294 2.237417
O -3.888066 1.577281 0.457528
C -3.677415 0.411634 -0.321509
C -2.604933 -0.436507 0.368294
O -2.006358 -1.219352 -0.657297
C -1.595219 0.467414 1.101241
C -0.310954 -0.256044 1.552549
O 0.468721 -0.454707 0.396515
C -4.984872 -0.336846 -0.511199
O -5.857693 0.612056 -1.094858
O -0.81804 -2.595821 1.611663
H -3.297801 0.694038 -1.316137
H -3.077809 -1.095147 1.110512
H 0.228536 0.377655 2.271258
H -0.881317 -1.533881 3.325656
H -4.722124 1.974753 0.162432
H -1.907305 2.156731 0.202767
H -1.544595 -1.972477 -0.248844
H -5.359303 -0.669762 0.466755
H -4.824997 -1.207357 -1.158466
H -6.747135 0.253146 -1.185131
H -2.097967 0.874329 1.98615
H 1.587353 -2.032671 1.097229
Na -0.277419 -0.149122 -1.755085

Transition state of 1st m1_5 reaction of Manβ(1→2)Manβ
46

C 1.444772 0.093872 -1.296773
O 1.939783 -1.202667 -1.28463
C 2.881222 -1.625262 -0.286026
C 3.937912 -0.553236 0.043976
O 4.861117 -0.415923 -1.019891
C 3.349999 0.857783 0.195958
C 2.52524 1.149748 -1.056719
O 1.860744 2.410316 -0.954185
O 2.589165 1.036159 1.373622
C 2.214766 -2.260743 0.931847
O 1.668322 -1.331942 1.854515
O -1.024441 -1.240592 1.420784
H 3.413742 -2.445279 -0.784018
H 4.442249 -0.82877 0.981191
H 4.192978 1.555963 0.23541
H 3.191138 1.127399 -1.924876
H 2.421615 3.105066 -1.318088
H 2.264389 0.147942 1.685928
H 5.421908 -1.197167 -1.088161
H 2.973668 -2.84403 1.464843
H 1.440961 -2.95324 0.583642
H 0.69907 -1.279557 1.733699
C -1.724031 1.263932 -0.512524
O -3.184425 0.457954 1.573256
C -3.57937 0.0134 0.397327
C -2.959314 -1.307548 -0.091991
O -3.150116 -1.515536 -1.465148
C -1.436655 -1.236402 0.069998
C -0.908133 -0.01676 -0.733326
O 0.417665 0.290561 -0.330718
C -5.042432 0.32483 0.123218
O -5.383854 -0.053427 -1.192455
O -1.561798 1.867156 0.636837
H -2.954739 0.774055 -0.507174
H -3.363566 -2.124299 0.526
H -0.94735 -0.286289 -1.793673
H -1.891075 1.907077 -1.385617
H -2.388017 1.245233 1.367647
H -1.653844 -0.738842 1.961539
H -4.080082 -1.373264 -1.699732
H -5.202301 1.393238 0.307438
H -5.611287 -0.24344 0.872783
H -6.323732 0.091244 -1.352699
H -1.007804 -2.131941 -0.38938
H 1.013889 0.232003 -2.294546
Na 0.678822 2.202612 1.019288

Reactant state of 1st m1_5 reaction of Manβ(1→2)Manβ
46

C 1.380979 -0.138586 -1.215536
O 1.421006 -1.484506 -0.857129
C 2.27946 -1.963495 0.189971
C 3.65576 -1.272438 0.21454
O 4.462148 -1.720963 -0.858921
C 3.572702 0.243877 0.000193
C 2.775736 0.486991 -1.281568
O 2.607584 1.884389 -1.520694
O 3.025564 0.942998 1.100036
C 1.572659 -2.033275 1.540934
O 1.375495 -0.771327 2.157567
O -1.268126 -0.510198 1.51243
H 2.461593 -3.007006 -0.09707
H 4.134146 -1.46669 1.184869
H 4.594747 0.60862 -0.146935
H 3.300947 0.007101 -2.113013
H 3.3173 2.207235 -2.087513
H 2.415927 0.33963 1.604755
H 4.738903 -2.633751 -0.719034
H 2.185194 -2.643406 2.214143
H 0.609792 -2.538656 1.40915
H 0.443954 -0.509598 2.020851
C -1.321873 2.008917 -0.111539
O -3.61893 0.775994 1.351465
C -3.982808 0.211134 0.097104
C -2.923316 -0.839064 -0.287191
O -2.889058 -1.004239 -1.685299
C -1.469549 -0.546828 0.112953
C -0.805828 0.665063 -0.579049
O 0.58949 0.654893 -0.339098
C -5.364147 -0.431261 0.180579
O -5.586001 -1.065478 -1.065607
O -0.616657 2.839674 0.421884
H -3.998969 0.981383 -0.688304
H -3.18069 -1.781733 0.221002
H -1.027318 0.587099 -1.656246
H -2.383386 2.22656 -0.297971
H -4.315933 1.349005 1.695304
H -1.981369 0.000399 1.928032
H -3.767499 -1.290888 -1.977401
H -6.124233 0.337922 0.375466
H -5.368716 -1.154362 1.007359
H -6.453081 -1.485357 -1.087387
H -0.901703 -1.406908 -0.255475
H 0.915407 -0.121143 -2.207953
Na 1.649657 2.610767 0.433814

Transition state of 2nd m1_5 reaction of Manβ(1→2)Manβ
46

C 1.442013 0.097214 -1.301059
O 1.935862 -1.19702 -1.2942
C 2.877901 -1.627775 -0.296713
C 3.93146 -0.563342 0.049157
O 4.85968 -0.554345 -1.021354
C 3.347981 0.854361 0.193077
C 2.525899 1.149059 -1.060458
O 1.867614 2.412894 -0.958713
O 2.587672 1.039827 1.370296
C 2.205713 -2.263004 0.91618
O 1.674297 -1.33123 1.847683
O -1.022703 -1.225217 1.430571
H 3.425422 -2.430615 -0.800681
H 4.411133 -0.850929 0.99477
H 4.186629 1.56135 0.233868
H 3.191005 1.120744 -1.929365
H 2.420318 3.104288 -1.340481
H 2.263757 0.149725 1.682496
H 5.719552 -0.242243 -0.716691
H 2.956966 -2.860986 1.442915
H 1.420633 -2.940775 0.564776
H 0.703942 -1.276609 1.740135
C -1.727805 1.263339 -0.51998
O -3.186729 0.469294 1.571683
C -3.580603 0.015495 0.398869
C -2.95757 -1.307299 -0.081578
O -3.147938 -1.524523 -1.453293
C -1.435047 -1.231908 0.079824
C -0.909218 -0.017057 -0.732375
O 0.41631 0.295544 -0.332584
C -5.044412 0.321749 0.122909
O -5.385725 -0.066959 -1.189682
O -1.566282 1.874742 0.625325
H -2.957466 0.77141 -0.510991
H -3.360064 -2.120844 0.541779
H -0.948243 -0.29446 -1.790695
H -1.896567 1.900352 -1.397244
H -2.391686 1.255924 1.360586
H -1.654305 -0.723079 1.968244
H -4.078612 -1.388265 -1.688472
H -5.206521 1.39115 0.299334
H -5.611617 -0.242162 0.877017
H -6.326252 0.073347 -1.34999
H -1.004477 -2.129727 -0.373412
H 1.009562 0.241239 -2.297479
Na 0.673869 2.203829 1.010602

Reactant state of 2nd m1_5 reaction of Manβ(1→2)Manβ
46

C 1.379708 -0.129237 -1.220399
O 1.419602 -1.475403 -0.873218
C 2.277047 -1.96701 0.17172
C 3.648435 -1.275849 0.218451
O 4.419277 -1.838181 -0.829436
C 3.568861 0.243895 0.001558
C 2.775846 0.49318 -1.281064
O 2.61411 1.892215 -1.515407
O 3.022581 0.942883 1.102258
C 1.564212 -2.044861 1.51715
O 1.379103 -0.785317 2.146681
O -1.266885 -0.509069 1.511533
H 2.479907 -2.999374 -0.131537
H 4.100082 -1.47455 1.19983
H 4.589227 0.620803 -0.144089
H 3.299542 0.01199 -2.113022
H 3.305113 2.211728 -2.106245
H 2.414296 0.333925 1.604485
H 5.35959 -1.766263 -0.628824
H 2.166511 -2.669157 2.185967
H 0.595864 -2.536641 1.375958
H 0.447818 -0.518305 2.020222
C -1.325523 2.011597 -0.107774
O -3.620833 0.771484 1.355927
C -3.984201 0.208435 0.100559
C -2.921951 -0.837353 -0.288585
O -2.888203 -0.995726 -1.687524
C -1.468552 -0.544002 0.112157
C -0.807005 0.670018 -0.57828
O 0.588676 0.661664 -0.339791
C -5.363133 -0.439125 0.184176
O -5.584399 -1.070774 -1.063415
O -0.621597 2.843392 0.426055
H -4.004163 0.980733 -0.68276
H -3.17695 -1.783014 0.215145
H -1.029444 0.59346 -1.655386
H -2.387926 2.226794 -0.291916
H -4.319946 1.340361 1.702314
H -1.981827 -0.002082 1.92852
H -3.764872 -1.287349 -1.97994
H -6.125787 0.326754 0.382157
H -5.363909 -1.164422 1.00904
H -6.448719 -1.496378 -1.083905
H -0.899444 -1.402452 -0.2581
H 0.915658 -0.101967 -2.213461
Na 1.643224 2.610903 0.43802

Transition state of 3rd m1_5 reaction of Manβ(1→2)Manβ
46

C -1.419786 -0.059472 -1.289685
O -2.09521 1.139703 -1.45176
C -3.066997 1.535045 -0.472103
C -4.001031 0.380762 -0.05868
O -4.884733 0.054979 -1.113586
C -3.269269 -0.944145 0.209785
C -2.355538 -1.229781 -0.984244
O -1.526679 -2.359491 -0.736373
O -2.54961 -0.972314 1.430995
C -2.463908 2.336099 0.682661
O -1.823795 1.575398 1.698526
O 1.030064 0.259099 1.81029
H -3.684358 2.256162 -1.022139
H -4.549215 0.674418 0.848832
H -4.034378 -1.725895 0.261328
H -2.981834 -1.378525 -1.869704
H -1.957543 -3.164597 -1.045704
H -2.443377 -0.057259 1.767126
H -5.502989 0.776306 -1.278465
H -3.274659 2.874657 1.181621
H -1.773724 3.07645 0.26257
H -0.978269 1.244458 1.35923
C 1.617321 -0.993587 -0.880962
O 3.460149 -1.159225 1.097101
C 3.650353 -0.212242 0.223041
C 2.991411 1.143869 0.525702
O 3.122327 2.089814 -0.500224
C 1.468112 1.004972 0.679963
C 0.879452 0.317912 -0.564382
O -0.469072 0.019781 -0.22948
C 5.033866 -0.269067 -0.409084
O 5.101555 0.601412 -1.520753
O 1.536665 -1.93988 0.010529
H 2.856538 -0.534602 -0.841326
H 3.433215 1.497927 1.472991
H 0.939636 1.01216 -1.409383
H 1.645894 -1.307105 -1.932921
H 2.506333 -1.676996 0.798099
H 1.423497 0.616207 2.616681
H 3.968722 1.983363 -0.963064
H 5.227872 -1.311197 -0.682791
H 5.743957 0.02224 0.378247
H 5.985499 0.587817 -1.906403
H 1.070923 2.028918 0.724487
H -0.892177 -0.237347 -2.232936
Na -0.393343 -1.799451 1.234494

Reactant state of 3rd m1_5 reaction of Manβ(1→2)Manβ
46

C 1.576387 0.099776 -1.207867
O 2.072575 -1.181852 -1.394105
C 2.983331 -1.761205 -0.442376
C 4.018627 -0.761577 0.110719
O 5.015793 -0.49041 -0.856465
C 3.445578 0.631316 0.418162
C 2.670021 1.094437 -0.811722
O 2.04431 2.346603 -0.572526
O 2.637703 0.707219 1.581391
C 2.251325 -2.56852 0.623322
O 1.595952 -1.770614 1.598538
O -1.453186 0.634609 1.591338
H 3.538536 -2.490409 -1.044807
H 4.456125 -1.179538 1.02919
H 4.301489 1.298187 0.566578
H 3.362764 1.149294 -1.657879
H 2.62538 3.06706 -0.841517
H 2.403409 -0.195534 1.876814
H 5.566124 -1.267618 -1.007022
H 2.976846 -3.182567 1.163999
H 1.536548 -3.23662 0.130045
H 0.887558 -1.292761 1.142092
C -1.260457 1.414426 -1.143374
O -3.725185 1.435173 0.361881
C -3.98947 0.182665 -0.256699
C -3.031545 -0.845537 0.365104
O -2.893691 -1.965583 -0.474774
C -1.591704 -0.370466 0.602783
C -0.804015 0.039273 -0.664957
O 0.544608 0.126799 -0.212073
C -5.444906 -0.218232 -0.031423
O -5.600345 -1.521595 -0.561988
O -0.841362 2.425106 -0.627267
H -3.790749 0.226691 -1.338324
H -3.423346 -1.126654 1.355919
H -0.921171 -0.728628 -1.438772
H -2.008267 1.459485 -1.954121
H -4.32569 2.121625 0.045312
H -2.216087 1.232501 1.51721
H -3.774755 -2.33683 -0.63601
H -6.108621 0.497037 -0.536247
H -5.651854 -0.19412 1.046868
H -6.508577 -1.827364 -0.460037
H -1.082218 -1.262554 0.984617
H 1.137992 0.392706 -2.168064
Na 0.627468 1.935894 1.216592

Transition state of 1st m1_6 reaction of Manβ(1→2)Manβ
46

C 1.442426 -0.040628 -1.284861
O 1.75277 -1.379729 -1.06683
C 2.604562 -1.791258 0.014561
C 3.765474 -0.815815 0.288021
O 4.752408 -0.915705 -0.721681
C 3.344448 0.659203 0.240323
C 2.641045 0.891744 -1.095436
O 2.152417 2.230141 -1.19133
O 2.538485 1.059209 1.330346
C 1.817823 -2.205396 1.25457
O 1.291295 -1.117784 1.996519
O -1.336945 -0.982858 1.270071
H 3.058809 -2.715273 -0.365138
H 4.183571 -1.032497 1.281245
H 4.260282 1.259156 0.266988
H 3.346413 0.673352 -1.90306
H 2.821353 2.793116 -1.597752
H 2.072629 0.262742 1.702947
H 5.234883 -1.74694 -0.646482
H 2.491713 -2.762744 1.914597
H 1.012545 -2.882618 0.949695
H 0.341 -1.014951 1.786956
C -1.702764 1.481612 -0.692123
O -5.225045 -0.925904 -0.398363
C -4.026771 -0.684863 0.30223
C -2.942257 -1.395246 -0.534671
O -3.077086 -1.024475 -1.888491
C -1.492419 -1.063996 -0.137514
C -0.933648 0.16189 -0.888302
O 0.391921 0.42312 -0.446584
C -3.799278 0.821318 0.458464
O -3.185802 1.221462 1.542919
O -1.409835 2.174362 0.37884
H -4.046297 -1.113468 1.311839
H -3.111872 -2.471414 -0.405178
H -0.941116 -0.083715 -1.954521
H -1.914147 2.054563 -1.604003
H -5.984618 -0.94152 0.19521
H -1.88352 -0.274392 1.653506
H -3.968067 -1.263298 -2.182378
H -4.59737 1.461731 0.056677
H -2.934904 1.062676 -0.545724
H -2.322791 1.868429 1.204554
H -0.856364 -1.899501 -0.446511
H 1.099387 0.00936 -2.324491
Na 0.833634 2.413738 0.696011

Reactant state of 1st m1_6 reaction of Manβ(1→2)Manβ
46

C 1.464136 0.019771 -1.241055
O 1.599465 -1.357946 -1.080813
C 2.408559 -1.923857 -0.036087
C 3.690245 -1.120286 0.255415
O 4.649235 -1.314351 -0.767946
C 3.466425 0.397438 0.261925
C 2.787937 0.763774 -1.056309
O 2.504029 2.161184 -1.116703
O 2.730708 0.862209 1.375953
C 1.587267 -2.277434 1.199296
O 1.178969 -1.150508 1.955729
O -1.382575 -0.938829 1.075629
H 2.732866 -2.881869 -0.461949
H 4.083054 -1.424227 1.235864
H 4.452902 0.871644 0.295759
H 3.44168 0.46076 -1.879848
H 3.225907 2.623999 -1.556894
H 2.142903 0.131456 1.709307
H 5.014079 -2.20579 -0.728397
H 2.19761 -2.915635 1.847782
H 0.710104 -2.854256 0.885704
H 0.254932 -0.945078 1.703725
C -1.522131 1.818622 -0.30763
O -5.218489 -1.347881 -0.633974
C -4.107333 -0.949514 0.153467
C -2.946565 -0.986673 -0.855875
O -3.173131 -0.049462 -1.893987
C -1.506035 -0.765263 -0.322213
C -0.841972 0.554659 -0.791906
O 0.505235 0.594961 -0.361045
C -4.42795 0.420274 0.744231
O -3.316132 0.849567 1.527431
O -0.96246 2.640188 0.391209
H -3.90122 -1.669155 0.956207
H -2.987229 -2.003084 -1.267298
H -0.905321 0.580358 -1.889643
H -2.549088 1.973134 -0.66751
H -5.932049 -1.692472 -0.08555
H -1.979306 -0.317132 1.534213
H -4.001601 -0.285884 -2.334969
H -5.324509 0.336045 1.367875
H -4.628901 1.12569 -0.070742
H -3.538424 1.62431 2.056999
H -0.871237 -1.544875 -0.752903
H 1.106638 0.154667 -2.268656
Na 1.274717 2.505026 0.779956

Transition state of 2nd m1_6 reaction of Manβ(1→2)Manβ
46

C 1.264329 -0.190419 1.451191
O 1.103706 1.200892 1.340809
C 1.891703 1.902194 0.349681
C 2.053769 0.989683 -0.865998
O 2.654627 1.632484 -1.959426
C 2.917087 -0.207769 -0.500967
C 2.646665 -0.627816 0.966074
O 2.71594 -2.03079 1.15718
O 2.641321 -1.307768 -1.363331
C 1.190318 3.204191 -0.021873
O 0.261504 3.011103 -1.066855
O -1.444192 1.103795 2.335531
H 2.867614 2.142971 0.795473
H 1.057833 0.61547 -1.132869
H 3.972268 0.076248 -0.607151
H 3.371317 -0.119225 1.611578
H 3.634896 -2.312031 1.247278
H 2.814903 -1.017169 -2.269934
H 1.996164 2.219666 -2.36092
H 1.95008 3.909643 -0.372204
H 0.725889 3.632154 0.874648
H -0.646687 3.011334 -0.721784
C -1.688092 -2.225685 0.554872
O -4.290155 0.459877 -0.294167
C -3.047536 0.363784 -0.938807
C -1.915642 0.898283 -0.050713
O -2.178052 2.284225 0.123817
C -1.844192 0.197243 1.331927
C -0.978564 -1.073108 1.308483
O 0.300178 -0.899162 0.697428
C -2.885079 -1.094101 -1.314854
O -1.857594 -1.449451 -2.023175
O -1.0057 -2.868247 -0.355669
H -3.032868 0.934081 -1.880289
H -0.96144 0.788387 -0.579365
H -0.830248 -1.387835 2.352036
H -2.353278 -2.828085 1.187476
H -4.514614 1.393393 -0.185783
H -0.539907 1.411479 2.148818
H -2.211132 2.467744 1.077353
H -3.830626 -1.639758 -1.447623
H -2.636182 -1.620563 -0.065916
H -1.353552 -2.259082 -1.45427
H -2.857751 -0.110929 1.620153
H 1.141129 -0.440915 2.512209
Na 1.217721 -2.79832 -0.427286

Reactant state of 2nd m1_6 reaction of Manβ(1→2)Manβ
46

C 1.223291 -0.38726 1.524443
O 1.171774 1.014486 1.528499
C 1.97378 1.734384 0.561933
C 2.042153 0.920653 -0.730041
O 2.67352 1.60944 -1.77811
C 2.80325 -0.372732 -0.507128
C 2.509063 -0.943686 0.905412
O 2.349361 -2.348204 0.897176
O 2.431345 -1.336199 -1.490511
C 1.351045 3.109916 0.312864
O 0.463894 3.092603 -0.788178
O -1.580963 1.230677 2.301384
H 2.976994 1.87202 0.989661
H 1.016625 0.646839 -1.006631
H 3.8777 -0.167266 -0.595205
H 3.325278 -0.646498 1.57443
H 3.207827 -2.778835 0.797725
H 2.724239 -0.997945 -2.347986
H 2.073798 2.310198 -2.07686
H 2.15627 3.811898 0.077106
H 0.865874 3.457105 1.232826
H -0.466301 3.111271 -0.504851
C -1.817543 -2.252738 1.038494
O -4.046322 0.423317 -0.818582
C -2.698139 0.482678 -1.22261
C -1.798672 0.995737 -0.093489
O -2.134764 2.377112 0.045126
C -1.942209 0.311648 1.283535
C -1.108 -0.977667 1.444185
O 0.146766 -0.939808 0.797206
C -2.383455 -0.908854 -1.768466
O -0.982588 -1.134773 -1.952045
O -1.299148 -3.111211 0.361614
H -2.569498 1.214462 -2.035331
H -0.756303 0.927427 -0.41466
H -0.969034 -1.088538 2.534842
H -2.841241 -2.379303 1.433734
H -4.383953 1.322519 -0.716176
H -0.629931 1.424174 2.224139
H -2.236538 2.571252 0.991101
H -2.925674 -1.051336 -2.707642
H -2.747472 -1.672867 -1.081362
H -0.697258 -0.681185 -2.75465
H -2.997495 0.076213 1.465428
H 1.145364 -0.710511 2.569827
Na 0.580031 -2.533009 -0.815717

Transition state of 3rd m1_6 reaction of Manβ(1→2)Manβ
46

C 1.439736 -0.034365 -1.28958
O 1.750639 -1.371899 -1.0798
C 2.603462 -1.793124 -0.000191
C 3.758382 -0.822175 0.292427
O 4.736167 -1.046759 -0.708227
C 3.341524 0.657479 0.238763
C 2.640384 0.894253 -1.097131
O 2.157335 2.23485 -1.19051
O 2.537107 1.061443 1.328779
C 1.812233 -2.211218 1.233641
O 1.298179 -1.1227 1.986547
O -1.334386 -0.979653 1.271227
H 3.075601 -2.701261 -0.389013
H 4.149825 -1.048534 1.293589
H 4.2534 1.267726 0.266161
H 3.344222 0.672235 -1.905396
H 2.815002 2.793739 -1.619709
H 2.073226 0.261769 1.700402
H 5.609169 -0.792792 -0.387129
H 2.478665 -2.783367 1.887892
H 0.998349 -2.87447 0.921592
H 0.34633 -1.016211 1.787362
C -1.707437 1.482603 -0.692233
O -5.224571 -0.93158 -0.390967
C -4.025391 -0.687691 0.307101
C -2.940796 -1.395786 -0.531628
O -3.078459 -1.024383 -1.885021
C -1.491058 -1.061962 -0.136155
C -0.935497 0.164551 -0.888235
O 0.390181 0.42879 -0.448367
C -3.801026 0.819144 0.461655
O -3.187287 1.222152 1.544958
O -1.414412 2.177389 0.377544
H -4.041927 -1.115725 1.316991
H -3.108214 -2.472313 -0.402507
H -0.943609 -0.082003 -1.954242
H -1.921644 2.054089 -1.604397
H -5.982592 -0.950751 0.204467
H -1.881764 -0.271975 1.654743
H -3.968487 -1.267126 -2.178506
H -4.600768 1.457368 0.05968
H -2.938061 1.061151 -0.54374
H -2.326437 1.870345 1.205015
H -0.853833 -1.896359 -0.445546
H 1.095855 0.023499 -2.328679
Na 0.828785 2.414501 0.692823

Reactant state of 3rd m1_6 reaction of Manβ(1→2)Manβ
46

C 1.463302 0.027899 -1.246066
O 1.599298 -1.348183 -1.095988
C 2.406684 -1.924651 -0.052865
C 3.681736 -1.125707 0.259666
O 4.615798 -1.440687 -0.758812
C 3.462813 0.396392 0.262023
C 2.788372 0.76793 -1.056386
O 2.510424 2.166736 -1.112509
O 2.728194 0.863955 1.375942
C 1.5792 -2.28279 1.17513
O 1.185813 -1.158015 1.946091
O -1.38115 -0.931485 1.080641
H 2.751095 -2.868556 -0.487807
H 4.046599 -1.437098 1.247762
H 4.446731 0.881081 0.298284
H 3.441152 0.462496 -1.880125
H 3.216228 2.627886 -1.579124
H 2.143127 0.128891 1.70839
H 5.516326 -1.316612 -0.437376
H 2.179087 -2.937945 1.815761
H 0.693864 -2.841874 0.853088
H 0.259946 -0.946891 1.70706
C -1.525364 1.820463 -0.310367
O -5.2154 -1.356748 -0.62764
C -4.105212 -0.952877 0.158336
C -2.944562 -0.988902 -0.851093
O -3.173825 -0.053857 -1.890717
C -1.504625 -0.763069 -0.317728
C -0.843147 0.556697 -0.791938
O 0.504601 0.600495 -0.362795
C -4.430168 0.417524 0.745379
O -3.319369 0.853592 1.526411
O -0.966998 2.644439 0.386953
H -3.89661 -1.669688 0.96295
H -2.982575 -2.006064 -1.260828
H -0.9078 0.578709 -1.889653
H -2.552563 1.972727 -0.670418
H -5.927027 -1.703684 -0.078194
H -1.979372 -0.309894 1.537174
H -4.001001 -0.294118 -2.332029
H -5.325971 0.331924 1.369937
H -4.634281 1.11977 -0.071551
H -3.545021 1.628117 2.054838
H -0.868136 -1.542538 -0.746133
H 1.106732 0.172218 -2.272855
Na 1.267837 2.50507 0.7787

Transition state of 1st RO1' reaction of Manβ(1→2)Manβ
46

C 0.984049 -1.014017 -0.000755
O 1.813301 -1.064609 1.136674
C 3.222085 -1.172049 0.949766
C 3.620007 -1.259009 -0.524147
O 3.156414 -2.46761 -1.09613
C 2.983009 -0.172983 -1.391887
C 1.475377 0.017647 -1.045523
O 1.230612 1.32709 -0.586142
O 3.694878 1.058418 -1.259467
C 3.858733 -0.01713 1.747838
O 3.08795 1.169302 1.640793
O -2.385283 0.051262 1.931384
H 3.543733 -2.111667 1.42174
H 4.713858 -1.178077 -0.599418
H 3.057092 -0.51823 -2.426784
H 0.900788 -0.15622 -1.963698
H 0.239582 1.459122 -0.557355
H 4.405249 1.071893 -1.910703
H 3.532607 -3.230356 -0.639689
H 4.864572 0.2068 1.384089
H 3.928406 -0.308172 2.80089
H 2.169804 0.895352 1.803894
C -1.801217 0.180396 -1.170273
O -3.135142 0.112468 -1.564739
C -4.172503 0.039574 -0.586155
C -3.900078 -1.063842 0.44162
O -4.069108 -2.285216 -0.255179
C -2.482465 -1.022936 1.012607
C -1.451406 -0.900377 -0.113725
O -0.231476 -0.597543 0.550682
C -4.555049 1.418887 -0.047083
O -3.52172 2.119145 0.633023
O -1.395573 1.46481 -0.761914
H -5.042339 -0.30868 -1.153693
H -4.62021 -0.96725 1.266006
H -1.397822 -1.867854 -0.629894
H -1.247854 -0.016211 -2.092638
H -2.112222 1.881858 -0.214733
H -1.457979 0.125178 2.197882
H -4.185467 -3.010305 0.369798
H -5.434228 1.316529 0.600965
H -4.829202 2.043276 -0.899736
H -3.283822 1.618395 1.431917
H -2.290843 -1.964786 1.545763
H 0.880894 -2.005779 -0.460573
Na 2.820301 2.701827 0.012763

Reactant state of 1st RO1' reaction of Manβ(1→2)Manβ
46

C 1.110307 -0.355156 -0.769014
O 1.259835 -0.579125 0.628805
C 2.511553 -1.118012 1.093371
C 3.512579 -1.321137 -0.04872
O 3.056294 -2.46959 -0.743284
C 3.613296 -0.135069 -1.03078
C 2.237378 0.568113 -1.237632
O 2.138206 1.797218 -0.536275
O 4.60892 0.743142 -0.547808
C 2.999428 -0.226788 2.228713
O 3.281546 1.102995 1.815519
O -1.381942 -0.287458 1.48745
H 2.312105 -2.115111 1.5051
H 4.506684 -1.485221 0.387634
H 3.925157 -0.559727 -1.993133
H 2.087067 0.801227 -2.295833
H 2.486444 1.651503 0.377174
H 4.705386 1.501725 -1.138036
H 3.770789 -2.856003 -1.264125
H 3.876345 -0.681616 2.703463
H 2.208662 -0.157601 2.979842
H 4.163313 1.13331 1.412054
C -2.368299 0.714092 -1.306515
O -3.663142 0.20785 -1.218545
C -4.060729 -0.525058 -0.058233
C -3.034254 -1.604504 0.321784
O -3.003523 -2.629479 -0.652316
C -1.589353 -1.099573 0.347249
C -1.314335 -0.35615 -0.96226
O -0.074989 0.356737 -0.940937
C -4.544787 0.390835 1.068439
O -3.564524 1.282263 1.580563
O -2.181333 1.898328 -0.553866
H -4.96115 -1.060243 -0.388061
H -3.279489 -2.001479 1.317447
H -1.307375 -1.092464 -1.775213
H -2.244164 1.000397 -2.354806
H -2.71625 1.825685 0.282238
H -0.422083 -0.221523 1.608259
H -3.867699 -3.049866 -0.731833
H -4.960511 -0.224398 1.875824
H -5.34332 1.020108 0.670561
H -2.860856 0.769313 2.012587
H -0.946487 -1.98575 0.403113
H 1.087015 -1.308721 -1.311805
Na -0.041734 2.556713 -0.428664

Transition state of 2nd RO1' reaction of Manβ(1→2)Manβ
46

C 0.984747 -1.150671 0.921723
O 1.379269 0.313344 1.092952
C 2.828644 0.5572 1.190342
C 3.653282 -0.207885 0.143661
O 4.938111 -0.265428 0.720402
C 3.107704 -1.596628 -0.224653
C 1.593453 -1.4645 -0.461946
O 1.376092 -0.416752 -1.315736
O 3.765669 -2.036769 -1.386258
C 2.983948 2.059112 1.072726
O 2.357334 2.469235 -0.153828
O -2.537378 -0.004874 2.042776
H 3.112813 0.222377 2.191702
H 3.670422 0.378486 -0.782525
H 3.353817 -2.316113 0.566399
H 1.187961 -2.43382 -0.805496
H 1.064572 0.649231 0.19414
H 3.336814 -1.595356 -2.135616
H 5.531624 -0.713841 0.102378
H 4.054963 2.285269 1.061549
H 2.502658 2.558637 1.918792
H 2.413464 3.431645 -0.201078
C -1.363316 -0.112578 -0.917561
O -2.605375 0.060989 -1.529674
C -3.787668 0.244345 -0.750906
C -3.928045 -0.863826 0.296677
O -4.19361 -2.049476 -0.428011
C -2.656669 -1.072794 1.118775
C -1.428976 -1.180387 0.204007
O -0.351525 -1.185324 1.161593
C -3.953172 1.689956 -0.280317
O -2.907691 2.187249 0.54779
O -0.776778 1.117676 -0.508742
H -4.600462 0.0678 -1.463688
H -4.749712 -0.609671 0.979723
H -1.469785 -2.15199 -0.300279
H -0.716979 -0.500193 -1.699899
H -1.482273 1.659894 -0.057308
H -1.713524 -0.122771 2.534855
H -4.649351 -2.689069 0.131249
H -4.919783 1.795528 0.226468
H -3.9622 2.328762 -1.165955
H -2.917016 1.688992 1.383728
H -2.748788 -2.017785 1.67047
H 1.481152 -1.644783 1.761216
Na 0.951189 1.527432 -1.993828

Reactant state of 2nd RO1' reaction of Manβ(1→2)Manβ
46

C 1.104722 -0.515446 0.910246
O 1.895074 0.634141 1.100292
C 3.320896 0.551278 1.024012
C 3.874627 -0.462294 0.001027
O 5.132722 -0.841444 0.493663
C 2.959295 -1.667521 -0.218141
C 1.526446 -1.182825 -0.401313
O 1.406369 -0.241736 -1.469346
O 3.435652 -2.457647 -1.279531
C 3.733296 1.963265 0.643136
O 3.00427 2.29245 -0.556678
O -2.619036 0.886336 1.746341
H 3.731239 0.283637 2.00532
H 4.007552 0.044218 -0.97138
H 3.006322 -2.303786 0.674202
H 0.875774 -2.028694 -0.645073
H 2.229361 0.259259 -1.570113
H 3.149605 -2.092828 -2.128093
H 5.529361 -1.481636 -0.113656
H 4.812309 1.990491 0.464454
H 3.470259 2.667331 1.437923
H 3.464732 2.999858 -1.023866
C -1.640378 -0.19828 -1.027998
O -2.885415 -0.670367 -1.445535
C -4.021875 -0.547465 -0.590915
C -3.722048 -1.04052 0.834611
O -3.495219 -2.437115 0.838882
C -2.426015 -0.476523 1.414408
C -1.298449 -0.67701 0.401975
O -0.173752 0.068014 0.874285
C -4.708514 0.813792 -0.731232
O -3.909986 1.933933 -0.380438
O -1.497316 1.203044 -1.148525
H -4.740017 -1.263412 -1.012829
H -4.553256 -0.764258 1.498846
H -1.086737 -1.751491 0.367436
H -0.92807 -0.631021 -1.733763
H -2.368331 1.627921 -0.936137
H -1.820703 1.210854 2.181491
H -4.28447 -2.911076 0.552012
H -5.637392 0.807608 -0.147455
H -4.966737 0.948798 -1.783412
H -3.728676 1.90452 0.572998
H -2.194316 -1.049123 2.32054
H 1.190905 -1.214149 1.757182
Na 0.571124 1.857036 -0.501165

Transition state of 3rd RO1' reaction of Manβ(1→2)Manβ
46

C -1.044282 -0.857011 -0.963851
O -1.422845 0.776553 -0.272432
C -2.736078 1.283514 -0.531484
C -3.831852 0.249271 -0.20656
O -4.993248 0.741355 -0.830716
C -3.513457 -1.177991 -0.709578
C -2.106014 -1.562446 -0.197732
O -1.949856 -1.293102 1.12218
O -4.467644 -2.068204 -0.193425
C -2.901349 2.508148 0.379977
O -2.608533 2.069132 1.693638
O 1.466659 0.736634 1.148171
H -2.791438 1.573585 -1.588278
H -3.967855 0.196638 0.888895
H -3.582075 -1.220371 -1.805865
H -1.747034 -2.518473 -0.565907
H -1.565864 0.232187 0.686866
H -4.277246 -2.213514 0.747161
H -5.696683 0.07927 -0.751795
H -3.935016 2.871992 0.297397
H -2.205405 3.301824 0.069855
H -2.918919 2.714875 2.340015
C 2.486708 -1.481875 -0.700953
O 3.71839 -0.936399 -1.055594
C 4.106013 0.37276 -0.635701
C 2.951975 1.40235 -0.696075
O 2.726692 1.802494 -2.032641
C 1.584132 0.860027 -0.244627
C 1.346604 -0.480797 -0.974048
O 0.15396 -1.102862 -0.483882
C 4.842013 0.337578 0.719661
O 3.985398 0.115605 1.821003
O 2.436216 -1.878512 0.653976
H 4.849255 0.681397 -1.386963
H 3.219113 2.269677 -0.068268
H 1.273199 -0.298653 -2.056235
H 2.361336 -2.370028 -1.346388
H 3.007199 -2.649513 0.768132
H 2.362468 0.557668 1.522606
H 3.469732 2.305752 -2.391456
H 5.325739 1.307203 0.879834
H 5.614375 -0.442176 0.673957
H 3.72042 -0.818385 1.825793
H 0.822009 1.577155 -0.579669
H -1.11487 -0.632646 -2.037126
Na -0.153675 -0.629836 1.807369

Reactant state of 3rd RO1' reaction of Manβ(1→2)Manβ
46

C -1.201995 -0.478437 -0.976428
O -1.411283 0.725677 -0.290971
C -2.714929 1.285055 -0.491452
C -3.790256 0.309946 -0.001806
O -5.028037 0.837452 -0.405383
C -3.577156 -1.090934 -0.57842
C -2.131199 -1.537772 -0.387638
O -1.810977 -1.691517 1.00114
O -4.495608 -2.004995 -0.03035
C -2.747153 2.589366 0.278914
O -2.507254 2.27475 1.639723
O 1.594935 -0.073327 1.495366
H -2.876712 1.486935 -1.561579
H -3.734032 0.264439 1.098361
H -3.792932 -1.052492 -1.654214
H -1.969113 -2.514567 -0.851935
H -1.885138 -0.814613 1.416924
H -4.21762 -2.229949 0.868722
H -5.724136 0.206146 -0.177855
H -3.732578 3.045167 0.130934
H -1.970758 3.256913 -0.114879
H -2.707767 3.03865 2.190465
C 2.374027 -0.85793 -1.333738
O 3.551221 -0.128326 -1.428694
C 3.915473 0.849046 -0.43974
C 2.714494 1.643665 0.106579
O 2.266815 2.587766 -0.848271
C 1.469725 0.787377 0.371515
C 1.177113 -0.005699 -0.906388
O 0.101216 -0.920935 -0.722986
C 4.833802 0.24644 0.617327
O 4.186027 -0.695314 1.463366
O 2.491444 -1.970208 -0.42472
H 4.533035 1.560679 -1.001738
H 3.011276 2.133917 1.044976
H 0.958149 0.69977 -1.715912
H 2.202145 -1.260466 -2.335601
H 2.901594 -2.701132 -0.905444
H 2.546375 -0.23703 1.686327
H 2.921053 3.285641 -0.969379
H 5.208666 1.042216 1.266743
H 5.688973 -0.220102 0.118065
H 3.997688 -1.484248 0.934508
H 0.63551 1.461931 0.568585
H -1.36128 -0.337037 -2.059103
Na 0.538904 -2.054518 1.171356

Transition state of 1st c0',3' reaction of Manβ(1$\rightarrow$2)Manβ
46

C -1.04073 -0.72429 -1.201759
O -1.602916 1.098377 0.170853
C -2.970364 1.14394 -0.197251
C -3.709923 -0.131982 0.270037
O -4.623607 -0.581481 -0.703812
C -2.725679 -1.26139 0.569718
C -1.980128 -1.769887 -0.807152
O -1.295911 -2.931528 -0.371924
O -1.7973 -0.920631 1.492188
C -3.662967 2.389289 0.329912
O -5.01406 2.25408 -0.088028
O 1.76112 -0.585322 1.414159
H -3.040281 1.170314 -1.297831
H -4.218474 0.069472 1.223795
H -3.28397 -2.182243 0.803021
H -2.760195 -1.949959 -1.551278
H -1.106784 -3.5119 -1.119254
H -1.556354 0.3763 0.939564
H -5.306733 0.098086 -0.799443
H -3.196793 3.28998 -0.086106
H -3.581579 2.414654 1.425269
H -5.54124 3.000557 0.215476
C 2.317359 -0.242678 -1.592797
O 3.319426 0.727478 -1.557367
C 3.739915 1.293058 -0.308556
C 2.569188 1.630173 0.628345
O 2.018773 2.82932 0.117454
C 1.456398 0.566916 0.657481
C 1.103199 0.234921 -0.796451
O 0.14337 -0.848588 -0.740553
C 4.891278 0.538333 0.33848
O 4.445268 -0.638219 1.006748
O 2.697824 -1.476609 -1.05619
H 4.156366 2.260799 -0.605225
H 2.955624 1.758683 1.648825
H 0.648261 1.106775 -1.273626
H 2.069354 -0.336332 -2.657286
H 3.502269 -1.777136 -1.499369
H 2.69226 -0.842402 1.247796
H 1.372386 3.193067 0.735432
H 5.368256 1.218649 1.054103
H 5.615806 0.28373 -0.442983
H 5.143809 -0.981001 1.575734
H 0.573278 1.016251 1.12188
H -1.279188 0.087553 -1.885226
Na 0.039341 -2.10703 1.492588

Reactant state of 1st c0',3' reaction of Manβ(1→2)Manβ
46

C 1.242657 -0.368006 1.255556
O 1.563876 0.798885 0.516911
C 2.974416 0.964012 0.376856
C 3.50707 -0.052675 -0.651591
O 4.815629 -0.450294 -0.332699
C 2.628108 -1.307414 -0.622986
C 2.139507 -1.564578 0.800574
O 1.41367 -2.782987 0.774818
O 1.515468 -1.22358 -1.509994
C 3.248247 2.394709 -0.045535
O 4.635734 2.426005 -0.329508
O -1.185448 0.030346 -1.393597
H 3.474363 0.774795 1.339785
H 3.453785 0.375219 -1.664593
H 3.227705 -2.158769 -0.952727
H 3.014843 -1.646841 1.455541
H 1.350381 -3.16105 1.659489
H 1.137428 -0.330458 -1.488096
H 5.379841 0.336315 -0.36087
H 2.97874 3.089589 0.757709
H 2.650875 2.627572 -0.938943
H 4.918451 3.315988 -0.565529
C -2.37165 -0.604205 1.357344
O -3.534133 0.156024 1.311525
C -3.794382 1.032214 0.208909
C -2.550116 1.785254 -0.282716
O -2.365566 2.84743 0.636411
C -1.264142 0.941657 -0.308947
C -1.118149 0.212637 1.03302
O -0.067518 -0.739512 0.972576
C -4.626006 0.383517 -0.887719
O -3.833385 -0.444232 -1.739565
O -2.411896 -1.70081 0.450404
H -4.441801 1.798 0.64764
H -2.748508 2.162012 -1.296449
H -0.959616 0.941492 1.836626
H -2.322982 -0.983462 2.382958
H -3.257068 -2.153606 0.570031
H -2.093619 -0.207129 -1.671547
H -1.763425 3.505004 0.268668
H -5.084471 1.188932 -1.473402
H -5.41964 -0.206511 -0.416521
H -4.325066 -0.65268 -2.542488
H -0.422028 1.630316 -0.418589
H 1.352518 -0.138692 2.328556
Na -0.415046 -2.21336 -0.701099

Transition state of 2nd c0',3' reaction of Manβ(1→2)Manβ
46

C -1.039425 -0.718369 -1.189117
O -1.608117 1.09401 0.196805
C -2.972747 1.148097 -0.181454
C -3.720945 -0.125311 0.276353
O -4.639023 -0.562754 -0.69918
C -2.741235 -1.263689 0.559164
C -1.991379 -1.755971 -0.815951
O -1.260151 -2.921389 -0.470553
O -1.815215 -0.948207 1.492674
C -3.662555 2.395017 0.345704
O -5.012212 2.266831 -0.079203
O 1.749679 -0.527021 1.439188
H -3.034237 1.178849 -1.282441
H -4.225974 0.071945 1.232867
H -3.323517 -2.175166 0.786936
H -2.745324 -1.911532 -1.593121
H -1.874884 -3.665004 -0.428887
H -1.566681 0.367268 0.957554
H -5.314966 0.124257 -0.793684
H -3.190476 3.294793 -0.065525
H -3.586398 2.416944 1.441461
H -5.53672 3.016939 0.219992
C 2.331465 -0.308321 -1.56958
O 3.333711 0.662845 -1.574703
C 3.756949 1.266996 -0.345369
C 2.586605 1.649654 0.575044
O 2.048788 2.835489 0.020982
C 1.461724 0.599348 0.638676
C 1.116565 0.215163 -0.803977
O 0.139819 -0.853895 -0.723702
C 4.897525 0.524722 0.336276
O 4.432442 -0.592955 1.08747
O 2.705972 -1.512141 -0.967715
H 4.184495 2.218543 -0.676154
H 2.971089 1.809954 1.591691
H 0.679218 1.073645 -1.320542
H 2.087745 -0.454521 -2.628748
H 3.46522 -1.880199 -1.438232
H 2.686307 -0.784786 1.305485
H 1.418846 3.236435 0.632746
H 5.399973 1.237326 1.001167
H 5.60561 0.201858 -0.434912
H 5.126331 -0.909016 1.677072
H 0.580634 1.075932 1.078943
H -1.270201 0.097983 -1.868832
Na 0.065735 -2.084814 1.443095

Reactant state of 2nd c0',3' reaction of Manβ(1→2)Manβ
46

C -1.242098 -0.377653 -1.257666
O -1.57152 0.799042 -0.537856
C -2.980221 0.961442 -0.38508
C -3.495234 -0.040896 0.66607
O -4.810339 -0.442185 0.377254
C -2.62064 -1.300205 0.632534
C -2.144393 -1.557543 -0.798224
O -1.379035 -2.753725 -0.867435
O -1.503238 -1.232929 1.512698
C -3.253227 2.397274 0.020108
O -4.637091 2.428729 0.322565
O 1.193994 0.017175 1.402548
H -3.49176 0.757895 -1.338709
H -3.422075 0.399957 1.672271
H -3.231801 -2.141338 0.972417
H -3.019126 -1.619156 -1.456069
H -1.969826 -3.502914 -1.00708
H -1.108476 -0.346937 1.487832
H -5.37219 0.346511 0.397366
H -2.996775 3.080793 -0.796922
H -2.644809 2.64511 0.901881
H -4.920829 3.322621 0.54196
C 2.365474 -0.595858 -1.360886
O 3.521467 0.175561 -1.327856
C 3.790781 1.035844 -0.215516
C 2.550271 1.782743 0.295024
O 2.355973 2.853339 -0.612223
C 1.265862 0.936545 0.323724
C 1.1115 0.213063 -1.019756
O 0.063439 -0.739698 -0.94771
C 4.630796 0.375615 0.868855
O 3.842233 -0.435182 1.740738
O 2.427742 -1.691218 -0.454452
H 4.434754 1.806752 -0.650183
H 2.757341 2.150094 1.310512
H 0.941623 0.943283 -1.819619
H 2.305206 -0.974521 -2.385782
H 3.251014 -2.168402 -0.621407
H 2.104294 -0.216366 1.677469
H 1.752564 3.504053 -0.234565
H 5.112798 1.175469 1.443063
H 5.406242 -0.228719 0.385641
H 4.341046 -0.635218 2.541251
H 0.422332 1.622722 0.442024
H -1.337557 -0.171767 -2.335339
Na 0.434891 -2.233037 0.671881

Transition state of 3rd c0',3' reaction of Manβ(1→2)Manβ
46

C -1.036009 -0.700468 -1.186479
O -1.628331 1.098346 0.211095
C -2.991424 1.146015 -0.171363
C -3.733499 -0.137356 0.266505
O -4.639633 -0.57143 -0.722145
C -2.750711 -1.272275 0.55063
C -1.977315 -1.74749 -0.834358
O -1.244106 -2.910023 -0.486951
O -1.841495 -0.973193 1.496361
C -3.690978 2.382726 0.366434
O -5.037541 2.252253 -0.069798
O 1.751585 -0.505187 1.447623
H -3.045914 1.189171 -1.272091
H -4.249887 0.046786 1.219645
H -3.33093 -2.193876 0.745321
H -2.727757 -1.901542 -1.614861
H -1.862965 -3.648348 -0.418109
H -1.584088 0.391542 0.969754
H -5.318594 0.112676 -0.815832
H -3.22009 3.289467 -0.02973
H -3.622614 2.391678 1.462741
H -5.567875 2.995023 0.23711
C 2.320859 -0.285506 -1.568997
O 3.343109 0.668365 -1.572533
C 3.785017 1.240766 -0.337743
C 2.616632 1.654724 0.579415
O 1.975416 2.804276 0.0614
C 1.475497 0.626197 0.65053
C 1.116115 0.245365 -0.79008
O 0.140958 -0.824204 -0.70668
C 4.909578 0.464273 0.337968
O 4.424922 -0.649661 1.078639
O 2.684147 -1.498463 -0.980267
H 4.261325 2.172415 -0.666332
H 2.992763 1.819515 1.598658
H 0.674974 1.107875 -1.296928
H 2.069942 -0.417476 -2.628232
H 3.426238 -1.880054 -1.466917
H 2.679363 -0.785638 1.303341
H 2.556063 3.572488 0.109969
H 5.427215 1.160602 1.00918
H 5.611644 0.135879 -0.436733
H 5.111324 -0.98091 1.668755
H 0.615717 1.134679 1.093337
H -1.269094 0.119584 -1.860653
Na 0.061673 -2.060181 1.441807

Reactant state of 3rd c0',3' reaction of Manβ(1→2)Manβ
46

C 1.241787 -0.380356 1.259599
O 1.564562 0.79302 0.535724
C 2.97138 0.964254 0.382214
C 3.491313 -0.038586 -0.665935
O 4.808334 -0.433649 -0.376305
C 2.621129 -1.301019 -0.630797
C 2.144919 -1.559373 0.799769
O 1.381087 -2.757066 0.86897
O 1.5027 -1.236969 -1.510271
C 3.23331 2.400422 -0.028529
O 4.617695 2.439757 -0.331619
O -1.196564 0.000418 -1.403027
H 3.484935 0.767058 1.336252
H 3.41687 0.399761 -1.673099
H 3.234691 -2.140517 -0.970653
H 3.019516 -1.62004 1.457949
H 1.974701 -3.505924 0.998081
H 1.110956 -0.349427 -1.490693
H 5.365266 0.3586 -0.396247
H 2.971892 3.085294 0.785413
H 2.622442 2.641087 -0.910166
H 4.892527 3.333889 -0.560931
C -2.366763 -0.581186 1.366514
O -3.522551 0.196266 1.322175
C -3.781405 1.042219 0.19954
C -2.529981 1.786401 -0.302336
O -2.188914 2.833491 0.587026
C -1.256287 0.92942 -0.332416
C -1.107833 0.216892 1.018004
O -0.066351 -0.744314 0.957601
C -4.621275 0.377952 -0.885542
O -3.839345 -0.454102 -1.739704
O -2.435213 -1.684826 0.472958
H -4.448021 1.80431 0.622297
H -2.722347 2.167612 -1.316115
H -0.931725 0.956449 1.807607
H -2.313037 -0.947057 2.396282
H -3.248674 -2.171023 0.660294
H -2.10727 -0.234431 -1.67263
H -2.887404 3.497478 0.616617
H -5.08783 1.177519 -1.473651
H -5.409686 -0.208186 -0.400524
H -4.335679 -0.660088 -2.540325
H -0.42004 1.61941 -0.454912
H 1.341702 -0.172385 2.336582
Na -0.433551 -2.232532 -0.664198

Transition state of 1st RO2' reaction of Manβ(1→2)Manβ
46

C 1.227516 -1.566972 0.96238
O 0.999614 0.960754 -0.63253
C 1.960398 1.002245 0.332222
C 3.299592 0.4645 -0.171256
O 4.184312 0.464625 0.932257
C 3.114445 -0.964977 -0.690921
C 2.584614 -1.945361 0.405383
O 2.448929 -3.219975 -0.162242
O 2.299762 -1.052189 -1.826031
C 2.015559 2.35468 1.089332
O 1.590344 3.43604 0.25948
O -0.841421 0.894195 0.90368
H 1.712771 0.253375 1.174378
H 3.662386 1.092438 -0.997364
H 4.093497 -1.383756 -0.958443
H 3.292067 -1.993228 1.236721
H 2.183041 -3.091158 -1.08866
H 1.568695 -0.407639 -1.696439
H 5.099465 0.388941 0.637568
H 3.015537 2.53015 1.495199
H 1.303116 2.332076 1.917093
H 2.31748 3.736521 -0.29716
C -1.850484 -1.533096 -0.908773
O -3.198712 -1.21328 -0.807764
C -3.603407 -0.073856 -0.036285
C -2.972773 -0.112187 1.363076
O -3.426568 -1.243809 2.078738
C -1.448027 -0.267889 1.337384
C -1.152049 -1.495844 0.465283
O 0.270514 -1.628752 0.13929
C -3.580957 1.237335 -0.838032
O -2.332125 1.698859 -1.326324
O -1.154079 -0.692725 -1.806909
H -4.675505 -0.254201 0.118825
H -3.199272 0.82458 1.889921
H -1.442603 -2.394956 1.015219
H -1.827807 -2.558979 -1.287017
H -1.223125 -1.054703 -2.699968
H -0.092729 0.761137 0.158578
H -4.375227 -1.195408 2.245001
H -3.990336 2.028006 -0.201845
H -4.266657 1.098227 -1.682173
H -1.88126 0.938751 -1.738389
H -1.146746 -0.503925 2.37083
H 1.014108 -1.453996 2.029364
Na -0.49382 2.771484 -0.501196

Reactant state of 1st RO2' reaction of Manβ(1→2)Manβ
46

C -1.422515 -1.2351 -0.488938
O -1.569477 -0.152916 -1.380401
C -2.565034 0.887732 -1.238352
C -3.700955 0.611963 -0.233938
O -4.743326 0.003025 -0.967429
C -3.260793 -0.294574 0.919343
C -2.686602 -1.564521 0.303255
O -2.437902 -2.592818 1.222969
O -2.347093 0.431694 1.742719
C -1.861084 2.222772 -1.015134
O -1.096438 2.275809 0.181268
O 1.119418 0.728704 -1.205102
H -3.054311 0.948317 -2.216314
H -4.027115 1.576381 0.181151
H -4.130233 -0.564929 1.529636
H -3.438169 -1.943113 -0.39481
H -1.658345 -2.376427 1.749712
H -1.498479 -0.035396 1.795851
H -5.565495 0.018099 -0.462176
H -2.613629 3.020766 -1.030097
H -1.172089 2.410808 -1.846057
H -1.604956 1.831567 0.892359
C 1.783936 -0.854033 1.343931
O 3.155805 -0.75879 1.070485
C 3.682554 -0.402494 -0.211498
C 2.847599 -0.949379 -1.370432
O 2.92471 -2.357626 -1.440212
C 1.363642 -0.667825 -1.179373
C 0.92303 -1.291658 0.142628
O -0.417297 -0.963156 0.47709
C 3.997259 1.11941 -0.323855
O 3.355654 1.903126 0.669885
O 1.26289 0.358433 1.878123
H 4.63862 -0.936057 -0.241881
H 3.184259 -0.47867 -2.303835
H 1.032773 -2.3788 0.044696
H 1.715342 -1.617411 2.124522
H 1.509544 0.403648 2.810285
H 0.163613 0.795368 -1.379625
H 3.793401 -2.639132 -1.750044
H 3.6563 1.510056 -1.285047
H 5.078656 1.272696 -0.260739
H 3.641613 1.572952 1.531485
H 0.827033 -1.149677 -2.004821
H -1.116285 -2.094005 -1.09695
Na 1.125107 2.214121 0.521063

Transition state of 2nd RO2' reaction of Manβ(1→2)Manβ
46

C 1.148108 -1.574377 0.963709
O 0.870905 0.956349 -0.441632
C 1.878462 0.858403 0.424179
C 3.181308 0.352396 -0.194695
O 4.148236 0.340117 0.835984
C 2.979687 -1.068394 -0.73755
C 2.480701 -2.026049 0.379411
O 2.297304 -3.311689 -0.141404
O 2.158405 -1.149994 -1.870462
C 1.984417 2.069442 1.36367
O 1.811152 3.226324 0.524435
O -0.907511 0.750922 1.278622
H 1.629538 -0.05603 1.190364
H 3.457354 1.022622 -1.019847
H 3.955084 -1.468018 -1.041295
H 3.21471 -2.069467 1.187851
H 1.990362 -3.211468 -1.058128
H 1.330425 -0.654961 -1.723945
H 5.031779 0.213596 0.470081
H 2.95689 2.086727 1.858915
H 1.178196 2.033987 2.103103
H 2.219815 3.988855 0.949459
C -1.803762 -1.309725 -1.117558
O -3.167455 -0.999681 -1.052602
C -3.588446 0.060183 -0.205655
C -3.137222 -0.202938 1.24445
O -3.714953 -1.388448 1.741823
C -1.624453 -0.453353 1.370825
C -1.228389 -1.496824 0.317024
O 0.207476 -1.559307 0.092238
C -3.348672 1.471778 -0.771436
O -2.000856 1.955614 -0.69523
O -1.066948 -0.435016 -1.905793
H -4.682799 -0.03299 -0.212529
H -3.383785 0.665704 1.873921
H -1.545846 -2.479286 0.677742
H -1.753316 -2.273777 -1.62712
H -1.156491 0.468869 -1.56322
H -0.206118 0.748622 0.521505
H -4.676871 -1.364201 1.674787
H -4.007661 2.167286 -0.238443
H -3.628702 1.463234 -1.826642
H -1.69782 1.750422 0.222837
H -1.467608 -0.879654 2.369847
H 0.898855 -1.657503 2.02599
Na 0.157049 2.942968 -1.087392

Reactant state of 2nd RO2' reaction of Manβ(1→2)Manβ
46

C 1.18065 0.755685 -1.061234
O 1.827602 -0.450087 -1.383256
C 3.138137 -0.662471 -0.831664
C 3.23657 -0.056766 0.568846
O 4.507547 -0.399333 1.063629
C 2.99521 1.460519 0.53218
C 2.160435 1.863711 -0.704054
O 1.433566 3.042595 -0.496626
O 2.387934 1.913902 1.728397
C 3.359623 -2.160822 -0.829286
O 2.35036 -2.762448 -0.005243
O -1.608856 -1.166752 -1.222427
H 3.896318 -0.207095 -1.483507
H 2.44787 -0.510209 1.190211
H 3.964755 1.966203 0.485921
H 2.83446 1.996847 -1.561081
H 1.575788 3.333163 0.416657
H 1.556801 1.42943 1.840028
H 4.632448 -0.007905 1.937694
H 4.355395 -2.364594 -0.429121
H 3.277779 -2.545612 -1.850557
H 2.689501 -3.580455 0.373301
C -1.600098 0.438422 1.358941
O -2.96558 0.688835 1.470176
C -3.865547 0.389982 0.39926
C -3.273371 0.649758 -1.000702
O -3.255717 2.034872 -1.281061
C -1.80077 0.233928 -1.14546
C -1.024109 0.874473 0.012701
O 0.346386 0.478156 0.064817
C -4.556693 -0.95388 0.578731
O -3.674988 -2.036792 0.288113
O -1.284289 -0.93725 1.528943
H -4.665704 1.12847 0.532389
H -3.861678 0.094527 -1.745451
H -1.104843 1.964028 -0.068869
H -1.149499 1.017372 2.17296
H -1.761495 -1.2608 2.304395
H -2.321417 -1.614473 -0.720584
H -4.147653 2.367829 -1.433029
H -5.421556 -0.979307 -0.095365
H -4.911005 -1.023765 1.612692
H -4.175796 -2.857766 0.214602
H -1.467753 0.654292 -2.097744
H 0.570445 1.044184 -1.919917
Na 0.270444 -1.844107 -0.089898

Transition state of 3rd RO2' reaction of Manβ(1→2)Manβ
46

C -1.028732 -1.033804 -1.241846
O -1.091621 0.868403 0.871089
C -2.124898 1.22614 0.048277
C -3.377469 0.364447 0.289624
O -4.335184 0.498229 -0.737779
C -3.103041 -1.140589 0.295137
C -2.375973 -1.65874 -0.967218
O -2.186588 -3.045911 -0.903769
O -2.402269 -1.631109 1.445445
C -2.424199 2.719838 0.194954
O -3.483643 3.07117 -0.688011
O 1.153529 1.407178 -0.021297
H -1.882324 1.076843 -1.043093
H -3.795778 0.626339 1.276454
H -4.080808 -1.632316 0.240082
H -3.018601 -1.432775 -1.822481
H -1.903943 -3.273724 -0.005698
H -3.033972 -2.081737 2.018558
H -4.44711 1.444548 -0.927671
H -1.50905 3.277076 -0.035553
H -2.698874 2.907404 1.241702
H -3.700994 4.005883 -0.605738
C 2.153046 -1.392164 0.379809
O 3.497192 -1.129755 0.126109
C 3.902013 0.164884 -0.339843
C 2.96069 0.750237 -1.413549
O 3.136228 0.065312 -2.641328
C 1.467813 0.58891 -1.080495
C 1.326227 -0.915068 -0.817615
O -0.06648 -1.341967 -0.507243
C 4.329821 1.062388 0.81967
O 3.293534 1.253963 1.771809
O 1.681606 -0.853612 1.590208
H 4.838987 -0.040939 -0.872508
H 3.153561 1.82534 -1.512296
H 1.609829 -1.481521 -1.708791
H 2.079096 -2.479616 0.470348
H 2.124484 0.024858 1.712626
H 0.185257 1.243705 0.342592
H 3.969507 0.310939 -3.058967
H 4.654029 2.027565 0.410944
H 5.186079 0.574393 1.297228
H 3.672679 1.574243 2.597965
H 0.92376 0.848889 -2.006408
H -0.843262 -0.411821 -2.121878
Na -0.480435 -0.604333 2.242663

Reactant state of 3rd RO2' reaction of Manβ(1→2)Manβ
46

C 1.104716 -0.516084 1.048072
O 1.402678 0.656435 0.298398
C 2.772852 1.04263 0.398171
C 3.645132 0.061593 -0.407142
O 4.932667 -0.038623 0.143252
C 3.022931 -1.341797 -0.359746
C 2.263718 -1.542234 0.949445
O 1.805544 -2.848245 1.131468
O 2.109573 -1.536462 -1.457742
C 2.891056 2.465464 -0.115724
O 4.283498 2.721139 -0.172674
O -1.271654 1.22625 -0.784848
H 3.098692 1.014623 1.448725
H 3.681432 0.383939 -1.459844
H 3.828626 -2.078947 -0.411706
H 2.975858 -1.345742 1.75833
H 1.06047 -2.99207 0.53272
H 2.514868 -2.127327 -2.101724
H 5.329609 0.844997 0.137201
H 2.373962 3.157737 0.557639
H 2.431829 2.529648 -1.112853
H 4.455281 3.640904 -0.400918
C -2.314479 -1.467007 0.122233
O -3.62965 -1.09798 0.402146
C -4.029109 0.274892 0.455013
C -2.951099 1.225578 1.020355
O -2.88865 1.123543 2.429957
C -1.523271 0.886473 0.566646
C -1.301069 -0.601923 0.865001
O -0.025522 -1.083529 0.449664
C -4.688529 0.726113 -0.839839
O -3.726485 0.886022 -1.87872
O -2.000424 -1.383013 -1.260496
H -4.843903 0.271924 1.189844
H -3.180774 2.252441 0.701871
H -1.43167 -0.763884 1.941443
H -2.243084 -2.508882 0.450175
H -2.690701 -1.842125 -1.756617
H -2.099811 1.132145 -1.299001
H -3.679198 1.496163 2.837085
H -5.201291 1.676356 -0.64566
H -5.43101 -0.026237 -1.126298
H -4.128885 1.32516 -2.637082
H -0.8406 1.483476 1.176978
H 0.886493 -0.246156 2.092234
Na 0.167647 -0.324057 -1.680972

Transition state of 1st RO reaction of Manβ(1→3)Manβ
46

C 1.410855 -0.289329 1.486804
O 1.138373 1.083723 1.609149
C 1.734255 1.982571 0.645165
C 1.876812 1.26224 -0.695174
O 2.334785 2.114365 -1.715727
C 2.867049 0.115849 -0.576709
C 2.751583 -0.577182 0.808947
O 2.836532 -1.985876 0.713136
O 2.631041 -0.846964 -1.599417
C 0.855498 3.225993 0.50622
O -0.112241 3.068283 -0.51184
O 0.449582 -0.95002 0.70425
H 2.719253 2.286857 1.027456
H 0.902285 0.836311 -0.959563
H 3.880584 0.521964 -0.692304
H 3.534009 -0.181317 1.4673
H 1.394037 -0.69647 2.505711
H 3.737986 -2.241308 0.479504
H 2.732713 -0.392568 -2.447918
H 1.59041 2.666004 -1.998544
H 1.49646 4.063809 0.216725
H 0.407311 3.470478 1.476278
H -0.99814 2.862167 -0.178356
C -2.030061 -2.036791 -0.818348
O -2.910101 -0.770461 -0.924992
C -3.010142 0.107362 0.231331
C -1.595684 0.348463 0.750819
O -1.691321 1.305335 1.775078
C -0.883939 -0.950762 1.185125
C -1.572574 -2.216404 0.628821
O -0.680418 -3.312488 0.54316
C -3.678165 1.393347 -0.21317
O -2.748436 2.084902 -1.030082
O -1.090195 -1.62089 -1.675843
H -3.649958 -0.396336 0.962826
H -1.016364 0.737786 -0.096755
H -2.435124 -2.462978 1.261356
H -2.677007 -2.859007 -1.148523
H -2.038796 -0.585371 -1.625795
H -0.5037 -3.683817 1.416248
H -0.790015 1.48431 2.08789
H -3.928538 1.973493 0.681137
H -4.593009 1.147033 -0.762327
H -3.197726 2.755216 -1.557404
H -0.875778 -1.001357 2.28499
Na 0.972306 -2.350573 -1.012172

Reactant state of 1st RO reaction of Manβ(1→3)Manβ
46

C -1.132639 0.074734 -1.543238
O -0.595001 1.37261 -1.475401
C -1.075709 2.287804 -0.471953
C -1.765458 1.519921 0.653107
O -2.264264 2.380352 1.654051
C -2.952924 0.734475 0.114842
C -2.636957 0.106629 -1.274258
O -3.095942 -1.228667 -1.377075
O -3.302145 -0.305466 1.0234
C 0.106509 3.101129 0.046517
O 0.808059 2.370111 1.038781
O -0.592053 -0.798944 -0.584952
H -1.790851 2.973604 -0.948672
H -1.047521 0.809877 1.074457
H -3.801494 1.424159 0.015955
H -3.083395 0.731036 -2.056004
H -0.914229 -0.292206 -2.55202
H -4.051072 -1.238874 -1.515689
H -3.523222 0.113319 1.867
H -1.529823 2.686935 2.20239
H -0.273216 4.033545 0.480517
H 0.750364 3.362604 -0.800806
H 1.755899 2.311893 0.838259
C 0.203021 -1.673208 1.683048
O 3.43463 -1.318333 0.702885
C 3.250983 -0.627743 -0.514121
C 1.786703 -0.238093 -0.778181
O 1.793132 0.320698 -2.080015
C 0.737637 -1.357741 -0.734465
C 0.769633 -2.34939 0.438886
O -0.146406 -3.397974 0.095039
C 4.098915 0.639007 -0.522281
O 3.597686 1.479821 0.533551
O 1.075089 -0.833472 2.154229
H 3.597979 -1.288833 -1.316179
H 1.485877 0.501434 -0.030649
H 1.78325 -2.727494 0.600408
H 3.314405 -0.681683 1.421821
H 0.712137 -0.371745 2.92659
H 0.208677 -4.252875 0.360952
H 1.136392 1.03006 -2.115674
H 4.025277 1.149541 -1.484657
H 5.137236 0.362424 -0.317189
H 4.303142 2.061816 0.837233
H 0.784894 -1.92748 -1.671888
Na -2.092844 -2.21837 0.556409

Transition state of 2nd RO reaction of Manβ(1→3)Manβ
46

C 1.410886 -0.289339 1.486826
O 1.138448 1.083729 1.609154
C 1.734333 1.982548 0.645145
C 1.876802 1.262193 -0.695189
O 2.334717 2.114287 -1.715794
C 2.867022 0.115793 -0.57674
C 2.751582 -0.577232 0.808925
O 2.836495 -1.985927 0.713111
O 2.63096 -0.847016 -1.599439
C 0.855616 3.225994 0.506212
O -0.112239 3.068247 -0.511728
O 0.449577 -0.950029 0.704322
H 2.719356 2.286802 1.027397
H 0.902254 0.836269 -0.95951
H 3.880565 0.521881 -0.692368
H 3.534043 -0.181381 1.467246
H 1.394099 -0.696451 2.505744
H 3.73794 -2.241378 0.479463
H 2.732643 -0.392626 -2.447942
H 1.590332 2.665937 -1.998569
H 1.496582 4.063761 0.216585
H 0.407546 3.470573 1.476301
H -0.998086 2.862116 -0.178115
C -2.030116 -2.036793 -0.818212
O -2.91018 -0.770496 -0.92489
C -3.01015 0.107405 0.231382
C -1.595652 0.348512 0.750764
O -1.691173 1.305539 1.774892
C -0.883942 -0.950693 1.185188
C -1.572589 -2.216362 0.628949
O -0.680435 -3.312442 0.543273
C -3.678157 1.393374 -0.213189
O -2.74843 2.08483 -1.030182
O -1.090271 -1.620903 -1.675743
H -3.649941 -0.396229 0.962942
H -1.016368 0.737696 -0.096897
H -2.43512 -2.462925 1.261516
H -2.677048 -2.859036 -1.148348
H -2.038909 -0.585422 -1.625738
H -0.503553 -3.683644 1.416383
H -0.789848 1.48441 2.087716
H -3.928479 1.973595 0.681083
H -4.59303 1.147045 -0.762292
H -3.197701 2.755148 -1.557515
H -0.875799 -1.001207 2.285058
Na 0.972268 -2.350536 -1.012392

Reactant state of 2nd RO reaction of Manβ(1→3)Manβ
46

C -1.132144 0.074667 -1.543345
O -0.594188 1.37242 -1.475163
C -1.075024 2.287669 -0.471869
C -1.76555 1.51996 0.652821
O -2.264636 2.380503 1.653552
C -2.952974 0.734813 0.114051
C -2.636565 0.106821 -1.274895
O -3.095618 -1.228458 -1.377699
O -3.302821 -0.304952 1.022548
C 0.107263 3.100506 0.047225
O 0.808279 2.369046 1.039549
O -0.592193 -0.799228 -0.58494
H -1.789704 2.973793 -0.948822
H -1.047996 0.809727 1.074506
H -3.801327 1.424702 0.014738
H -3.082715 0.731189 -2.056827
H -0.913447 -0.292184 -2.552092
H -4.050525 -1.238667 -1.517777
H -3.523971 0.113995 1.866051
H -1.530417 2.686823 2.202324
H -0.272298 4.03296 0.481292
H 0.751494 3.361957 -0.799825
H 1.756171 2.310816 0.839276
C 0.20263 -1.671996 1.683704
O 3.434298 -1.317804 0.703684
C 3.250899 -0.628236 -0.513921
C 1.78667 -0.238684 -0.77844
O 1.792987 0.319009 -2.080737
C 0.737525 -1.358223 -0.733894
C 0.769431 -2.349087 0.440091
O -0.146504 -3.397906 0.096629
C 4.098917 0.638459 -0.522984
O 3.597969 1.479911 0.532473
O 1.074533 -0.831764 2.154293
H 3.597937 -1.290014 -1.315394
H 1.485852 0.501467 -0.031547
H 1.783035 -2.727089 0.601916
H 3.313822 -0.680633 1.422096
H 0.711368 -0.369272 2.926093
H 0.207608 -4.252376 0.365244
H 1.137211 1.029246 -2.116534
H 4.025078 1.148397 -1.48566
H 5.137269 0.361958 -0.317915
H 4.303151 2.06308 0.834541
H 0.784769 -1.928607 -1.670924
Na -2.093243 -2.217954 0.556407

Transition state of 3rd RO reaction of Manβ(1→3)Manβ
46

C -1.289519 0.071901 1.379202
O -1.211812 -1.300836 1.100234
C -1.781097 -1.863141 -0.098164
C -3.028074 -1.138989 -0.655257
O -4.129765 -1.856527 -0.121945
C -3.194574 0.337882 -0.264629
C -2.702275 0.601435 1.153575
O -2.707487 1.999367 1.390552
O -2.543057 1.200594 -1.189007
C -0.716009 -2.164504 -1.137559
O -0.278304 -0.983956 -1.791668
O -0.382144 0.837714 0.627559
H -2.156461 -2.836719 0.231332
H -3.005574 -1.198628 -1.751501
H -4.271467 0.550561 -0.299723
H -3.354517 0.060918 1.849111
H -1.029686 0.158542 2.441934
H -3.392497 2.233356 2.026481
H -2.753527 2.098917 -0.89134
H -4.927632 -1.675579 -0.633655
H -1.145165 -2.862335 -1.868039
H 0.124104 -2.663998 -0.638904
H 0.636187 -1.085066 -2.091652
C 2.516441 1.587388 -0.789218
O 3.466882 0.463449 -0.36812
C 3.164975 -0.338807 0.809689
C 1.650118 -0.462043 0.983746
O 1.443113 -1.280891 2.108887
C 0.946754 0.894266 1.134144
C 1.671235 2.047739 0.395412
O 0.761351 2.958987 -0.192735
C 3.791343 -1.693191 0.530035
O 3.131676 -2.19445 -0.625849
O 1.891784 0.88396 -1.7522
H 3.641438 0.136537 1.67257
H 1.25284 -0.924272 0.076978
H 2.32891 2.562381 1.108858
H 3.181792 2.387921 -1.134702
H 2.906366 0.058013 -1.277392
H 0.148631 3.28509 0.480826
H 0.58944 -1.727663 2.00323
H 3.629891 -2.338439 1.398458
H 4.863923 -1.564021 0.350838
H 3.623708 -2.940065 -0.986765
H 0.903229 1.131363 2.207304
Na -0.318886 1.277662 -1.673252

Reactant state of 3rd RO reaction of Manβ(1→3)Manβ
46

C -1.350376 0.297464 1.370338
O -1.332417 -1.103661 1.27674
C -1.882065 -1.801255 0.144493
C -3.042468 -1.096862 -0.596164
O -4.216288 -1.696511 -0.073567
C -3.152434 0.422409 -0.403808
C -2.721363 0.854443 0.993521
O -2.665596 2.270454 1.032633
O -2.403853 1.115452 -1.395805
C -0.790589 -2.350443 -0.758817
O -0.165018 -1.347662 -1.553733
O -0.374812 0.914601 0.573937
H -2.34949 -2.684664 0.591124
H -2.94309 -1.291822 -1.672644
H -4.211857 0.68167 -0.531495
H -3.438394 0.447177 1.715552
H -1.137832 0.511243 2.425296
H -3.356817 2.623889 1.60332
H -2.555718 2.05732 -1.222003
H -4.967475 -1.52911 -0.655917
H -1.243319 -3.108398 -1.410088
H -0.044239 -2.845706 -0.125189
H 0.765656 -1.594941 -1.667989
C 2.758536 1.382928 -0.633622
O 3.642551 0.61068 0.107697
C 3.108955 -0.44031 0.920935
C 1.586729 -0.417611 1.091305
O 1.325077 -1.135184 2.277653
C 0.947482 0.980783 1.113499
C 1.716538 2.035906 0.264696
O 0.858401 2.773237 -0.589302
C 3.548189 -1.774458 0.336978
O 2.917469 -1.953747 -0.945373
O 2.073112 0.607371 -1.606449
H 3.564654 -0.337159 1.911628
H 1.1394 -0.922458 0.236256
H 2.259706 2.707369 0.938917
H 3.367947 2.1447 -1.127497
H 2.643749 -0.151419 -1.818761
H 0.197021 3.231652 -0.05317
H 0.431222 -1.504187 2.218589
H 3.25004 -2.577694 1.018796
H 4.635438 -1.768023 0.217704
H 3.346833 -2.690302 -1.396602
H 0.886016 1.317543 2.156977
Na -0.170116 0.935011 -1.749853

Transition state of 1st m1_2 reaction of Manβ(1→3)Manβ
46

C -1.158356 -1.181893 -0.956457
O -1.824778 -0.312159 -1.813988
C -2.668212 0.719192 -1.278934
C -3.496198 0.283757 -0.054625
O -4.603005 -0.431467 -0.576163
C -2.740294 -0.635584 0.924134
C -2.077477 -1.755096 0.118257
O -1.274794 -2.580984 0.953973
O -1.809712 0.042401 1.744699
C -1.944489 2.051263 -1.122778
O -1.207885 2.123975 0.090557
O -0.09027 -0.551374 -0.284265
H -3.401787 0.877428 -2.075371
H -3.818036 1.186652 0.482576
H -3.47932 -1.08336 1.600167
H -2.854719 -2.333973 -0.391347
H -0.774269 -1.987295 -1.595467
H -1.787375 -3.33318 1.270672
H -1.459346 0.806199 1.245279
H -5.315296 -0.462884 0.073382
H -2.698292 2.848255 -1.14473
H -1.2828 2.173462 -1.989023
H -0.578038 2.861937 0.081371
C 3.26856 -1.867403 -1.147782
O 1.14801 1.014696 1.641222
C 2.135038 1.321571 0.669567
C 1.725853 0.952508 -0.771374
O 2.921269 1.021899 -1.535614
C 1.137826 -0.455505 -0.96471
C 2.088906 -1.516257 -0.402005
O 2.163295 -1.709131 0.877433
C 2.474346 2.802172 0.754125
O 1.240404 3.512419 0.573039
O 4.204109 -2.447529 -0.478741
H 3.023158 0.733482 0.921933
H 0.97427 1.662035 -1.135589
H 1.843501 -2.548163 -1.065858
H 3.397911 -1.762489 -2.221014
H 0.533124 1.761266 1.678248
H 3.907667 -2.391216 0.470865
H 2.777792 1.507514 -2.355108
H 3.197491 3.072278 -0.020849
H 2.890269 3.020621 1.742669
H 1.360341 4.440974 0.802448
H 0.995276 -0.594675 -2.046518
Na 0.285664 -1.111743 1.957232

Reactant state of 1st m1_2 reaction of Manβ(1→3)Manβ
46

C -1.244314 -1.136648 -0.981864
O -1.830314 -0.186771 -1.812675
C -2.613295 0.882799 -1.261681
C -3.487077 0.468805 -0.062532
O -4.624986 -0.168235 -0.616496
C -2.801761 -0.517261 0.902297
C -2.209535 -1.662102 0.077834
O -1.471295 -2.557305 0.904928
O -1.83209 0.08265 1.739749
C -1.804435 2.157117 -1.054781
O -1.081131 2.134837 0.168104
O -0.120871 -0.630348 -0.293177
H -3.321506 1.1114 -2.063998
H -3.763902 1.376163 0.491891
H -3.573638 -0.928367 1.564727
H -3.020949 -2.178145 -0.445331
H -0.934001 -1.951421 -1.648081
H -2.035161 -3.281751 1.198432
H -1.450741 0.853 1.269561
H -5.355775 -0.153675 0.012731
H -2.501069 3.004717 -1.056784
H -1.124442 2.262849 -1.908744
H -0.384594 2.810398 0.189667
C 3.366868 -1.768719 -1.162219
O 1.265676 0.808597 1.665683
C 2.238107 1.095947 0.669696
C 1.756065 0.794396 -0.763336
O 2.926279 0.862823 -1.564914
C 1.10151 -0.572308 -1.003789
C 1.952438 -1.78631 -0.595167
O 2.015731 -1.882294 0.810081
C 2.65296 2.555448 0.787648
O 1.446584 3.32877 0.700636
O 4.307723 -1.86575 -0.411643
H 3.112756 0.469062 0.870224
H 1.021951 1.551465 -1.062285
H 1.456782 -2.684259 -1.000437
H 3.486439 -1.7186 -2.255293
H 0.729252 1.606546 1.781464
H 2.949275 -2.042642 1.040404
H 2.736256 1.266769 -2.419154
H 3.345664 2.817118 -0.016669
H 3.130315 2.718475 1.759032
H 1.626443 4.244759 0.941641
H 0.908518 -0.635377 -2.085429
Na 0.136955 -1.181932 1.927962

Transition state of 2nd m1_2 reaction of Manβ(1→3)Manβ
46

C -1.165359 -1.183161 -0.955325
O -1.833892 -0.320742 -1.819496
C -2.663737 0.722335 -1.284987
C -3.489369 0.300355 -0.054339
O -4.604231 -0.40496 -0.570934
C -2.738553 -0.619707 0.926571
C -2.086719 -1.747329 0.128663
O -1.339141 -2.550674 1.03164
O -1.80063 0.056669 1.74115
C -1.927259 2.049083 -1.139549
O -1.191461 2.126608 0.074255
O -0.096734 -0.546885 -0.290996
H -3.399643 0.883337 -2.078645
H -3.800863 1.20848 0.48043
H -3.47242 -1.06655 1.606777
H -2.877772 -2.321226 -0.364863
H -0.779631 -1.989494 -1.594889
H -1.381974 -3.480578 0.785066
H -1.445596 0.813868 1.235822
H -5.298337 -0.464812 0.09631
H -2.674086 2.852238 -1.169469
H -1.263572 2.157768 -2.006265
H -0.573485 2.874219 0.07045
C 3.275424 -1.844844 -1.149401
O 1.150447 0.999199 1.644238
C 2.139707 1.304801 0.675165
C 1.726793 0.950872 -0.768252
O 2.920909 1.017308 -1.535345
C 1.129607 -0.451795 -0.972557
C 2.076191 -1.523692 -0.419737
O 2.125083 -1.75302 0.85483
C 2.491699 2.78192 0.771445
O 1.264044 3.502912 0.592648
O 4.201449 -2.434324 -0.475601
H 3.023212 0.70736 0.923043
H 0.979463 1.667795 -1.125477
H 1.854971 -2.538799 -1.118661
H 3.426768 -1.709101 -2.216186
H 0.539139 1.748868 1.682758
H 3.884262 -2.405755 0.46863
H 2.786136 1.533053 -2.337677
H 3.219311 3.05211 0.000597
H 2.907174 2.989078 1.762614
H 1.389816 4.427759 0.833689
H 0.985658 -0.579883 -2.055511
Na 0.2722 -1.120861 1.964015

Reactant state of 2nd m1_2 reaction of Manβ(1→3)Manβ
46

C -1.243941 -1.137095 -0.981662
O -1.829585 -0.186987 -1.812464
C -2.613482 0.882161 -1.261951
C -3.487197 0.468158 -0.062709
O -4.625027 -0.169208 -0.616468
C -2.801582 -0.517603 0.902214
C -2.209355 -1.662463 0.077774
O -1.470722 -2.558256 0.903906
O -1.831994 0.082599 1.739551
C -1.805321 2.156897 -1.055153
O -1.08151 2.134352 0.167411
O -0.120542 -0.630983 -0.292793
H -3.321737 1.1101 -2.064419
H -3.764137 1.375533 0.491601
H -3.573421 -0.928591 1.564813
H -3.020614 -2.178471 -0.445724
H -0.933683 -1.951875 -1.647867
H -2.03913 -3.274101 1.209701
H -1.44982 0.852161 1.26861
H -5.356057 -0.153929 0.012456
H -2.502442 3.004102 -1.05647
H -1.12575 2.263379 -1.909342
H -0.38444 2.809383 0.188895
C 3.367396 -1.76827 -1.16227
O 1.265542 0.808993 1.665724
C 2.237744 1.096674 0.66958
C 1.755636 0.794654 -0.763337
O 2.925969 0.863977 -1.564591
C 1.101831 -0.572474 -1.003459
C 1.953276 -1.785882 -0.594565
O 2.017226 -1.88082 0.810631
C 2.651858 2.556415 0.787202
O 1.444898 3.329012 0.701286
O 4.308568 -1.866267 -0.412203
H 3.112731 0.470241 0.87003
H 1.021079 1.551314 -1.062314
H 1.457755 -2.684213 -0.999258
H 3.486516 -1.717355 -2.255371
H 0.729953 1.607306 1.782882
H 2.950507 -2.043006 1.04058
H 2.734683 1.262537 -2.421106
H 3.343647 2.818477 -0.017749
H 3.129901 2.719763 1.758197
H 1.624349 4.245128 0.942116
H 0.908736 -0.635988 -2.085072
Na 0.137057 -1.181839 1.927719

Transition state of 3rd m1_2 reaction of Manβ(1→3)Manβ
46

C -1.110468 -1.144555 -1.004164
O -1.776691 -0.236503 -1.82493
C -2.618148 0.76135 -1.23781
C -3.494918 0.224112 -0.087055
O -4.545878 -0.570232 -0.601989
C -2.767219 -0.738344 0.867035
C -2.036987 -1.786391 0.027109
O -1.228003 -2.62324 0.844476
O -1.871715 -0.111369 1.769278
C -1.905489 2.084331 -0.962621
O -1.270469 2.130391 0.310218
O -0.052396 -0.535392 -0.302392
H -3.308164 0.997516 -2.056798
H -3.883755 1.075254 0.492168
H -3.543803 -1.237567 1.457138
H -2.782664 -2.367337 -0.525471
H -0.715758 -1.913347 -1.680067
H -1.738043 -3.384216 1.145085
H -1.812719 0.834883 1.562045
H -5.182109 -0.028955 -1.083366
H -2.657215 2.882032 -1.012707
H -1.176379 2.245216 -1.765744
H -0.680295 2.904263 0.36406
C 3.337833 -1.808803 -1.123237
O 0.976892 1.012036 1.598262
C 2.035646 1.365923 0.726692
C 1.75855 0.983104 -0.740935
O 2.985205 1.034962 -1.452152
C 1.192125 -0.428301 -0.961006
C 2.138945 -1.489506 -0.393174
O 2.189059 -1.710467 0.882422
C 2.318164 2.857061 0.842672
O 1.165299 3.563971 0.365387
O 4.266614 -2.392793 -0.44884
H 2.920645 0.812863 1.058202
H 1.024669 1.679562 -1.165092
H 1.919881 -2.509974 -1.084497
H 3.487124 -1.673148 -2.190446
H 0.146099 1.254366 1.156073
H 3.951546 -2.360292 0.495814
H 3.009977 1.795465 -2.042372
H 3.200414 3.123851 0.249485
H 2.500358 3.094549 1.895184
H 1.291238 4.509126 0.507205
H 1.071743 -0.555054 -2.046023
Na 0.287789 -1.177259 1.948287

Reactant state of 3rd m1_2 reaction of Manβ(1→3)Manβ
46

C -1.194781 -1.097363 -1.030887
O -1.776667 -0.10572 -1.818485
C -2.55881 0.927263 -1.210907
C -3.487251 0.413897 -0.090503
O -4.575366 -0.29968 -0.644088
C -2.833385 -0.616923 0.843674
C -2.171827 -1.692876 -0.017745
O -1.428143 -2.603096 0.786811
O -1.903923 -0.06795 1.7632
C -1.764495 2.190312 -0.886276
O -1.123817 2.146204 0.384097
O -0.082977 -0.616159 -0.31214
H -3.219662 1.234904 -2.030362
H -3.832248 1.270444 0.507913
H -3.643478 -1.075704 1.421054
H -2.95301 -2.209361 -0.584622
H -0.872244 -1.872598 -1.736871
H -1.990646 -3.336476 1.061687
H -1.760777 0.870981 1.558129
H -5.178472 0.294676 -1.105161
H -2.464516 3.035055 -0.901615
H -1.027911 2.337377 -1.685111
H -0.481391 2.876109 0.461075
C 3.426839 -1.742765 -1.130226
O 1.078894 0.829412 1.619347
C 2.136181 1.150288 0.730247
C 1.793425 0.82283 -0.734996
O 3.01209 0.887205 -1.456726
C 1.158778 -0.549652 -0.998586
C 2.004486 -1.762451 -0.581242
O 2.052154 -1.871296 0.822962
C 2.506038 2.619568 0.87332
O 1.374548 3.403802 0.469621
O 4.355671 -1.865542 -0.369171
H 3.003463 0.55084 1.025147
H 1.077607 1.570879 -1.098842
H 1.515025 -2.658203 -0.999697
H 3.561213 -1.669604 -2.220233
H 0.263689 1.18304 1.225759
H 2.984924 -2.027258 1.060773
H 2.907086 1.391281 -2.271099
H 3.371704 2.847409 0.243082
H 2.746366 2.817057 1.922502
H 1.575022 4.337881 0.598282
H 0.98877 -0.608958 -2.083953
Na 0.134416 -1.257271 1.915772

Transition state of 1st m1_4 reaction of Manβ(1→3)Manβ
46

C 1.291748 0.052711 -1.262296
O 1.581334 -1.302816 -1.320694
C 2.401432 -1.940736 -0.327333
C 3.557635 -1.058642 0.184787
O 4.590405 -0.976965 -0.779686
C 3.157671 0.407152 0.410815
C 2.505082 0.899022 -0.878505
O 2.057198 2.24968 -0.755131
O 2.323688 0.614202 1.532649
C 1.56854 -2.590475 0.770972
O 1.027981 -1.668885 1.703718
O 0.232238 0.345655 -0.353777
H 2.86311 -2.768517 -0.879928
H 3.926184 -1.474367 1.133193
H 4.083123 0.969138 0.577422
H 3.230108 0.802226 -1.693123
H 0.962922 0.320936 -2.273283
H 2.799211 2.844573 -0.921713
H 1.85254 -0.229681 1.746593
H 5.065483 -1.813427 -0.843738
H 2.210831 -3.279449 1.329465
H 0.769893 -3.176147 0.303777
H 0.121244 -1.434002 1.441026
C -2.382485 1.698749 0.638454
O -3.454599 -0.775488 -1.892482
C -2.993044 -1.4117 -0.726944
C -2.033845 -0.469559 -0.009457
O -1.585927 -0.821775 1.168299
C -1.07394 0.393414 -0.885651
C -1.668743 1.820264 -0.715316
O -0.719132 2.848267 -0.599698
C -4.137811 -1.851704 0.173696
O -4.922005 -0.682164 0.362144
O -1.608415 1.481344 1.669854
H -2.400615 -2.287207 -1.026909
H -2.910891 0.531458 0.297093
H -2.398995 2.013092 -1.511157
H -3.309206 2.256916 0.819757
H -4.317614 -0.380965 -1.692639
H -0.111696 2.855513 -1.353264
H -1.452735 0.203585 1.656971
H -3.743254 -2.234159 1.120592
H -4.709192 -2.634266 -0.337192
H -5.731575 -0.887992 0.842681
H -1.094128 0.047193 -1.924035
Na 0.632699 2.143286 1.187955

Reactant state of 1st m1_4 reaction of Manβ(1→3)Manβ
46

C 1.093101 -0.260835 -1.253782
O 1.312856 -1.590915 -0.912763
C 2.201261 -1.945444 0.160566
C 3.466612 -1.069144 0.21648
O 4.347791 -1.388606 -0.84482
C 3.179617 0.424577 0.016404
C 2.380362 0.56571 -1.278542
O 2.003483 1.923031 -1.510753
O 2.524811 1.032466 1.109867
C 1.472894 -2.105774 1.492593
O 1.026985 -0.884118 2.06142
O 0.170465 0.388902 -0.385724
H 2.534057 -2.954529 -0.114512
H 3.949857 -1.208054 1.193903
H 4.144971 0.926831 -0.108363
H 2.98787 0.181649 -2.104063
H 0.656461 -0.288354 -2.259169
H 2.709283 2.373434 -1.989266
H 1.97498 0.35507 1.586711
H 4.731571 -2.263532 -0.716185
H 2.162517 -2.563373 2.209309
H 0.623668 -2.780162 1.350081
H 0.15736 -0.68355 1.671108
C -2.042951 1.602386 1.327626
O -3.484209 -0.643716 -1.963161
C -3.082922 -1.499388 -0.918612
C -1.850702 -0.948899 -0.198815
O -1.418977 -1.485924 0.797788
C -1.18993 0.315532 -0.750046
C -1.916599 1.561555 -0.187103
O -1.208058 2.735966 -0.565917
C -4.204606 -1.721897 0.084845
O -4.601529 -0.409987 0.478146
O -0.732991 1.607902 1.897579
H -2.783739 -2.46396 -1.349385
H -2.618219 0.73748 1.67106
H -2.935474 1.569704 -0.591756
H -2.578722 2.519947 1.594114
H -4.256006 -0.14369 -1.660927
H -1.299636 2.894506 -1.513809
H -0.796648 1.609225 2.860086
H -3.831057 -2.303606 0.933028
H -5.030149 -2.253019 -0.400986
H -5.40943 -0.447742 1.002787
H -1.297786 0.327454 -1.843039
Na 0.876584 2.460727 0.456026

Transition state of 2nd m1_4 reaction of Manβ(1→3)Manβ
46

C 1.290738 0.062863 -1.266053
O 1.580539 -1.289316 -1.336409
C 2.39727 -1.939004 -0.345108
C 3.54684 -1.067877 0.189164
O 4.571187 -1.109532 -0.788698
C 3.153994 0.404363 0.411677
C 2.506269 0.902901 -0.877293
O 2.064448 2.255233 -0.751935
O 2.320893 0.616276 1.533622
C 1.556355 -2.59182 0.743199
O 1.033201 -1.674009 1.692346
O 0.232139 0.351356 -0.353701
H 2.877363 -2.749859 -0.902238
H 3.884929 -1.496158 1.142547
H 4.07684 0.973986 0.582389
H 3.230866 0.802142 -1.692213
H 0.96167 0.342191 -2.274093
H 2.804808 2.849961 -0.925114
H 1.851219 -0.229936 1.745664
H 5.431572 -0.955791 -0.381354
H 2.187779 -3.300311 1.288815
H 0.746345 -3.155191 0.268471
H 0.12317 -1.436038 1.445373
C -2.385534 1.700706 0.63615
O -3.454458 -0.780379 -1.885776
C -2.989038 -1.414697 -0.720693
C -2.031686 -0.468898 -0.005731
O -1.583225 -0.816625 1.173144
C -1.074488 0.394031 -0.884659
C -1.673033 1.819855 -0.718408
O -0.725157 2.850289 -0.60607
C -4.130851 -1.857708 0.182142
O -4.918217 -0.690409 0.371543
O -1.609939 1.488558 1.667554
H -2.394277 -2.288461 -1.021182
H -2.911483 0.531867 0.298805
H -2.403995 2.009021 -1.514375
H -3.313093 2.257868 0.816373
H -4.318842 -0.389403 -1.684912
H -0.12953 2.867284 -1.368577
H -1.451245 0.208659 1.65812
H -3.733529 -2.238607 1.128497
H -4.70074 -2.642213 -0.32741
H -5.726671 -0.898719 0.852881
H -1.094509 0.04408 -1.921806
Na 0.628094 2.14671 1.181279

Reactant state of 2nd m1_4 reaction of Manβ(1→3)Manβ
46

C 1.33346 0.498875 -1.088185
O 1.894538 -0.672612 -1.577232
C 2.785951 -1.477727 -0.794357
C 3.742709 -0.645802 0.079903
O 4.722896 -0.007729 -0.715681
C 3.048393 0.518067 0.800177
C 2.308367 1.338862 -0.255762
O 1.543115 2.385975 0.360795
O 2.189598 0.106405 1.843739
C 2.063021 -2.609144 -0.070249
O 1.336237 -2.200118 1.077556
O 0.177559 0.259275 -0.279567
H 3.407565 -1.970021 -1.553379
H 4.200615 -1.304044 0.832186
H 3.834417 1.140107 1.242196
H 3.043574 1.758041 -0.948953
H 1.019703 1.059106 -1.972232
H 2.10457 3.15988 0.495646
H 1.913149 -0.837728 1.6868
H 5.332988 -0.653174 -1.091007
H 2.809194 -3.336883 0.264963
H 1.39845 -3.108083 -0.783122
H 0.416442 -1.984013 0.830941
C -2.519614 1.917907 0.224338
O -3.809368 -0.397358 -1.637883
C -3.238415 -1.368542 -0.800236
C -1.829843 -0.993575 -0.324801
O -1.315544 -1.695399 0.524537
C -1.052479 0.146656 -0.980698
C -1.747496 1.543187 -1.051705
O -0.788773 2.51351 -1.398883
C -4.113116 -1.703205 0.398299
O -4.28345 -0.480745 1.123477
O -2.017543 1.264683 1.397412
H -3.10309 -2.287536 -1.38831
H -3.576625 1.66531 0.117029
H -2.449829 1.523036 -1.885052
H -2.441818 2.999529 0.365832
H -4.497035 0.077183 -1.151734
H -0.131926 2.616409 -0.692984
H -2.687634 0.621491 1.680518
H -3.622502 -2.461214 1.013802
H -5.080013 -2.07398 0.042959
H -4.986921 -0.580146 1.776346
H -0.86212 -0.176238 -2.016399
Na 0.189009 1.141139 1.869769

Transition state of 3rd m1_4 reaction of Manβ(1→3)Manβ
46

C 1.301397 0.066081 -1.264014
O 1.599721 -1.287506 -1.331509
C 2.409918 -1.92847 -0.331309
C 3.556299 -1.044421 0.199152
O 4.598394 -0.951714 -0.754461
C 3.147119 0.417982 0.43173
C 2.505665 0.91626 -0.860783
O 2.047985 2.262894 -0.732236
O 2.3007 0.613368 1.545798
C 1.567348 -2.588032 0.753862
O 1.018201 -1.675718 1.689831
O 0.231335 0.344504 -0.364848
H 2.881739 -2.750805 -0.883377
H 3.91687 -1.465211 1.148321
H 4.068401 0.982757 0.611651
H 3.239908 0.829843 -1.668356
H 0.98225 0.341611 -2.276321
H 2.788839 2.863655 -0.882125
H 1.831919 -0.234703 1.749606
H 5.085575 -1.781748 -0.810257
H 2.205521 -3.281955 1.311115
H 0.773309 -3.170278 0.274483
H 0.109769 -1.445947 1.426828
C -2.400501 1.665602 0.616475
O -3.467106 -0.70174 -1.865408
C -3.002498 -1.423265 -0.738829
C -2.031838 -0.48748 -0.044338
O -1.589024 -0.848971 1.13185
C -1.069945 0.38566 -0.908071
C -1.678563 1.806438 -0.730996
O -0.737727 2.840936 -0.599885
C -4.148906 -1.821083 0.192807
O -4.852817 -0.679317 0.62312
O -1.631974 1.446995 1.651632
H -2.427768 -2.31434 -1.036128
H -2.918729 0.498105 0.262943
H -2.406202 1.998155 -1.529138
H -3.336647 2.207407 0.797599
H -3.818521 -1.30237 -2.533375
H -0.126101 2.86141 -1.349607
H -1.476569 0.177668 1.636522
H -3.743164 -2.306663 1.08317
H -4.802368 -2.53311 -0.326574
H -5.435889 -0.377327 -0.083496
H -1.07498 0.048348 -1.949322
Na 0.60076 2.132183 1.198242

Reactant state of 3rd m1_4 reaction of Manβ(1→3)Manβ
46

C 1.093728 -0.265857 -1.25287
O 1.332577 -1.592044 -0.909697
C 2.22074 -1.92951 0.169775
C 3.474756 -1.036934 0.224615
O 4.362617 -1.351213 -0.832903
C 3.170002 0.452315 0.016172
C 2.370362 0.576764 -1.2803
O 1.976766 1.927578 -1.521123
O 2.50743 1.059331 1.105006
C 1.490553 -2.091164 1.500653
O 1.032046 -0.871829 2.06366
O 0.161612 0.372979 -0.387505
H 2.567035 -2.935824 -0.098528
H 3.957145 -1.164711 1.203927
H 4.129764 0.964731 -0.110947
H 2.983504 0.19539 -2.103023
H 0.658392 -0.301228 -2.258756
H 2.682775 2.386817 -1.990953
H 1.966222 0.378488 1.586572
H 4.76205 -2.217619 -0.694739
H 2.183682 -2.538337 2.220578
H 0.648787 -2.77499 1.359914
H 0.162925 -0.678109 1.669661
C -2.060302 1.571444 1.322646
O -3.470191 -0.644118 -1.926239
C -3.098883 -1.532907 -0.885683
C -1.84634 -0.983542 -0.209976
O -1.398364 -1.528715 0.773797
C -1.196814 0.289116 -0.756045
C -1.93973 1.525666 -0.192874
O -1.245538 2.707012 -0.580675
C -4.215396 -1.65024 0.152415
O -4.539908 -0.366267 0.647609
O -0.746579 1.599847 1.887405
H -2.833176 -2.527285 -1.270995
H -2.624817 0.704738 1.675869
H -2.957038 1.513363 -0.600811
H -2.606276 2.484148 1.585298
H -3.938544 -1.117332 -2.623358
H -1.365642 2.875884 -1.52322
H -0.809232 1.597276 2.849999
H -3.8659 -2.249814 0.995833
H -5.087447 -2.142685 -0.293827
H -5.106631 0.0802 0.007518
H -1.297802 0.305994 -1.849248
Na 0.839492 2.464502 0.44158

Transition state of 1st m1_5 reaction of Manβ(1→3)Manβ
46

C 1.294494 0.187573 -1.279074
O 1.594616 -1.161194 -1.421767
C 2.383697 -1.87534 -0.457766
C 3.494081 -1.029894 0.197882
O 4.581354 -0.852913 -0.691483
C 3.058876 0.405741 0.526951
C 2.474384 1.002258 -0.748749
O 2.006079 2.334755 -0.538268
O 2.154216 0.505946 1.607564
C 1.514634 -2.63905 0.532396
O 0.931349 -1.81241 1.525358
O 0.153009 0.412361 -0.452056
H 2.889892 -2.63791 -1.06297
H 3.81257 -1.52451 1.126352
H 3.9641 0.96003 0.798105
H 3.245482 0.979677 -1.525759
H 1.049576 0.533798 -2.289625
H 2.743616 2.948816 -0.639378
H 1.688225 -0.360196 1.721424
H 5.072435 -1.675608 -0.79812
H 2.140596 -3.378125 1.04431
H 0.74122 -3.178522 -0.024329
H 0.002188 -1.620963 1.307967
C -2.45602 1.508537 0.790521
O -1.691584 -1.026388 1.319131
C -2.563116 -0.877031 0.34879
C -1.986553 -0.690648 -1.062738
O -2.959693 -0.443254 -2.042228
C -1.08245 0.553983 -1.129746
C -1.848267 1.785747 -0.582088
O -0.975764 2.882962 -0.370341
C -3.821475 -1.706817 0.567767
O -4.840801 -1.273601 -0.308818
O -1.619006 1.267584 1.761312
H -3.05711 0.353162 0.505707
H -1.382305 -1.585036 -1.28729
H -2.648809 2.032042 -1.287067
H -3.37634 2.045304 1.04974
H -1.481549 0.045419 1.714847
H -0.544353 3.135184 -1.197914
H -3.797764 -0.882091 -1.827669
H -4.10384 -1.605505 1.620752
H -3.541602 -2.753719 0.380054
H -5.644278 -1.790718 -0.176155
H -0.901246 0.725024 -2.199357
Na 0.516853 2.073938 1.246751

Reactant state of 1st m1_5 reaction of Manβ(1→3)Manβ
46

C 0.875824 -0.308553 -1.236752
O 0.894946 -1.660798 -0.908974
C 1.761678 -2.16716 0.117854
C 3.152364 -1.504201 0.129423
O 3.928399 -1.954153 -0.965867
C 3.104739 0.017747 -0.059319
C 2.278148 0.298353 -1.312574
O 2.117098 1.701109 -1.522122
O 2.616114 0.723195 1.062176
C 1.060097 -2.21533 1.470425
O 0.839144 -0.940037 2.048903
O 0.076524 0.4585 -0.335706
H 1.918325 -3.212709 -0.176681
H 3.643108 -1.726218 1.087574
H 4.132722 0.352967 -0.233153
H 2.779098 -0.169741 -2.165996
H 0.411442 -0.259334 -2.228022
H 2.825806 2.025854 -2.089546
H 1.999955 0.146072 1.579683
H 4.192244 -2.873098 -0.842329
H 1.679348 -2.78463 2.170738
H 0.105803 -2.738915 1.34595
H 0.033246 -0.572899 1.64585
C -1.50022 1.95411 1.431143
O -1.851391 -0.791195 1.406747
C -2.877359 -0.683436 0.42829
C -2.235655 -0.276041 -0.905295
O -3.215533 0.239783 -1.776582
C -1.191599 0.847911 -0.841289
C -1.619104 2.115813 -0.081389
O -0.729729 3.175869 -0.410323
C -3.602543 -2.022188 0.308084
O -4.55527 -1.896669 -0.730469
O -0.453817 2.162639 2.00134
H -3.602442 0.097908 0.707155
H -1.731628 -1.158665 -1.3326
H -2.653567 2.356576 -0.352563
H -2.401042 1.656157 1.989599
H -2.222697 -1.067636 2.255683
H -0.925512 3.501138 -1.297708
H -3.930523 -0.40952 -1.86157
H -4.089195 -2.263361 1.263309
H -2.859241 -2.799166 0.082081
H -5.061626 -2.710075 -0.833949
H -1.076106 1.168431 -1.887588
Na 1.296578 2.486616 0.502681

Transition state of 2nd m1_5 reaction of Manβ(1→3)Manβ
46

C -1.292711 0.200784 1.282546
O -1.589943 -1.144888 1.435425
C -2.377979 -1.871809 0.476971
C -3.481212 -1.040899 -0.200398
O -4.563834 -0.987481 0.712434
C -3.055 0.402033 -0.526378
C -2.47702 1.005902 0.748289
O -2.019101 2.341231 0.534935
O -2.150272 0.509006 -1.606672
C -1.502678 -2.638737 -0.502843
O -0.938776 -1.817396 -1.513923
O -0.152438 0.425219 0.452399
H -2.901701 -2.615826 1.085789
H -3.767825 -1.548932 -1.13123
H -3.9579 0.962402 -0.802561
H -3.247523 0.976786 1.526094
H -1.048084 0.557809 2.289539
H -2.755231 2.954223 0.649295
H -1.686199 -0.359167 -1.719643
H -5.394591 -0.856588 0.240766
H -2.118931 -3.396834 -0.997641
H -0.716628 -3.154114 0.058791
H -0.005502 -1.62648 -1.315763
C 2.460305 1.50745 -0.792428
O 1.683539 -1.022611 -1.325708
C 2.556013 -0.879501 -0.355333
C 1.981893 -0.692109 1.0572
O 2.958111 -0.450811 2.035286
C 1.084846 0.557508 1.128353
C 1.85807 1.785324 0.582178
O 0.992372 2.889323 0.375109
C 3.809646 -1.715741 -0.57688
O 4.832322 -1.290609 0.299687
O 1.618663 1.272994 -1.760957
H 3.056274 0.349166 -0.511593
H 1.37334 -1.583594 1.281498
H 2.661994 2.024436 1.285641
H 3.382104 2.040829 -1.053421
H 1.4758 0.05023 -1.716734
H 0.596698 3.168256 1.21159
H 3.792378 -0.896805 1.820777
H 4.091716 -1.61388 -1.629892
H 3.523627 -2.761368 -0.391234
H 5.632038 -1.813235 0.165865
H 0.906315 0.726851 2.198666
Na -0.51294 2.082071 -1.234216

Reactant state of 2nd m1_5 reaction of Manβ(1→3)Manβ
46

C 0.872484 -0.300403 -1.241951
O 0.892008 -1.652042 -0.922359
C 1.75717 -2.167489 0.104121
C 3.14341 -1.50498 0.135515
O 3.881297 -2.074625 -0.931895
C 3.1003 0.019512 -0.062309
C 2.276279 0.302555 -1.316385
O 2.121307 1.706024 -1.525488
O 2.614576 0.73 1.057662
C 1.050263 -2.219086 1.451913
O 0.845054 -0.944449 2.041695
O 0.07546 0.46642 -0.337553
H 1.934085 -3.203379 -0.203295
H 3.606851 -1.726966 1.106501
H 4.127091 0.365764 -0.237076
H 2.773959 -0.169299 -2.169909
H 0.408257 -0.243694 -2.233036
H 2.815054 2.027529 -2.112464
H 2.002525 0.148968 1.577599
H 4.827301 -2.007972 -0.757307
H 1.658513 -2.803604 2.148771
H 0.088871 -2.726942 1.318745
H 0.038511 -0.569246 1.647824
C -1.501779 1.953051 1.437095
O -1.847346 -0.787866 1.409475
C -2.8739 -0.688442 0.430849
C -2.235432 -0.276515 -0.902888
O -3.218844 0.23687 -1.771821
C -1.195902 0.851551 -0.838604
C -1.626618 2.115934 -0.074901
O -0.742822 3.18055 -0.405777
C -3.588509 -2.032772 0.309855
O -4.54143 -1.913844 -0.729371
O -0.453219 2.162683 2.00314
H -3.604855 0.087453 0.709614
H -1.728582 -1.156287 -1.33248
H -2.662924 2.352643 -0.342402
H -2.399919 1.653524 1.998874
H -2.215461 -1.07298 2.256905
H -0.950237 3.512908 -1.287821
H -3.92958 -0.416855 -1.858697
H -4.074125 -2.278281 1.264488
H -2.838925 -2.803673 0.083849
H -5.036754 -2.733385 -0.837834
H -1.084579 1.175178 -1.884422
Na 1.288182 2.490304 0.495276

Transition state of 3rd m1_5 reaction of Manβ(1→3)Manβ
46

C 1.108577 -0.047617 -1.279391
O 1.575682 -1.355263 -1.190476
C 2.613186 -1.728299 -0.279071
C 3.72478 -0.672416 -0.164703
O 4.495864 -0.622172 -1.349156
C 3.198819 0.757207 -0.023008
C 2.212089 1.012557 -1.157977
O 1.565521 2.271686 -1.043554
O 2.599763 0.970415 1.250629
C 2.019176 -2.284018 1.031285
O 0.914509 -1.555684 1.523656
O 0.086206 0.198179 -0.321936
H 3.090012 -2.586138 -0.770523
H 4.349802 -0.916097 0.706845
H 4.045169 1.442637 -0.146944
H 2.797057 0.954792 -2.082322
H 0.661369 0.027195 -2.27697
H 2.091139 2.957138 -1.472622
H 3.278091 1.292162 1.855536
H 4.966824 -1.451867 -1.490509
H 2.783323 -2.290019 1.814041
H 1.716018 -3.321258 0.854072
H 0.175683 -1.67827 0.903074
C -2.399002 1.042629 1.360236
O -1.96378 -1.538407 0.691685
C -2.779715 -0.87519 -0.090639
C -2.154043 -0.147798 -1.288988
O -3.056493 0.658692 -1.997866
C -1.074533 0.839796 -0.82311
C -1.618933 1.790521 0.27229
O -0.546171 2.438824 0.932735
C -4.134533 -1.55867 -0.224416
O -5.06454 -0.688813 -0.837118
O -1.715879 0.26707 2.156095
H -3.118248 0.23593 0.586691
H -1.691935 -0.9217 -1.924869
H -2.290858 2.509021 -0.208935
H -3.260564 1.56003 1.798533
H -1.722885 -0.823569 1.556177
H 0.137778 2.663623 0.276381
H -3.944433 0.268197 -2.001553
H -4.448929 -1.864052 0.779126
H -3.965818 -2.462656 -0.827075
H -5.919499 -1.123829 -0.936207
H -0.815267 1.456818 -1.691391
Na 0.515046 0.631227 2.016986

Reactant state of 3rd m1_5 reaction of Manβ(1→3)Manβ
46

C 0.921228 -0.523531 -1.167096
O 1.301856 -1.738922 -0.609867
C 2.365746 -1.831762 0.343747
C 3.548201 -0.895289 0.038557
O 4.291996 -1.372329 -1.06732
C 3.134688 0.51157 -0.401901
C 2.111674 0.373507 -1.523819
O 1.577825 1.623834 -1.931819
O 2.622484 1.280929 0.67979
C 1.810606 -1.838393 1.777058
O 0.866636 -0.826536 2.045944
O 0.010662 0.167802 -0.319428
H 2.7634 -2.843146 0.186772
H 4.176585 -0.820282 0.937701
H 4.024807 1.004096 -0.809992
H 2.631805 -0.119483 -2.352542
H 0.392717 -0.78458 -2.090724
H 2.147918 2.036828 -2.591222
H 3.345057 1.796903 1.055047
H 4.70122 -2.222305 -0.867154
H 2.632511 -1.726082 2.49071
H 1.350711 -2.817889 1.95024
H 0.057203 -1.025719 1.538617
C -2.187555 2.019618 0.977481
O -1.81638 -1.072748 1.284493
C -2.858349 -0.820628 0.352049
C -2.308384 -0.278365 -0.986015
O -3.325982 0.428307 -1.659916
C -1.135443 0.708989 -0.958277
C -1.419941 2.10587 -0.33183
O -0.204082 2.761252 -0.042661
C -3.627189 -2.119668 0.107953
O -4.60093 -1.854325 -0.887008
O -1.630155 1.857842 2.042061
H -3.556008 -0.072006 0.750356
H -1.966787 -1.14396 -1.578665
H -2.031756 2.669409 -1.045286
H -3.28535 2.107825 0.920507
H -2.200834 -1.195999 2.162149
H 0.439037 2.578384 -0.752528
H -4.071688 -0.174662 -1.805377
H -4.096521 -2.449327 1.044073
H -2.915011 -2.888028 -0.22114
H -5.131476 -2.638334 -1.066394
H -0.898716 0.913971 -2.00992
Na 0.600732 1.391094 1.683015

Transition state of 1st m1_6 reaction of Manβ(1→3)Manβ
46

C 1.249031 -0.299837 1.139951
O 1.915275 0.902362 1.360405
C 2.920173 1.407609 0.467042
C 3.721264 0.314515 -0.270452
O 4.694385 -0.255043 0.584825
C 2.860628 -0.873246 -0.710892
C 2.132618 -1.389582 0.525267
O 1.353721 -2.516222 0.147366
O 1.970575 -0.549342 -1.766148
C 2.344191 2.499363 -0.426285
O 1.368365 2.017582 -1.336385
O 0.110878 -0.155431 0.287318
H 3.635097 1.907452 1.133122
H 4.184433 0.759301 -1.161994
H 3.527917 -1.658336 -1.076906
H 2.88834 -1.669834 1.266933
H 0.893652 -0.607885 2.127609
H 0.771708 -2.758382 0.883358
H 1.704854 0.40076 -1.704174
H 5.436236 0.348508 0.704786
H 3.154536 2.951253 -1.00813
H 1.909136 3.276593 0.210394
H 0.498088 2.161625 -0.927746
C -2.71908 -1.601396 0.102334
O -3.595003 1.462716 1.445876
C -3.258416 1.426939 0.075123
C -1.748195 1.239215 -0.055061
O -1.068485 2.477178 0.049938
C -1.101437 0.2272 0.912875
C -1.937932 -1.017756 1.277342
O -1.04202 -2.010254 1.76981
C -4.037501 0.336379 -0.66609
O -3.676518 0.031976 -1.870365
O -2.180051 -1.597884 -1.091846
H -3.501363 2.370818 -0.433281
H -1.548126 0.902962 -1.076099
H -2.651043 -0.728088 2.057036
H -3.316968 -2.488857 0.362063
H -4.353097 2.036398 1.608423
H -1.473451 -2.524214 2.462999
H -1.141863 2.825082 0.949416
H -3.693094 -0.750482 0.082373
H -5.108085 0.269723 -0.42797
H -2.884558 -0.766874 -1.757283
H -0.877491 0.751294 1.852091
Na 0.053101 -1.735122 -1.545481

Reactant state of 1st m1_6 reaction of Manβ(1→3)Manβ
46

C -1.069742 -0.162541 -1.093337
O -1.609944 1.112192 -1.248077
C -2.713899 1.588823 -0.466577
C -3.722882 0.488665 -0.083607
O -4.51031 0.12238 -1.200686
C -3.050892 -0.815534 0.356057
C -2.105729 -1.244807 -0.761066
O -1.462693 -2.448526 -0.364521
O -2.394374 -0.696476 1.604598
C -2.230166 2.459762 0.687926
O -1.507868 1.742545 1.673741
O -0.040701 -0.212849 -0.101564
H -3.244355 2.264253 -1.150498
H -4.347245 0.855353 0.743131
H -3.827276 -1.578166 0.464395
H -2.711688 -1.405573 -1.659375
H -0.607155 -0.383839 -2.059178
H -0.795039 -2.681993 -1.027471
H -2.080472 0.23904 1.737806
H -5.150698 0.813086 -1.405322
H -3.095241 2.919215 1.17736
H -1.607831 3.263096 0.278769
H -0.565227 1.737711 1.409904
C 2.334632 -2.109183 0.286617
O 2.970326 2.460135 -1.05191
C 3.274817 1.637191 0.065585
C 1.941679 0.990089 0.476186
O 1.063056 2.060559 0.77957
C 1.264841 0.092552 -0.578325
C 1.998874 -1.217171 -0.906701
O 1.140377 -2.023577 -1.72182
C 4.419998 0.684452 -0.278298
O 4.331162 -0.456576 0.563175
O 1.698721 -2.121257 1.318332
H 3.55982 2.253344 0.927572
H 2.086667 0.42846 1.402513
H 2.924142 -1.000527 -1.445229
H 3.146405 -2.83808 0.124024
H 3.577249 3.206861 -1.109791
H 1.619989 -2.367992 -2.484286
H 1.119179 2.695085 0.047281
H 4.347882 0.401463 -1.33632
H 5.375985 1.200786 -0.142963
H 5.207034 -0.759883 0.825529
H 1.182554 0.671148 -1.50948
Na -0.507807 -1.872277 1.632559

Transition state of 2nd m1_6 reaction of Manβ(1→3)Manβ
46

C -1.234513 -0.337505 -1.140547
O -1.927192 0.85282 -1.380662
C -2.944483 1.35055 -0.508726
C -3.706262 0.269793 0.294564
O -4.706373 -0.324418 -0.508328
C -2.823476 -0.904095 0.727257
C -2.107755 -1.427389 -0.512894
O -1.321171 -2.545561 -0.132572
O -1.921245 -0.557619 1.769545
C -2.429287 2.529227 0.301277
O -1.414964 2.108495 1.224576
O -0.1144 -0.156907 -0.286385
H -3.686567 1.78667 -1.189697
H -4.136748 0.732135 1.194038
H -3.47347 -1.690771 1.118519
H -2.871951 -1.716546 -1.242912
H -0.875656 -0.648988 -2.12521
H -0.716171 -2.766073 -0.858127
H -1.641235 0.372411 1.663545
H -5.467427 0.260107 -0.599711
H -3.268818 2.981832 0.840389
H -2.009251 3.269445 -0.388388
H -1.336351 2.754659 1.938535
C 2.761579 -1.571981 -0.091088
O 3.665498 1.438719 -1.436323
C 3.234718 1.459974 -0.093913
C 1.728923 1.258576 0.051495
O 1.114734 2.506437 -0.168849
C 1.10894 0.22668 -0.911373
C 1.952114 -1.017017 -1.260089
O 1.062201 -2.03517 -1.721827
C 4.040382 0.403979 0.65083
O 3.699163 0.094162 1.863011
O 2.229873 -1.581367 1.106003
H 3.477014 2.414833 0.395557
H 1.532581 0.922647 1.078405
H 2.647874 -0.732714 -2.056716
H 3.379546 -2.445008 -0.354144
H 3.489363 2.292816 -1.84987
H 1.501576 -2.566525 -2.39666
H 0.198712 2.430004 0.141822
H 3.717033 -0.702711 -0.08461
H 5.107689 0.370822 0.400219
H 2.927448 -0.70037 1.768456
H 0.884242 0.754953 -1.845489
Na 0.018754 -1.746828 1.52598

Reactant state of 2nd m1_6 reaction of Manβ(1→3)Manβ
46

C -1.038156 -0.226085 -1.088961
O -1.604248 1.033595 -1.2885
C -2.737107 1.512435 -0.563701
C -3.709295 0.410483 -0.085019
O -4.531977 -0.015991 -1.152034
C -3.004315 -0.869363 0.377066
C -2.059682 -1.312105 -0.733948
O -1.401932 -2.497233 -0.312765
O -2.332009 -0.712134 1.618671
C -2.327375 2.532083 0.48662
O -1.66968 1.890241 1.58523
O -0.025298 -0.220577 -0.085747
H -3.294329 2.096094 -1.307702
H -4.30751 0.801966 0.75051
H -3.766201 -1.641026 0.516229
H -2.669412 -1.498984 -1.625103
H -0.561992 -0.464736 -2.042832
H -0.717479 -2.725126 -0.961882
H -2.124333 0.231079 1.770029
H -5.209158 0.641398 -1.348513
H -3.224711 3.052208 0.840937
H -1.646758 3.254068 0.023878
H -1.549325 2.515965 2.311456
C 2.436435 -2.067 0.302917
O 2.978641 2.496758 -1.09692
C 3.223118 1.714355 0.049255
C 1.940352 0.998807 0.49911
O 1.054936 2.063812 0.781916
C 1.289005 0.097904 -0.561393
C 2.044548 -1.197298 -0.889509
O 1.19028 -2.039258 -1.677283
C 4.404025 0.817281 -0.306465
O 4.374953 -0.345745 0.51585
O 1.78472 -2.133407 1.323928
H 3.498672 2.351337 0.901388
H 2.154291 0.416518 1.404773
H 2.948854 -0.953043 -1.451618
H 3.302688 -2.731457 0.152702
H 2.299186 3.146506 -0.871743
H 1.671345 -2.394754 -2.433869
H 0.167404 1.70817 0.946254
H 4.33734 0.546002 -1.368122
H 5.334116 1.375317 -0.171009
H 5.268529 -0.606598 0.761818
H 1.198469 0.685447 -1.484101
Na -0.402372 -1.857802 1.642901

Transition state of 3rd m1_6 reaction of Manβ(1→3)Manβ
46

C -0.587859 -0.728431 -1.517366
O -1.448947 0.373043 -1.559519
C -2.626108 0.343638 -0.737108
C -2.316009 -0.335503 0.602648
O -3.4063 -0.386437 1.48713
C -1.911626 -1.795972 0.362963
C -1.313196 -1.98206 -1.044741
O -0.386139 -3.051905 -1.109954
O -0.962948 -2.23589 1.328652
C -3.047658 1.803638 -0.617572
O -4.234937 1.841605 0.152174
O 0.505231 -0.516927 -0.641224
H -3.432295 -0.202685 -1.249276
H -1.451879 0.177888 1.057997
H -2.822211 -2.40239 0.449277
H -2.135208 -2.147671 -1.750532
H -0.200011 -0.871768 -2.532834
H -0.855602 -3.892089 -1.178811
H -1.337655 -2.085009 2.207089
H -3.872755 0.463489 1.489863
H -3.201965 2.203016 -1.625934
H -2.245978 2.379718 -0.134983
H -4.649069 2.709895 0.100692
C 2.972773 -0.107947 0.994347
O 0.781309 3.609672 0.122172
C 0.741861 2.200963 0.229799
C 1.420322 1.724455 -1.060239
O 2.70892 2.288512 -1.171576
C 1.607788 0.203611 -1.184466
C 2.893235 -0.313069 -0.526942
O 2.928915 -1.716491 -0.785498
C 1.398439 1.741894 1.53592
O 0.757273 0.868561 2.257111
O 2.222282 -0.873447 1.733158
H -0.281401 1.814086 0.243084
H 0.771347 2.064369 -1.878777
H 3.730839 0.19981 -1.004706
H 3.970739 0.130588 1.388889
H 0.24024 4.028339 0.801983
H 3.837783 -2.010975 -0.915672
H 2.619608 3.252186 -1.167666
H 2.499027 1.1122 1.100792
H 1.958246 2.524446 2.07009
H 1.315768 -0.107709 2.170946
H 1.705844 -0.045198 -2.24961
Na 1.157049 -2.493473 0.534875

Reactant state of 3rd m1_6 reaction of Manβ(1→3)Manβ
46

C -0.711417 -0.963506 -1.418847
O -1.676588 -0.026596 -1.775806
C -2.753358 0.226591 -0.857656
C -2.403806 -0.196594 0.576742
O -3.477687 -0.055934 1.474262
C -2.017639 -1.676581 0.633929
C -1.339413 -2.133158 -0.673008
O -0.328267 -3.097814 -0.454687
O -1.126356 -1.927191 1.716198
C -3.013719 1.723051 -0.995268
O -4.058816 2.074506 -0.106231
O 0.290347 -0.377015 -0.594044
H -3.645036 -0.328325 -1.183327
H -1.527794 0.377981 0.912877
H -2.940979 -2.249429 0.78712
H -2.109441 -2.528806 -1.346337
H -0.248432 -1.315993 -2.345107
H -0.730984 -3.929208 -0.174047
H -1.548256 -1.59643 2.520829
H -3.883398 0.817463 1.360569
H -3.276801 1.937542 -2.036582
H -2.087468 2.264046 -0.752276
H -4.41751 2.941861 -0.321974
C 3.432454 -0.29991 0.127407
O 1.022152 3.578702 0.514262
C 0.845874 2.168165 0.532132
C 1.385795 1.786218 -0.857043
O 2.737448 2.176646 -0.990968
C 1.317156 0.314821 -1.289725
C 2.654532 -0.477712 -1.173847
O 2.263448 -1.82773 -1.318045
C 1.555669 1.604469 1.762823
O 1.050363 0.310994 2.085464
O 3.287841 -1.063653 1.059369
H -0.215546 1.89795 0.582804
H 0.759843 2.360249 -1.554265
H 3.299888 -0.136427 -1.99182
H 4.147183 0.535392 0.174009
H 0.595819 3.992871 1.273267
H 3.021118 -2.373638 -1.562158
H 2.791297 3.131023 -0.837988
H 2.641642 1.586479 1.605198
H 1.356517 2.289134 2.595892
H 1.353592 0.102394 2.977839
H 1.080807 0.295013 -2.359215
Na 1.090917 -1.876873 0.962787

Transition state of 1st RO1' reaction of Manβ(1→3)Manβ
46

C 0.577702 -1.162069 -0.312215
O 1.011859 -0.24782 0.884422
C 2.374332 -0.444056 1.429296
C 3.211304 -1.303781 0.475764
O 2.826305 -2.638152 0.759301
C 3.014003 -0.992017 -1.018537
C 1.516171 -0.667914 -1.420527
O 1.356077 0.68103 -1.551362
O 3.798176 0.13217 -1.359105
C 2.911844 0.940398 1.773814
O 2.94141 1.804053 0.653747
O -0.688679 -0.798084 -0.579951
H 2.225948 -1.017821 2.347675
H 4.267821 -1.135334 0.727213
H 3.372072 -1.868871 -1.572039
H 1.256173 -1.232065 -2.331786
H 0.704059 -2.173908 0.080851
H 0.954435 0.629683 0.402269
H 3.168991 0.677409 -1.882295
H 3.457436 -3.259677 0.375331
H 3.907224 0.82111 2.217321
H 2.262859 1.405421 2.520532
H 3.501023 1.370993 -0.027901
C -3.519753 0.14506 -0.86034
O -3.739445 0.794885 0.36403
C -2.621386 0.918032 1.248022
C -1.996304 -0.460456 1.514033
O -2.850418 -1.217394 2.348032
C -1.754908 -1.297593 0.237623
C -2.995422 -1.281713 -0.660781
O -2.76412 -1.91182 -1.885067
C -1.690866 2.096785 0.929065
O -0.606642 1.87402 0.029834
O -2.589725 0.844054 -1.664719
H -3.091553 1.212345 2.193351
H -1.059392 -0.337188 2.064076
H -3.784064 -1.847281 -0.155119
H -4.495222 0.111286 -1.351871
H -3.043429 1.547776 -2.144963
H -2.134221 -1.368871 -2.380824
H -3.77341 -1.043046 2.118546
H -1.228287 2.413093 1.868575
H -2.323704 2.915309 0.56709
H -0.923646 1.352239 -0.735915
H -1.528651 -2.327836 0.5373
Na 1.326261 2.697786 -0.858366

Reactant state of 1st RO1' reaction of Manβ(1→3)Manβ
46

C 0.6887 -0.991074 0.410133
O 1.183066 0.313151 0.713528
C 2.462995 0.278525 1.349653
C 3.378146 -0.792733 0.710876
O 3.230992 -1.961652 1.498481
C 2.971518 -1.129559 -0.72793
C 1.467661 -1.528658 -0.800491
O 0.955619 -0.939411 -1.987668
O 3.247746 0.008867 -1.536409
C 2.982396 1.710868 1.319249
O 3.016993 2.26232 0.009821
O -0.628451 -0.887486 0.015234
H 2.3406 -0.018133 2.401328
H 4.417309 -0.437893 0.715625
H 3.585277 -1.969627 -1.070182
H 1.369502 -2.619065 -0.835894
H 0.803907 -1.637353 1.292001
H 0.017112 -1.17408 -2.04883
H 3.278109 -0.273057 -2.460078
H 3.985733 -2.551703 1.384004
H 3.966875 1.760759 1.79745
H 2.295362 2.337988 1.891834
H 3.779319 1.907018 -0.467067
C -3.519496 -0.530188 -0.613968
O -3.743649 0.789102 -0.227087
C -2.691129 1.532 0.393478
C -1.884795 0.72219 1.428508
O -2.620219 0.573399 2.629576
C -1.61616 -0.740122 1.03046
C -2.898555 -1.374704 0.499151
O -2.701389 -2.695372 0.079337
C -1.875544 2.322046 -0.628263
O -0.961293 1.557712 -1.401624
O -2.672871 -0.612785 -1.768362
H -3.231223 2.300996 0.961366
H -0.927619 1.230524 1.604696
H -3.618021 -1.3939 1.322022
H -4.502698 -0.931695 -0.870214
H -3.216456 -0.487535 -2.55717
H -2.151422 -2.672401 -0.714775
H -2.825333 1.432446 3.01583
H -1.286373 3.074393 -0.093461
H -2.581102 2.844178 -1.284867
H -1.41904 0.739586 -1.67229
H -1.295929 -1.278751 1.930664
Na 1.269941 1.425728 -1.33325

Transition state of 2nd RO1' reaction of Manβ(1→3)Manβ
46

C 0.587079 -1.152902 0.57978
O 1.467897 0.926418 0.392229
C 2.774011 1.023286 0.97885
C 3.337659 -0.393791 1.187598
O 2.718091 -0.980481 2.33323
C 3.097162 -1.396347 0.045119
C 1.586749 -1.562021 -0.444003
O 1.379021 -0.943377 -1.628973
O 3.830905 -1.035716 -1.093313
C 3.617741 1.978193 0.127168
O 3.463289 1.718558 -1.247147
O -0.575394 -0.835777 0.157659
H 2.637541 1.463726 1.972351
H 4.424038 -0.328567 1.328411
H 3.447123 -2.361207 0.427251
H 1.398947 -2.665526 -0.475801
H 0.73865 -1.297378 1.65237
H 1.635163 0.726158 -0.562862
H 3.15226 -0.995544 -1.808535
H 3.224181 -0.792744 3.131323
H 4.667275 1.932915 0.445396
H 3.258469 2.997408 0.291008
H 3.950404 0.905599 -1.45389
C -3.55285 -0.488518 -0.564649
O -3.808966 0.805759 -0.124338
C -2.744018 1.600678 0.416482
C -1.91953 0.829636 1.459859
O -2.578458 0.807288 2.70932
C -1.659225 -0.651486 1.113917
C -2.886764 -1.335906 0.518626
O -2.607024 -2.635557 0.088785
C -1.933186 2.352144 -0.642582
O -0.779448 1.670243 -1.108048
O -2.746884 -0.524173 -1.744959
H -3.284867 2.375969 0.970106
H -0.955831 1.329652 1.616355
H -3.616945 -1.417855 1.331103
H -4.532161 -0.914247 -0.798019
H -3.330399 -0.360364 -2.498113
H -2.228508 -2.602274 -0.800024
H -3.537158 0.828855 2.590449
H -1.638126 3.320533 -0.224811
H -2.583124 2.542321 -1.500261
H -0.025746 1.873933 -0.532664
H -1.366692 -1.158958 2.037201
Na -0.487974 -0.182335 -2.333718

Reactant state of 2nd RO1' reaction of Manβ(1→3)Manβ
46

C 0.808074 -0.544721 -0.723058
O 1.111538 -0.677975 0.663308
C 2.463795 -1.03001 1.018242
C 3.369982 -1.157028 -0.21227
O 3.020331 -2.394468 -0.807629
C 3.210608 -0.022419 -1.248117
C 1.743111 0.501203 -1.327581
O 1.566822 1.733458 -0.652903
O 4.132739 0.994923 -0.916508
C 2.940107 -0.031011 2.064871
O 3.026005 1.298014 1.570242
O -0.500497 -0.064612 -0.809692
H 2.434275 -2.023651 1.481364
H 4.414148 -1.157429 0.127462
H 3.467563 -0.459063 -2.221183
H 1.460301 0.67119 -2.371013
H 0.898595 -1.51601 -1.224801
H 2.036385 1.677899 0.216132
H 4.070291 1.726721 -1.543933
H 3.733942 -2.712067 -1.374208
H 3.902825 -0.357722 2.474122
H 2.21659 -0.010967 2.884237
H 3.855854 1.407297 1.079522
C -3.501915 0.548039 -0.591754
O -3.75517 0.122266 0.710226
C -2.709395 -0.458585 1.505761
C -1.898075 -1.494473 0.715588
O -2.623101 -2.704432 0.594372
C -1.532727 -1.055988 -0.720808
C -2.768966 -0.506538 -1.425601
O -2.506023 -0.055954 -2.722968
C -1.910928 0.548347 2.340041
O -0.781909 1.149176 1.725446
O -2.769978 1.776007 -0.626315
H -3.272782 -1.029791 2.252993
H -0.982221 -1.736968 1.263753
H -3.467183 -1.342992 -1.530862
H -4.485678 0.727533 -1.032941
H -3.398185 2.493429 -0.473026
H -1.89399 0.688494 -2.67296
H -3.571618 -2.526259 0.55506
H -1.589746 0.032341 3.253093
H -2.585411 1.356313 2.633005
H -0.083886 0.481623 1.604151
H -1.19813 -1.951692 -1.256908
Na -0.600912 2.215422 -0.210506

Transition state of 3rd RO1' reaction of Manβ(1→3)Manβ
46

C -0.633384 -1.76075 -1.098329
O -1.747816 0.381836 -1.418945
C -3.082091 0.562248 -0.954854
C -3.333857 -0.237301 0.329188
O -4.729661 -0.411907 0.400071
C -2.624129 -1.59408 0.405984
C -1.067551 -1.485291 0.312395
O -0.570344 -0.362569 0.879573
O -2.9752 -2.183226 1.643939
C -3.344728 2.042102 -0.732117
O -2.371668 2.536623 0.211137
O 0.411728 -1.45615 -1.753954
H -3.759863 0.194497 -1.731139
H -2.975856 0.348767 1.189203
H -3.035178 -2.266013 -0.357844
H -0.725663 -2.445499 0.77697
H -1.272593 -2.410759 -1.702824
H -1.197893 0.450816 -0.603589
H -2.52142 -1.700156 2.349838
H -4.926175 -0.984356 1.154843
H -4.356005 2.171045 -0.333024
H -3.239981 2.583317 -1.677372
H -2.632496 3.432097 0.456049
C 3.60874 -1.017046 0.260087
O 4.004853 0.137537 -0.401489
C 2.993175 1.126741 -0.619605
C 1.59806 0.506437 -0.663456
O 0.722596 1.514526 -1.13843
C 1.678169 -0.801906 -1.406304
C 2.501982 -1.790539 -0.527767
O 1.667605 -2.505311 0.33075
C 2.998271 2.250277 0.432071
O 2.103076 2.017731 1.520412
O 3.114949 -0.718346 1.559323
H 3.231167 1.565035 -1.594363
H 1.290115 0.242409 0.344198
H 2.987407 -2.513513 -1.187729
H 4.501365 -1.640189 0.335794
H 3.76623 -0.970265 2.225592
H 1.552395 -1.985584 1.144038
H 0.148672 1.224893 -1.863349
H 2.655849 3.173396 -0.038528
H 4.021059 2.397267 0.792705
H 2.427509 1.246284 2.00991
H 2.125871 -0.682998 -2.395941
Na -0.260127 1.832565 1.0567

Reactant state of 3rd RO1' reaction of Manβ(1→3)Manβ
46

C -1.133394 -1.097586 -0.932668
O -1.676868 0.147413 -1.35612
C -3.084889 0.281483 -1.120507
C -3.571983 -0.247153 0.255709
O -4.909007 -0.631001 0.045018
C -2.732929 -1.383145 0.868709
C -1.251681 -1.104902 0.607509
O -0.938441 0.173427 1.197684
O -3.038125 -1.519092 2.235245
C -3.344725 1.771353 -1.219068
O -2.49745 2.384732 -0.235964
O 0.056462 -1.328795 -1.595073
H -3.636379 -0.261426 -1.89845
H -3.539092 0.58637 0.97536
H -3.017649 -2.332744 0.404311
H -0.598016 -1.847171 1.063336
H -1.772417 -1.90464 -1.313319
H -1.467681 0.837846 0.71646
H -2.63178 -0.786873 2.718411
H -5.29487 -0.902652 0.888571
H -4.401219 1.968097 -1.009045
H -3.086279 2.135723 -2.218127
H -2.730822 3.315567 -0.149249
C 3.635234 -1.233731 -0.37067
O 3.863806 -0.005627 -0.981782
C 2.90419 1.026057 -0.756516
C 1.490507 0.473915 -0.521811
O 0.624508 1.584083 -0.706988
C 1.338216 -0.757678 -1.394206
C 2.234568 -1.826785 -0.728922
O 1.549715 -2.306835 0.405156
C 3.325605 1.978066 0.367441
O 2.846835 1.581869 1.656348
O 3.738055 -1.130407 1.047241
H 2.881233 1.595406 -1.691367
H 1.392596 0.098538 0.504919
H 2.377083 -2.643702 -1.445097
H 4.429164 -1.883748 -0.743636
H 4.6647 -1.233028 1.30108
H 2.184803 -2.446869 1.120124
H -0.235882 1.304097 -1.077238
H 2.898302 2.966652 0.179853
H 4.415831 2.070984 0.371483
H 3.21623 0.704585 1.852556
H 1.708499 -0.546184 -2.403288
Na 0.585038 1.84797 1.621475

Transition state of 1st c0',3' reaction of Manβ(1→3)Manβ
46

C 0.612942 -1.123235 0.835181
O 1.608245 0.991637 0.576602
C 2.946594 0.534335 0.670037
C 3.303783 -0.369268 -0.53354
O 4.106207 -1.457288 -0.138031
C 2.043862 -0.914269 -1.205029
C 1.244489 -1.939092 -0.19775
O 0.275034 -2.511659 -1.058154
O 1.202151 0.059339 -1.621806
C 3.934041 1.682728 0.792107
O 5.211038 1.061438 0.82585
O -0.556985 -0.699484 0.554931
H 3.048454 -0.08549 1.576978
H 3.807277 0.231783 -1.304631
H 2.328538 -1.63811 -1.985726
H 1.973045 -2.649173 0.201758
H 1.045785 -0.917883 1.811777
H -0.113856 -3.296147 -0.651964
H 1.340955 0.836322 -0.438966
H 4.944293 -1.099196 0.189068
H 3.738334 2.258747 1.704139
H 3.831861 2.343942 -0.079423
H 5.90931 1.719991 0.903039
C -3.342107 -0.7256 0.423459
O -2.895392 0.080083 -0.656423
C -2.683452 1.450085 -0.306294
C -1.427647 1.557191 0.574857
O -1.62633 2.649391 1.435452
C -1.248615 0.26439 1.394638
C -2.589214 -0.367271 1.737522
O -2.43766 -1.475213 2.574867
C -2.535963 2.213322 -1.60769
O -1.507878 1.568123 -2.353608
O -3.044307 -2.047345 0.08984
H -3.545212 1.846007 0.250115
H -0.544222 1.690649 -0.057776
H -3.160133 0.396201 2.279989
H -4.423661 -0.588396 0.551214
H -3.799486 -2.462218 -0.344287
H -2.501132 -2.278237 2.038008
H -0.773902 2.999406 1.724545
H -2.273364 3.250963 -1.376449
H -3.477186 2.19107 -2.165635
H -1.032464 2.193966 -2.910561
H -0.648448 0.44714 2.290131
Na -0.900006 -0.61122 -1.955135

Reactant state of 1st c0',3' reaction of Manβ(1→3)Manβ
46

C -0.777274 -0.517502 -1.075428
O -1.463119 0.674343 -0.718052
C -2.878013 0.484401 -0.71851
C -3.283124 -0.347011 0.51539
O -4.399047 -1.151524 0.233936
C -2.130471 -1.280996 0.8999
C -1.404307 -1.751687 -0.355547
O -0.412269 -2.675983 0.059148
O -1.197264 -0.666919 1.784627
C -3.546976 1.845408 -0.728391
O -4.924961 1.568715 -0.549875
O 0.538308 -0.398208 -0.642277
H -3.183406 -0.06882 -1.620594
H -3.474816 0.320234 1.369747
H -2.54161 -2.145261 1.426067
H -2.133094 -2.234755 -1.017269
H -0.805898 -0.619328 -2.173029
H -0.082782 -3.182966 -0.692057
H -1.007237 0.233261 1.476016
H -5.136335 -0.566496 0.005354
H -3.352226 2.36113 -1.675317
H -3.1491 2.450382 0.098932
H -5.447407 2.37624 -0.601769
C 3.318425 -0.613929 -0.372653
O 2.861814 -0.026492 0.830888
C 2.591918 1.375927 0.826365
C 1.519352 1.769034 -0.216857
O 2.014435 2.936463 -0.836851
C 1.322962 0.639091 -1.235382
C 2.67435 0.049527 -1.614305
O 2.598189 -0.85159 -2.68078
C 2.150048 1.68868 2.2411
O 1.022897 0.850031 2.505963
O 2.900101 -1.957759 -0.289927
H 3.505595 1.939458 0.594325
H 0.556554 1.951584 0.275846
H 3.308807 0.882521 -1.937979
H 4.412622 -0.561255 -0.423782
H 3.65812 -2.531272 -0.127
H 2.332285 -1.710172 -2.324014
H 1.304207 3.402099 -1.294153
H 1.878421 2.748264 2.306729
H 2.96106 1.472138 2.943502
H 0.681816 1.024711 3.391369
H 0.825043 1.019745 -2.135215
Na 1.021398 -1.365066 1.415072

Transition state of 2nd c0',3' reaction of Manβ(1→3)Manβ
46

C 0.612965 -1.123215 0.835139
O 1.608302 0.991602 0.576691
C 2.946652 0.534288 0.670071
C 3.303809 -0.369187 -0.533613
O 4.106271 -1.457224 -0.138234
C 2.043876 -0.914147 -1.205105
C 1.244501 -1.939029 -0.197814
O 0.275024 -2.511493 -1.058261
O 1.202136 0.059459 -1.621794
C 3.934071 1.6827 0.792255
O 5.21111 1.061487 0.825823
O -0.55692 -0.699391 0.554855
H 3.04851 -0.085634 1.576944
H 3.807247 0.231954 -1.304671
H 2.328535 -1.637949 -1.985848
H 1.973047 -2.649147 0.201642
H 1.045731 -0.918013 1.811802
H -0.114005 -3.295922 -0.652082
H 1.34097 0.836414 -0.438868
H 4.944411 -1.099155 0.18875
H 3.73839 2.258575 1.704383
H 3.831803 2.344038 -0.079172
H 5.909335 1.720068 0.903196
C -3.342022 -0.725752 0.423589
O -2.89542 0.079937 -0.65634
C -2.683567 1.449957 -0.306247
C -1.427749 1.5572 0.574876
O -1.626581 2.649353 1.435498
C -1.248551 0.264407 1.394631
C -2.589078 -0.367352 1.737608
O -2.43736 -1.475282 2.574943
C -2.536248 2.213211 -1.607649
O -1.508205 1.5681 -2.353706
O -3.044174 -2.047488 0.089994
H -3.54533 1.84582 0.250202
H -0.544342 1.690788 -0.057759
H -3.160028 0.396071 2.280111
H -4.423577 -0.588599 0.551398
H -3.799329 -2.462385 -0.344153
H -2.500879 -2.278318 2.038109
H -0.774207 2.99949 1.724597
H -2.273688 3.250865 -1.376417
H -3.477534 2.190918 -2.165488
H -1.033096 2.193947 -2.910914
H -0.648328 0.447194 2.290079
Na -0.899914 -0.611089 -1.955278

Reactant state of 2nd c0',3' reaction of Manβ(1→3)Manβ
46

C -0.777165 -0.517551 -1.075461
O -1.462867 0.674277 -0.71785
C -2.877756 0.484546 -0.718447
C -3.283058 -0.347024 0.51528
O -4.399116 -1.151336 0.233726
C -2.130591 -1.281282 0.899658
C -1.40431 -1.751803 -0.355803
O -0.412429 -2.676214 0.059017
O -1.197324 -0.66757 1.784584
C -3.546555 1.845631 -0.728218
O -4.924616 1.569001 -0.550135
O 0.538482 -0.398427 -0.642452
H -3.183224 -0.068455 -1.620652
H -3.474666 0.320091 1.369768
H -2.541918 -2.145612 1.425573
H -2.132986 -2.234784 -1.017714
H -0.805895 -0.619198 -2.17308
H -0.081152 -3.181535 -0.692539
H -1.007423 0.232824 1.476493
H -5.136075 -0.56619 0.004366
H -3.351452 2.361534 -1.674972
H -3.148933 2.450414 0.099362
H -5.447096 2.376455 -0.602832
C 3.318381 -0.614087 -0.371787
O 2.861765 -0.026078 0.831437
C 2.591687 1.376316 0.826258
C 1.519367 1.768999 -0.217386
O 2.014521 2.936236 -0.837689
C 1.323301 0.638696 -1.235592
C 2.674853 0.049093 -1.613809
O 2.599264 -0.85226 -2.680115
C 2.149417 1.689606 2.240726
O 1.022079 0.851205 2.505588
O 2.899411 -1.957734 -0.288698
H 3.505373 1.939839 0.594244
H 0.55643 1.951622 0.274996
H 3.309395 0.882078 -1.937355
H 4.412611 -0.561938 -0.422754
H 3.657121 -2.531489 -0.125181
H 2.333179 -1.710775 -2.323332
H 1.304246 3.402083 -1.2947
H 1.877898 2.749248 2.3059
H 2.960196 1.473222 2.943448
H 0.681346 1.025783 3.391151
H 0.825724 1.019045 -2.13575
Na 1.020775 -1.365446 1.414885

Transition state of 3rd c0',3' reaction of Manβ(1→3)Manβ
46

C 0.613072 -1.123289 0.834821
O 1.608228 0.991271 0.576751
C 2.94669 0.534274 0.670095
C 3.303979 -0.369029 -0.533695
O 4.106397 -1.457117 -0.138355
C 2.044119 -0.913932 -1.205365
C 1.244658 -1.938947 -0.198229
O 0.275207 -2.511271 -1.058812
O 1.202353 0.059699 -1.621921
C 3.933876 1.682878 0.792402
O 5.211024 1.061887 0.825957
O -0.556786 -0.699374 0.554544
H 3.048705 -0.085701 1.576908
H 3.807511 0.232206 -1.304617
H 2.328867 -1.637582 -1.986213
H 1.973121 -2.649176 0.201188
H 1.045757 -0.918316 1.811564
H -0.114022 -3.295602 -0.65264
H 1.341025 0.836332 -0.438932
H 4.944533 -1.099103 0.188701
H 3.738071 2.25861 1.704596
H 3.831516 2.344289 -0.078954
H 5.909165 1.720616 0.902838
C -3.341906 -0.72606 0.423927
O -2.895567 0.079686 -0.656077
C -2.683906 1.449725 -0.305961
C -1.427879 1.557137 0.574856
O -1.626675 2.649199 1.435603
C -1.248347 0.264316 1.394501
C -2.588696 -0.367642 1.737782
O -2.436573 -1.475592 2.575021
C -2.537095 2.213134 -1.607292
O -1.508901 1.568347 -2.353416
O -3.044038 -2.047762 0.090278
H -3.545601 1.845384 0.250737
H -0.544656 1.690921 -0.057998
H -3.159623 0.395656 2.280485
H -4.423443 -0.588952 0.551947
H -3.799024 -2.462522 -0.344295
H -2.50019 -2.278623 2.038192
H -0.774281 2.999485 1.724465
H -2.274708 3.250824 -1.375987
H -3.478513 2.190629 -2.164916
H -1.036727 2.193824 -2.913535
H -0.64794 0.447149 2.289814
Na -0.899844 -0.610683 -1.955182

Reactant state of 3rd c0',3' reaction of Manβ(1→3)Manβ
46

C -0.777073 -0.517776 -1.075621
O -1.462669 0.674084 -0.717936
C -2.877545 0.484474 -0.718513
C -3.282919 -0.347154 0.515166
O -4.399039 -1.151392 0.233548
C -2.130533 -1.28151 0.899512
C -1.404229 -1.752015 -0.35595
O -0.412313 -2.676399 0.058881
O -1.197296 -0.667911 1.784515
C -3.546247 1.84561 -0.72822
O -4.924358 1.569059 -0.550284
O 0.538629 -0.398789 -0.642765
H -3.183109 -0.068448 -1.620738
H -3.474534 0.319908 1.36969
H -2.541936 -2.145848 1.425357
H -2.1329 -2.235026 -1.017845
H -0.805933 -0.619397 -2.173245
H -0.081094 -3.181762 -0.692671
H -1.007445 0.232555 1.476604
H -5.135969 -0.56616 0.00431
H -3.351037 2.361576 -1.674917
H -3.148691 2.450314 0.099447
H -5.446784 2.376534 -0.603074
C 3.318534 -0.613722 -0.371328
O 2.861648 -0.025671 0.831782
C 2.5911 1.376644 0.826417
C 1.518896 1.768957 -0.217489
O 2.013917 2.936261 -0.837772
C 1.323336 0.638552 -1.235681
C 2.675142 0.049262 -1.613513
O 2.600116 -0.852129 -2.679816
C 2.148477 1.689958 2.240758
O 1.021209 0.851418 2.505514
O 2.899887 -1.957489 -0.288315
H 3.504658 1.94043 0.594537
H 0.555816 1.951399 0.274681
H 3.309556 0.882406 -1.936905
H 4.412761 -0.561381 -0.422148
H 3.65767 -2.530991 -0.124237
H 2.333872 -1.710639 -2.323131
H 1.303662 3.401824 -1.29511
H 1.876787 2.749564 2.305813
H 2.959169 1.473757 2.943637
H 0.680652 1.025701 3.391205
H 0.825911 1.018729 -2.135997
Na 1.020858 -1.365552 1.41479

Transition state of 1st RO2' reaction of Manβ(1→3)Manβ
46

C 0.767296 0.958931 -1.21595
O 1.885119 -1.563728 0.246634
C 2.976786 -1.074655 0.029871
C 3.390699 0.343564 0.435782
O 3.986749 0.998119 -0.661053
C 2.278058 1.277943 0.923758
C 1.39783 1.889698 -0.182521
O 0.388685 2.662377 0.43814
O 1.478861 0.630996 1.923243
C 4.022755 -1.900176 -0.70125
O 5.300835 -1.339887 -0.474878
O -0.306341 0.228427 -0.616856
H 1.501094 0.265688 -1.642653
H 4.107222 0.208891 1.261766
H 2.782544 2.133602 1.384365
H 2.042895 2.579358 -0.73342
H 0.371702 1.58211 -2.02341
H -0.169616 2.053362 0.952184
H 1.549632 1.119739 2.749984
H 4.862309 0.611067 -0.812717
H 3.754616 -1.894165 -1.767859
H 3.945169 -2.927513 -0.330259
H 5.988772 -1.913003 -0.832036
C -3.026823 1.140049 0.376349
O -3.732592 -0.05225 0.531792
C -3.349637 -1.267578 -0.133111
C -2.165771 -1.220254 -1.143292
O -2.553481 -1.824638 -2.358092
C -1.512586 0.155985 -1.381589
C -2.461683 1.29747 -1.032228
O -1.929116 2.568488 -1.252131
C -3.078762 -2.269789 0.982901
O -2.063567 -1.744999 1.838806
O -1.976943 1.234624 1.351597
H -4.233155 -1.594006 -0.692987
H -1.36519 -1.871753 -0.775197
H -3.32952 1.210239 -1.70149
H -3.744689 1.942503 0.565146
H -2.326541 1.703735 2.120061
H -1.173288 2.74089 -0.662722
H -3.300236 -1.355167 -2.752515
H -2.716083 -3.217267 0.57835
H -3.989893 -2.453426 1.559572
H -2.447006 -0.94605 2.231704
H -1.257723 0.206578 -2.446329
Na -0.009785 -0.959342 1.298229

Reactant state of 1st RO2' reaction of Manβ(1→3)Manβ
46

C -0.974489 -0.927132 0.066669
O -1.17319 0.466521 0.39849
C -2.082376 1.097786 -0.522451
C -3.453703 0.428545 -0.445012
O -4.274638 1.050433 -1.399606
C -3.339269 -1.073036 -0.690186
C -2.290824 -1.677307 0.244806
O -2.747338 -1.650963 1.581303
O -4.59639 -1.680699 -0.534276
C -2.205643 2.56149 -0.157969
O -0.905213 3.159124 -0.16202
O -0.046372 -1.476237 0.935559
H -1.680951 1.004741 -1.543338
H -3.858912 0.586395 0.570703
H -3.039739 -1.229618 -1.735124
H -2.131353 -2.729864 -0.007095
H -0.651573 -0.990583 -0.978847
H -2.603157 -0.770861 1.953555
H -4.727414 -1.867828 0.407778
H -5.082838 0.524976 -1.49079
H -2.653102 2.659739 0.839767
H -2.86473 3.036102 -0.888462
H -0.965207 4.082724 -0.429214
C 3.15969 0.293981 1.194547
O 3.942986 -0.45125 0.316045
C 3.299212 -1.012842 -0.831093
C 1.760617 -1.028506 -0.740209
O 1.236361 -1.902293 -1.71767
C 1.3548 -1.438456 0.674226
C 2.050332 -0.581223 1.781616
O 1.132308 0.267924 2.444759
C 3.800194 -0.305272 -2.078967
O 3.397901 1.060045 -2.000849
O 2.564932 1.409516 0.554622
H 3.635457 -2.057713 -0.884009
H 1.366167 -0.040635 -0.996926
H 2.517171 -1.255328 2.507723
H 3.841139 0.63686 1.977384
H 3.059206 1.591992 -0.270612
H 0.390663 -0.281698 2.736889
H 1.518938 -2.81144 -1.547749
H 3.366416 -0.785541 -2.963167
H 4.891496 -0.38518 -2.108662
H 3.806147 1.566258 -2.712175
H 1.690151 -2.476297 0.805848
Na 0.495819 1.995242 1.131801

Transition state of 2nd RO2' reaction of Manβ(1→3)Manβ
46

C -0.638632 0.992354 1.328178
O -1.773382 -1.575968 -0.205383
C -2.840119 -1.054402 0.051236
C -3.241193 0.334434 -0.444058
O -4.017026 1.00771 0.519406
C -2.116842 1.305734 -0.81887
C -1.369363 1.924218 0.368166
O -0.47187 2.836521 -0.244512
O -1.238307 0.699037 -1.758039
C -3.863805 -1.808954 0.889375
O -5.169647 -1.486846 0.448579
O 0.420368 0.287917 0.691821
H -1.347614 0.277871 1.769634
H -3.814716 0.136561 -1.364761
H -2.625029 2.150951 -1.298473
H -2.122003 2.4584 0.962743
H -0.233616 1.601543 2.146041
H 0.13922 3.205522 0.411375
H -0.839484 1.392047 -2.300882
H -4.885981 0.583071 0.559167
H -3.713371 -1.502777 1.935688
H -3.640874 -2.875759 0.799937
H -5.82258 -2.014249 0.923139
C 2.613047 1.035782 -0.939374
O 2.35813 -0.367899 -1.118734
C 3.022399 -1.192183 -0.170351
C 2.377072 -1.020713 1.218473
O 3.434627 -1.142265 2.144799
C 1.707535 0.360711 1.30386
C 2.59429 1.367246 0.585011
O 2.102523 2.668368 0.847801
C 2.92975 -2.613413 -0.677193
O 1.5378 -2.901353 -0.839761
O 1.674176 1.728528 -1.660513
H 4.083872 -0.913062 -0.090739
H 1.604579 -1.784658 1.37575
H 3.606304 1.257737 0.997076
H 3.600028 1.240596 -1.375702
H 0.938648 2.016092 -1.082611
H 2.734654 3.332258 0.543854
H 3.097392 -1.349085 3.024045
H 3.382839 -3.290063 0.055996
H 3.453486 -2.697397 -1.634804
H 1.428219 -3.816133 -1.136037
H 1.609896 0.64841 2.358241
Na 0.132923 -1.079341 -1.304464

Reactant state of 2nd RO2' reaction of Manβ(1→3)Manβ
46

C -0.974401 -0.92708 0.066727
O -1.173274 0.466568 0.398493
C -2.082558 1.097743 -0.522474
C -3.453805 0.428362 -0.444982
O -4.274835 1.050129 -1.399556
C -3.33918 -1.073206 -0.690168
C -2.29068 -1.677385 0.244805
O -2.747218 -1.651097 1.581306
O -4.59624 -1.681004 -0.534259
C -2.205923 2.561458 -0.158005
O -0.905596 3.159251 -0.162507
O -0.046291 -1.476038 0.935742
H -1.681113 1.004717 -1.543349
H -3.858974 0.586184 0.570758
H -3.039652 -1.229717 -1.73512
H -2.131103 -2.729928 -0.00709
H -0.651364 -0.990541 -0.978754
H -2.603142 -0.770987 1.953579
H -4.727217 -1.868164 0.407799
H -5.083041 0.524652 -1.490606
H -2.653067 2.659699 0.839875
H -2.865362 3.035944 -0.888268
H -0.965417 4.082568 -0.430703
C 3.15966 0.294434 1.194495
O 3.943096 -0.450957 0.31623
C 3.299415 -1.012908 -0.830816
C 1.760812 -1.028694 -0.74
O 1.236657 -1.902746 -1.717259
C 1.354947 -1.438308 0.674519
C 2.050391 -0.5808 1.781724
O 1.132251 0.268308 2.444735
C 3.800369 -0.305594 -2.078842
O 3.397906 1.059701 -2.001101
O 2.564893 1.40978 0.554295
H 3.635765 -2.057758 -0.883469
H 1.366297 -0.040907 -0.996946
H 2.517343 -1.254701 2.507956
H 3.841006 0.637549 1.977322
H 3.058867 1.591885 -0.271221
H 0.389986 -0.281178 2.735598
H 1.51916 -2.811859 -1.547024
H 3.366704 -0.786125 -2.962957
H 4.891682 -0.385372 -2.108446
H 3.806699 1.565933 -2.712101
H 1.690258 -2.476129 0.806408
Na 0.495511 1.99554 1.131447

Transition state of 3rd RO2' reaction of Manβ(1→3)Manβ
46

C 0.76727 0.959542 -1.21608
O 1.884659 -1.563523 0.247266
C 2.976425 -1.074771 0.030232
C 3.390645 0.343604 0.435291
O 3.986135 0.997639 -0.662166
C 2.278351 1.278259 0.923497
C 1.397833 1.890133 -0.18252
O 0.388651 2.662474 0.438552
O 1.479516 0.631416 1.923341
C 4.022245 -1.900999 -0.700328
O 5.300346 -1.340271 -0.475199
O -0.306183 0.228769 -0.617009
H 1.501096 0.266479 -1.643045
H 4.107621 0.209246 1.260928
H 2.783163 2.133832 1.383908
H 2.042632 2.580081 -0.733365
H 0.371509 1.58286 -2.023352
H -0.169773 2.053141 0.952079
H 1.548954 1.121499 2.749407
H 4.86159 0.610462 -0.814116
H 3.753642 -1.896404 -1.76682
H 3.944976 -2.927884 -0.328004
H 5.988184 -1.913576 -0.832245
C -3.026741 1.139673 0.376483
O -3.732605 -0.052603 0.531484
C -3.349297 -1.267859 -0.133383
C -2.16536 -1.220215 -1.143476
O -2.552854 -1.824644 -2.35832
C -1.512452 0.156186 -1.381681
C -2.461687 1.29749 -1.032082
O -1.929296 2.568627 -1.251759
C -3.078384 -2.270085 0.982605
O -2.063523 -1.745124 1.838789
O -1.976794 1.233886 1.351763
H -4.232715 -1.594421 -0.693334
H -1.36464 -1.871526 -0.775345
H -3.329554 1.210287 -1.701303
H -3.744547 1.942105 0.565593
H -2.326308 1.702964 2.120288
H -1.173444 2.741034 -0.662394
H -3.299817 -1.35545 -2.752678
H -2.71536 -3.217434 0.578063
H -3.989601 -2.454017 1.559046
H -2.447254 -0.946312 2.231631
H -1.257662 0.206952 -2.446429
Na -0.010018 -0.958438 1.298744

Reactant state of 3rd RO2' reaction of Manβ(1→3)Manβ
46

C -0.974237 -0.926559 0.068212
O -1.173647 0.467322 0.398443
C -2.082988 1.097115 -0.523372
C -3.453982 0.427216 -0.445649
O -4.274957 1.047733 -1.401102
C -3.338669 -1.074527 -0.689335
C -2.29023 -1.677283 0.246627
O -2.747275 -1.649883 1.582917
O -4.595522 -1.682701 -0.533281
C -2.20725 2.561157 -0.160521
O -0.907168 3.159493 -0.164674
O -0.046154 -1.474269 0.938101
H -1.68126 1.003223 -1.544065
H -3.859548 0.585836 0.5698
H -3.038685 -1.231965 -1.734012
H -2.130025 -2.729987 -0.004201
H -0.650915 -0.991095 -0.977112
H -2.60336 -0.769476 1.954544
H -4.726747 -1.869003 0.408904
H -5.082935 0.521866 -1.491869
H -2.655076 2.660322 0.836934
H -2.866322 3.034582 -0.891793
H -0.966987 4.082369 -0.43442
C 3.159745 0.2967 1.193674
O 3.943232 -0.450129 0.316713
C 3.29955 -1.014331 -0.829225
C 1.760956 -1.02984 -0.738411
O 1.23675 -1.905515 -1.714195
C 1.355052 -1.437012 0.6768
C 2.050558 -0.577559 1.782473
O 1.132418 0.272634 2.444036
C 3.800573 -0.309401 -2.07858
O 3.398311 1.056091 -2.003329
O 2.564878 1.41088 0.551402
H 3.635832 -2.0593 -0.879834
H 1.366555 -0.042461 -0.997048
H 2.517615 -1.250169 2.50983
H 3.841057 0.641295 1.975878
H 3.05931 1.591645 -0.27411
H 0.390267 -0.276386 2.736059
H 1.518899 -2.814403 -1.542216
H 3.366821 -0.791497 -2.961793
H 4.891874 -0.389389 -2.108065
H 3.806828 1.560846 -2.715533
H 1.690361 -2.474596 0.810508
Na 0.495915 1.998222 1.128838

Transition state of 1st RO reaction of Manβ(1→4)Manβ
46

C 1.00497 -1.47144 -0.15457
O 1.005789 -0.378288 0.736112
C 2.278779 -0.12078 1.315953
C 3.254504 0.275495 0.212162
O 4.492451 0.545364 0.818856
C 3.350582 -0.861451 -0.799405
C 1.955383 -1.217461 -1.328087
O 1.462419 -0.139737 -2.104384
O 4.162643 -0.390732 -1.864237
C 2.079685 0.954461 2.367214
O 1.4918 2.128219 1.793885
O -0.580641 1.375938 -1.141138
H 2.65498 -1.023711 1.824859
H 2.859834 1.164815 -0.302688
H 3.809034 -1.730213 -0.306971
H 1.993393 -2.12946 -1.939554
H 2.168816 0.125977 -2.713505
H 4.670888 -1.10996 -2.257887
H 5.143035 0.724966 0.126555
H 3.039524 1.186153 2.833897
H 1.390678 0.584436 3.131448
H 2.156672 2.819861 1.705876
C -3.512656 0.890206 -0.075115
O -3.578397 -0.572946 0.473316
C -2.634671 -1.541504 -0.071085
C -1.317243 -0.795151 -0.318273
O -0.297616 -1.729999 -0.618956
C -1.457059 0.279152 -1.403503
C -2.890056 0.830365 -1.464947
O -2.954526 2.085677 -2.076616
C -2.4907 -2.622683 0.981609
O -1.901709 -2.006394 2.113775
O -2.789343 1.367171 0.960198
H -3.071682 -1.958478 -0.98366
H -1.08502 -0.321479 0.630773
H -3.503889 0.147945 -2.066675
H -4.559775 1.209772 -0.108385
H -3.081609 0.067811 1.290137
H -2.114889 2.540259 -1.921954
H 0.298654 1.103782 -1.465733
H -1.854784 -3.41961 0.581161
H -3.483347 -3.025478 1.209888
H -2.063786 -2.537847 2.900369
H -1.193336 -0.161735 -2.371495
H 1.344199 -2.372048 0.382447
Na -0.668151 2.022254 1.047537

Reactant state of 1st RO reaction of Manβ(1→4)Manβ
46

C -0.963611 -1.409499 -0.063386
O -0.996879 -0.159062 -0.734581
C -2.284807 0.11437 -1.2865
C -3.275986 0.292441 -0.14025
O -4.522774 0.611126 -0.703904
C -3.330651 -0.99758 0.670362
C -1.92357 -1.397773 1.129706
O -1.460171 -0.462576 2.088586
O -4.151118 -0.735432 1.798539
C -2.157116 1.33511 -2.175674
O -1.609547 2.435529 -1.440472
O 0.675833 1.120094 1.496141
H -2.607315 -0.727485 -1.9202
H -2.914265 1.10053 0.513757
H -3.765329 -1.78589 0.040276
H -1.93094 -2.405495 1.567155
H -2.177 -0.317711 2.725418
H -4.632023 -1.526512 2.069769
H -5.180085 0.653639 0.003845
H -3.138121 1.584207 -2.585465
H -1.471183 1.113257 -2.99846
H -2.278846 3.113315 -1.298537
C 3.569095 0.710928 0.032836
O 3.654409 -0.551809 -0.561585
C 2.665801 -1.532595 -0.213444
C 1.381183 -0.808152 0.183519
O 0.326613 -1.733639 0.370943
C 1.60102 0.0323 1.441044
C 3.033563 0.607799 1.45731
O 3.130431 1.818986 2.143617
C 2.451763 -2.415606 -1.427238
O 1.720015 -1.673519 -2.390844
O 2.715201 1.58546 -0.719045
H 3.04369 -2.153288 0.612012
H 1.159686 -0.183244 -0.676418
H 3.686173 -0.118038 1.95777
H 4.579336 1.126435 0.031263
H 3.156926 1.761693 -1.560262
H 2.248706 2.210287 2.205688
H -0.168203 0.751441 1.824319
H 1.897113 -3.310172 -1.120847
H 3.435131 -2.711646 -1.808235
H 1.85529 -2.060331 -3.261753
H 1.446843 -0.606389 2.318296
H -1.2848 -2.188226 -0.773602
Na 0.416638 1.949809 -0.572242

Transition state of 2nd RO reaction of Manβ(1→4)Manβ
46

C 1.316657 -0.208674 1.700975
O 1.295297 1.012882 1.000083
C 2.299442 1.410609 0.056386
C 2.862762 0.277665 -0.831607
O 4.250325 0.247559 -0.566733
C 2.22768 -1.080337 -0.537554
C 2.178755 -1.269408 0.972951
O 1.737719 -2.579559 1.277585
O 0.966169 -1.106439 -1.176736
C 1.643247 2.511218 -0.772483
O 0.489797 2.024497 -1.449316
O -0.775946 -2.677887 0.188621
H 3.153279 1.833594 0.600676
H 2.670611 0.512695 -1.887813
H 2.872217 -1.870102 -0.947798
H 3.214774 -1.128569 1.309229
H 2.165949 -2.911738 2.075145
H 0.434002 -1.859878 -0.844814
H 4.720455 -0.217896 -1.269132
H 2.360174 2.865952 -1.5189
H 1.377709 3.36132 -0.135192
H -0.310019 2.300417 -0.971978
C -3.07275 0.056681 -0.843762
O -3.05914 0.847988 0.50361
C -2.116429 0.523528 1.568604
C -1.011799 -0.359093 0.971844
O -0.019202 -0.607905 1.950311
C -1.688903 -1.660026 0.537874
C -2.564679 -1.400879 -0.710134
O -1.813525 -1.618338 -1.89653
C -1.645953 1.862487 2.111047
O -0.953534 2.584419 1.110418
O -2.277867 0.922993 -1.490493
H -2.671904 -0.019145 2.340781
H -0.569511 0.146943 0.113824
H -3.425851 -2.076911 -0.697414
H -4.133944 0.097167 -1.111397
H -2.525353 1.503764 -0.266699
H -1.601509 -2.560984 -1.941036
H -0.162164 -2.837582 0.929342
H -1.017285 1.687494 2.990621
H -2.514824 2.45164 2.414585
H -0.033936 2.277756 1.162334
H -2.32877 -2.014212 1.358067
H 1.720521 -0.049516 2.708153
Na -0.425779 0.193044 -2.487455

Reactant state of 2nd RO reaction of Manβ(1→4)Manβ
46

C 1.293076 0.287847 -1.704295
O 1.298364 -0.964932 -1.057494
C 2.336611 -1.393629 -0.168769
C 2.917752 -0.299259 0.756064
O 4.296772 -0.24624 0.453973
C 2.26331 1.065078 0.548375
C 2.163284 1.324361 -0.948715
O 1.703355 2.644874 -1.171086
O 1.024495 1.048911 1.231313
C 1.71179 -2.5397 0.624294
O 0.532708 -2.118046 1.301677
O -0.754009 2.661655 0.043386
H 3.178449 -1.781096 -0.756979
H 2.758665 -0.585981 1.805442
H 2.913917 1.841297 0.97471
H 3.19087 1.214655 -1.321264
H 2.033641 2.985891 -2.010852
H 0.463529 1.800571 0.935844
H 4.780847 0.207207 1.154804
H 2.427488 -2.894491 1.370643
H 1.477881 -3.378721 -0.04042
H -0.218371 -2.236426 0.690466
C -3.112963 -0.120278 0.677002
O -3.295993 -0.460127 -0.658338
C -2.161477 -0.409936 -1.547886
C -1.022716 0.403855 -0.921371
O -0.046669 0.681907 -1.913244
C -1.674377 1.681361 -0.393297
C -2.554658 1.312658 0.821987
O -1.808295 1.383413 2.029728
C -1.784921 -1.813634 -2.001746
O -0.986739 -2.527565 -1.065367
O -2.230575 -1.025025 1.331619
H -2.512916 0.13437 -2.43418
H -0.56198 -0.154436 -0.109546
H -3.403921 2.000949 0.878254
H -4.104477 -0.194414 1.129711
H -2.568102 -1.918644 1.172565
H -1.585481 2.312472 2.180446
H -0.159863 2.901792 -0.691587
H -1.259097 -1.743137 -2.960315
H -2.706748 -2.378933 -2.159297
H -0.071001 -2.309223 -1.29481
H -2.310578 2.10446 -1.181764
H 1.68166 0.176298 -2.724117
Na -0.323637 -0.338855 2.430278

Transition state of 3rd RO reaction of Manβ(1→4)Manβ
46

C 1.571146 -0.711044 1.446189
O 1.179038 0.620417 1.573805
C 1.8262 1.592641 0.742217
C 2.157004 0.971873 -0.609565
O 2.582057 2.021013 -1.455635
C 3.171758 -0.154041 -0.443469
C 3.000355 -0.861492 0.930014
O 3.371382 -2.210103 0.862797
O 2.951838 -1.047418 -1.524852
C 0.894466 2.790178 0.613014
O -0.125071 2.584981 -0.368687
O 0.119162 -1.374083 -2.102545
H 2.74789 1.932487 1.237155
H 1.241821 0.522647 -1.013973
H 4.184606 0.265747 -0.502711
H 3.666622 -0.389446 1.659622
H 2.675087 -2.690877 0.392818
H 3.684411 -1.676243 -1.572398
H 2.903145 1.649547 -2.288329
H 1.476436 3.679588 0.359196
H 0.392947 2.950678 1.569669
H 0.295039 2.707063 -1.233081
C -3.324434 -0.922264 -0.775475
O -2.90276 -1.336756 0.742228
C -1.55939 -1.333058 1.274271
C -0.535471 -0.966809 0.19478
O 0.751325 -1.448233 0.52656
C -0.889697 -1.603212 -1.150785
C -2.124458 -0.90422 -1.727074
O -1.789759 0.445764 -2.009194
C -1.573408 -0.366274 2.446841
O -1.936754 0.921787 1.9386
O -3.840249 0.237946 -0.391205
H -1.346496 -2.346474 1.631385
H -0.499556 0.120094 0.06961
H -2.420075 -1.408647 -2.654524
H -4.02561 -1.734645 -0.992409
H -3.476077 -0.332743 0.759318
H -0.989101 0.402258 -2.554914
H 1.00429 -1.56199 -1.742083
H -0.587844 -0.325501 2.918164
H -2.315599 -0.703639 3.178374
H -2.095993 1.509499 2.686875
H -1.085717 -2.678667 -1.021412
H 1.469588 -1.171725 2.435301
Na -2.315083 1.887709 -0.300216

Reactant state of 3rd RO reaction of Manβ(1→4)Manβ
46

C 1.523922 -0.732364 1.452742
O 1.188535 0.619962 1.561642
C 1.905613 1.548615 0.739324
C 2.222811 0.903418 -0.603705
O 2.712838 1.924111 -1.451275
C 3.176069 -0.270196 -0.415295
C 2.955633 -0.952574 0.965904
O 3.260786 -2.318666 0.913025
O 2.922898 -1.158995 -1.493064
C 1.051832 2.798597 0.579556
O 0.043928 2.647656 -0.424221
O 0.028806 -1.28051 -2.142959
H 2.836212 1.838766 1.249383
H 1.290563 0.499905 -1.016698
H 4.209778 0.097072 -0.466608
H 3.636019 -0.505655 1.698133
H 2.53291 -2.768907 0.460433
H 3.608458 -1.840288 -1.509815
H 3.023733 1.529719 -2.277219
H 1.692473 3.648104 0.330549
H 0.541634 3.004539 1.523141
H 0.492762 2.731449 -1.278923
C -3.314591 -0.945192 -0.533615
O -2.894715 -1.584196 0.639102
C -1.628949 -1.27596 1.23207
C -0.567334 -0.934382 0.179113
O 0.711162 -1.4369 0.511304
C -0.937114 -1.562071 -1.157878
C -2.228605 -0.899481 -1.64221
O -1.935606 0.448803 -1.993989
C -1.741005 -0.247211 2.348704
O -1.89597 1.084218 1.840719
O -3.756036 0.371631 -0.280675
H -1.32608 -2.216265 1.707511
H -0.494537 0.149194 0.045142
H -2.601693 -1.423929 -2.527534
H -4.158071 -1.554431 -0.866591
H -4.476983 0.319085 0.360877
H -1.167481 0.393576 -2.584724
H 0.92252 -1.514503 -1.833687
H -0.82588 -0.296222 2.9495
H -2.59847 -0.508048 2.978318
H -1.68796 1.693319 2.558472
H -1.087832 -2.643647 -1.038534
H 1.372563 -1.174961 2.44424
Na -2.121575 1.947093 -0.291863

Transition state of 1st c2 reaction of Manβ(1→4)Manβ
46

C -0.592298 -0.825245 -1.263762
O -1.586036 0.132906 -1.500522
C -2.776211 0.077681 -0.704872
C -2.452732 -0.40754 0.708522
O -3.63977 -0.325534 1.454846
C -1.902059 -1.836615 0.651039
C -1.191361 -2.106775 -0.691223
O -0.215881 -3.103365 -0.47137
O -1.028269 -2.007318 1.765521
C -3.375337 1.467542 -0.731945
O -2.389376 2.357875 -0.225334
O 2.056106 1.079181 1.323112
H -3.5014 -0.611708 -1.162561
H -1.678018 0.258633 1.119273
H -2.747332 -2.52785 0.750228
H -1.945456 -2.459668 -1.40716
H 0.221692 -3.312353 -1.309068
H -0.7876 -2.943275 1.819314
H -3.47174 -0.563821 2.374694
H -4.279345 1.472481 -0.116059
H -3.631048 1.722546 -1.767144
H -2.78393 3.222865 -0.065724
C 1.398816 3.574396 0.244963
O 2.037957 -1.899618 -1.926279
C 2.637341 -0.905076 -1.090662
C 1.562542 0.185685 -0.887525
O 0.370564 -0.354073 -0.333702
C 2.087412 1.263713 0.049081
C 0.571317 2.577883 -0.247782
O 0.169022 2.653814 -1.546842
C 3.17334 -1.563633 0.177388
O 2.144281 -2.140539 0.967277
O 1.877296 3.579863 1.459924
H 3.479644 -0.430213 -1.612461
H 1.348112 0.636557 -1.864158
H -0.062458 2.002631 0.419956
H 1.774598 4.353521 -0.419677
H 2.717258 -2.320417 -2.467051
H -0.730392 2.284118 -1.58742
H 1.905755 2.621023 1.78928
H 3.686211 -0.829493 0.803828
H 3.900299 -2.333093 -0.113732
H 1.517401 -2.609063 0.387699
H 2.972206 1.766607 -0.375159
H -0.101852 -1.005741 -2.22306
Na 0.816875 -0.653811 2.001252

Reactant state of 1st c2 reaction of Manβ(1→4)Manβ
46

C -0.633282 -0.952933 -1.231508
O -1.576745 0.030094 -1.55105
C -2.756229 0.11222 -0.746938
C -2.455244 -0.279899 0.700435
O -3.630459 -0.065553 1.440249
C -2.004107 -1.743258 0.745875
C -1.292972 -2.15399 -0.561234
O -0.357758 -3.168081 -0.247044
O -1.15808 -1.90641 1.883678
C -3.287017 1.52229 -0.885707
O -2.28783 2.418546 -0.407857
O 2.110682 0.959822 1.236356
H -3.52669 -0.56788 -1.142675
H -1.636319 0.361281 1.060647
H -2.89803 -2.364774 0.876194
H -2.05448 -2.532961 -1.254298
H -0.136715 -3.664043 -1.045639
H -0.98768 -2.853453 1.990566
H -3.471728 -0.246415 2.374727
H -4.207019 1.607766 -0.30059
H -3.499366 1.715457 -1.943255
H -2.68124 3.288394 -0.272896
C 1.278324 3.686124 0.51978
O 2.029442 -1.94477 -1.933784
C 2.616885 -0.908184 -1.14336
C 1.501127 0.130731 -0.946175
O 0.349197 -0.458002 -0.336413
C 1.890502 1.346766 -0.12107
C 0.776877 2.410825 -0.146524
O 0.346539 2.691962 -1.445709
C 3.217051 -1.539222 0.109268
O 2.226788 -2.177765 0.905522
O 2.075467 3.678011 1.432589
H 3.421741 -0.408238 -1.700473
H 1.225614 0.507339 -1.938293
H -0.056055 2.032639 0.466807
H 0.871364 4.630007 0.113342
H 2.714465 -2.396508 -2.441075
H -0.602337 2.486863 -1.491311
H 2.390873 1.765744 1.707456
H 3.703253 -0.788052 0.733561
H 3.972944 -2.272332 -0.202019
H 1.623465 -2.666017 0.31825
H 2.814138 1.768587 -0.542426
H -0.147698 -1.235925 -2.167737
Na 0.821642 -0.765375 1.930163

Transition state of 2nd c2 reaction of Manβ(1→4)Manβ
46

C -0.376076 -0.927789 -1.275252
O -1.505847 -0.124867 -1.526974
C -2.69234 -0.40607 -0.764709
C -2.311067 -0.817449 0.663679
O -3.447537 -0.998201 1.467262
C -1.530554 -2.128495 0.63793
C -0.774987 -2.285976 -0.700352
O 0.352536 -3.101581 -0.464162
O -0.639992 -2.13267 1.746666
C -3.549507 0.84072 -0.795685
O -2.908935 1.811928 0.018762
O 1.771132 1.471514 1.325918
H -3.255125 -1.217537 -1.248083
H -1.658963 -0.030683 1.067517
H -2.248968 -2.949048 0.748
H -1.450821 -2.76283 -1.422375
H 0.790393 -3.300259 -1.303182
H -0.294458 -3.028978 1.85652
H -3.727554 -0.147986 1.828211
H -4.548929 0.595962 -0.418012
H -3.638778 1.182347 -1.83395
H -3.365817 2.657745 -0.055835
C 0.611397 3.727961 0.18648
O 2.4435 -1.490628 -1.872741
C 2.822808 -0.400612 -1.029775
C 1.551205 0.458475 -0.874903
O 0.480729 -0.306339 -0.339176
C 1.800457 1.641739 0.046923
C 0.039176 2.569934 -0.318664
O -0.340287 2.555128 -1.626063
C 3.42663 -0.941 0.262772
O 2.498628 -1.704665 1.017489
O 1.015961 3.83992 1.420926
H 3.576563 0.224338 -1.528584
H 1.285079 0.833566 -1.870999
H -0.47781 1.882949 0.343802
H 0.839193 4.565316 -0.475094
H 3.213352 -1.805565 -2.361841
H -0.953955 1.810088 -1.747706
H 1.269695 2.903661 1.747471
H 3.763951 -0.119418 0.90034
H 4.299985 -1.555062 0.006285
H 1.979903 -2.270657 0.417446
H 2.569401 2.316906 -0.362975
H 0.148575 -1.035384 -2.228223
Na 0.911194 -0.48131 2.033396

Reactant state of 2nd c2 reaction of Manβ(1→4)Manβ
46

C -0.567635 -0.988996 -1.230653
O -1.55987 -0.050642 -1.550547
C -2.749983 -0.060648 -0.755637
C -2.42172 -0.416849 0.7008
O -3.567011 -0.354527 1.513928
C -1.882091 -1.844344 0.760756
C -1.166116 -2.220605 -0.556249
O -0.176304 -3.184871 -0.254287
O -1.007281 -1.948603 1.878137
C -3.387214 1.301886 -0.909797
O -2.506072 2.257962 -0.326849
O 2.059795 1.09219 1.229509
H -3.460934 -0.802076 -1.151965
H -1.642213 0.273927 1.050325
H -2.73815 -2.512024 0.913461
H -1.912436 -2.637732 -1.243926
H 0.052527 -3.676033 -1.05343
H -0.833196 -2.887144 2.033321
H -3.668103 0.531928 1.879021
H -4.362364 1.301636 -0.410723
H -3.531555 1.503565 -1.977201
H -2.898361 3.137041 -0.390334
C 1.042065 3.757086 0.480587
O 2.154589 -1.821188 -1.937237
C 2.67721 -0.760253 -1.134931
C 1.50176 0.211757 -0.946232
O 0.385395 -0.440519 -0.336717
C 1.809096 1.455285 -0.128823
C 0.621723 2.438702 -0.155371
O 0.147718 2.672695 -1.449545
C 3.298822 -1.362386 0.121473
O 2.338946 -2.046852 0.915314
O 1.849065 3.816517 1.382171
H 3.458843 -0.213116 -1.681056
H 1.210087 0.563028 -1.943197
H -0.172677 2.016897 0.480263
H 0.56541 4.663711 0.065442
H 2.871597 -2.248471 -2.421049
H -0.745241 2.297298 -1.509193
H 2.297939 1.915204 1.69323
H 3.746229 -0.587774 0.746509
H 4.091521 -2.057377 -0.186174
H 1.753619 -2.560153 0.330357
H 2.699158 1.939119 -0.55535
H -0.067444 -1.24894 -2.165991
Na 0.879862 -0.701238 1.955554

Transition state of 3rd c2 reaction of Manβ(1→4)Manβ
46

C -0.700076 -1.173479 -1.129961
O -1.610372 -0.29377 -1.722863
C -2.875351 -0.123146 -1.070106
C -2.691213 -0.188384 0.448826
O -3.892273 0.053857 1.145324
C -2.185938 -1.566402 0.879443
C -1.38404 -2.231577 -0.258884
O -0.419041 -3.091421 0.31205
O -1.360715 -1.451177 2.033444
C -3.438193 1.202921 -1.534504
O -2.670987 2.24572 -0.933698
O 1.950072 0.525652 1.481999
H -3.571427 -0.910437 -1.397291
H -1.924524 0.541792 0.724543
H -3.052351 -2.196453 1.112939
H -2.086542 -2.790599 -0.888047
H -0.1576 -3.776714 -0.314108
H -1.923582 -1.253041 2.792179
H -4.023752 1.004822 1.244033
H -4.48776 1.271161 -1.228446
H -3.376751 1.251433 -2.626781
H -3.057194 3.101952 -1.15574
C 1.150033 3.16344 1.075413
O 2.651568 -1.823973 0.001516
C 2.376703 -1.042828 -1.149134
C 1.364169 0.079572 -0.812449
O 0.18804 -0.507696 -0.264371
C 1.930187 0.979386 0.278266
C 0.393976 2.329766 0.270928
O 0.09953 2.72117 -1.003971
C 3.68081 -0.545112 -1.769888
O 4.453241 -0.014326 -0.706082
O 1.539483 2.816981 2.270807
H 1.899865 -1.708345 -1.877575
H 1.12706 0.665712 -1.709151
H -0.243189 1.577707 0.725063
H 1.550338 4.098131 0.677799
H 3.447445 -1.438429 0.407408
H -0.814586 2.447552 -1.204133
H 1.683795 1.790068 2.258598
H 3.472769 0.212357 -2.536351
H 4.192713 -1.393517 -2.236761
H 5.3733 0.092993 -0.971575
H 2.773569 1.588739 -0.069649
H -0.151297 -1.641545 -1.955556
Na 0.926811 -1.571674 1.631604

Reactant state of 3rd c2 reaction of Manβ(1→4)Manβ
46

C -0.831388 -1.194681 -1.127816
O -1.680576 -0.260897 -1.728277
C -2.898466 0.04838 -1.037974
C -2.674649 -0.024023 0.475191
O -3.818542 0.354634 1.206255
C -2.312515 -1.445545 0.904067
C -1.586226 -2.187431 -0.239204
O -0.684564 -3.119014 0.324789
O -1.474925 -1.413433 2.055169
C -3.336883 1.424325 -1.492615
O -2.483293 2.390245 -0.878473
O 1.983376 0.378829 1.39686
H -3.682045 -0.664801 -1.335995
H -1.821374 0.616856 0.7178
H -3.237167 -1.984071 1.143553
H -2.341097 -2.692311 -0.852954
H -0.511164 -3.841946 -0.289235
H -2.008945 -1.134288 2.809407
H -3.856096 1.317298 1.267311
H -4.377325 1.58563 -1.190029
H -3.265818 1.475532 -2.584086
H -2.758825 3.27677 -1.142679
C 1.342388 3.265751 1.097422
O 2.525502 -1.969972 0.059424
C 2.288644 -1.20734 -1.115848
C 1.303156 -0.053766 -0.855599
O 0.105871 -0.581351 -0.276162
C 1.785038 1.002569 0.128432
C 0.74726 2.132395 0.272799
O 0.341919 2.627646 -0.972817
C 3.620775 -0.753114 -1.711896
O 4.374319 -0.206295 -0.640661
O 2.124005 3.072184 2.001585
H 1.819068 -1.88744 -1.836105
H 1.069159 0.449062 -1.802644
H -0.100184 1.731406 0.849726
H 1.01811 4.284705 0.815783
H 3.287489 -1.54928 0.494006
H -0.595279 2.411403 -1.115928
H 2.31507 1.06961 1.995465
H 3.451225 -0.011933 -2.503672
H 4.130173 -1.622794 -2.14024
H 5.304121 -0.134286 -0.882637
H 2.731668 1.417144 -0.237961
H -0.319613 -1.710302 -1.94963
Na 0.7783 -1.693428 1.600676

Transition state of 1st m1_2 reaction of Manβ(1→4)Manβ
46

C 0.787976 -0.924028 0.450472
O 1.096631 0.450918 0.649468
C 2.391652 0.800308 1.130657
C 3.513637 0.082731 0.366845
O 4.76605 0.281779 0.987426
C 3.219869 -1.399574 0.167952
C 1.836641 -1.569422 -0.44572
O 1.780825 -0.897778 -1.696407
O 4.126434 -1.97252 -0.743744
C 2.479995 2.304394 0.928819
O 2.196538 2.551326 -0.451075
O -2.970804 0.565356 1.960181
H 2.466069 0.567349 2.205794
H 3.607672 0.54108 -0.621868
H 3.251437 -1.923803 1.139662
H 1.601987 -2.633776 -0.569499
H 2.531529 -1.209502 -2.224006
H 5.026472 -1.70303 -0.512427
H 4.767914 -0.095325 1.878007
H 3.488481 2.640368 1.190135
H 1.742999 2.810441 1.561227
H 2.514124 3.428348 -0.69294
C -3.22969 2.591691 0.091789
O -3.97219 -1.324813 0.229959
C -2.703217 -1.469993 -0.394861
C -1.625566 -0.875405 0.521426
O -0.416041 -0.947778 -0.232518
C -1.964085 0.560196 0.980278
C -2.343924 1.488813 -0.18361
O -1.563586 1.670968 -1.214504
C -2.452424 -2.934053 -0.717136
O -2.620078 -3.62638 0.51276
O -3.241854 3.524493 -0.795742
H -2.71834 -0.894458 -1.328915
H -1.536665 -1.494031 1.42658
H -3.492157 1.123977 -0.46941
H -3.933157 2.645713 0.916747
H -4.148313 -2.151127 0.709074
H -2.498003 3.2779 -1.415494
H -3.669557 -0.042558 1.667193
H -1.443827 -3.063229 -1.123671
H -3.189552 -3.263578 -1.458555
H -2.680997 -4.575883 0.362744
H -1.067824 0.992675 1.434876
H 0.721135 -1.442164 1.421123
Na 0.521637 1.120554 -1.502207

Reactant state of 1st m1_2 reaction of Manβ(1→4)Manβ
46

C -0.71901 0.929887 0.443515
O -1.053519 -0.439593 0.634865
C -2.34671 -0.787012 1.123644
C -3.468563 -0.025689 0.403926
O -4.712299 -0.219465 1.041738
C -3.149771 1.456817 0.243365
C -1.780017 1.62128 -0.402208
O -1.773849 0.990052 -1.676055
O -4.066528 2.073745 -0.628183
C -2.454253 -2.281999 0.867259
O -2.177619 -2.482028 -0.523756
O 3.010232 -0.650545 1.856555
H -2.400576 -0.593485 2.207221
H -3.591509 -0.451547 -0.596333
H -3.146624 1.951303 1.230868
H -1.528885 2.684397 -0.500503
H -2.538437 1.326442 -2.167871
H -4.966411 1.817133 -0.381632
H -4.68967 0.124062 1.945587
H -3.465978 -2.616125 1.117695
H -1.72124 -2.820811 1.476267
H -2.493755 -3.352781 -0.790245
C 2.949664 -2.882986 0.14802
O 4.026352 1.303272 0.158557
C 2.748868 1.494725 -0.429539
C 1.689795 0.819727 0.451213
O 0.464117 0.919627 -0.281829
C 2.049545 -0.635423 0.828044
C 2.574319 -1.489692 -0.339851
O 1.619439 -1.633166 -1.377804
C 2.482186 2.979088 -0.62231
O 2.645066 3.564633 0.661805
O 2.536022 -3.850357 -0.449774
H 2.754137 1.006287 -1.411755
H 1.598464 1.376985 1.39428
H 3.497516 -1.027795 -0.727917
H 3.598684 -2.962832 1.031691
H 4.203294 2.080557 0.712876
H 1.716226 -2.561042 -1.669902
H 3.7553 -0.100145 1.566893
H 1.470971 3.133294 -1.014784
H 3.213154 3.378841 -1.334616
H 2.716581 4.522701 0.592464
H 1.148787 -1.11157 1.229161
H -0.598553 1.433252 1.415587
Na -0.557011 -1.003534 -1.540405

Transition state of 2nd m1_2 reaction of Manβ(1→4)Manβ
46

C 0.788012 -0.923931 0.450642
O 1.096658 0.451065 0.649345
C 2.391685 0.800614 1.130423
C 3.513682 0.082844 0.366809
O 4.766082 0.28204 0.987371
C 3.21992 -1.399506 0.168283
C 1.836704 -1.569551 -0.445354
O 1.780852 -0.898286 -1.696241
O 4.126505 -1.972689 -0.743245
C 2.480043 2.30465 0.928211
O 2.196666 2.55127 -0.451747
O -2.970687 0.565258 1.960398
H 2.466102 0.567971 2.205628
H 3.607741 0.540961 -0.62201
H 3.251469 -1.923475 1.140135
H 1.602089 -2.633951 -0.568814
H 2.531632 -1.210043 -2.223714
H 5.026537 -1.703142 -0.511978
H 4.767953 -0.094929 1.87801
H 3.488527 2.640662 1.18949
H 1.743033 2.810858 1.560474
H 2.514352 3.428198 -0.69382
C -3.229748 2.591595 0.092004
O -3.972188 -1.324557 0.22976
C -2.703168 -1.469841 -0.394944
C -1.62554 -0.875443 0.521489
O -0.415979 -0.947828 -0.232402
C -1.964021 0.560137 0.980442
C -2.343942 1.488763 -0.183406
O -1.563704 1.67089 -1.214373
C -2.452521 -2.9339 -0.717313
O -2.620146 -3.626278 0.512559
O -3.242038 3.524362 -0.795568
H -2.718117 -0.894216 -1.328946
H -1.536729 -1.494163 1.426587
H -3.492213 1.123893 -0.469084
H -3.933146 2.645616 0.91702
H -4.14841 -2.150852 0.708871
H -2.498217 3.277792 -1.415369
H -3.669517 -0.042531 1.66734
H -1.443971 -3.063147 -1.123942
H -3.189746 -3.263315 -1.458685
H -2.6813 -4.575756 0.362482
H -1.067742 0.992605 1.435016
H 0.721105 -1.441835 1.421411
Na 0.521434 1.120203 -1.502425

Reactant state of 2nd m1_2 reaction of Manβ(1→4)Manβ
46

C -0.719263 0.929904 0.443231
O -1.053598 -0.439511 0.63533
C -2.346825 -0.7869 1.124039
C -3.468695 -0.025984 0.40392
O -4.712464 -0.219613 1.041721
C -3.15004 1.456471 0.242707
C -1.780304 1.620672 -0.402943
O -1.774068 0.988597 -1.676357
O -4.066836 2.072936 -0.62912
C -2.454115 -2.281965 0.868061
O -2.177352 -2.482272 -0.522872
O 3.010358 -0.649958 1.856502
H -2.400872 -0.593029 2.207549
H -3.59153 -0.452291 -0.596164
H -3.146916 1.95142 1.229978
H -1.529224 2.683748 -0.501864
H -2.538747 1.324509 -2.16836
H -4.966687 1.816233 -0.382542
H -4.689897 0.124191 1.945466
H -3.465797 -2.616193 1.11853
H -1.721053 -2.820489 1.477268
H -2.493614 -3.352994 -0.789307
C 2.951016 -2.882338 0.147818
O 4.025955 1.304233 0.158672
C 2.748445 1.49532 -0.429488
C 1.689522 0.819855 0.451093
O 0.463896 0.919358 -0.282091
C 2.049753 -0.635198 0.827888
C 2.574907 -1.489235 -0.339988
O 1.620079 -1.633188 -1.37795
C 2.48125 2.979626 -0.622066
O 2.64381 3.565054 0.662144
O 2.537858 -3.849903 -0.449977
H 2.753954 1.007026 -1.411769
H 1.597896 1.376988 1.394204
H 3.497865 -1.026883 -0.728058
H 3.600176 -2.96188 1.031419
H 4.202622 2.081528 0.713063
H 1.717404 -2.560998 -1.670054
H 3.755281 -0.099341 1.566884
H 1.470023 3.13354 -1.014618
H 3.212146 3.379734 -1.334248
H 2.714764 4.523176 0.592962
H 1.149142 -1.111675 1.228939
H -0.598859 1.433849 1.415005
Na -0.556642 -1.00431 -1.539811

Transition state of 3rd m1_2 reaction of Manβ(1→4)Manβ
46

C 0.788007 -0.924094 0.450528
O 1.09663 0.450901 0.649285
C 2.391641 0.800393 1.130472
C 3.513669 0.082624 0.366887
O 4.766028 0.281709 0.987565
C 3.21988 -1.399708 0.168206
C 1.836694 -1.569654 -0.445523
O 1.780935 -0.898291 -1.696366
O 4.126489 -1.972783 -0.743368
C 2.480098 2.304438 0.928346
O 2.196765 2.55117 -0.451598
O -2.970576 0.565422 1.960324
H 2.465958 0.567678 2.205668
H 3.607822 0.540807 -0.621892
H 3.251403 -1.92378 1.139999
H 1.60203 -2.63403 -0.569098
H 2.531737 -1.210036 -2.223814
H 5.026559 -1.703633 -0.511791
H 4.76771 -0.095049 1.878293
H 3.488602 2.640369 1.189652
H 1.743112 2.810662 1.560624
H 2.514347 3.428168 -0.693555
C -3.229361 2.591791 0.092004
O -3.972339 -1.323894 0.229392
C -2.703247 -1.469682 -0.39504
C -1.625572 -0.875443 0.521451
O -0.416013 -0.948088 -0.232425
C -1.963869 0.560181 0.980406
C -2.34366 1.488874 -0.183438
O -1.563406 1.670933 -1.214414
C -2.452985 -2.933889 -0.717073
O -2.621347 -3.626022 0.512836
O -3.241543 3.524602 -0.795535
H -2.717823 -0.894235 -1.329157
H -1.536875 -1.494182 1.42655
H -3.491935 1.124094 -0.469159
H -3.932683 2.645913 0.917078
H -4.149005 -2.15014 0.708426
H -2.497818 3.277962 -1.41535
H -3.669453 -0.042294 1.66724
H -1.444305 -3.063539 -1.123254
H -3.189998 -3.263183 -1.458708
H -2.683034 -4.57548 0.362852
H -1.067562 0.992513 1.435047
H 0.721297 -1.442028 1.421302
Na 0.521741 1.12029 -1.502354

Reactant state of 3rd m1_2 reaction of Manβ(1→4)Manβ
46

C -0.719279 0.929911 0.44322
O -1.053612 -0.439501 0.635349
C -2.34684 -0.786873 1.124065
C -3.468707 -0.025991 0.403906
O -4.71248 -0.219601 1.041705
C -3.150057 1.45646 0.242638
C -1.780308 1.620646 -0.402992
O -1.774039 0.988527 -1.676385
O -4.066838 2.072886 -0.629231
C -2.454129 -2.281952 0.868163
O -2.177372 -2.482334 -0.522759
O 3.01038 -0.649953 1.856485
H -2.400892 -0.592948 2.207566
H -3.591532 -0.452339 -0.596162
H -3.146955 1.951447 1.22989
H -1.529232 2.683719 -0.501947
H -2.538698 1.32443 -2.168425
H -4.966694 1.816198 -0.382654
H -4.689923 0.124242 1.945436
H -3.465811 -2.616168 1.118653
H -1.721067 -2.820446 1.477397
H -2.493611 -3.353081 -0.789139
C 2.951102 -2.882298 0.147774
O 4.025935 1.304296 0.158705
C 2.748427 1.495353 -0.42947
C 1.689514 0.819859 0.451099
O 0.463888 0.919356 -0.282088
C 2.049771 -0.635195 0.827875
C 2.574945 -1.489202 -0.340014
O 1.620113 -1.633164 -1.37797
C 2.481197 2.979652 -0.622045
O 2.643723 3.565074 0.662172
O 2.537982 -3.849868 -0.450042
H 2.75396 1.007063 -1.411753
H 1.597878 1.376981 1.394215
H 3.497887 -1.026815 -0.728081
H 3.600257 -2.961827 1.03138
H 4.20257 2.081586 0.713112
H 1.717487 -2.560952 -1.670135
H 3.755287 -0.099308 1.56688
H 1.469972 3.133544 -1.014612
H 3.212096 3.379782 -1.334212
H 2.714666 4.523198 0.592996
H 1.149172 -1.111693 1.228922
H -0.598899 1.433882 1.414982
Na -0.556652 -1.004404 -1.539742

Transition state of 1st m1_5 reaction of Manβ(1→4)Manβ
46

C 0.891023 -0.865213 0.079841
O 1.214501 0.524496 -0.06173
C 2.43065 0.863448 0.611229
C 3.598045 0.063932 0.032541
O 4.735944 0.406944 0.785515
C 3.308445 -1.429315 0.097611
C 1.962925 -1.729523 -0.568452
O 1.999298 -1.405735 -1.934591
O 4.36256 -2.063647 -0.605749
C 2.679306 2.345303 0.417463
O 1.525844 3.07514 0.855058
O -1.136302 1.098403 -1.920091
H 2.325652 0.644596 1.685764
H 3.720129 0.338045 -1.025731
H 3.293471 -1.739099 1.152689
H 1.685157 -2.78323 -0.413676
H 2.777768 -1.830868 -2.321323
H 4.51564 -2.954348 -0.269136
H 5.488989 -0.094988 0.445722
H 2.860719 2.552828 -0.644567
H 3.566142 2.624083 0.990174
H 1.794692 3.903793 1.265623
C -4.19995 0.308064 0.066063
O -3.115669 -2.16727 -0.166028
C -2.451009 -1.355033 0.592066
C -1.430355 -0.42277 -0.063718
O -0.306985 -1.119369 -0.569349
C -2.088202 0.290364 -1.248133
C -3.212914 1.175828 -0.728419
O -2.607525 2.173084 0.096992
C -2.118864 -1.877326 1.978179
O -1.434581 -0.863355 2.68929
O -4.812489 -0.623705 -0.578035
H -3.314127 -0.335037 0.872037
H -1.116007 0.295964 0.700805
H -3.744935 1.638473 -1.568214
H -4.746189 0.821871 0.871818
H -4.057564 -1.573603 -0.551121
H -3.264649 2.828818 0.360719
H -0.465544 0.504957 -2.295455
H -1.48874 -2.766814 1.835773
H -3.047736 -2.187711 2.467377
H -1.396743 -1.089991 3.625029
H -2.507021 -0.456689 -1.931701
H 0.80939 -1.081354 1.159666
Na -0.299987 2.386714 -0.249105

Reactant state of 1st m1_5 reaction of Manβ(1→4)Manβ
46

C 0.856427 -0.81231 0.083699
O 1.162562 0.590562 0.017528
C 2.407252 0.897919 0.649365
C 3.548649 0.159056 -0.049132
O 4.723659 0.460659 0.663742
C 3.277154 -1.338481 -0.085002
C 1.897608 -1.60608 -0.691558
O 1.846032 -1.17511 -2.028965
O 4.295435 -1.900979 -0.895367
C 2.630338 2.394246 0.548565
O 1.469343 3.07348 1.043704
O -1.210053 1.065531 -1.779127
H 2.360855 0.600264 1.708651
H 3.609332 0.513833 -1.088692
H 3.326283 -1.72991 0.94144
H 1.641879 -2.673221 -0.605984
H 2.600845 -1.559015 -2.496775
H 4.473553 -2.813809 -0.640413
H 5.461355 -0.002639 0.244793
H 2.795593 2.672716 -0.500035
H 3.519864 2.6521 1.126885
H 1.726877 3.887176 1.489726
C -4.491591 0.690999 -0.049944
O -2.955358 -2.391215 -0.246657
C -2.405544 -1.531459 0.717723
C -1.516406 -0.44907 0.084672
O -0.368496 -1.038859 -0.518003
C -2.191396 0.310749 -1.0759
C -3.24616 1.327015 -0.652956
O -2.636924 2.245071 0.255142
C -1.601883 -2.402213 1.675272
O -0.929053 -1.541834 2.586667
O -4.982679 -0.316738 -0.492938
H -3.183366 -1.026283 1.308964
H -1.209864 0.238157 0.884476
H -3.563739 1.853145 -1.565384
H -4.943793 1.226264 0.81008
H -3.835719 -2.08564 -0.504911
H -3.225925 2.990376 0.428132
H -0.535578 0.438341 -2.091695
H -0.890638 -3.003033 1.094196
H -2.292442 -3.079134 2.186308
H -0.783411 -1.998094 3.421812
H -2.66143 -0.417265 -1.746112
H 0.839062 -1.100967 1.146643
Na -0.321093 2.364533 -0.120611

Transition state of 2nd m1_5 reaction of Manβ(1→4)Manβ
46

C 1.01098 -0.292879 1.145992
O 1.113625 0.626839 0.064659
C 2.472379 1.006456 -0.219123
C 3.313583 -0.220597 -0.563093
O 4.621338 0.235515 -0.809756
C 3.283171 -1.207515 0.596696
C 1.83722 -1.561472 0.914785
O 1.351444 -2.29233 -0.184697
O 4.051245 -2.344009 0.29176
C 2.466796 1.969122 -1.385038
O 1.716726 3.138727 -1.029684
O -1.387446 -2.91566 -0.159698
H 2.898499 1.506687 0.665299
H 2.883862 -0.706045 -1.453394
H 3.754928 -0.733449 1.46746
H 1.793333 -2.155398 1.838007
H 0.497167 -2.698477 0.041483
H 3.505376 -2.931447 -0.252747
H 5.209398 -0.533657 -0.810378
H 2.011467 1.488611 -2.259671
H 3.504358 2.224125 -1.611422
H 2.062282 3.906006 -1.498542
C -4.150586 -0.351793 -0.272489
O -2.257593 1.572774 -0.417408
C -2.152066 0.714653 0.568289
C -1.275845 -0.529239 0.307846
O -0.333564 -0.661731 1.352935
C -2.139213 -1.805892 0.263146
C -3.326063 -1.581603 -0.683755
O -2.744249 -1.412213 -1.951216
C -2.09278 1.338516 1.962865
O -0.924917 2.160346 1.963219
O -4.279273 0.616088 -1.124852
H -3.323335 0.154077 0.679545
H -0.792018 -0.405347 -0.66455
H -3.979791 -2.465152 -0.653635
H -4.998518 -0.526974 0.400352
H -3.318737 1.196655 -1.062056
H -3.408418 -1.145385 -2.600808
H -1.387014 -2.920381 -1.130958
H -2.020672 0.56151 2.728911
H -2.988026 1.951089 2.111089
H -0.802959 2.556699 2.834051
H -2.500083 -2.026231 1.276384
H 1.336437 0.207804 2.070526
Na -0.258334 2.621216 -0.172162

Reactant state of 2nd m1_5 reaction of Manβ(1→4)Manβ
46

C 1.016783 -0.325597 1.041732
O 1.081908 0.501844 -0.10926
C 2.403523 1.009147 -0.347427
C 3.385941 -0.140969 -0.554837
O 4.653957 0.43479 -0.755414
C 3.380102 -1.054447 0.666429
C 1.958373 -1.52673 0.940646
O 1.604986 -2.366184 -0.129345
O 4.266911 -2.127964 0.473108
C 2.352449 1.888493 -1.575566
O 1.442951 2.972487 -1.335322
O -1.183499 -2.621124 -0.657375
H 2.721457 1.610375 0.520336
H 3.066959 -0.723946 -1.432578
H 3.759135 -0.484933 1.525303
H 1.91989 -2.062893 1.899758
H 0.683773 -2.660313 -0.027152
H 3.80695 -2.8034 -0.048053
H 5.314933 -0.268878 -0.682977
H 2.009847 1.30197 -2.436236
H 3.36151 2.261095 -1.765868
H 1.683217 3.725736 -1.885987
C -4.498876 -0.886893 -0.335431
O -2.563715 1.654181 0.151109
C -2.277475 0.599604 1.065635
C -1.353761 -0.446333 0.379947
O -0.285243 -0.845423 1.21091
C -2.086292 -1.742423 -0.022751
C -3.264674 -1.487108 -0.982571
O -2.757376 -0.674807 -2.013181
C -1.696547 1.23112 2.318615
O -0.555785 2.008675 1.945535
O -4.911237 0.212421 -0.626766
H -3.211866 0.126354 1.397752
H -0.973238 -0.01083 -0.546572
H -3.565805 -2.480331 -1.354024
H -5.026104 -1.522089 0.401601
H -3.303521 1.403523 -0.428457
H -3.42742 -0.534503 -2.694931
H -1.006278 -2.287681 -1.550492
H -1.410706 0.447331 3.026173
H -2.461047 1.878063 2.759564
H -0.240949 2.507273 2.708121
H -2.437387 -2.260098 0.878041
H 1.264982 0.28489 1.923763
Na -0.436043 2.423176 -0.271418

Transition state of 3rd m1_5 reaction of Manβ(1→4)Manβ
46

C 1.010921 -0.292849 1.145881
O 1.11374 0.626725 0.064425
C 2.472617 1.006261 -0.218916
C 3.313565 -0.220901 -0.563107
O 4.62141 0.234904 -0.80981
C 3.282959 -1.207915 0.596612
C 1.836932 -1.561595 0.914736
O 1.351012 -2.292428 -0.184697
O 4.050772 -2.344555 0.29158
C 2.467558 1.969407 -1.38443
O 1.717449 3.138959 -1.028989
O -1.387854 -2.915614 -0.158946
H 2.898573 1.506043 0.665839
H 2.88364 -0.706152 -1.453419
H 3.754841 -0.734017 1.4674
H 1.792959 -2.155462 1.83799
H 0.496758 -2.698548 0.041609
H 3.50475 -2.931842 -0.252939
H 5.209305 -0.534395 -0.810386
H 2.012537 1.489237 -2.259366
H 3.505219 2.224401 -1.610364
H 2.06302 3.90631 -1.497722
C -4.150719 -0.351426 -0.272708
O -2.257534 1.573019 -0.417596
C -2.152124 0.714969 0.56817
C -1.276026 -0.529059 0.307901
O -0.333676 -0.66135 1.352973
C -2.139503 -1.805632 0.263551
C -3.326268 -1.581492 -0.68348
O -2.744286 -1.412614 -1.950938
C -2.092718 1.338978 1.962661
O -0.924875 2.160846 1.962736
O -4.279082 0.616294 -1.125299
H -3.323487 0.15443 0.6794
H -0.792271 -0.405401 -0.664568
H -3.980099 -2.464954 -0.653091
H -4.998878 -0.526339 0.39991
H -3.318455 1.196541 -1.062636
H -3.408507 -1.146895 -2.600926
H -1.387552 -2.9207 -1.130205
H -2.020469 0.562098 2.728821
H -2.987977 1.951526 2.110886
H -0.804208 2.559265 2.832808
H -2.500451 -2.025644 1.276831
H 1.336538 0.207875 2.070333
Na -0.257778 2.620605 -0.172812

Reactant state of 3rd m1_5 reaction of Manβ(1→4)Manβ
46

C 1.016783 -0.325596 1.041731
O 1.081909 0.501844 -0.109261
C 2.403525 1.009145 -0.347426
C 3.385941 -0.140972 -0.554836
O 4.653958 0.434785 -0.755413
C 3.380101 -1.05445 0.666429
C 1.958371 -1.526731 0.940646
O 1.604983 -2.366185 -0.129344
O 4.266907 -2.127969 0.473108
C 2.352455 1.888494 -1.575564
O 1.442958 2.972489 -1.335322
O -1.183503 -2.621123 -0.657375
H 2.721459 1.610371 0.520338
H 3.066958 -0.723949 -1.432578
H 3.759135 -0.484937 1.525303
H 1.919888 -2.062894 1.899758
H 0.68377 -2.660314 -0.02715
H 3.806946 -2.803404 -0.048052
H 5.314933 -0.268884 -0.682977
H 2.009856 1.301973 -2.436237
H 3.361517 2.261094 -1.765861
H 1.683223 3.725735 -1.885991
C -4.498879 -0.886891 -0.33543
O -2.563712 1.654185 0.151105
C -2.277475 0.599607 1.065633
C -1.353763 -0.446331 0.379946
O -0.285244 -0.845422 1.21091
C -2.086295 -1.742421 -0.02275
C -3.264678 -1.487108 -0.982568
O -2.75738 -0.674809 -2.013181
C -1.696548 1.231124 2.318613
O -0.555785 2.008677 1.945533
O -4.911239 0.212423 -0.626766
H -3.211868 0.126359 1.397749
H -0.973239 -0.01083 -0.546573
H -3.56581 -2.480331 -1.35402
H -5.026109 -1.522084 0.401604
H -3.303519 1.403528 -0.428461
H -3.427423 -0.53451 -2.694933
H -1.006279 -2.287677 -1.55049
H -1.41071 0.447336 3.026172
H -2.461049 1.878069 2.75956
H -0.240949 2.507278 2.708118
H -2.437387 -2.260095 0.878044
H 1.264982 0.284891 1.923763
Na -0.436033 2.423173 -0.271417

Transition state of 1st m1_6 reaction of Manβ(1→4)Manβ
46

C 1.284187 -0.566705 1.522526
O 1.582781 0.691532 0.960162
C 2.928355 0.917797 0.549026
C 3.502988 -0.259495 -0.268979
O 4.417641 -0.931658 0.576666
C 2.392621 -1.226856 -0.689126
C 1.682965 -1.740146 0.563302
O 0.57937 -2.485444 0.096225
O 1.449327 -0.632634 -1.574863
C 2.886969 2.20037 -0.26652
O 2.133985 2.006768 -1.457841
O -1.997352 -2.016685 0.192099
H 3.572249 1.050593 1.429743
H 4.006753 0.130196 -1.163742
H 2.839212 -2.086846 -1.204014
H 2.373809 -2.393121 1.106952
H -0.057716 -2.639208 0.808876
H 1.679673 0.314661 -1.702985
H 5.009978 -1.490754 0.059747
H 3.903812 2.482917 -0.553058
H 2.460309 3.008594 0.335984
H 1.224316 2.316075 -1.325136
C -3.035957 0.711436 -1.098658
O -2.250865 1.749556 2.046545
C -1.16769 1.517357 1.17221
C -0.939168 0.02249 0.870034
O -0.087574 -0.568543 1.850508
C -2.251335 -0.776441 0.847061
C -3.441725 -0.092665 0.137037
O -4.318612 -1.134243 -0.262992
C -1.446879 2.304175 -0.106714
O -0.682507 2.097641 -1.140146
O -2.032111 0.31965 -1.847476
H -0.229135 1.916671 1.57243
H -0.431731 -0.03169 -0.093282
H -3.932993 0.583668 0.841838
H -3.883066 1.172114 -1.628486
H -1.949512 1.746824 2.962489
H -5.237517 -0.898268 -0.093975
H -2.852436 -2.468584 0.116108
H -1.837017 3.318604 0.043093
H -2.634503 1.787523 -0.470811
H -1.184758 1.187637 -1.694594
H -2.54306 -0.969625 1.886634
H 1.809598 -0.683297 2.478154
Na -0.620992 -1.653647 -1.691841

Reactant state of 1st m1_6 reaction of Manβ(1→4)Manβ
46

C 1.247919 -0.481499 1.520951
O 1.601502 0.727142 0.895756
C 2.968004 0.894176 0.527894
C 3.535195 -0.335077 -0.217486
O 4.430281 -0.974635 0.672797
C 2.412748 -1.303435 -0.601689
C 1.671 -1.722921 0.665665
O 0.56931 -2.502042 0.244415
O 1.506095 -0.739266 -1.5441
C 2.980601 2.13576 -0.349749
O 2.200802 1.910737 -1.516346
O -2.04577 -1.946244 0.277317
H 3.584384 1.049184 1.424429
H 4.053932 -0.002781 -1.126726
H 2.849625 -2.201204 -1.056974
H 2.348475 -2.33415 1.271082
H -0.102639 -2.532244 0.941971
H 1.732817 0.209328 -1.683072
H 5.027995 -1.558936 0.191366
H 4.005418 2.358508 -0.659667
H 2.594361 2.991124 0.213694
H 1.299775 2.246627 -1.361544
C -3.24659 0.103139 -1.447586
O -2.230244 1.866663 1.975149
C -1.153822 1.649359 1.075002
C -0.961691 0.150804 0.765802
O -0.13707 -0.447293 1.77531
C -2.276803 -0.636473 0.802194
C -3.47397 -0.050029 0.049059
O -4.543907 -0.978967 0.143046
C -1.449553 2.525067 -0.151414
O -0.534174 2.301203 -1.211535
O -2.173235 -0.064081 -1.995961
H -0.209496 1.98931 1.506546
H -0.459217 0.074038 -0.201602
H -3.747134 0.924019 0.471272
H -4.145426 0.352218 -2.038166
H -1.90753 1.888197 2.882843
H -5.242411 -0.651446 0.721418
H -2.86914 -2.446181 0.38491
H -1.365074 3.570656 0.154227
H -2.481507 2.388028 -0.494562
H -0.937244 1.747328 -1.891878
H -2.569099 -0.712941 1.856406
H 1.719632 -0.544893 2.509769
Na -0.531586 -1.782068 -1.597671

Transition state of 2nd m1_6 reaction of Manβ(1→4)Manβ
46

C 0.963587 -0.550579 1.392983
O 1.450626 0.69393 0.881204
C 2.882934 0.707406 0.788773
C 3.358921 -0.38679 -0.171803
O 4.760302 -0.399551 -0.107112
C 2.764308 -1.747629 0.195428
C 1.247531 -1.637609 0.347144
O 0.725784 -1.295127 -0.942539
O 3.137768 -2.709211 -0.758885
C 3.301026 2.090828 0.33772
O 2.673256 2.347963 -0.925242
O -1.765119 -2.128748 -0.021348
H 3.31868 0.520145 1.781323
H 3.029355 -0.136605 -1.194811
H 3.202123 -2.072073 1.147788
H 0.824626 -2.590382 0.681098
H -0.155639 -1.708129 -1.015839
H 2.569434 -2.602386 -1.535523
H 5.083116 -1.181437 -0.577378
H 4.390037 2.104461 0.238325
H 2.978365 2.835589 1.07193
H 3.09825 3.098152 -1.35541
C -3.575783 0.280835 -1.155718
O -2.895547 1.488334 1.960194
C -1.780869 1.428846 1.100865
C -1.298487 0.008726 0.807429
O -0.35635 -0.40337 1.79272
C -2.375292 -1.072752 0.70756
C -3.704638 -0.655992 0.045515
O -4.306869 -1.856001 -0.409015
C -2.189127 2.106485 -0.208569
O -1.387033 1.962556 -1.238495
O -2.61516 0.124868 -2.012482
H -0.925545 1.989126 1.504623
H -0.819576 0.033135 -0.16417
H -4.32188 -0.156299 0.799016
H -4.535991 0.65125 -1.546164
H -2.612652 1.454844 2.881813
H -5.25111 -1.871273 -0.217359
H -2.455652 -2.763018 -0.267361
H -2.685339 3.077727 -0.091784
H -3.25951 1.431719 -0.479116
H -1.916078 1.010105 -1.870901
H -2.612398 -1.417398 1.722627
H 1.507913 -0.794487 2.314886
Na 0.782867 1.186494 -1.33669

Reactant state of 2nd m1_6 reaction of Manβ(1→4)Manβ
46

C -0.89824 0.532974 1.350604
O -1.48857 -0.675014 0.857659
C -2.920856 -0.595783 0.817603
C -3.352472 0.51751 -0.140278
O -4.749265 0.612946 -0.052201
C -2.673782 1.840005 0.216983
C -1.162289 1.649198 0.334793
O -0.678486 1.303462 -0.968777
O -3.014034 2.828285 -0.723052
C -3.446326 -1.953012 0.400805
O -2.8802 -2.265916 -0.878414
O 1.855647 2.093948 0.139745
H -3.306365 -0.369247 1.822787
H -3.05292 0.243729 -1.166742
H -3.070726 2.182689 1.180948
H -0.687209 2.573256 0.677291
H 0.197557 1.713372 -1.065248
H -2.481001 2.685213 -1.518553
H -5.032676 1.41659 -0.51101
H -4.536489 -1.89418 0.337723
H -3.149276 -2.708894 1.134508
H -3.367442 -2.991474 -1.284039
C 3.500736 0.592537 -1.604928
O 2.875755 -1.626049 1.885728
C 1.760902 -1.573664 1.0155
C 1.347841 -0.135931 0.680291
O 0.422881 0.301795 1.684736
C 2.453073 0.916349 0.678367
C 3.7434 0.63134 -0.10319
O 4.626903 1.716657 0.102874
C 2.136388 -2.417986 -0.207152
O 1.192136 -2.263762 -1.269617
O 2.658039 -0.10419 -2.126545
H 0.877415 -2.026722 1.483971
H 0.883102 -0.127804 -0.301544
H 4.179164 -0.324153 0.217947
H 4.152279 1.25429 -2.202736
H 2.585197 -1.605309 2.804651
H 5.232623 1.536296 0.830617
H 2.478816 2.827918 0.239249
H 2.160394 -3.468993 0.088444
H 3.137596 -2.149195 -0.556818
H 1.650354 -1.755074 -1.955028
H 2.743055 1.083939 1.723628
H -1.383109 0.7982 2.300011
Na -0.88246 -1.299361 -1.284867

Transition state of 3rd m1_6 reaction of Manβ(1→4)Manβ
46

C 0.963352 -0.550518 1.393003
O 1.450501 0.693932 0.881136
C 2.882819 0.707269 0.788812
C 3.358757 -0.386995 -0.171708
O 4.76013 -0.399912 -0.106905
C 2.763985 -1.747764 0.19553
C 1.247209 -1.637613 0.347195
O 0.72557 -1.295174 -0.942543
O 3.137394 -2.709392 -0.758757
C 3.301184 2.090619 0.337812
O 2.673755 2.347837 -0.925313
O -1.765289 -2.128713 -0.020851
H 3.318463 0.519957 1.781397
H 3.029302 -0.136808 -1.194752
H 3.201739 -2.072228 1.147911
H 0.824214 -2.590333 0.681184
H -0.155874 -1.708094 -1.015878
H 2.568983 -2.602634 -1.535347
H 5.082897 -1.181774 -0.577241
H 4.39022 2.104095 0.238648
H 2.978459 2.835418 1.071958
H 3.097992 3.098958 -1.354608
C -3.575292 0.280841 -1.156046
O -2.895761 1.4888 1.960008
C -1.780973 1.429046 1.100866
C -1.298708 0.00885 0.807566
O -0.35654 -0.403192 1.792855
C -2.37557 -1.072564 0.707741
C -3.704722 -0.655864 0.045244
O -4.306794 -1.855945 -0.409289
C -2.188862 2.10658 -0.208739
O -1.386466 1.962378 -1.238341
O -2.614297 0.124554 -2.012398
H -0.925588 1.989231 1.504634
H -0.819743 0.03317 -0.163981
H -4.322212 -0.156056 0.798466
H -4.535276 0.651306 -1.546986
H -2.613028 1.455107 2.88167
H -5.251181 -1.870986 -0.218351
H -2.455755 -2.76312 -0.266688
H -2.685275 3.077763 -0.092353
H -3.259125 1.431494 -0.479458
H -1.91538 1.009884 -1.870949
H -2.612906 -1.417021 1.722819
H 1.507722 -0.794445 2.314875
Na 0.783151 1.186764 -1.337042

Reactant state of 3rd m1_6 reaction of Manβ(1→4)Manβ
46

C -0.863478 0.336388 1.532638
O -1.2375 -0.804273 0.769488
C -2.654292 -0.907843 0.585283
C -3.208031 0.336273 -0.118173
O -4.607651 0.220801 -0.088891
C -2.732045 1.623087 0.554181
C -1.217116 1.588263 0.726849
O -0.646161 1.533925 -0.580067
O -3.16122 2.740041 -0.186451
C -2.908432 -2.166723 -0.21726
O -2.268173 -2.01376 -1.489494
O 1.737581 2.140164 0.500555
H -3.149623 -1.00788 1.562776
H -2.850667 0.348171 -1.160443
H -3.210443 1.703132 1.538311
H -0.869034 2.481088 1.257061
H 0.267117 1.873035 -0.488642
H -2.565575 2.846134 -0.942144
H -4.989739 1.052248 -0.403126
H -3.988776 -2.284511 -0.341946
H -2.490615 -3.03073 0.309894
H -2.552041 -2.720711 -2.079226
C 2.953667 -0.09833 -1.561222
O 3.389176 -1.631507 1.217424
C 1.976023 -1.53188 1.324678
C 1.434752 -0.148086 0.931662
O 0.473815 0.218087 1.913947
C 2.481941 0.959334 0.771711
C 3.49073 0.714056 -0.386983
O 3.857085 2.013046 -0.854835
C 1.438609 -2.645272 0.417875
O 0.792524 -2.458537 -0.588213
O 1.70899 0.396281 -2.062957
H 1.638802 -1.729013 2.348948
H 0.947675 -0.231819 -0.028797
H 4.358661 0.183768 0.012142
H 3.702607 -0.102402 -2.360953
H 3.774913 -1.952598 2.040072
H 4.805672 2.066334 -1.013554
H 2.371388 2.815924 0.21442
H 1.745942 -3.666397 0.704704
H 2.779912 -1.134466 -1.267178
H 1.864393 1.268715 -2.44941
H 3.03621 1.092744 1.709671
H -1.419511 0.345966 2.480446
Na -0.42445 -0.673318 -1.583139

Transition state of 1st RO1' reaction of Manβ(1→4)Manβ
46

C -1.287534 -0.969322 -1.100017
O -1.506064 0.593547 -1.075246
C -2.845005 1.109126 -0.685255
C -3.79736 -0.047097 -0.421877
O -4.116428 -0.567455 -1.698171
C -3.197706 -1.133597 0.510208
C -1.650282 -1.300851 0.350337
O -0.991816 -0.433901 1.206694
O -3.471293 -0.828456 1.848913
C -2.651442 2.008321 0.56246
O -1.290825 2.428057 0.696088
O 1.001833 -2.543313 0.785223
H -3.169924 1.667795 -1.564344
H -4.68285 0.367337 0.080865
H -3.697692 -2.073842 0.241415
H -1.393135 -2.356531 0.517644
H -0.970159 0.799092 -0.256186
H -2.601883 -0.6365 2.247624
H -4.884687 -1.148961 -1.640856
H -2.890028 1.444536 1.46266
H -3.321912 2.867798 0.490668
H -1.126052 3.24873 0.213934
C 3.902581 -0.355157 -0.223765
O 3.366484 -0.14 -1.496604
C 1.987424 0.197698 -1.639139
C 1.079834 -0.678488 -0.765952
O -0.024172 -1.141564 -1.559132
C 1.83097 -1.80319 -0.062265
C 2.9665 -1.142124 0.726537
O 2.369281 -0.300691 1.720635
C 1.716837 1.68116 -1.376204
O 1.72247 2.014083 0.012763
O 4.244613 0.856709 0.425732
H 1.767758 -0.012691 -2.690054
H 0.67939 -0.070721 0.029807
H 3.539206 -1.924475 1.233564
H 4.80858 -0.936974 -0.413651
H 5.031354 1.228222 0.004714
H 3.079537 -0.012606 2.308958
H 0.529309 -1.922221 1.367578
H 0.717894 1.925456 -1.752236
H 2.447834 2.289465 -1.918921
H 2.636567 1.888317 0.327288
H 2.25893 -2.497833 -0.792669
H -1.98092 -1.32248 -1.86088
Na 0.405575 1.117645 1.83432

Reactant state of 1st RO1' reaction of Manβ(1→4)Manβ
46

C 1.338187 -0.309131 -1.337976
O 1.158399 -1.154252 -0.231126
C 2.167236 -1.251274 0.766297
C 3.547815 -0.960831 0.176633
O 3.858808 -1.891346 -0.840653
C 3.608914 0.411701 -0.527026
C 2.227913 0.891012 -1.038613
O 1.605791 1.742464 -0.079811
O 4.083944 1.439205 0.327465
C 1.864718 -0.37963 2.010763
O 0.493265 -0.0482 2.133409
O -1.153039 2.417346 -0.82494
H 2.130916 -2.305537 1.069791
H 4.296887 -0.970821 0.981942
H 4.269842 0.278383 -1.389596
H 2.358274 1.449775 -1.973315
H 2.323663 2.308967 0.249914
H 5.046024 1.491981 0.30029
H 3.849531 -2.792251 -0.495153
H 2.412204 0.564234 1.96788
H 2.209426 -0.92206 2.899427
H -0.079652 -0.823711 1.963263
C -3.861183 -0.113082 -0.334035
O -3.26331 -0.997708 -1.224835
C -1.85757 -1.277427 -1.120668
C -1.036377 0.01032 -0.910508
O 0.065231 0.094156 -1.807781
C -1.903081 1.246823 -1.109225
C -3.048251 1.19166 -0.106716
O -2.470371 1.253508 1.19368
C -1.565154 -2.351712 -0.071519
O -1.494553 -1.84872 1.257997
O -4.083253 -0.686368 0.944268
H -1.605765 -1.681228 -2.105849
H -0.667827 0.010222 0.111433
H -3.704636 2.057329 -0.245625
H -4.821365 0.127371 -0.797452
H -4.836695 -1.289564 0.900524
H -3.106739 0.894743 1.828143
H -0.386134 2.4244 -1.416635
H -0.59036 -2.79242 -0.28147
H -2.331039 -3.131739 -0.148159
H -2.384241 -1.566632 1.521285
H -2.307492 1.282167 -2.128072
H 1.80199 -0.871422 -2.159003
Na -0.289373 1.913323 1.289147

Transition state of 2nd RO1' reaction of Manβ(1→4)Manβ
46

C 1.029658 -0.912542 1.699662
O 1.400424 1.025788 1.293429
C 2.525727 1.505879 0.524504
C 3.245756 0.383215 -0.228359
O 4.578309 0.817381 -0.335131
C 3.168823 -0.994569 0.428697
C 1.714584 -1.502298 0.50175
O 1.083372 -1.391764 -0.694615
O 3.931966 -1.884279 -0.355397
C 1.990409 2.559113 -0.445304
O 0.865299 1.990703 -1.138109
O -0.98273 -2.750696 -0.184277
H 3.229979 1.948205 1.233853
H 2.805718 0.272604 -1.23153
H 3.646155 -0.963591 1.416926
H 1.788972 -2.561619 0.859602
H 0.624932 1.181452 0.726908
H 3.375535 -2.175706 -1.094086
H 5.102075 0.089807 -0.701564
H 2.76894 2.819211 -1.166497
H 1.676552 3.461043 0.087568
H 0.039901 2.438353 -0.878757
C -3.808449 -0.259582 -0.281001
O -3.574754 0.085821 1.053292
C -2.256537 0.377464 1.510952
C -1.203619 -0.536576 0.860108
O -0.238506 -0.816277 1.914565
C -1.860675 -1.778734 0.250756
C -2.731843 -1.202415 -0.879861
O -1.893835 -0.504802 -1.811217
C -1.923685 1.858925 1.356489
O -1.634157 2.217872 0.009716
O -3.892226 0.884287 -1.116661
H -2.286653 0.142684 2.580283
H -0.671219 -0.011656 0.071936
H -3.217668 -2.033343 -1.399811
H -4.775735 -0.767026 -0.25725
H -4.761372 1.294792 -1.015974
H -2.491211 0.006617 -2.377014
H -0.148758 -2.307256 -0.542139
H -1.03122 2.095487 1.946081
H -2.755108 2.453888 1.748354
H -2.423798 2.043649 -0.532018
H -2.536891 -2.227108 0.990033
H 1.581362 -0.940426 2.640448
Na 0.361211 -0.058148 -2.180043

Reactant state of 2nd RO1' reaction of Manβ(1→4)Manβ
46

C 1.004802 -0.607025 1.334066
O 1.306256 0.708415 0.859292
C 2.71523 0.944247 0.789332
C 3.399234 -0.06203 -0.145802
O 4.781849 0.122087 0.024717
C 2.978441 -1.500438 0.154066
C 1.453407 -1.59901 0.257011
O 0.900955 -1.240631 -1.015452
O 3.441006 -2.273305 -0.943666
C 2.904129 2.370227 0.315386
O 2.250557 2.494673 -0.954633
O -1.420601 -2.724695 0.108375
H 3.157333 0.847871 1.7923
H 3.10743 0.159017 -1.18299
H 3.447948 -1.822162 1.093074
H 1.123263 -2.606362 0.537338
H 1.390682 -1.759046 -1.674756
H 3.641375 -3.176561 -0.669692
H 5.25381 -0.441491 -0.602102
H 3.975545 2.568342 0.22141
H 2.453993 3.060498 1.03587
H 2.512597 3.322046 -1.373686
C -4.059402 -0.025153 -0.012437
O -3.571915 0.558261 1.157812
C -2.167447 0.781941 1.301209
C -1.329941 -0.4096 0.809477
O -0.304236 -0.666934 1.760083
C -2.1943 -1.641479 0.556784
C -3.218573 -1.230874 -0.505864
O -2.454008 -0.92468 -1.66449
C -1.721014 2.097372 0.666523
O -1.552766 2.011194 -0.749196
O -4.13219 0.904042 -1.081467
H -2.02318 0.861339 2.382787
H -0.88654 -0.177181 -0.152608
H -3.885253 -2.074923 -0.714781
H -5.066656 -0.35528 0.254768
H -4.868537 1.508879 -0.920722
H -3.057328 -0.674938 -2.375577
H -1.122601 -2.510169 -0.788497
H -0.742145 2.366674 1.074913
H -2.437862 2.885385 0.920066
H -2.40886 1.758829 -1.140626
H -2.697922 -1.956976 1.475222
H 1.598243 -0.801335 2.238177
Na 0.453454 1.141915 -1.323583

Transition state of 3rd RO1' reaction of Manβ(1→4)Manβ
46

C 1.029576 -0.912676 1.699334
O 1.400411 1.025113 1.293664
C 2.526069 1.505446 0.525267
C 3.246198 0.383008 -0.22776
O 4.578892 0.816888 -0.333894
C 3.168719 -0.995119 0.42844
C 1.714321 -1.502437 0.501245
O 1.083027 -1.391255 -0.695016
O 3.931591 -1.884404 -0.356434
C 1.991352 2.55897 -0.444559
O 0.866533 1.990844 -1.138001
O -0.982957 -2.750464 -0.18504
H 3.229943 1.947496 1.23516
H 2.80645 0.273013 -1.231119
H 3.646019 -0.96505 1.416716
H 1.788387 -2.561886 0.858756
H 0.624845 1.181417 0.727349
H 3.374417 -2.176599 -1.094254
H 5.102561 0.089349 -0.70054
H 2.770271 2.818962 -1.165368
H 1.677515 3.460928 0.088279
H 0.041001 2.438319 -0.878881
C -3.80877 -0.259526 -0.280758
O -3.575033 0.085004 1.053746
C -2.256806 0.377259 1.51095
C -1.203761 -0.536651 0.860075
O -0.238623 -0.816665 1.914423
C -1.860853 -1.778589 0.25027
C -2.732014 -1.20185 -0.880158
O -1.894116 -0.503615 -1.81116
C -1.924195 1.858757 1.356031
O -1.633609 2.217171 0.009303
O -3.892747 0.88483 -1.115726
H -2.28663 0.142818 2.580361
H -0.671315 -0.011458 0.072143
H -3.217649 -2.032601 -1.40056
H -4.775976 -0.767137 -0.257272
H -4.761699 1.295541 -1.014183
H -2.491543 0.00784 -2.376854
H -0.148956 -2.306853 -0.542727
H -1.032301 2.09582 1.946273
H -2.756107 2.453672 1.746933
H -2.423144 2.043419 -0.532731
H -2.537166 -2.227146 0.989342
H 1.581338 -0.940999 2.640074
Na 0.361254 -0.056909 -2.180372

Reactant state of 3rd RO1' reaction of Manβ(1→4)Manβ
46

C -1.009773 0.63109 1.380992
O -1.277037 -0.686475 0.894455
C -2.681818 -0.946036 0.778022
C -3.324851 0.034492 -0.207625
O -4.709988 -0.184378 -0.149036
C -2.966397 1.481523 0.132525
C -1.454462 1.625948 0.299677
O -0.869532 1.329009 -0.97391
O -3.47968 2.348136 -0.849037
C -2.839987 -2.385886 0.338002
O -2.155416 -2.536421 -0.911961
O 1.309799 2.666554 0.083909
H -3.164014 -0.82602 1.759548
H -2.950186 -0.178518 -1.223117
H -3.463637 1.752071 1.072324
H -1.197952 2.644734 0.603585
H -0.063826 1.869589 -1.064603
H -2.894985 2.309778 -1.619735
H -5.15376 0.518735 -0.64403
H -3.907107 -2.596784 0.223325
H -2.400613 -3.052915 1.086728
H -2.392081 -3.380155 -1.312479
C 3.995028 0.012671 -0.114764
O 3.572145 -0.536976 1.095962
C 2.177421 -0.748557 1.337506
C 1.314814 0.424244 0.846438
O 0.310437 0.704873 1.805002
C 2.177238 1.635119 0.518334
C 3.109899 1.188167 -0.613914
O 2.27807 0.825526 -1.701746
C 1.684003 -2.078181 0.771234
O 1.478189 -2.038258 -0.641115
O 4.036356 -0.946205 -1.156932
H 2.098992 -0.787315 2.428109
H 0.852887 0.144279 -0.08959
H 3.765643 2.016948 -0.910978
H 5.007034 0.370045 0.093613
H 4.798862 -1.525218 -1.02493
H 2.839276 0.472762 -2.404778
H 1.777519 3.508752 0.03374
H 0.711801 -2.307596 1.21586
H 2.392359 -2.87087 1.033295
H 2.334151 -1.852854 -1.065783
H 2.756124 1.950991 1.394316
H -1.601331 0.805421 2.289463
Na -0.470781 -1.072409 -1.330652

Transition state of 1st c0',3' reaction of Manβ(1→4)Manβ
46

C -0.925909 -1.552506 -1.08684
O -1.833095 0.718932 -1.370718
C -3.199512 0.828111 -1.006166
C -3.637347 -0.420303 -0.211004
O -3.774795 -1.546336 -1.068308
C -2.682131 -0.843314 0.911229
C -1.37488 -1.901559 0.226355
O -0.458517 -1.919495 1.290969
O -2.144332 0.113017 1.612334
C -3.443253 2.189065 -0.335455
O -2.470934 2.473736 0.652778
O 1.920018 -2.607968 0.104873
H -3.773987 0.812239 -1.940707
H -4.595833 -0.199777 0.282273
H -3.135999 -1.658785 1.497723
H -1.956115 -2.82233 0.123471
H 0.289284 -2.504371 1.074673
H -1.329394 1.05938 -0.611997
H -4.625338 -1.527647 -1.521117
H -4.456292 2.224796 0.087201
H -3.363095 2.968366 -1.096767
H -2.442537 1.665571 1.231482
C 3.923591 0.478829 -0.678079
O 2.922111 0.952844 -1.552617
C 1.573042 0.921043 -1.106048
C 1.172942 -0.483434 -0.61026
O 0.183404 -1.044152 -1.505595
C 2.358452 -1.427163 -0.543809
C 3.441415 -0.678601 0.220479
O 2.86478 -0.209672 1.428773
C 1.323043 2.028713 -0.055001
O 0.155971 1.731586 0.706996
O 4.410321 1.462546 0.188069
H 0.974872 1.124322 -1.99954
H 0.724039 -0.416007 0.365513
H 4.292068 -1.336717 0.437764
H 4.713683 0.121644 -1.347508
H 4.911768 2.12435 -0.304719
H 3.503895 0.3964 1.83234
H 2.535803 -3.334811 -0.047279
H 1.21841 2.98551 -0.572467
H 2.176737 2.093015 0.619121
H -0.555035 2.396251 0.608198
H 2.709358 -1.648843 -1.559573
H -1.600936 -1.799267 -1.901784
Na -0.144685 0.131193 2.408262

Reactant state of 1st c0',3' reaction of Manβ(1→4)Manβ
46

C -1.20571 -0.8391 -1.243332
O -1.473721 0.541669 -1.004322
C -2.868157 0.810732 -0.974425
C -3.62278 -0.190441 -0.053811
O -4.248271 -1.202896 -0.819996
C -2.653267 -0.950557 0.856743
C -1.633205 -1.706363 -0.010567
O -0.590724 -2.092206 0.866053
O -1.967239 -0.092742 1.76219
C -3.029282 2.26198 -0.573391
O -2.502309 2.408459 0.745853
O 1.845735 -2.626644 -0.144011
H -3.281645 0.691962 -1.988435
H -4.355816 0.3467 0.56448
H -3.22519 -1.679502 1.438814
H -2.124932 -2.600505 -0.408392
H 0.084303 -2.608583 0.389025
H -0.570585 1.677143 0.697925
H -5.103221 -0.907661 -1.152469
H -4.091159 2.532125 -0.599129
H -2.476604 2.896531 -1.274176
H -2.318426 0.810847 1.700655
C 3.862107 0.490249 -0.620027
O 2.897627 1.068147 -1.463893
C 1.525785 0.999666 -1.063064
C 1.082839 -0.444055 -0.745903
O 0.116477 -0.971327 -1.646166
C 2.270647 -1.391715 -0.699804
C 3.330597 -0.723058 0.161051
O 2.720017 -0.334561 1.391292
C 1.275604 2.002409 0.104679
O 0.321382 1.576714 1.06785
O 4.367236 1.379287 0.341502
H 0.973311 1.311301 -1.951679
H 0.634215 -0.424486 0.228941
H 4.160938 -1.408989 0.366261
H 4.660115 0.165145 -1.297216
H 4.871632 2.080944 -0.088625
H 3.333282 0.280406 1.822203
H 2.385334 -3.353498 -0.474277
H 0.975861 2.967724 -0.316556
H 2.210056 2.149994 0.642434
H -2.637072 3.306337 1.072076
H 2.657713 -1.53751 -1.715971
H -1.806522 -1.16287 -2.102739
Na 0.285788 -0.42575 2.258831

Transition state of 2nd c0',3' reaction of Manβ(1→4)Manβ
46

C 0.75555 1.335226 0.088436
O 1.034089 -0.378658 -1.118235
C 2.316604 -0.547734 -1.739476
C 3.32206 0.415268 -1.092938
O 3.127005 1.736795 -1.591989
C 3.168586 0.509207 0.437411
C 2.020955 1.582453 0.806641
O 1.763594 1.484372 2.197199
O 2.930366 -0.662187 1.060319
C 2.658168 -2.050897 -1.650903
O 2.213988 -2.570858 -0.421585
O -0.569382 -1.72418 1.094872
H 2.208579 -0.25838 -2.791095
H 4.336988 0.062191 -1.316703
H 4.074657 1.022657 0.817005
H 2.365592 2.58015 0.516378
H 2.59414 1.659804 2.660175
H 0.959614 -1.157153 -0.519273
H 3.562894 1.847076 -2.444431
H 3.73741 -2.192964 -1.794271
H 2.133241 -2.586882 -2.445863
H 2.647359 -1.971339 0.266562
C -3.888942 -0.631708 -0.227752
O -3.826338 0.604746 0.438134
C -2.594306 1.324888 0.366408
C -1.419167 0.356119 0.194477
O -0.208332 0.873444 0.80554
C -1.713866 -0.895031 1.014252
C -2.920774 -1.650203 0.423582
O -2.466419 -2.628831 -0.476335
C -2.668468 2.385733 -0.723278
O -1.407567 3.038939 -0.720822
O -3.517026 -0.530826 -1.579539
H -2.47579 1.821181 1.336804
H -1.202604 0.128401 -0.853144
H -3.442061 -2.167367 1.233371
H -4.927064 -0.954369 -0.125946
H -4.262538 -0.233027 -2.11561
H -2.552892 -2.289813 -1.380671
H -0.702181 -2.489775 0.504746
H -3.485866 3.074938 -0.48745
H -2.862366 1.910486 -1.691754
H -1.465284 3.871749 -1.202155
H -1.957903 -0.565363 2.031985
H 0.554493 1.775193 -0.881286
Na 1.21376 -0.875396 2.345544

Reactant state of 2nd c0',3' reaction of Manβ(1→4)Manβ
46

C 0.841929 0.874574 0.180341
O 1.148017 -0.319328 -0.547613
C 2.172359 -0.099409 -1.51062
C 3.391716 0.605938 -0.871923
O 3.339074 1.961928 -1.27717
C 3.305509 0.526856 0.65794
C 2.009782 1.217955 1.143554
O 1.678121 0.741667 2.439785
O 3.2975 -0.814236 1.1267
C 2.509084 -1.431552 -2.14839
O 3.153171 -2.255314 -1.18048
O -1.045165 -2.028858 1.009668
H 1.792901 0.570101 -2.296132
H 4.318541 0.127648 -1.21706
H 4.170998 1.046456 1.093251
H 2.14026 2.303834 1.153869
H 2.384749 0.971177 3.056037
H 3.494855 -3.05699 -1.592556
H 4.195602 2.387114 -1.150143
H 3.170663 -1.249002 -3.003234
H 1.586177 -1.904631 -2.50068
H 3.576674 -1.415178 0.41365
C -4.092633 -0.322325 -0.303869
O -3.858396 0.850641 0.420014
C -2.520137 1.365822 0.412849
C -1.477982 0.246092 0.258632
O -0.278245 0.598937 0.946682
C -2.014306 -0.993461 0.969942
C -3.302684 -1.508523 0.297114
O -2.970489 -2.47852 -0.673248
C -2.424494 2.470835 -0.63143
O -1.131756 3.044178 -0.521767
O -3.694722 -0.211777 -1.652474
H -2.365258 1.806941 1.403636
H -1.26017 0.02437 -0.793506
H -3.922246 -2.00181 1.05075
H -5.169541 -0.491634 -0.231395
H -4.362441 0.26908 -2.156655
H -3.027327 -2.065493 -1.548345
H -1.270541 -2.638953 0.285334
H -3.21083 3.204685 -0.421701
H -2.583512 2.049488 -1.631925
H -1.104549 3.875629 -1.007351
H -2.22998 -0.709849 2.006485
H 0.667443 1.691559 -0.530305
Na 1.047437 -1.384174 1.652033

Transition state of 3rd c0',3' reaction of Manβ(1→4)Manβ
46

C 0.751065 1.383571 0.059846
O 1.003994 -0.382572 -1.15346
C 2.291182 -0.57384 -1.753327
C 3.303532 0.378239 -1.098372
O 3.14102 1.698791 -1.613366
C 3.133031 0.482281 0.429796
C 2.02169 1.613117 0.775527
O 1.843836 1.509981 2.177459
O 2.833614 -0.67764 1.038734
C 2.6107 -2.082129 -1.658668
O 2.138454 -2.600202 -0.439174
O -0.520995 -1.654438 1.186512
H 2.203149 -0.287207 -2.807677
H 4.316143 0.006788 -1.302771
H 4.036387 0.967984 0.840176
H 2.432042 2.576743 0.463845
H 1.534819 2.351284 2.533865
H 0.928228 -1.134948 -0.523884
H 3.589055 1.790155 -2.461691
H 3.69034 -2.239157 -1.783785
H 2.092355 -2.609667 -2.463493
H 2.55555 -2.002163 0.256928
C -3.844468 -0.687741 -0.230456
O -3.823734 0.569826 0.399393
C -2.608274 1.316268 0.326509
C -1.410205 0.371061 0.191132
O -0.213643 0.936448 0.784793
C -1.682296 -0.858786 1.052343
C -2.859103 -1.661644 0.463586
O -2.364585 -2.65088 -0.402152
C -2.690132 2.350817 -0.786979
O -1.452701 3.048966 -0.762619
O -3.458096 -0.613508 -1.579612
H -2.515279 1.837953 1.286729
H -1.187137 0.114336 -0.847995
H -3.379672 -2.171364 1.278617
H -4.875157 -1.034265 -0.132335
H -4.206931 -0.362392 -2.134507
H -2.436865 -2.336121 -1.31638
H -0.611859 -2.43671 0.61001
H -3.537112 3.015866 -0.588566
H -2.839935 1.84844 -1.749628
H -1.515127 3.846683 -1.299874
H -1.955159 -0.500103 2.052865
H 0.553976 1.783596 -0.928088
Na 1.273685 -0.790109 2.481815

Reactant state of 3rd c0',3' reaction of Manβ(1→4)Manβ
46

C 0.834117 0.891882 0.128178
O 1.141582 -0.324861 -0.552242
C 2.186556 -0.133494 -1.502428
C 3.395064 0.595416 -0.866292
O 3.34337 1.935785 -1.320453
C 3.293677 0.564232 0.664486
C 1.992201 1.265478 1.096123
O 1.729451 0.822086 2.41709
O 3.286603 -0.766841 1.160582
C 2.531178 -1.483549 -2.096556
O 3.145117 -2.288017 -1.093926
O -1.007463 -1.995299 1.050721
H 1.820232 0.511381 -2.314337
H 4.327299 0.108356 -1.18435
H 4.142821 1.09996 1.106114
H 2.136316 2.349549 1.0708
H 0.99187 1.336495 2.769195
H 3.498247 -3.09696 -1.481583
H 4.187257 2.377265 -1.165727
H 3.214308 -1.325163 -2.939187
H 1.61439 -1.959694 -2.460797
H 3.597448 -1.376786 0.469847
C -4.078281 -0.372978 -0.315658
O -3.875237 0.817533 0.390153
C -2.547745 1.356628 0.389696
C -1.485902 0.254727 0.251799
O -0.285104 0.64783 0.916446
C -1.996344 -0.981479 0.987
C -3.271623 -1.532536 0.315245
O -2.914059 -2.514505 -0.63302
C -2.456917 2.456729 -0.659663
O -1.17942 3.058523 -0.523575
O -3.66735 -0.276338 -1.660882
H -2.41104 1.809271 1.378531
H -1.270518 0.017874 -0.797145
H -3.888199 -2.021845 1.073862
H -5.152268 -0.562365 -0.251338
H -4.341086 0.176943 -2.182421
H -2.96976 -2.120099 -1.516813
H -1.218084 -2.623864 0.337479
H -3.263857 3.173958 -0.471749
H -2.585177 2.025219 -1.660397
H -1.152857 3.878118 -1.029253
H -2.225247 -0.684296 2.017224
H 0.649197 1.682895 -0.609325
Na 1.107537 -1.362701 1.662058

Transition state of 1st RO2' reaction of Manβ(1→4)Manβ
46

C -0.785966 -2.10083 -1.164618
O -0.954613 1.008325 0.321527
C -2.132784 1.07678 -0.463853
C -3.185133 0.127041 0.126318
O -4.456574 0.570032 -0.294783
C -3.01685 -1.342205 -0.299912
C -1.584819 -1.906699 0.083624
O -1.003216 -1.308358 1.144085
O -4.023886 -2.033039 0.401733
C -2.621979 2.519214 -0.500915
O -3.002993 2.970182 0.778071
O 1.177124 -2.55882 1.170036
H -1.907774 0.776459 -1.506595
H -3.103586 0.161616 1.2211
H -3.196316 -1.398756 -1.384083
H -1.794289 -2.993394 0.26915
H -0.961869 0.089918 0.807033
H -4.318318 -2.816295 -0.076577
H -5.115972 -0.03685 0.069883
H -3.432911 2.616918 -1.229108
H -1.787458 3.14866 -0.830825
H -3.912111 2.68674 0.937859
C 3.796198 -0.002292 0.323805
O 3.551081 -0.141558 -1.062409
C 2.235607 -0.225136 -1.605073
C 1.271547 -0.929975 -0.645277
O 0.390031 -1.762418 -1.478568
C 2.012653 -1.780957 0.386856
C 2.768647 -0.727056 1.215488
O 1.835138 0.187418 1.793978
C 1.740728 1.164904 -1.999872
O 1.806635 2.052181 -0.89522
O 3.895724 1.348032 0.707457
H 2.340513 -0.842293 -2.504912
H 0.627666 -0.210692 -0.157124
H 3.308677 -1.233261 2.021133
H 4.764906 -0.491834 0.45393
H 4.807028 1.647214 0.596613
H 1.331067 -0.314254 2.452339
H 0.30115 -2.069641 1.330714
H 0.692967 1.118042 -2.314397
H 2.340241 1.549245 -2.831261
H 2.738224 2.122691 -0.626092
H 2.738714 -2.433734 -0.114632
H -1.251745 -2.695095 -1.957198
Na 0.735318 2.156914 1.086032

Reactant state of 1st RO2' reaction of Manβ(1→4)Manβ
46

C -1.004713 -0.607425 -1.334193
O -1.306397 0.70812 -0.859911
C -2.715352 0.943779 -0.789765
C -3.399074 -0.062283 0.145773
O -4.781764 0.121591 -0.024609
C -2.97821 -1.500728 -0.153753
C -1.453174 -1.599139 -0.256814
O -0.900853 -1.240264 1.015579
O -3.44036 -2.273317 0.944364
C -2.904492 2.369859 -0.316205
O -2.251206 2.494769 0.953909
O 1.421642 -2.724635 -0.108617
H -3.157629 0.847061 -1.792633
H -3.107098 0.159104 1.182849
H -3.447885 -1.822791 -1.092547
H -1.12284 -2.606507 -0.536807
H -1.389787 -1.759477 1.674837
H -3.642294 -3.176206 0.670376
H -5.253522 -0.440886 0.603341
H -3.975976 2.567757 -0.222505
H -2.454374 3.060036 -1.03678
H -2.51385 3.321951 1.372967
C 4.059606 -0.024193 0.011746
O 3.571403 0.559284 -1.158161
C 2.166771 0.78271 -1.300603
C 1.329916 -0.409525 -0.8094
O 0.304337 -0.667086 -1.760177
C 2.194834 -1.641047 -0.55725
C 3.219554 -1.230496 0.505027
O 2.455503 -0.925159 1.664193
C 1.72016 2.097599 -0.664884
O 1.55185 2.010559 0.75079
O 4.132132 0.904666 1.081083
H 2.022021 0.862904 -2.38205
H 0.886099 -0.177763 0.152657
H 3.88668 -2.074388 0.713149
H 5.066976 -0.353647 -0.255844
H 4.867955 1.510115 0.920242
H 3.059071 -0.675952 2.375257
H 1.12489 -2.510798 0.788838
H 0.741239 2.366961 -1.073124
H 2.436824 2.885937 -0.917929
H 2.407933 1.75803 1.142133
H 2.698092 -1.956241 -1.475983
H -1.598156 -0.802242 -2.238198
Na -0.45451 1.141285 1.32383

Transition state of 2nd RO2' reaction of Manβ(1→4)Manβ
46

C -0.938025 -1.587409 0.167015
O -1.287818 1.220272 0.860288
C -1.771771 0.929412 -0.373719
C -3.239941 0.445718 -0.336011
O -3.926383 0.876524 -1.491991
C -3.337165 -1.083211 -0.255436
C -2.309871 -1.676567 0.74093
O -2.414657 -1.113325 2.004821
O -4.633379 -1.490357 0.083316
C -1.614788 2.064214 -1.389401
O -0.244293 2.49478 -1.359584
O 0.980462 1.038218 2.08805
H -1.174502 0.055594 -0.842851
H -3.683825 0.888384 0.567994
H -3.143657 -1.483307 -1.259567
H -2.548923 -2.750851 0.809796
H -2.08821 -0.186837 1.945533
H -4.805687 -1.253029 1.006825
H -4.832727 0.541202 -1.456969
H -2.277234 2.887603 -1.09595
H -1.8894 1.729261 -2.39131
H -0.085522 3.050123 -2.131371
C 3.760303 -0.0729 -0.191952
O 3.559127 -1.40698 0.167042
C 2.215406 -1.902352 0.157657
C 1.259373 -0.744458 0.448124
O -0.020139 -1.207576 0.956602
C 1.88369 0.14337 1.512395
C 3.007624 0.902215 0.769549
O 2.453562 2.002592 0.073173
C 1.872944 -2.662044 -1.132657
O 1.198079 -1.877597 -2.095002
O 3.308452 0.172257 -1.517038
H 2.17781 -2.601211 1.001385
H 1.019178 -0.158368 -0.447455
H 3.71631 1.291571 1.50524
H 4.839313 0.076145 -0.130447
H 4.036111 0.041597 -2.138665
H 2.50519 1.823307 -0.878188
H 0.060789 0.864542 1.741863
H 1.19559 -3.488841 -0.895035
H 2.796706 -3.100055 -1.526779
H 1.792944 -1.183119 -2.410708
H 2.328527 -0.485509 2.294523
H -0.678103 -1.91372 -0.846002
Na 0.384414 2.831737 0.792888

Reactant state of 2nd RO2' reaction of Manβ(1→4)Manβ
46

C -1.17736 -0.724939 -0.972899
O -1.125962 0.427258 -0.136071
C -2.333609 1.198661 -0.20676
C -3.532293 0.363657 0.238604
O -4.66932 1.178866 0.085574
C -3.638125 -0.904716 -0.604258
C -2.314115 -1.657107 -0.556933
O -2.155128 -2.085612 0.7733
O -4.713485 -1.691936 -0.156992
C -2.161389 2.397983 0.700049
O -1.048518 3.20284 0.281526
O 0.574594 -1.644964 1.848515
H -2.489437 1.534508 -1.244688
H -3.388667 0.071014 1.29016
H -3.86983 -0.617114 -1.638232
H -2.332674 -2.50586 -1.255008
H -1.243244 -2.379667 0.930555
H -4.41646 -2.196682 0.615087
H -5.453911 0.619782 0.180362
H -1.959654 2.056306 1.7214
H -3.087885 2.975423 0.702944
H -1.357177 4.049619 -0.057519
C 3.936603 -0.696549 0.423101
O 3.574661 -1.161071 -0.846151
C 2.297993 -0.856068 -1.41974
C 1.173887 -0.870319 -0.373862
O 0.005604 -1.48292 -0.8629
C 1.626667 -1.525341 0.927029
C 2.775169 -0.66611 1.447802
O 2.262364 0.65787 1.659358
C 2.339094 0.468146 -2.173635
O 2.476581 1.577006 -1.284693
O 4.500301 0.601972 0.381831
H 2.116066 -1.668362 -2.130144
H 0.930152 0.148105 -0.120183
H 3.128297 -1.058988 2.406007
H 4.693486 -1.411011 0.758834
H 5.409506 0.542016 0.059046
H 2.944377 1.153364 2.132433
H 0.231506 -0.767643 2.070379
H 1.396749 0.610502 -2.712608
H 3.157096 0.461077 -2.900515
H 3.337762 1.495659 -0.835324
H 1.990427 -2.54108 0.741843
H -1.30891 -0.387992 -2.015038
Na 0.923979 2.183524 0.252985

Transition state of 3rd RO2' reaction of Manβ(1→4)Manβ
46

C 1.089328 -1.70085 0.112792
O 1.421849 0.889942 -0.989233
C 1.925044 0.966208 0.285823
C 3.376946 0.460892 0.360734
O 4.007259 0.996519 1.505015
C 3.468047 -1.072697 0.427874
C 2.437387 -1.776636 -0.5111
O 2.4585 -1.266454 -1.789917
O 4.761323 -1.513738 0.119718
C 1.820482 2.373978 0.870045
O 0.459058 2.799602 0.70558
O -1.066275 0.287644 -2.37221
H 1.319983 0.316071 1.004089
H 3.872976 0.806835 -0.559228
H 3.287427 -1.372428 1.468515
H 2.730284 -2.84183 -0.501571
H 2.085303 -0.305767 -1.671981
H 4.938061 -1.330194 -0.815084
H 4.922652 0.687407 1.532663
H 2.48954 3.041157 0.312466
H 2.100679 2.380309 1.924685
H 0.243723 3.449722 1.381675
C -3.94649 -0.473077 -0.126155
O -3.34486 -1.668624 0.29438
C -1.98275 -1.686164 0.724427
C -1.125306 -0.803807 -0.197734
O 0.12212 -1.42206 -0.64175
C -1.855054 -0.483112 -1.498637
C -3.067221 0.350194 -1.110886
O -2.558689 1.548462 -0.547959
C -1.838902 -1.293144 2.198674
O -1.842961 0.096044 2.402585
O -4.284399 0.377698 0.945359
H -1.688782 -2.735914 0.60116
H -0.812287 0.115688 0.310062
H -3.653196 0.584145 -2.006198
H -4.851579 -0.810805 -0.635919
H -5.104406 0.078213 1.359837
H -3.219718 1.917965 0.054864
H -0.201663 -0.138533 -2.491125
H -0.876722 -1.657555 2.577724
H -2.627715 -1.804557 2.763996
H -2.735082 0.432495 2.235767
H -2.179618 -1.414701 -1.980428
H 0.917098 -1.863728 1.180992
Na -0.348128 2.208637 -1.301331

Reactant state of 3rd RO2' reaction of Manβ(1→4)Manβ
46

C 1.000086 -0.882222 0.12703
O 1.276968 0.497896 -0.125661
C 2.440031 0.964871 0.57551
C 3.667634 0.170506 0.136926
O 4.761219 0.664322 0.869426
C 3.448683 -1.314231 0.409259
C 2.167897 -1.782655 -0.271987
O 2.40767 -1.717947 -1.661464
O 4.565834 -2.059411 -0.002265
C 2.625391 2.432538 0.261095
O 1.453503 3.153454 0.666042
O -0.945457 1.016139 -1.983418
H 2.286511 0.839946 1.659257
H 3.812357 0.314816 -0.945767
H 3.361633 -1.455593 1.494444
H 1.934216 -2.808318 0.042561
H 1.719113 -2.202965 -2.131785
H 4.508598 -2.182355 -0.961704
H 5.500499 0.048974 0.760028
H 2.781734 2.563969 -0.816584
H 3.50927 2.784991 0.796896
H 1.699802 4.022082 1.001465
C -4.134165 -0.151374 -0.397042
O -3.660048 -1.458873 -0.304696
C -2.333204 -1.743522 0.164884
C -1.333538 -0.675388 -0.293219
O -0.088332 -1.263517 -0.65182
C -1.891516 0.082716 -1.48966
C -3.110555 0.854643 -1.005118
O -2.652286 1.811785 -0.054688
C -2.295067 -1.936443 1.678177
O -2.340669 -0.715838 2.384778
O -4.5393 0.375214 0.852391
H -2.075825 -2.687703 -0.326284
H -1.172602 0.024195 0.533953
H -3.578513 1.38454 -1.841055
H -5.000521 -0.231791 -1.057874
H -5.423759 0.054732 1.072094
H -3.348064 1.93864 0.60845
H -0.144439 0.526347 -2.226856
H -1.347225 -2.416307 1.945877
H -3.108176 -2.611458 1.970587
H -3.213533 -0.316954 2.260995
H -2.18829 -0.616829 -2.281045
H 0.774398 -0.995497 1.202101
Na -0.428187 2.316375 -0.185808

Transition state of 1st c2 reaction of GlcNAcβ(1→2)Manβ
52

C -1.412538 -0.985647 -3.036051
C -0.954662 -1.019177 -1.731509
C -2.828825 -0.818164 -0.949576
O -3.53304 -1.758441 -1.440304
C -2.415198 -0.981719 0.511692
C -3.497253 -0.365101 1.4228
C -3.067674 -0.410538 2.877288
O -1.945866 0.475415 3.011365
O -3.695685 1.016795 1.086791
O -2.257563 -2.336806 0.822295
O -2.244107 -1.872146 -3.481572
H -4.429827 -0.929324 1.306826
H -1.200984 -0.12588 -3.677977
H -2.998735 0.221251 -1.269841
H -1.465031 -0.468917 0.70148
H -4.478094 1.126842 0.535463
H -3.885346 -0.069977 3.520329
H -2.784046 -1.435243 3.132057
H -2.86193 -2.829839 0.239989
H -2.912575 -2.086204 -2.688195
H -1.485135 0.293068 3.837451
C 1.121875 -0.13367 -1.066374
C 1.693382 1.07665 -0.326726
N 1.482563 2.325103 -1.029595
C 3.178097 0.829322 -0.097981
O 3.700981 1.916217 0.6202
C 3.394025 -0.487688 0.641491
C 2.663009 -1.630878 -0.076953
C 2.625157 -2.915175 0.739501
O 3.961461 -3.222733 1.099762
O 1.295614 -1.275709 -0.269089
O 4.789634 -0.652878 0.675614
O -0.248758 0.075029 -1.255993
H 3.152886 -1.814453 -1.046446
H 1.615908 -0.283033 -2.044556
H 1.177202 1.144215 0.636614
H 3.686227 0.735892 -1.075816
H 2.97705 -0.387081 1.658334
H 2.001861 -2.743971 1.627171
H 2.174677 -3.71344 0.139606
H 4.993231 -1.522757 1.050917
H 4.613841 1.696786 0.857129
H 4.005096 -4.079137 1.538216
H -0.813449 -1.958513 -1.201471
C 0.505709 3.19131 -0.70121
H 2.225792 2.661394 -1.627989
O -0.355378 2.919439 0.146508
C 0.504091 4.521813 -1.405235
H 1.262069 4.596257 -2.185689
H 0.67652 5.303696 -0.661768
H -0.482914 4.686207 -1.841337
Na -1.81234 2.200449 1.528647

Reactant state of 1st c2 reaction of GlcNAcβ(1→2)Manβ
52

C 1.111415 -0.803537 3.220042
C 0.994784 -0.970822 1.713264
C 2.371783 -0.917758 1.033228
O 3.228307 -1.949447 1.463307
C 2.22833 -1.098654 -0.477835
C 3.495276 -0.668533 -1.236418
C 3.267257 -0.701796 -2.7363
O 2.296484 0.314393 -3.03404
O 3.836272 0.686083 -0.899876
O 1.921816 -2.434771 -0.794576
O 2.05787 -1.202978 3.857357
H 4.318473 -1.345458 -0.981025
H 0.279482 -0.264041 3.713761
H 2.796187 0.074426 1.247093
H 1.384111 -0.491426 -0.823558
H 4.539891 0.705265 -0.242322
H 4.200913 -0.484831 -3.264198
H 2.889622 -1.688415 -3.018053
H 2.456479 -3.001042 -0.216086
H 3.269786 -1.9458 2.432877
H 1.9552 0.186364 -3.9256
C -1.112032 -0.049787 1.01678
C -1.634812 1.141201 0.209128
N -1.363602 2.418286 0.836174
C -3.130013 0.94776 0.00258
O -3.614057 2.019016 -0.764517
C -3.404389 -0.394026 -0.669228
C -2.720375 -1.530747 0.104309
C -2.734096 -2.851105 -0.653582
O -4.081783 -3.120666 -1.002578
O -1.339316 -1.227158 0.285967
O -4.805981 -0.503371 -0.690199
O 0.260115 0.121777 1.181599
H -3.221251 -1.64821 1.078783
H -1.621573 -0.121065 1.997471
H -1.119742 1.125423 -0.757496
H -3.633329 0.923961 0.987202
H -2.988991 -0.362161 -1.690937
H -2.102947 -2.744946 -1.545568
H -2.317069 -3.639951 -0.01782
H -5.046148 -1.379148 -1.02832
H -4.535374 1.823511 -0.989397
H -4.154103 -3.981369 -1.428701
H 0.510354 -1.92891 1.47911
C -0.32589 3.200264 0.481528
H -2.105609 2.843505 1.376742
O 0.540468 2.821284 -0.317841
C -0.263171 4.573066 1.096993
H -1.046573 4.750082 1.834636
H -0.349364 5.312405 0.297239
H 0.712967 4.701423 1.568485
Na 2.109804 2.018724 -1.531026

Transition state of 2nd c2 reaction of GlcNAcβ(1→2)Manβ
52

C -1.422996 -0.970739 -3.040147
C -0.959931 -1.010434 -1.737588
C -2.830427 -0.81183 -0.947151
O -3.537385 -1.749493 -1.439029
C -2.410823 -0.981913 0.511653
C -3.4882 -0.367854 1.429996
C -3.052005 -0.41921 2.882311
O -1.928851 0.465343 3.014741
O -3.687012 1.015497 1.100354
O -2.253323 -2.33846 0.81594
O -2.257114 -1.85457 -3.486159
H -4.421819 -0.9308 1.316208
H -1.213308 -0.108325 -3.679111
H -3.000959 0.229091 -1.262164
H -1.459323 -0.47092 0.699537
H -4.470773 1.128084 0.551458
H -3.866433 -0.08048 3.530408
H -2.768064 -1.445113 3.131848
H -2.860645 -2.828387 0.234081
H -2.922465 -2.071744 -2.690979
H -1.464247 0.279017 3.837763
C 1.118665 -0.13067 -1.070555
C 1.691985 1.076257 -0.326715
N 1.483918 2.327267 -1.025766
C 3.176073 0.825452 -0.097704
O 3.70042 1.9091 0.624315
C 3.389086 -0.494288 0.637754
C 2.65649 -1.633846 -0.084823
C 2.615545 -2.92067 0.727519
O 3.950808 -3.231308 1.088979
O 1.289888 -1.275514 -0.276736
O 4.78436 -0.66209 0.67233
O -0.25148 0.08119 -1.260033
H 3.146677 -1.8152 -1.054572
H 1.612927 -0.27781 -2.048939
H 1.175456 1.14152 0.636602
H 3.684726 0.734187 -1.075467
H 2.97157 -0.396173 1.654627
H 1.991022 -2.751357 1.614692
H 2.164882 -3.716407 0.124418
H 4.986124 -1.533383 1.045338
H 4.612723 1.687261 0.861161
H 3.992539 -4.089313 1.524478
H -0.817299 -1.952201 -1.212283
C 0.508229 3.194314 -0.695998
H 2.228316 2.663969 -1.622478
O -0.354562 2.921848 0.149787
C 0.51008 4.526837 -1.396209
H 1.267114 4.600974 -2.17761
H 0.686369 5.305997 -0.650767
H -0.477071 4.695821 -1.830174
Na -1.802008 2.197277 1.539303

Reactant state of 2nd c2 reaction of GlcNAcβ(1→2)Manβ
52

C 1.111327 -0.802506 3.219985
C 0.99498 -0.970345 1.713291
C 2.371997 -0.917338 1.033311
O 3.228856 -1.948592 1.463872
C 2.228631 -1.09892 -0.477698
C 3.495431 -0.66861 -1.236381
C 3.267268 -0.701938 -2.73625
O 2.296249 0.314092 -3.033725
O 3.836196 0.686095 -0.899922
O 1.922743 -2.435271 -0.794006
O 2.057889 -1.201377 3.857504
H 4.318726 -1.345404 -0.980972
H 0.279273 -0.263052 3.71348
H 2.796215 0.075026 1.246657
H 1.384173 -0.49219 -0.823709
H 4.540432 0.70538 -0.243038
H 4.200834 -0.484697 -3.264204
H 2.88981 -1.688607 -3.018058
H 2.457158 -3.001059 -0.214813
H 3.269881 -1.944913 2.43347
H 1.954807 0.186212 -3.925246
C -1.111845 -0.049636 1.016505
C -1.634951 1.141163 0.208806
N -1.364062 2.418408 0.835648
C -3.130135 0.94732 0.00239
O -3.614471 2.018338 -0.764852
C -3.404345 -0.394642 -0.669161
C -2.72001 -1.531088 0.10448
C -2.7335 -2.851639 -0.653082
O -4.081181 -3.121787 -1.001615
O -1.338985 -1.22713 0.285796
O -4.805918 -0.50417 -0.689953
O 0.260283 0.122087 1.181041
H -3.220662 -1.648468 1.079079
H -1.621231 -0.121005 1.997254
H -1.119955 1.125451 -0.757863
H -3.633356 0.9236 0.987066
H -2.989065 -0.362867 -1.690924
H -2.102648 -2.745497 -1.545283
H -2.315983 -3.640155 -0.01724
H -5.046059 -1.380018 -1.027899
H -4.535757 1.822577 -0.989638
H -4.153417 -3.982966 -1.426788
H 0.510572 -1.928482 1.479293
C -0.326632 3.200698 0.480858
H -2.106004 2.843316 1.376539
O 0.539812 2.821938 -0.318528
C -0.264332 4.57357 1.096205
H -1.047582 4.750316 1.834074
H -0.351145 5.312784 0.296398
H 0.711902 4.702449 1.567348
Na 2.109312 2.018463 -1.530815

Transition state of 3rd c2 reaction of GlcNAcβ(1→2)Manβ
52

C -1.440349 -0.946467 -3.047393
C -0.967863 -0.995787 -1.748524
C -2.831963 -0.801062 -0.942645
O -3.543969 -1.73417 -1.436049
C -2.401589 -0.982432 0.511639
C -3.471398 -0.374632 1.442921
C -3.02365 -0.436651 2.891289
O -1.899125 0.446626 3.021398
O -3.67263 1.011159 1.125434
O -2.242894 -2.341408 0.804489
O -2.278848 -1.8262 -3.493283
H -4.406093 -0.936505 1.33262
H -1.234256 -0.079907 -3.681873
H -3.003403 0.242413 -1.24863
H -1.44831 -0.473557 0.69614
H -4.458407 1.127422 0.580173
H -3.832762 -0.102412 3.548299
H -2.738097 -1.464405 3.131131
H -2.85534 -2.826343 0.223811
H -2.938632 -2.048104 -2.694703
H -1.427364 0.253116 3.838664
C 1.113886 -0.124688 -1.078592
C 1.689062 1.076618 -0.327048
N 1.485038 2.332167 -1.019014
C 3.172101 0.820969 -0.096813
O 3.697634 1.899183 0.6325
C 3.380643 -0.503465 0.631493
C 2.646425 -1.637121 -0.098648
C 2.600776 -2.928377 0.706384
O 3.934519 -3.244124 1.069014
O 1.281059 -1.274238 -0.290694
O 4.775421 -0.674883 0.667512
O -0.255399 0.092015 -1.268492
H 3.137719 -1.814244 -1.068613
H 1.609173 -0.267708 -2.057029
H 1.171103 1.137235 0.635823
H 3.682353 0.734162 -1.07413
H 2.961629 -0.410066 1.648214
H 1.974642 -2.762598 1.593097
H 2.14958 -3.71968 0.097881
H 4.974413 -1.548774 1.035913
H 4.609142 1.674052 0.869302
H 3.973339 -4.104695 1.499694
H -0.82233 -1.941286 -1.230824
C 0.508998 3.198392 -0.687945
H 2.231873 2.671424 -1.611207
O -0.357602 2.922226 0.152709
C 0.514452 4.534533 -1.381219
H 1.276624 4.613396 -2.15714
H 0.684753 5.310099 -0.630683
H -0.469955 4.704644 -1.820984
Na -1.785378 2.19079 1.559314

Reactant state of 3rd c2 reaction of GlcNAcβ(1→2)Manβ
52

C 1.111275 -0.803899 3.219983
C 0.994703 -0.971026 1.713191
C 2.371738 -0.917907 1.033202
O 3.228228 -1.949687 1.463158
C 2.228329 -1.098571 -0.477905
C 3.495363 -0.668454 -1.236338
C 3.267481 -0.701535 -2.736244
O 2.296788 0.314746 -3.033925
O 3.83642 0.686098 -0.899618
O 1.921718 -2.434608 -0.794873
O 2.057774 -1.203294 3.857256
H 4.318474 -1.34548 -0.980932
H 0.27936 -0.264407 3.713724
H 2.796182 0.074222 1.247206
H 1.384173 -0.491236 -0.823592
H 4.540078 0.705174 -0.242106
H 4.2012 -0.484552 -3.264023
H 2.889812 -1.688095 -3.018156
H 2.456327 -3.001016 -0.216471
H 3.269683 -1.946231 2.432732
H 1.95568 0.18697 -3.925589
C -1.112085 -0.049853 1.016807
C -1.634821 1.14118 0.209201
N -1.363636 2.418245 0.836292
C -3.130018 0.947756 0.002574
O -3.614012 2.019047 -0.764504
C -3.404391 -0.393999 -0.669303
C -2.720418 -1.530759 0.104208
C -2.734094 -2.85108 -0.653748
O -4.081755 -3.120605 -1.002859
O -1.339366 -1.227182 0.285921
O -4.805985 -0.503325 -0.690344
O 0.260071 0.121655 1.181615
H -3.22133 -1.648269 1.078658
H -1.621619 -0.121195 1.997493
H -1.119702 1.125444 -0.757397
H -3.633376 0.92393 0.987174
H -2.988947 -0.36209 -1.690993
H -2.102877 -2.744879 -1.545681
H -2.317115 -3.639959 -0.017997
H -5.046166 -1.379062 -1.028558
H -4.535311 1.823541 -0.989457
H -4.154098 -3.981408 -1.428773
H 0.510264 -1.929068 1.47888
C -0.325961 3.200283 0.481679
H -2.105592 2.843359 1.377009
O 0.540394 2.821392 -0.317738
C -0.263318 4.573071 1.097183
H -1.046735 4.750032 1.834824
H -0.349555 5.312422 0.297444
H 0.71281 4.701479 1.56868
Na 2.109903 2.018866 -1.53071

Transition state of 1st m1_4 reaction of GlcNAcβ(1→2)Manβ
52

C 1.711907 0.214742 1.926701
C 1.042765 -0.70481 0.868104
C 2.114277 -0.859787 -0.243519
O 1.567485 -0.825123 -1.539839
C 3.008794 0.383569 -0.000884
C 4.504292 0.323081 -0.376783
C 5.373066 -0.29371 0.705937
O 4.874069 -1.610937 0.913235
O 4.631688 -0.371082 -1.593587
O 2.408173 1.534061 -0.215893
O 1.393461 1.46514 1.906979
H 4.817771 1.355518 -0.553929
H 2.031579 -0.202829 2.885805
H 2.698855 -1.769888 -0.071135
H 2.982336 0.19595 1.291885
H 4.672029 -1.31984 -1.406838
H 6.409884 -0.312664 0.352947
H 5.317702 0.300878 1.626998
H 1.710473 1.753325 0.859481
H 0.760591 -1.376998 -1.558214
H 5.505972 -2.133531 1.41913
C -1.282065 -0.164692 1.012603
C -2.365665 0.527712 0.168439
N -2.46196 1.944951 0.44638
C -3.695613 -0.182813 0.419243
O -4.801654 0.494194 -0.131442
C -3.570753 -1.592117 -0.149688
C -2.334252 -2.306103 0.42964
C -1.454699 -2.963433 -0.6229
O -1.008732 -2.032735 -1.619162
O -1.59852 -1.48212 1.342069
O -4.690245 -2.381776 0.15899
O -0.073563 -0.075544 0.278865
H -2.715918 -3.109975 1.066374
H -1.161189 0.351512 1.973658
H -2.090591 0.413383 -0.889151
H -3.901931 -0.250703 1.494483
H -3.461362 -1.490284 -1.24429
H -0.58286 -3.412498 -0.133602
H -2.038518 -3.7641 -1.088903
H -5.491417 -1.900781 -0.091209
H -4.646896 0.682519 -1.067468
H -1.170388 -2.40574 -2.493276
H 0.748986 -1.661111 1.31206
C -1.80014 2.877454 -0.274872
H -3.173405 2.252777 1.09684
O -1.007486 2.55948 -1.167626
C -2.066457 4.32035 0.05678
H -2.843271 4.453358 0.810521
H -2.352173 4.838404 -0.860681
H -1.136734 4.768422 0.414982
Na 0.697641 1.315012 -1.676785

Reactant state of 1st m1_4 reaction of GlcNAcβ(1→2)Manβ
52

C -1.102606 -0.767841 -2.669035
C -0.818266 -1.076747 -1.205642
C -2.115358 -1.018766 -0.352495
O -1.735555 -0.945445 1.012786
C -2.998867 0.189204 -0.719885
C -4.189873 0.447747 0.216586
C -5.22632 -0.665942 0.240164
O -4.639885 -1.76196 0.927431
O -3.750173 0.732049 1.538536
O -2.263579 1.405836 -0.71319
O -0.935305 0.339755 -3.123762
H -4.665779 1.360341 -0.156424
H -1.489025 -1.593686 -3.294783
H -2.702065 -1.929397 -0.528287
H -3.410603 0.006783 -1.724314
H -3.650796 -0.131301 1.973863
H -6.112858 -0.300917 0.769714
H -5.512486 -0.941216 -0.783473
H -1.604417 1.401856 -1.426604
H -0.871776 -1.385302 1.131145
H -5.321814 -2.379337 1.213189
C 1.407945 -0.175086 -1.072538
C 2.201602 0.65729 -0.056043
N 2.125221 2.07585 -0.33195
C 3.641843 0.149445 -0.044955
O 4.528126 0.984889 0.663752
C 3.609809 -1.251844 0.5547
C 2.599037 -2.150889 -0.183502
C 1.663339 -2.898275 0.750146
O 0.877765 -1.987305 1.529602
O 1.896965 -1.472887 -1.232793
O 4.860735 -1.885772 0.477407
O 0.064537 -0.144718 -0.628213
H 3.191302 -2.903284 -0.713165
H 1.46482 0.276848 -2.07131
H 1.746918 0.492738 0.929761
H 4.045206 0.094369 -1.063916
H 3.299962 -1.141202 1.609451
H 1.000924 -3.546126 0.164733
H 2.276375 -3.529076 1.402987
H 5.533685 -1.292725 0.839666
H 4.175919 1.171709 1.544973
H 0.822037 -2.316376 2.434092
H -0.398717 -2.090026 -1.141026
C 1.26148 2.899766 0.303946
H 2.847883 2.488388 -0.907618
O 0.426482 2.470829 1.106678
C 1.360835 4.368682 -0.011544
H 2.179732 4.610395 -0.690026
H 1.488841 4.914685 0.925198
H 0.417235 4.691728 -0.457302
Na -1.457777 1.392413 1.397983

Transition state of 2nd m1_4 reaction of GlcNAcβ(1→2)Manβ
52

C 1.557582 0.377016 2.234509
C 0.902099 -0.656605 1.29741
C 2.042644 -1.12768 0.336273
O 1.694768 -0.968313 -1.026265
C 3.252482 -0.224678 0.714248
C 4.084037 0.548449 -0.30632
C 4.924307 -0.404106 -1.191904
O 4.229812 -0.723815 -2.380216
O 3.286063 1.383241 -1.13489
O 3.94266 -0.679798 1.711416
O 2.274846 -0.058921 3.208784
H 4.760303 1.165461 0.294592
H 1.023121 1.326877 2.382
H 2.334294 -2.16373 0.542414
H 2.469525 0.768276 1.264019
H 3.487627 1.107396 -2.047218
H 5.831659 0.11448 -1.508452
H 5.21501 -1.289784 -0.616947
H 3.224881 -0.544821 2.668755
H 0.837397 -1.418951 -1.19154
H 3.447626 -1.252581 -2.164915
C -1.411752 -0.092674 1.041747
C -2.34648 0.631569 0.056349
N -2.477627 2.046302 0.342639
C -3.714107 -0.053307 0.111293
O -4.713286 0.658123 -0.581357
C -3.549741 -1.464417 -0.440002
C -2.427791 -2.209506 0.308091
C -1.429354 -2.896909 -0.608199
O -0.834163 -1.978979 -1.53969
O -1.79787 -1.401046 1.309452
O -4.720098 -2.224594 -0.29254
O -0.094829 -0.032407 0.503305
H -2.91732 -2.995822 0.890355
H -1.426758 0.412267 2.016209
H -1.925107 0.522329 -0.953344
H -4.068981 -0.116187 1.147391
H -3.284496 -1.367257 -1.508223
H -0.639451 -3.35473 -0.002422
H -1.956787 -3.690029 -1.147537
H -5.469519 -1.727511 -0.648942
H -4.450733 0.797427 -1.502033
H -0.906002 -2.338462 -2.431493
H 0.473838 -1.478236 1.878853
C -1.668061 2.980555 -0.206122
H -3.341482 2.353538 0.772853
O -0.682294 2.659682 -0.878501
C -2.016913 4.423536 0.034776
H -2.893142 4.555032 0.670326
H -2.193584 4.899812 -0.932339
H -1.157913 4.918072 0.492671
Na 0.999164 1.310925 -1.113852

Reactant state of 2nd m1_4 reaction of GlcNAcβ(1→2)Manβ
52

C 1.136906 -0.333627 2.772367
C 0.884284 -0.826036 1.355373
C 2.162993 -0.924989 0.478365
O 1.754152 -0.974729 -0.885984
C 3.176453 0.233421 0.692937
C 4.002987 0.682745 -0.527601
C 4.783443 -0.446962 -1.2361
O 4.112309 -0.889017 -2.402951
O 3.194871 1.380966 -1.470427
O 4.163093 -0.149791 1.615442
O 2.105158 -0.654877 3.4165
H 4.726892 1.384927 -0.100758
H 0.367827 0.335753 3.202575
H 2.685816 -1.851068 0.747796
H 2.630635 1.129993 1.049158
H 3.384088 0.967732 -2.331539
H 5.744629 -0.06095 -1.580004
H 4.979826 -1.261416 -0.530356
H 3.76863 -0.415096 2.458267
H 0.918587 -1.476593 -0.977446
H 3.308727 -1.352678 -2.129829
C -1.415588 -0.142954 1.079375
C -2.285632 0.65598 0.099003
N -2.37678 2.056442 0.454782
C -3.672827 0.010116 0.066694
O -4.63031 0.793717 -0.60476
C -3.520386 -1.358648 -0.582467
C -2.468365 -2.202899 0.158142
C -1.509031 -2.917171 -0.77207
O -0.752843 -1.960287 -1.528045
O -1.770957 -1.483569 1.185883
O -4.716119 -2.09186 -0.55676
O -0.048771 0.025525 0.690898
H -3.024613 -2.967838 0.708263
H -1.545378 0.266753 2.089071
H -1.832759 0.58649 -0.899838
H -4.063965 -0.11647 1.084352
H -3.193188 -1.189602 -1.624345
H -0.825878 -3.545293 -0.189858
H -2.094072 -3.559106 -1.438888
H -5.430508 -1.548506 -0.917642
H -4.324876 1.008555 -1.497336
H -0.666851 -2.27034 -2.437528
H 0.446272 -1.8303 1.440943
C -1.579744 3.000789 -0.100974
H -3.215406 2.361191 0.933592
O -0.638115 2.690941 -0.836098
C -1.890985 4.437755 0.213838
H -2.733719 4.557278 0.895429
H -2.103801 4.956053 -0.723881
H -1.002124 4.898702 0.649281
Na 0.975079 1.263224 -1.112166

Transition state of 3rd m1_4 reaction of GlcNAcβ(1→2)Manβ
52

C -1.627582 -0.098909 -1.908189
C -0.765702 -1.245896 -1.37197
C -1.702477 -2.009929 -0.395874
O -1.028185 -2.603785 0.654386
C -2.757183 -0.897678 -0.034982
C -3.025673 -0.287202 1.360516
C -2.005718 -0.479966 2.479171
O -0.65862 -0.305662 2.130274
O -3.385749 1.087868 1.144477
O -3.874254 -0.996389 -0.742058
O -2.684984 -0.310104 -2.627585
H -3.947599 -0.768493 1.712478
H -1.131904 0.870491 -2.042913
H -2.267406 -2.788085 -0.918758
H -2.109119 0.099137 -0.605217
H -2.572293 1.613234 1.00183
H -2.307811 0.195562 3.292357
H -2.099543 -1.508465 2.838306
H -3.415729 -0.814266 -1.928376
H -0.375748 -1.96116 1.003416
H -0.535907 0.491922 1.586492
C 1.333174 -0.087921 -1.113987
C 1.872694 0.936469 -0.095881
N 1.341226 2.280209 -0.307818
C 3.385664 0.975212 -0.261974
O 3.893691 2.050486 0.489979
C 3.953239 -0.360776 0.193707
C 3.192682 -1.528522 -0.484532
C 2.550982 -2.481651 0.517585
O 1.799959 -1.740604 1.466266
O 2.272715 -1.053082 -1.476943
O 5.32635 -0.317805 -0.154835
O 0.192791 -0.655844 -0.532535
H 3.917244 -2.098417 -1.072837
H 1.082201 0.419821 -2.056101
H 1.61776 0.590742 0.913931
H 3.635113 1.1028 -1.327918
H 3.831123 -0.41704 1.283051
H 1.908996 -3.190658 -0.020346
H 3.357946 -3.040745 1.010209
H 5.840544 -0.901904 0.413153
H 4.858554 2.024426 0.425646
H 1.67539 -2.250662 2.274159
H -0.318722 -1.8671 -2.157504
C 0.124676 2.711164 0.065969
H 2.042684 2.989477 -0.489866
O -0.759102 1.945465 0.49236
C -0.142164 4.188614 -0.066184
H -0.32623 4.592806 0.932266
H -1.048838 4.332067 -0.657341
H 0.679335 4.735988 -0.529027
Na -5.132291 0.798607 -0.252445

Reactant state of 3rd m1_4 reaction of GlcNAcβ(1→2)Manβ
52

C -1.081208 -1.403448 -2.629318
C -0.617969 -1.689605 -1.211164
C -1.738212 -1.920349 -0.178426
O -1.144537 -2.404815 0.992132
C -2.544303 -0.599552 -0.009414
C -3.006206 -0.149423 1.384701
C -2.04817 -0.25534 2.570954
O -0.688762 -0.036695 2.272548
O -3.457192 1.218254 1.216596
O -3.76151 -0.655534 -0.785961
O -2.245699 -1.306386 -2.956695
H -3.909559 -0.713453 1.652454
H -0.289594 -1.26996 -3.38883
H -2.423548 -2.697555 -0.534549
H -1.930912 0.22367 -0.390347
H -2.672806 1.777049 1.058689
H -2.410154 0.451054 3.330378
H -2.11305 -1.265148 2.979501
H -3.519239 -0.974696 -1.674767
H -0.459291 -1.762471 1.266781
H -0.572098 0.748105 1.708843
C 1.35754 -0.223878 -1.15971
C 1.801203 0.947283 -0.271105
N 1.218635 2.209601 -0.699202
C 3.318937 1.004826 -0.361262
O 3.768637 2.207891 0.208698
C 3.87176 -0.210004 0.368996
C 3.181845 -1.515184 -0.102312
C 2.546755 -2.281862 1.046495
O 1.602478 -1.426318 1.663341
O 2.247331 -1.30042 -1.171543
O 5.264671 -0.204288 0.109835
O 0.076122 -0.561549 -0.700916
H 3.950479 -2.144802 -0.559922
H 1.307709 0.112761 -2.205767
H 1.49042 0.735924 0.758449
H 3.631776 0.940483 -1.417494
H 3.67799 -0.05587 1.439073
H 2.060633 -3.187232 0.661508
H 3.339402 -2.579219 1.746629
H 5.745296 -0.655609 0.812401
H 4.735809 2.188707 0.210895
H 1.423598 -1.683879 2.574977
H 0.040043 -2.570376 -1.225082
C 0.049391 2.714933 -0.255463
H 1.859939 2.863942 -1.130722
O -0.741574 2.079371 0.457457
C -0.271291 4.126839 -0.679445
H -0.32781 4.747997 0.217593
H -1.253539 4.136198 -1.155838
H 0.463226 4.551478 -1.364501
Na -5.150482 0.951653 -0.181914

Transition state of 1st m1_5 reaction of GlcNAcβ(1→2)Manβ
52

C 1.537053 -0.505429 -2.194239
C 1.090676 0.908121 -1.783565
C 2.204396 1.576394 -0.974769
O 1.914403 2.900471 -0.642043
C 2.458154 0.747293 0.306072
C 2.614329 -0.749067 -0.016814
C 3.999679 -1.3573 0.212556
O 4.220075 -1.422089 1.600309
O 1.596217 -1.536421 0.274554
O 3.636571 1.209285 0.930865
O 0.765627 -1.512255 -1.919173
H 2.589187 -0.670103 -1.33331
H 2.136073 -0.573623 -3.110822
H 3.134456 1.5799 -1.560472
H 1.592432 0.853558 0.971883
H 0.993943 -1.7051 -0.804792
H 3.99236 -2.379806 -0.17097
H 4.770724 -0.773709 -0.302304
H 3.60274 2.176893 0.956228
H 0.971579 2.970734 -0.395947
H 4.442842 -0.530475 1.9036
C -1.27061 0.42301 -1.256123
C -1.601813 -0.648281 -0.202379
N -2.427434 -1.720495 -0.713589
C -2.120284 -0.032188 1.100657
O -1.476915 -0.702823 2.1867
C -1.837111 1.458712 1.161275
C -2.482158 2.13988 -0.054257
C -2.121707 3.611219 -0.161851
O -0.720038 3.783723 0.032242
O -2.188022 1.468426 -1.290778
O -2.420401 1.919342 2.365361
O 0.009095 0.89923 -0.883071
H -3.570451 2.082863 0.078128
H -1.256146 -0.00765 -2.260796
H -0.64389 -1.097269 0.024977
H -3.207046 -0.176767 1.179947
H -0.756193 1.626564 1.167126
H -2.429508 3.968465 -1.150095
H -2.678151 4.164954 0.604691
H -1.971258 2.718098 2.666119
H -1.773225 -0.267268 3.000215
H -0.501108 4.717103 -0.078031
H 0.862949 1.496621 -2.684249
C -2.121424 -3.012422 -0.436845
H -3.224476 -1.511954 -1.299875
O -1.173812 -3.303073 0.301168
C -2.972453 -4.076109 -1.072191
H -3.349997 -4.736105 -0.289146
H -2.335379 -4.67203 -1.729943
H -3.807402 -3.672911 -1.646598
Na 0.167656 -2.370094 1.804123

Reactant state of 1st m1_5 reaction of GlcNAcβ(1→2)Manβ
52

C 1.431402 -0.439041 -2.877191
C 1.721852 0.401183 -1.648573
C 2.772428 -0.284852 -0.7427
O 3.477577 0.687722 0.000948
C 2.16821 -1.288126 0.27404
C 1.451979 -2.540104 -0.277425
C 1.424134 -3.645181 0.772064
O 0.726891 -3.174387 1.931255
O 0.076856 -2.279787 -0.54799
O 3.230037 -1.75417 1.086611
O 0.408649 -1.069506 -3.031559
H 1.956742 -2.920998 -1.177499
H 2.227127 -0.453749 -3.644922
H 3.520712 -0.808788 -1.351567
H 1.436389 -0.744152 0.887687
H -0.0375 -1.897042 -1.433125
H 0.849009 -4.485229 0.376462
H 2.428786 -3.97828 1.031591
H 3.801621 -0.99627 1.281107
H 2.866055 1.402128 0.252974
H 1.375143 -2.757688 2.514245
C -0.585211 1.207028 -1.20384
C -1.582562 0.52931 -0.254376
N -2.936844 0.45416 -0.753922
C -1.480316 1.106071 1.166737
O -1.376777 0.014198 2.078975
C -0.298118 2.048229 1.342292
C -0.325445 3.124931 0.245115
C 0.919597 3.990379 0.237956
O 2.069695 3.156009 0.120015
O -0.519662 2.59189 -1.074485
O -0.4704 2.637377 2.619252
O 0.614784 0.577717 -0.812729
H -1.180352 3.780259 0.459165
H -0.82796 1.011615 -2.252467
H -1.232689 -0.497018 -0.215702
H -2.3907 1.681162 1.388495
H 0.632021 1.475359 1.305232
H 0.855574 4.678315 -0.611264
H 0.95885 4.575538 1.165432
H 0.381058 2.898805 2.987744
H -1.322409 0.388596 2.9704
H 2.846161 3.707351 -0.035329
H 2.142671 1.360658 -1.989396
C -3.665038 -0.674823 -0.536376
H -3.367743 1.252051 -1.200999
O -3.187305 -1.635159 0.072648
C -5.068026 -0.703995 -1.077131
H -5.756392 -0.878592 -0.24768
H -5.153978 -1.550364 -1.761839
H -5.348071 0.213075 -1.597076
Na -1.346754 -2.23974 1.282608

Transition state of 2nd m1_5 reaction of GlcNAcβ(1→2)Manβ
52

C 1.536939 -0.506824 -2.193843
C 1.091466 0.907185 -1.783618
C 2.205707 1.574869 -0.975101
O 1.916697 2.899226 -0.642654
C 2.459001 0.745931 0.305965
C 2.61362 -0.750701 -0.016272
C 3.998385 -1.360318 0.212852
O 4.219021 -1.424893 1.60058
O 1.594726 -1.536845 0.27519
O 3.638074 1.206911 0.930269
O 0.764423 -1.512951 -1.918708
H 2.588369 -0.672114 -1.333419
H 2.135636 -0.575622 -3.110593
H 3.135734 1.577574 -1.560857
H 1.593571 0.853322 0.971973
H 0.992526 -1.705467 -0.804088
H 3.989888 -2.382935 -0.170353
H 4.769904 -0.777688 -0.30238
H 3.605184 2.174556 0.955462
H 0.974012 2.970076 -0.396191
H 4.442799 -0.533423 1.903551
C -1.270119 0.423945 -1.256112
C -1.60225 -0.647155 -0.202439
N -2.428521 -1.718751 -0.713906
C -2.120558 -0.030747 1.100504
O -1.477965 -0.701965 2.186649
C -1.836194 1.459925 1.161265
C -2.480533 2.141682 -0.05431
C -2.11879 3.612718 -0.161774
O -0.717052 3.784069 0.032852
O -2.18681 1.470014 -1.290801
O -2.419289 1.920977 2.365289
O 0.009917 0.899262 -0.883016
H -3.568889 2.085583 0.077942
H -1.255892 -0.006705 -2.26079
H -0.644697 -1.096777 0.025157
H -3.207454 -0.174458 1.179535
H -0.755146 1.626921 1.167275
H -2.425897 3.970197 -1.150149
H -2.675059 4.16694 0.604539
H -1.969381 2.71921 2.666288
H -1.774222 -0.266315 3.000132
H -0.497347 4.717275 -0.077344
H 0.864025 1.495615 -2.684411
C -2.123575 -3.010924 -0.437173
H -3.225131 -1.509575 -1.300553
O -1.176463 -3.302332 0.301195
C -2.975256 -4.07389 -1.07288
H -3.35295 -4.734046 -0.290048
H -2.338598 -4.6698 -1.73104
H -3.810154 -3.670063 -1.646922
Na 0.165412 -2.370633 1.804316

Reactant state of 2nd m1_5 reaction of GlcNAcβ(1→2)Manβ
52

C 1.431828 -0.437445 -2.876843
C 1.721868 0.402815 -1.648137
C 2.772583 -0.282761 -0.742158
O 3.476638 0.690171 0.002252
C 2.168893 -1.286844 0.274044
C 1.453533 -2.538938 -0.278222
C 1.426311 -3.644844 0.770378
O 0.728971 -3.175335 1.929889
O 0.078247 -2.278815 -0.54856
O 3.230886 -1.752584 1.086532
O 0.409114 -1.067879 -3.031706
H 1.958417 -2.918611 -1.178724
H 2.227863 -0.452125 -3.644246
H 3.521624 -0.805779 -1.350875
H 1.43666 -0.743526 0.887789
H -0.03622 -1.897297 -1.434186
H 0.85154 -4.484827 0.374121
H 2.43116 -3.977741 1.029461
H 3.801599 -0.994272 1.281994
H 2.864408 1.404026 0.25409
H 1.376866 -2.757276 2.512351
C -0.585842 1.207002 -1.203424
C -1.582519 0.528008 -0.254211
N -2.936894 0.452715 -0.753505
C -1.480138 1.103975 1.167218
O -1.374574 0.011681 2.078723
C -0.299161 2.047666 1.342973
C -0.327639 3.124667 0.246058
C 0.916963 3.990761 0.238564
O 2.067294 3.157063 0.118047
O -0.522288 2.592004 -1.073617
O -0.472531 2.636102 2.620143
O 0.614763 0.579189 -0.812219
H -1.182802 3.779437 0.460741
H -0.828202 1.011671 -2.252147
H -1.232056 -0.498135 -0.216337
H -2.391135 1.677739 1.389871
H 0.63174 1.476017 1.306021
H 0.851706 4.679885 -0.609584
H 0.957074 4.574701 1.166798
H 0.378283 2.900071 2.988305
H -1.320694 0.385562 2.970388
H 2.843248 3.708936 -0.037969
H 2.142337 1.362412 -1.988985
C -3.664925 -0.676325 -0.535853
H -3.367132 1.24983 -1.202591
O -3.187465 -1.636099 0.074275
C -5.06748 -0.706176 -1.077683
H -5.756518 -0.879883 -0.248598
H -5.152885 -1.553302 -1.761524
H -5.347162 0.210318 -1.598834
Na -1.345414 -2.241835 1.280992

Transition state of 3rd m1_5 reaction of GlcNAcβ(1→2)Manβ
52

C 1.536918 -0.506842 -2.194073
C 1.091664 0.907147 -1.78362
C 2.205983 1.574587 -0.974994
O 1.917161 2.898968 -0.642462
C 2.459105 0.745487 0.305988
C 2.613488 -0.751138 -0.016469
C 3.998088 -1.361034 0.212968
O 4.21837 -1.425754 1.600747
O 1.594409 -1.537114 0.274924
O 3.638226 1.20623 0.93038
O 0.764385 -1.512931 -1.919077
H 2.588495 -0.672415 -1.333353
H 2.135797 -0.575618 -3.1107
H 3.136025 1.577201 -1.560726
H 1.593676 0.852924 0.971989
H 0.992149 -1.70547 -0.804541
H 3.989497 -2.383621 -0.170318
H 4.76985 -0.778515 -0.302022
H 3.605493 2.173876 0.955661
H 0.974507 2.969904 -0.395922
H 4.442381 -0.534371 1.903801
C -1.270016 0.42416 -1.256192
C -1.602344 -0.646921 -0.202555
N -2.428811 -1.718373 -0.714005
C -2.120502 -0.030447 1.100431
O -1.47804 -0.701787 2.186567
C -1.835846 1.46017 1.161214
C -2.48011 2.142062 -0.05432
C -2.118167 3.613051 -0.161782
O -0.71647 3.784229 0.033275
O -2.186497 1.470411 -1.290845
O -2.418802 1.921286 2.365277
O 0.010109 0.899214 -0.883038
H -3.568467 2.086098 0.077977
H -1.255866 -0.006447 -2.260889
H -0.644874 -1.096786 0.024974
H -3.207429 -0.173941 1.179465
H -0.754768 1.626951 1.167199
H -2.424896 3.970446 -1.150307
H -2.674628 4.167438 0.604273
H -1.968963 2.719638 2.666075
H -1.774073 -0.26595 3.000034
H -0.496619 4.717418 -0.076782
H 0.864317 1.495722 -2.684344
C -2.12421 -3.01059 -0.437072
H -3.225786 -1.509064 -1.300108
O -1.176966 -3.302178 0.301046
C -2.976338 -4.073456 -1.072342
H -3.81134 -3.669465 -1.646101
H -3.353843 -4.733497 -0.289317
H -2.340072 -4.669578 -1.730701
Na 0.165251 -2.370571 1.804117

Reactant state of 3rd m1_5 reaction of GlcNAcβ(1→2)Manβ
52

C 1.431445 -0.438983 -2.87718
C 1.721862 0.40122 -1.648542
C 2.772431 -0.28482 -0.742665
O 3.477566 0.687739 0.001013
C 2.168208 -1.288118 0.274049
C 1.451999 -2.540099 -0.277448
C 1.424143 -3.64519 0.772027
O 0.726905 -3.174402 1.931224
O 0.076875 -2.279793 -0.548026
O 3.230032 -1.754155 1.086631
O 0.408721 -1.069493 -3.031551
H 1.956775 -2.920982 -1.17752
H 2.227162 -0.453634 -3.644919
H 3.520725 -0.80874 -1.351535
H 1.436369 -0.744165 0.887691
H -0.037457 -1.896954 -1.433129
H 0.849009 -4.485228 0.376416
H 2.428791 -3.978303 1.031553
H 3.801612 -0.996253 1.28113
H 2.866039 1.402137 0.253049
H 1.375161 -2.757711 2.514214
C -0.585221 1.207009 -1.203834
C -1.582574 0.529295 -0.254366
N -2.936858 0.454153 -0.753904
C -1.480314 1.106048 1.16675
O -1.376748 0.014172 2.078978
C -0.298125 2.048222 1.342293
C -0.325474 3.12492 0.245113
C 0.91955 3.990393 0.237953
O 2.06966 3.156045 0.119976
O -0.519668 2.59187 -1.074487
O -0.470403 2.637376 2.619251
O 0.61477 0.577698 -0.81272
H -1.180397 3.780229 0.459159
H -0.827969 1.01159 -2.252461
H -1.232711 -0.497037 -0.215697
H -2.390703 1.681126 1.388523
H 0.632019 1.475363 1.305225
H 0.855502 4.678342 -0.611253
H 0.958805 4.575535 1.16544
H 0.381059 2.898786 2.987747
H -1.322377 0.388562 2.970406
H 2.846106 3.707399 -0.035422
H 2.142659 1.360713 -1.989335
C -3.665051 -0.674831 -0.536364
H -3.367762 1.25205 -1.200968
O -3.187309 -1.635174 0.072643
C -5.068038 -0.704007 -1.077125
H -5.348115 0.213097 -1.596992
H -5.756398 -0.878704 -0.247691
H -5.153955 -1.550318 -1.761909
Na -1.346745 -2.239762 1.282561

Transition state of 1st m1_6 reaction of GlcNAcβ(1→2)Manβ
52

C -2.044307 1.473267 -1.494177
C -1.328334 0.107596 -1.466174
C -2.232293 -1.090846 -1.168786
O -1.605497 -2.297464 -1.560463
C -2.645158 -1.212797 0.312253
C -3.737381 -0.225049 0.739031
C -3.381179 1.238919 0.550736
O -2.30818 1.728186 1.129708
O -4.898986 -0.428033 -0.021726
O -3.211089 -2.484764 0.533303
O -1.454333 2.465251 -0.923204
H -3.933562 -0.381822 1.811803
H -2.633858 1.678489 -2.395701
H -3.143306 -1.001115 -1.774249
H -1.752875 -1.090806 0.947319
H -5.156618 -1.356487 0.071484
H -4.268076 1.888354 0.52201
H -3.150699 1.230563 -0.722567
H -2.784149 -3.11033 -0.07222
H -0.642568 -2.231674 -1.440527
H -1.691444 2.297364 0.205392
C 1.015844 -0.194826 -1.047966
C 2.120291 0.471225 -0.224014
N 2.236285 1.870839 -0.552034
C 3.392176 -0.333368 -0.45986
O 4.482777 0.387722 0.043607
C 3.208996 -1.665613 0.26922
C 1.809577 -2.269796 -0.015049
C 0.907201 -2.428597 1.20939
O 0.773223 -1.232713 1.996101
O 1.149464 -1.595271 -1.097837
O 4.259918 -2.49475 -0.177594
O -0.245427 0.17018 -0.538483
H 1.968065 -3.277257 -0.412846
H 1.092408 0.142487 -2.089348
H 1.886077 0.408196 0.841208
H 3.522671 -0.548029 -1.532628
H 3.312611 -1.451206 1.343071
H -0.091669 -2.735066 0.880029
H 1.315914 -3.228879 1.834617
H 4.500137 -3.149637 0.487399
H 5.287651 -0.123685 -0.116039
H 1.104145 -1.404953 2.884898
H -0.924257 -0.04045 -2.478566
C 1.62175 2.815185 0.207252
H 2.971452 2.159232 -1.184112
O 0.885721 2.504522 1.147466
C 1.847486 4.251163 -0.170091
H 2.053064 4.822692 0.735841
H 0.918008 4.629785 -0.60428
H 2.658404 4.385056 -0.887098
Na -0.365478 0.817419 1.755614

Reactant state of 1st m1_6 reaction of GlcNAcβ(1→2)Manβ
52

C -1.818159 1.982251 -1.352614
C -1.190887 0.617737 -1.555068
C -2.210368 -0.542319 -1.448082
O -1.752621 -1.639488 -2.215315
C -2.510744 -0.985043 0.015722
C -3.785713 -0.399453 0.638232
C -3.774488 1.08135 0.978983
O -2.652277 1.379027 1.820844
O -4.891261 -0.578009 -0.215828
O -2.71167 -2.389723 0.05689
O -1.476858 2.738405 -0.473844
H -3.920543 -0.954962 1.577349
H -2.615127 2.254111 -2.068685
H -3.146963 -0.229958 -1.925462
H -1.656662 -0.737331 0.658372
H -5.075111 -1.523929 -0.284107
H -4.710023 1.308706 1.496098
H -3.74775 1.697351 0.073907
H -2.576206 -2.742241 -0.836372
H -0.867828 -1.897876 -1.900868
H -2.938601 2.016741 2.484178
C 0.999049 -0.257203 -1.045489
C 2.221355 0.190553 -0.242882
N 2.72058 1.472479 -0.683935
C 3.251992 -0.925223 -0.356457
O 4.493256 -0.444464 0.085336
C 2.742168 -2.066992 0.513822
C 1.243419 -2.354943 0.240902
C 0.345881 -2.210869 1.461158
O 0.377601 -0.873892 1.985139
O 0.742469 -1.633973 -0.902041
O 3.56858 -3.176023 0.225758
O -0.104397 0.519958 -0.653867
H 1.173959 -3.400328 -0.075209
H 1.175348 -0.100781 -2.119276
H 1.937489 0.294748 0.808941
H 3.321208 -1.285215 -1.396
H 2.869193 -1.747728 1.558252
H -0.678475 -2.492374 1.1942
H 0.714151 -2.913572 2.218907
H 3.622003 -3.776366 0.977735
H 5.131769 -1.169307 0.039906
H 0.197732 -0.921623 2.931286
H -0.826869 0.599759 -2.59544
C 2.423255 2.60101 0.015241
H 3.566653 1.475052 -1.240033
O 1.568791 2.601774 0.903513
C 3.18559 3.847007 -0.342989
H 3.819784 4.114476 0.505941
H 2.47383 4.659814 -0.495524
H 3.805314 3.729096 -1.232621
Na -0.374487 1.630952 1.306957

Transition state of 2nd m1_6 reaction of GlcNAcβ(1→2)Manβ
52

C 0.985052 -1.718722 -1.851773
C 1.427647 -0.265941 -1.607616
C 2.770579 -0.105967 -0.887792
O 3.176578 1.241388 -0.983971
C 2.71809 -0.482847 0.613389
C 2.676437 -1.982513 0.92062
C 1.510913 -2.750349 0.322405
O 0.264327 -2.458102 0.635062
O 3.819049 -2.61809 0.405543
O 3.91879 -0.052941 1.221694
O -0.254877 -2.015254 -1.612029
H 2.622735 -2.094444 2.015801
H 1.471514 -2.234128 -2.689379
H 3.538476 -0.709899 -1.385302
H 1.851642 0.009551 1.075563
H 4.589925 -2.115915 0.708644
H 1.74531 -3.817401 0.196431
H 1.679721 -2.411945 -0.909195
H 4.175159 0.783848 0.805317
H 2.421798 1.819276 -0.758205
H -0.257198 -2.254323 -0.479714
C -0.770441 0.772511 -1.285421
C -1.742483 0.222827 -0.228729
N -3.018869 -0.174637 -0.782463
C -1.852001 1.146664 0.989672
O -1.758989 0.338561 2.165299
C -0.761255 2.20359 0.987159
C -0.847483 2.997152 -0.323875
C 0.269707 4.013608 -0.468993
O 1.514885 3.396995 -0.152463
O -0.878341 2.14644 -1.481708
O -1.021865 3.035472 2.101499
O 0.511827 0.432313 -0.794457
H -1.794348 3.553662 -0.312661
H -0.954608 0.311407 -2.259232
H -1.266304 -0.690151 0.105315
H -2.823383 1.660708 0.983044
H 0.218196 1.726139 1.080477
H 0.264911 4.388036 -1.498063
H 0.076246 4.849375 0.214651
H -0.205347 3.45552 2.396951
H -1.763446 0.943787 2.922389
H 2.221556 4.047314 -0.24912
H 1.512089 0.21618 -2.594915
C -3.589741 -1.348943 -0.418275
H -3.473324 0.407587 -1.473264
O -3.070755 -2.075153 0.43705
C -4.870357 -1.73821 -1.101655
H -5.621886 -1.952734 -0.339793
H -4.690752 -2.660156 -1.659698
H -5.245025 -0.971954 -1.781383
Na -1.497689 -1.980643 1.988705

Reactant state of 2nd m1_6 reaction of GlcNAcβ(1→2)Manβ
52

C 0.861414 -1.387036 -2.155218
C 1.390107 -0.038879 -1.732535
C 2.679797 -0.069103 -0.884437
O 3.164091 1.261652 -0.867141
C 2.48898 -0.541349 0.587132
C 2.629804 -2.041341 0.890008
C 1.573256 -2.997326 0.362297
O 0.264954 -2.55453 0.727166
O 3.850403 -2.534852 0.392703
O 3.535029 0.016605 1.364425
O -0.257009 -1.788947 -1.919923
H 2.613193 -2.094439 1.990462
H 1.572982 -1.989671 -2.753054
H 3.444936 -0.682781 -1.37208
H 1.514588 -0.187136 0.954249
H 4.559698 -2.014405 0.795299
H 1.766899 -3.988491 0.783204
H 1.661251 -3.089612 -0.724161
H 3.816502 0.83783 0.93329
H 2.416381 1.866078 -0.690805
H -0.359684 -2.773576 0.016375
C -0.846132 0.928017 -1.337454
C -1.660452 0.212801 -0.237452
N -2.87403 -0.390714 -0.751311
C -1.900264 1.13719 0.956674
O -1.862461 0.355375 2.152341
C -0.846203 2.231514 1.018552
C -0.960133 3.086553 -0.24996
C 0.185883 4.072121 -0.416682
O 1.435102 3.444224 -0.140266
O -1.135848 2.286001 -1.43041
O -1.144179 2.995724 2.172847
O 0.505995 0.750812 -0.976721
H -1.878263 3.679981 -0.158439
H -1.062608 0.507028 -2.322319
H -1.023335 -0.608304 0.086421
H -2.887878 1.613189 0.873537
H 0.150593 1.785442 1.097302
H 0.156485 4.460273 -1.440455
H 0.025021 4.903844 0.280866
H -0.34337 3.415062 2.508931
H -1.909618 0.98521 2.888799
H 2.132656 4.10913 -0.192939
H 1.66427 0.471479 -2.674222
C -3.210195 -1.663487 -0.450105
H -3.442257 0.123748 -1.411644
O -2.552966 -2.327998 0.365117
C -4.401117 -2.252017 -1.152023
H -5.054954 -2.713968 -0.410827
H -4.042786 -3.041227 -1.818141
H -4.961883 -1.5196 -1.734023
Na -1.34036 -1.874415 2.167242

Transition state of 3rd m1_6 reaction of GlcNAcβ(1→2)Manβ
52

C 2.317359 0.753552 1.623096
C 1.316163 -0.361673 1.258235
C 1.926467 -1.701267 0.833496
O 0.933159 -2.706501 0.856609
C 2.59577 -1.708644 -0.554402
C 3.948609 -0.990822 -0.611204
C 3.904762 0.474406 -0.223359
O 3.087195 1.289951 -0.852416
O 4.849894 -1.586105 0.284274
O 2.892732 -3.035104 -0.915816
O 2.086472 1.93538 1.164139
H 4.318965 -1.051195 -1.646961
H 2.771807 0.672813 2.617584
H 2.678904 -1.990818 1.577349
H 1.898198 -1.271066 -1.289324
H 4.919683 -2.524963 0.05902
H 4.893917 0.874047 0.042173
H 3.455983 0.344458 0.980044
H 2.193867 -3.607965 -0.565257
H 0.128631 -2.378752 0.416677
H 2.470609 1.867261 0.066545
C -0.943209 0.010743 0.543952
C -1.741027 1.152877 -0.087974
N -1.331351 2.438197 0.433948
C -3.215136 0.906037 0.212193
O -3.957208 1.929408 -0.390455
C -3.651247 -0.47191 -0.276305
C -2.732395 -1.556139 0.301793
C -2.953513 -2.924074 -0.330432
O -4.329552 -3.223992 -0.197274
O -1.366592 -1.229097 0.012557
O -4.980923 -0.593421 0.158517
O 0.419726 0.130639 0.258246
H -2.884403 -1.613578 1.390628
H -1.097158 0.01878 1.636159
H -1.592825 1.147471 -1.174939
H -3.363582 0.916277 1.308598
H -3.582418 -0.495002 -1.377318
H -2.656644 -2.871622 -1.386849
H -2.328121 -3.667031 0.179614
H -5.302757 -1.484292 -0.045734
H -4.89601 1.713172 -0.290577
H -4.516791 -4.113414 -0.517221
H 0.744663 -0.562593 2.175188
C -0.401573 3.20765 -0.178239
H -1.931172 2.878794 1.11939
O 0.213002 2.807561 -1.172491
C -0.121302 4.554935 0.422378
H 0.841659 4.491398 0.937448
H -0.884718 4.87586 1.132249
H -0.030436 5.285197 -0.382324
Na 1.162545 1.059601 -1.931479

Reactant state of 3rd m1_6 reaction of GlcNAcβ(1→2)Manβ
52

C -2.389803 1.473112 -1.41559
C -1.413293 0.322174 -1.379542
C -2.094441 -1.055751 -1.237634
O -1.2225 -2.04342 -1.766776
C -2.540672 -1.502199 0.174064
C -3.851765 -0.918606 0.728911
C -3.860046 0.502576 1.283815
O -2.671109 0.821019 2.014766
O -4.865574 -0.933148 -0.251816
O -2.849704 -2.886655 0.097547
O -2.295134 2.451029 -0.708616
H -4.105465 -1.605112 1.550539
H -3.206529 1.379098 -2.152974
H -2.972437 -1.055424 -1.893858
H -1.718869 -1.331579 0.884822
H -5.089611 -1.853681 -0.444081
H -4.742245 0.627401 1.917907
H -3.94677 1.231008 0.479812
H -2.316195 -3.285782 -0.604128
H -0.436332 -2.064848 -1.194574
H -2.703827 0.399954 2.882023
C 0.849794 0.033411 -0.644124
C 1.950879 0.87784 0.003517
N 2.077835 2.187507 -0.607337
C 3.274429 0.134044 -0.143381
O 4.270287 0.903915 0.472771
C 3.193151 -1.26549 0.448551
C 2.024606 -2.023042 -0.188356
C 1.719401 -3.342217 0.511268
O 2.921647 -4.086769 0.534337
O 0.819971 -1.255737 -0.055574
O 4.440948 -1.843882 0.163747
O -0.40307 0.615645 -0.423285
H 2.249577 -2.198979 -1.252473
H 1.030518 -0.059669 -1.729892
H 1.717492 1.00756 1.067089
H 3.497149 0.010029 -1.220422
H 3.021695 -1.186189 1.535214
H 1.362636 -3.122076 1.526253
H 0.928821 -3.874623 -0.033146
H 4.429118 -2.774907 0.43182
H 5.083841 0.3784 0.485018
H 2.774125 -4.95893 0.916642
H -0.96743 0.304841 -2.389108
C 1.576403 3.306004 -0.025546
H 2.865109 2.318201 -1.230922
O 0.77485 3.241763 0.909376
C 2.054986 4.626874 -0.56219
H 1.203685 5.302698 -0.649277
H 2.558428 4.538938 -1.525818
H 2.750134 5.054688 0.165569
Na -1.103467 2.238544 1.228631
